# Piperidine scaffold as the novel P2-ligands in cyclopropyl-containing HIV-1 protease inhibitors: Structure-based design, synthesis, biological evaluation and docking study

**DOI:** 10.1371/journal.pone.0235483

**Published:** 2020-07-22

**Authors:** Huiyu Zhou, Mei Zhu, Ling Ma, Jinming Zhou, Biao Dong, Guoning Zhang, Shan Cen, Yucheng Wang, Juxian Wang

**Affiliations:** 1 Institute of Medicinal Biotechnology, Chinese Academy of Medical Science and Peking Union Medical College, Beijing, China; 2 Key Laboratory of the Ministry of Education for Advanced Catalysis Materials, Department of Chemistry, Zhejiang Normal University, Jinhua, China; University of East Anglia, UNITED KINGDOM

## Abstract

A series of potent HIV-1 protease inhibitors, containing diverse piperidine analogues as the P2-ligands, 4-substituted phenylsulfonamides as the P2’-ligands and a hydrophobic cyclopropyl group as the P1’-ligand, were designed, synthesized and evaluated in this work. Among these twenty-four target compounds, many of them exhibited excellent activity against HIV-1 protease with half maximal inhibitory concentration (IC_50_) values below 20 nM. Particularly, compound **22a** containing a (*R*)-piperidine-3-carboxamide as the P2-ligand and a 4-methoxylphenylsulfonamide as the P2’-ligand exhibited the most effective inhibitory activity with an IC_50_ value of 3.61 nM. More importantly, **22a** exhibited activity with inhibition of 42% and 26% against wild-type and Darunavir (DRV)-resistant HIV-1 variants, respectively. Additionally, the molecular docking of **22a** with HIV-1 protease provided insight into the ligand-binding properties, which was of great value for further study.

## Introduction

HIV-1 infection has become a serious threat to human beings around the world since the first case was reported in 1981 in the USA [1]. An estimated 37.9 million people lived with HIV-1 and 770 thousand people died from AIDS-related diseases in 2018, according to the Joint United Nations Programme on HIV/AIDS (UNAIDS)’s 2019 fact sheet on global HIV & AIDS statistics [2]. Fortunately, the emergence of a large variety of antiviral drugs, especially the application of HIV-1 protease inhibitors (PIs) in highly active antiretroviral therapy (HAART), made significant contributions to transforming HIV-1 infection from an inevitably fatal disease into a manageable chronic ailment [3, 4]. HIV-1 PIs serve as a critical therapeutic approach for the treatment of HIV-1 infection due to their ability to block the production of viral proteins for mature virions [4–6]. So the design of potent PIs continues to be essential for long-term control of HIV-1 infection and AIDS [7–10].

**Fig 1:**
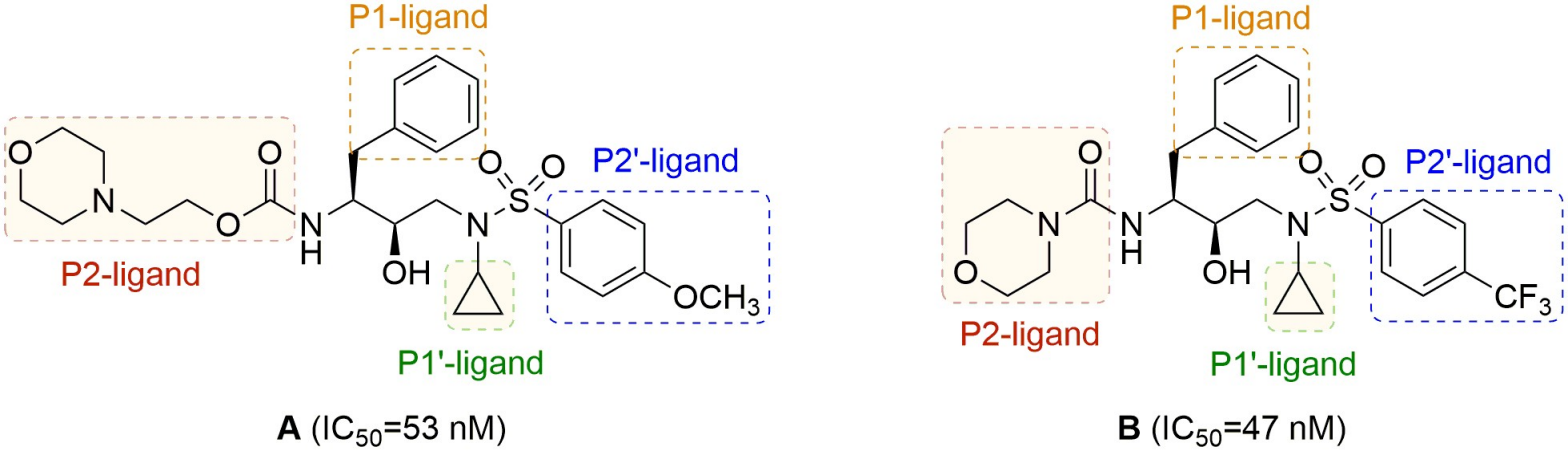
Structures of HIV-1 PIs A and B.

**Fig 2:**
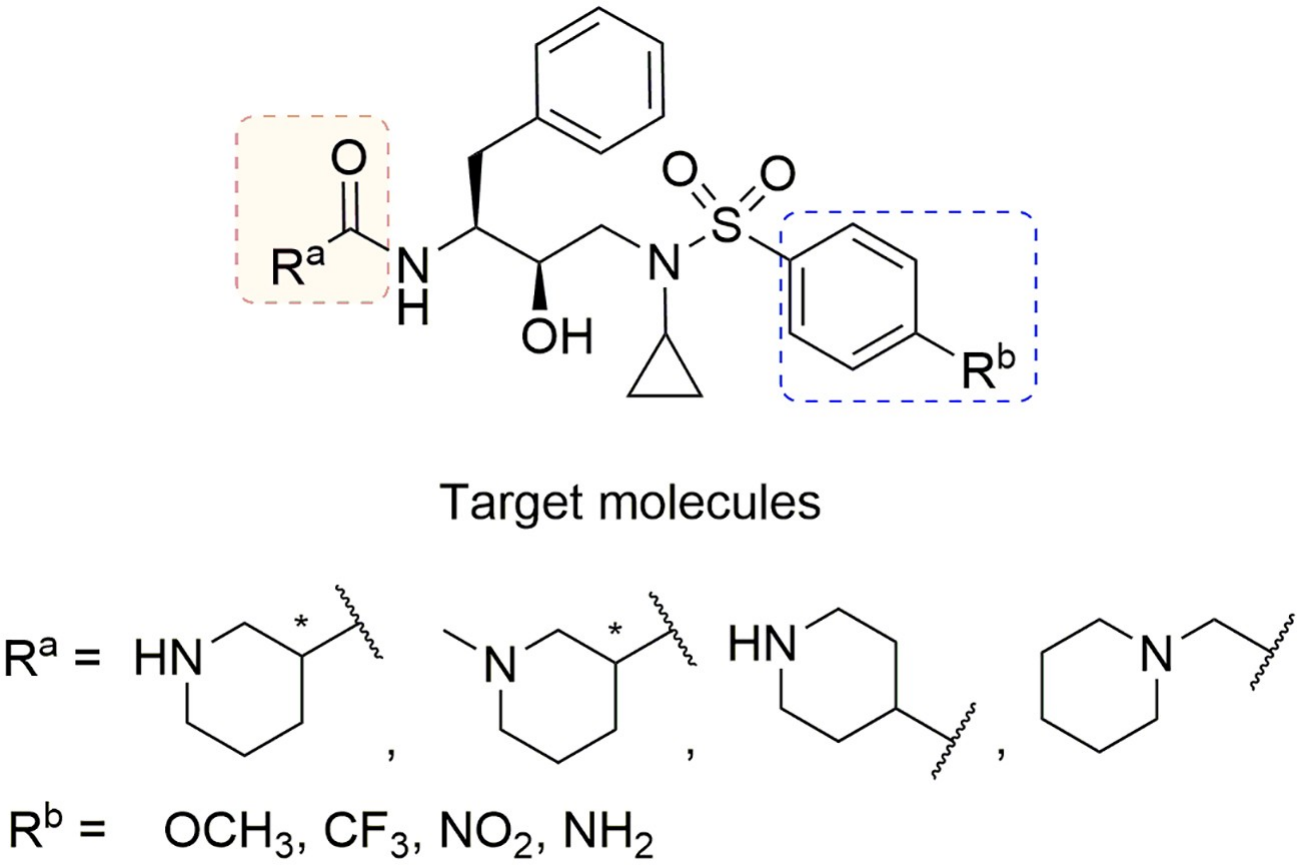
Chemical structures of target molecules.

In an effort to develop structurally novel PIs that exhibit potent inhibitory activity, one of the major design strategies is to optimize ligand-binding site interactions with the active site of HIV-1 protease (PR) [6, 11–15]. Recently, we reported a series of PIs incorporating a cyclopropyl as the P1’-ligand and morpholine derivatives as the P2-ligands [16]. Among which, compounds **A** and **B** in Fig 1 showed IC_50_ values of 53 nM and 47 nM, respectively. The molecular docking of compound **A** revealed that the small hydrophobic cyclopropyl group filled in the pocket of the S1 Appendix-subsite subtly [17–19]. However, the oxygen atom of morpholine in the P2-ligand formed weak van der Waals interaction with the backbone atoms, while the wrapped nitrogen atom failed to make contact with the active site, which might be amenable for the suboptimal activity. In view of the above phenomena, piperidine—a flexible heterocycle containing exposed nitrogen atom—was introduced as the P2-ligand in the newly designed HIV-1 PIs, with the aim of promoting extensive hydrogen bonding interactions or favorable van der Waals interactions with the backbone atoms in the corresponding S2 Appendix-subsite of PR. In addition, the effect of P2’-ligands incorporating functionalized 4-substituted phenylsulfonamides on HIV-1 protease inhibitory activity was investigated (Fig 2).

## Materials and methods

### Chemistry

The syntheses of substituted piperidine carboxylic acids **9–14** are shown in Scheme 1. All starting materials are commercially available. Chiral piperidine-3-carboxylic acids **1**, **2** and piperidine-4-carboxylic acid **3** were reacted with (Boc)_2_O in the presence of NaHCO_3_ to obtain Boc-protected amine derivatives **9–11** in excellent yields (92.5–97.3%) [20]. Reaction of the optically active ethyl piperidine-3-carboxylates **4**, **5** with formaldehyde and formic acid in methanol at reflux for 6 h afforded the corresponding derivatives **7**, **8** in yields of 95.6% and 93.4%, respectively [21]. Saponification of **7** or **8** with aqueous sodium hydroxide provided corresponding carboxylic acids **12** or **13** in nearly quantitative yield. Treatment of piperidine **6** with bromoacetic acid and potassium carbonate in anhydrous DMF furnished **14** in 83.0% yield [11].

**Scheme 1. Syntheses of substituted piperidine carboxylic acids 9–14.** Reagents and conditions: (a) (Boc)_2_O, NaHCO_3_, THF/H_2_O (1:1), Argon, 25 °C, overnight; (b) 40% formaldehyde, formic acid, MeOH, 0 °C to reflux, 6 h; (c) (ⅰ) NaOH, H_2_O, 25 °C, 1 h; (ⅱ) 1 N HCl, 0 °C, 0.5 h; (d) bromoacetic acid, K_2_CO_3_, anhydrous DMF, Argon, 25 °C, overnight.

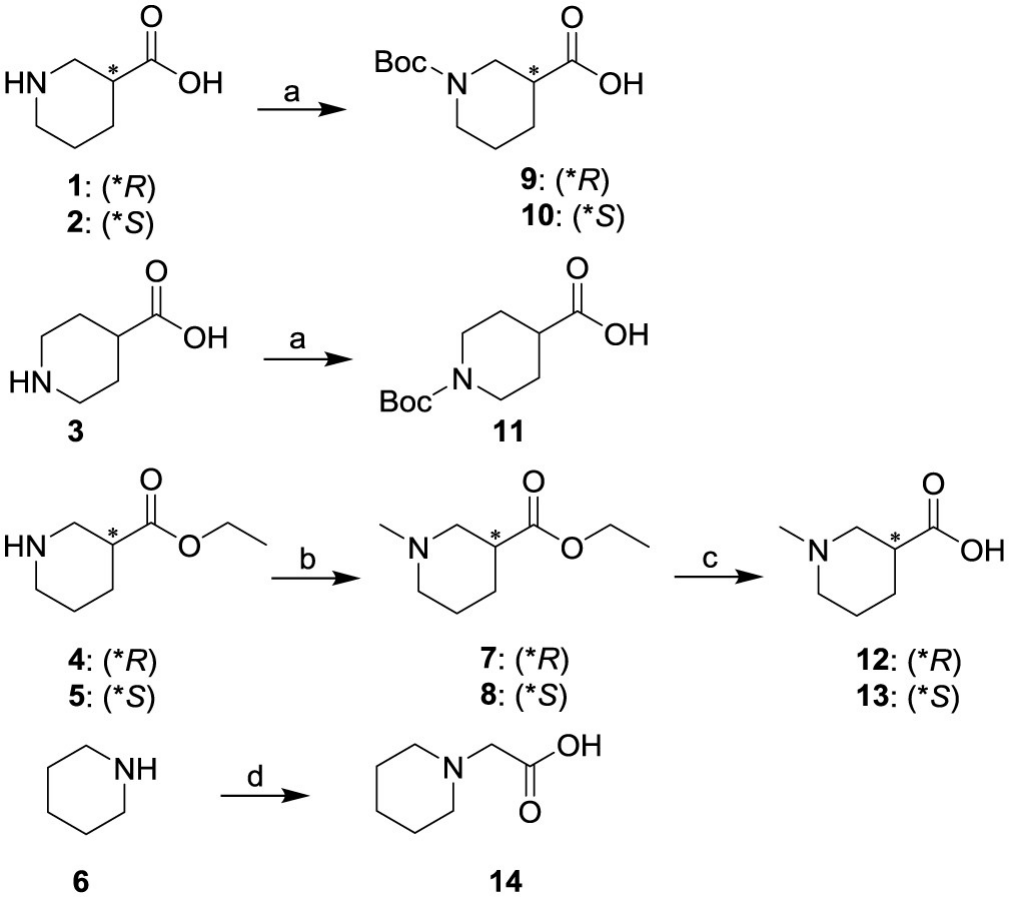


As depicted in Scheme 2, hydroxyethylamine sulfonamide isosteres **18a-d** were synthesized similarly according to the literature procedure [11, 16, 22]. Exposure of the commercially available epoxide **15** to cyclopropanamine in acetonitrile afforded amino alcohol **16** in 87.5% yield. Treatment of the resulting amino alcohol with 4-substituded-penzenesulfonly chlorides provided compounds **17a-c** in yields of 88.7–92.1%. They were subsequently converted to sulfonamide derivatives **18a-c** by deprotection of the Boc-group with trifluoroacetic acid in moderate yields (74.2–80.4%). Catalytic hydrogenation of **18c** over 10% Pd/C in a mixtre of ethyl acetate and methanol (1:2) for 4 h furnished aminosulfonamide derivate **18d** in 96.9% yield.

**Scheme 2. Syntheses of hydroxyethylamine sulfonamide isosteres 18a-d.** Reagents and conditions: (a) cyclopropanamine, acetonitrile, reflux, 7 h; (b) aryl sulfonyl chloride, DIEA, DMAP (Cat.), THF, 0–25 °C, overnight; (c) CF_3_COOH/CH_2_Cl_2_ (1:3), 25 °C, 5 h; (d) H_2_ (gas), 50 psi, 10% Pd/C, ethyl acetate/methanol (1:2), 25 °C, 4 h.

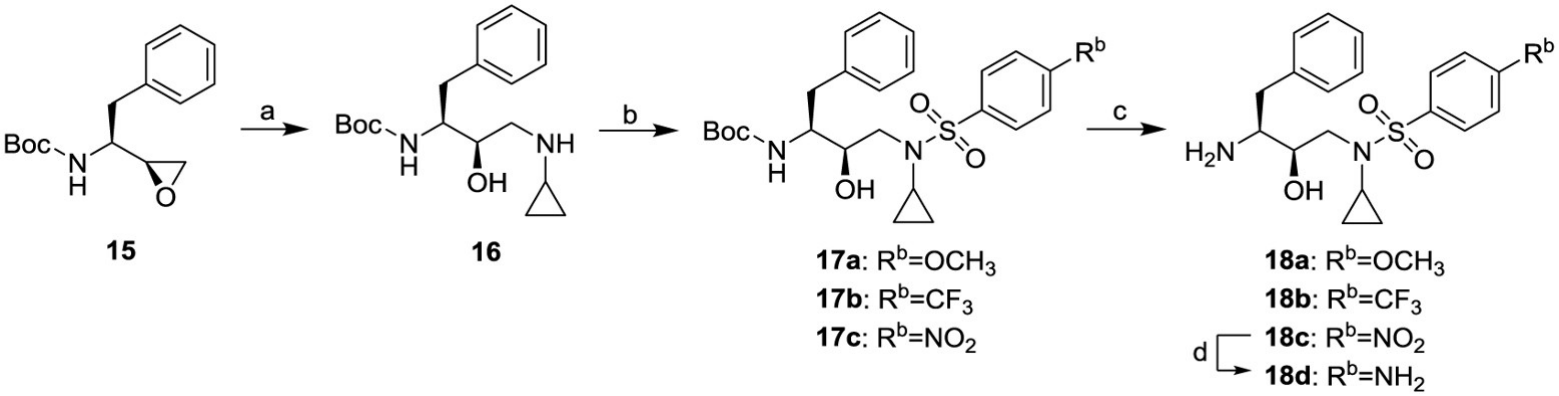


The syntheses of the target molecules **22a-27d** were illustrated in Scheme 3. Coupling of amines **18a-d** with acids **9–14** in the presence of EDCI, HOBt and catalytic amounts of DMAP obtained compounds **19a-21d**, **24a-25d** and **27a-d** in yields of 34.7–92.4%. Removal of the Boc-group by exposure of **19a-21d** to hydrogen chloride gas in CH_2_Cl_2_ provided corresponding piperidine derivatives **22a**-**23d** and **26a-d** in yields of 60.7–92.0%.

**Scheme 3. Syntheses of inhibitors 22a-27d.** Reagents and conditions: (a) EDCI, HOBt, DMAP, anhydrous DMF, Argon, 0–25 °C, 8 h; (b) HCl (gas), CH_2_Cl_2_, 25 °C, 0.5 h.

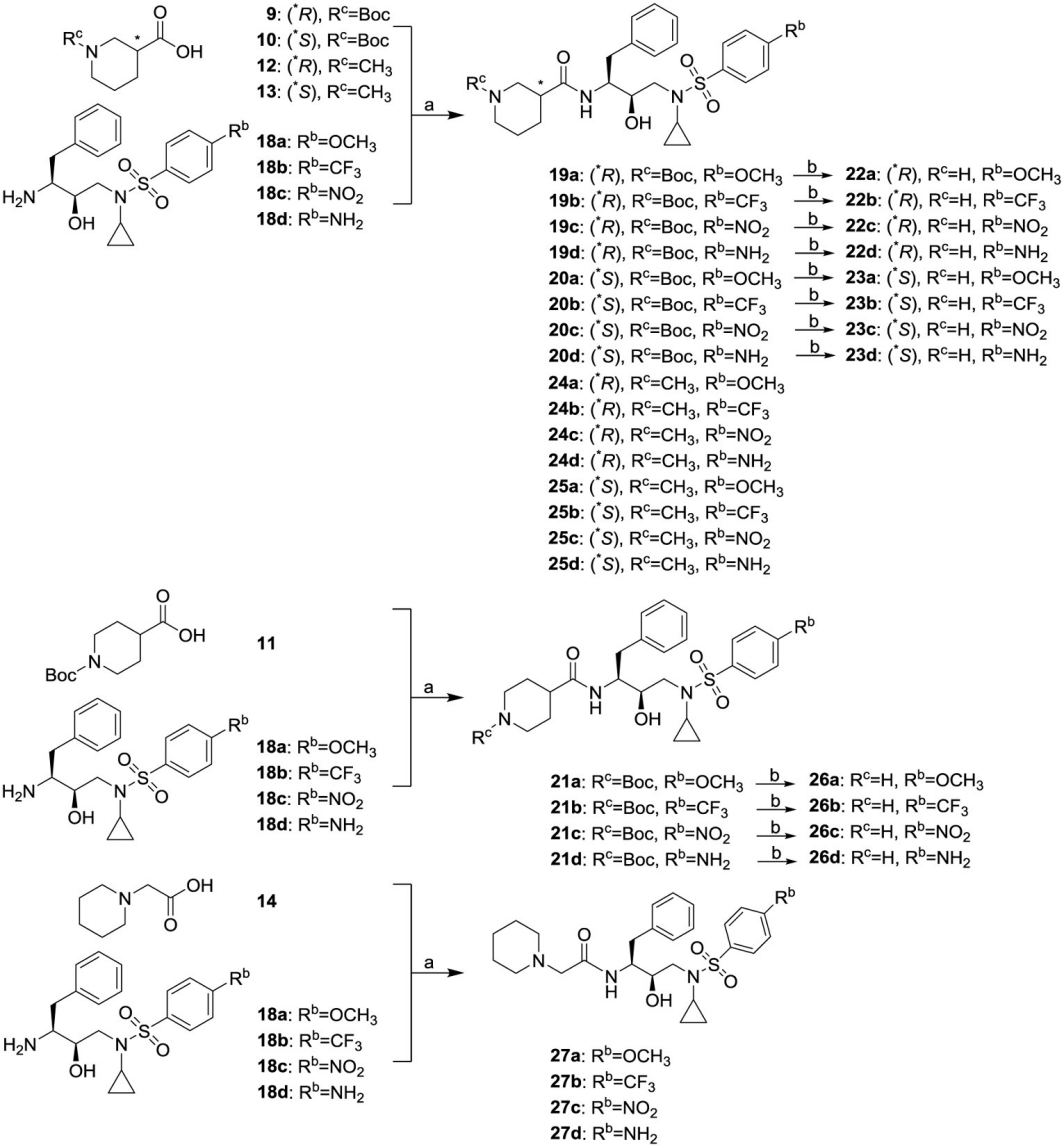


### *In vitro* HIV-1 PR activity assay

The inhibitory effect of all new designed inhibitors were measured using fluorescence resonance energy transfer (FRET) method. Peptide (Arg-Glu (EDANS)-Ser-Gln-Asn-Tyr-Pro-Ile-Val-Gln- Lys(DABCYL)-Arg) purchased from AnaSpec was selected as the substrate. The energy transfer donor (EDANS) and acceptor (DABCYL) dyes are labeled at two ends of the peptide to perform FRET. Excitation and emission wavelengths were set at 340 nm and 490 nm. Inhibitors were dissolved in dimethylsulfoxide (DMSO) and diluted to appropriate concentrations. HIV-1 protease was cloned and heterologously expressed in Escherichia coli and purified. The experiment was carried out in 96-well plates. The FRET assay reaction buffer contained 0.1 M sodium acetate, 1 M sodium chloride, 1 mM ethylenediaminetetraacetic acid (EDTA), 1 mM dithiothreitol (DTT), 2% DMSO and 1 mg/mL bovine serum albumin (BSA) with an adjusted pH 4.7. Protease and inhibitor were mixed and incubated for 20–30 mins at room temperature and then the substrate was added. Each reaction was recorded for about 10 mins. From plots of concentration versus the calculated percent inhibition, IC_50_ values were determined.

### HIV-1 infectivity assay

The inhibitory effect of compounds on HIV-1 infectivity were determined using a single-round HIV-1 infectivity assay. 293T cells were co-transfected with either plasmid pNL4-3-E^-^R^-^ (pHIV-1_NL4-3_) or DRV-resistant pNL4-3-E^-^R^-^ variants (pHIV-1_DRV_^R^_S_) and pHCMV-G (VSV-G) to produce VSV-G pseudotyped HIV-1. Inhibitors dissolved in dimethylsulfoxide (DMSO) and diluted to appropriate concentrations, were added into culture medium at 5 hours of post-transfection. After incubating for 48 hours at temperature 37 °C, pseudotyped viruses in 10 μL of supernatant were used to infect SupT1 cells for 48 hours, followed by measuring luciferase activity of newly infected cells using Centro LB960 (Berthold).

For the assay using wild type HIV-1, 1×10^6^ SupT1 cells were infected with 100 μL HIV-1 NL4-3 in the presence of 100 nM chemicals and 10 μg/mL polybrene, keeping a total volume of 500 μL (Spin infection at 1800rpm, 45min). Cells were washed once in the next morning and medium were replaced with fresh medium containing 100 nM chemicals. At 48 hpi, viruses were harvested and 50 μL of viruses were used to infect TZM-bl cells, followed by measuring luciferase activity in the infected cells.

### Cytotoxicity assay

Selected inhibitors were further evaluated in cytotoxicity assay using a cell counting kit-8 assay. Plates were prepared with 20 000 293T cells per well. After 24h of culture, 1μL of drugs were added to each well. After another 24h of culture, 10 μL of CCK-8 was added to each well. Absorbance was quantified at wavelength 450 nm using an EnVision multilabel reader (PerkinElmer) after 2h at room temperature. The 50% cytotoxic concentrations (CC_50_) were determined as the concentration required to reduce the number of the cells by 50% compared to that of drug-unexposed control cultures.

### Molecular docking

In general, the docking was performed through “DOCK” module in the Molecular Operating Environment (MOE) using the alpha triangle placement method. Refinement of the docked poses was carried out using the Forcefield refinement scheme and scored using both the affinity dG and london dG scoring system. The pose with the higher docking negative score implied better binding.

## Results and discussion

### Structure activity relationships

**Fig 3:**
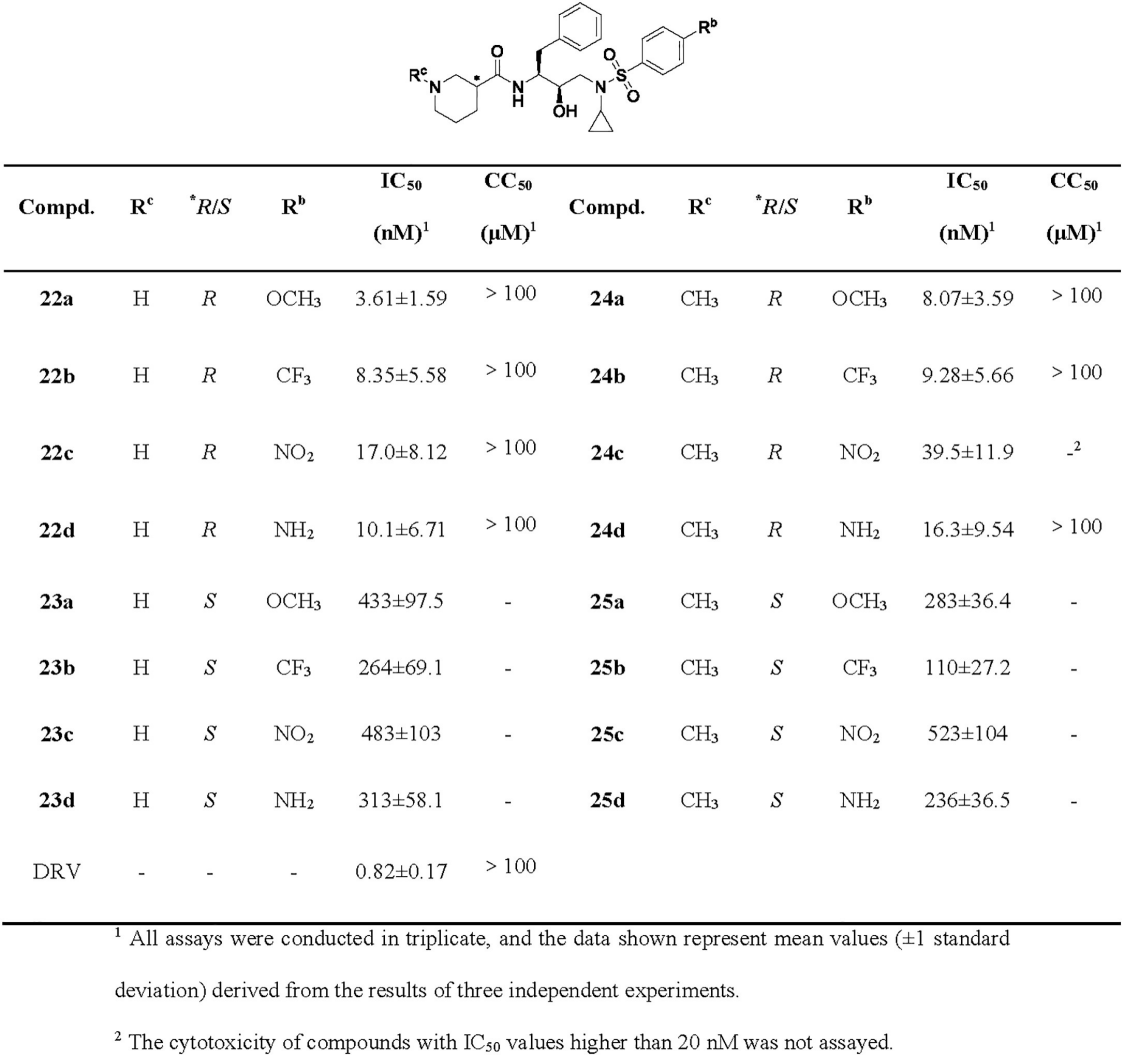
Enzymatic inhibitory activity and cytotoxicity of inhibitors 22a-25d.

**Fig 4:**
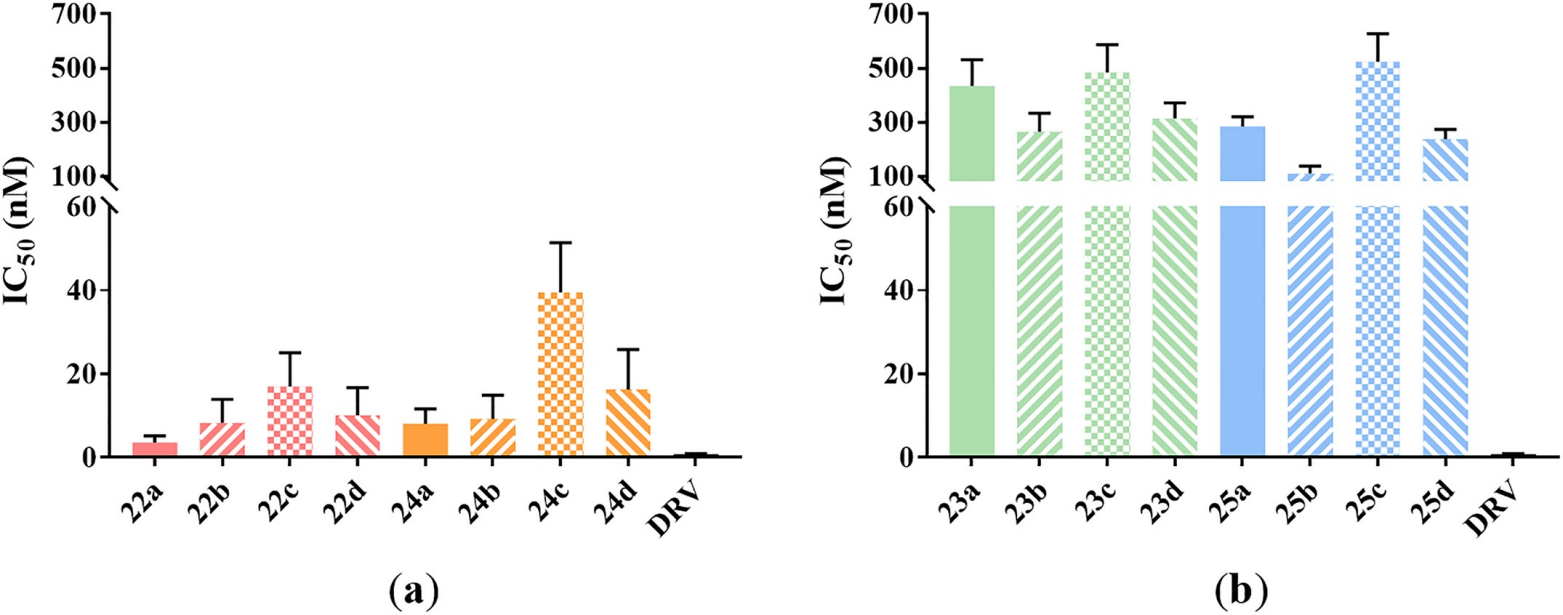
Enzymatic inhibitory activity of inhibitors. (a) Enzymatic inhibitory activity of inhibitors with (*R*)-piperidine derivatives as the P2-ligands; (b) Enzymatic inhibitory activity of inhibitors with (*S*)-piperidine derivatives as the P2-ligands.

**Fig 5:**
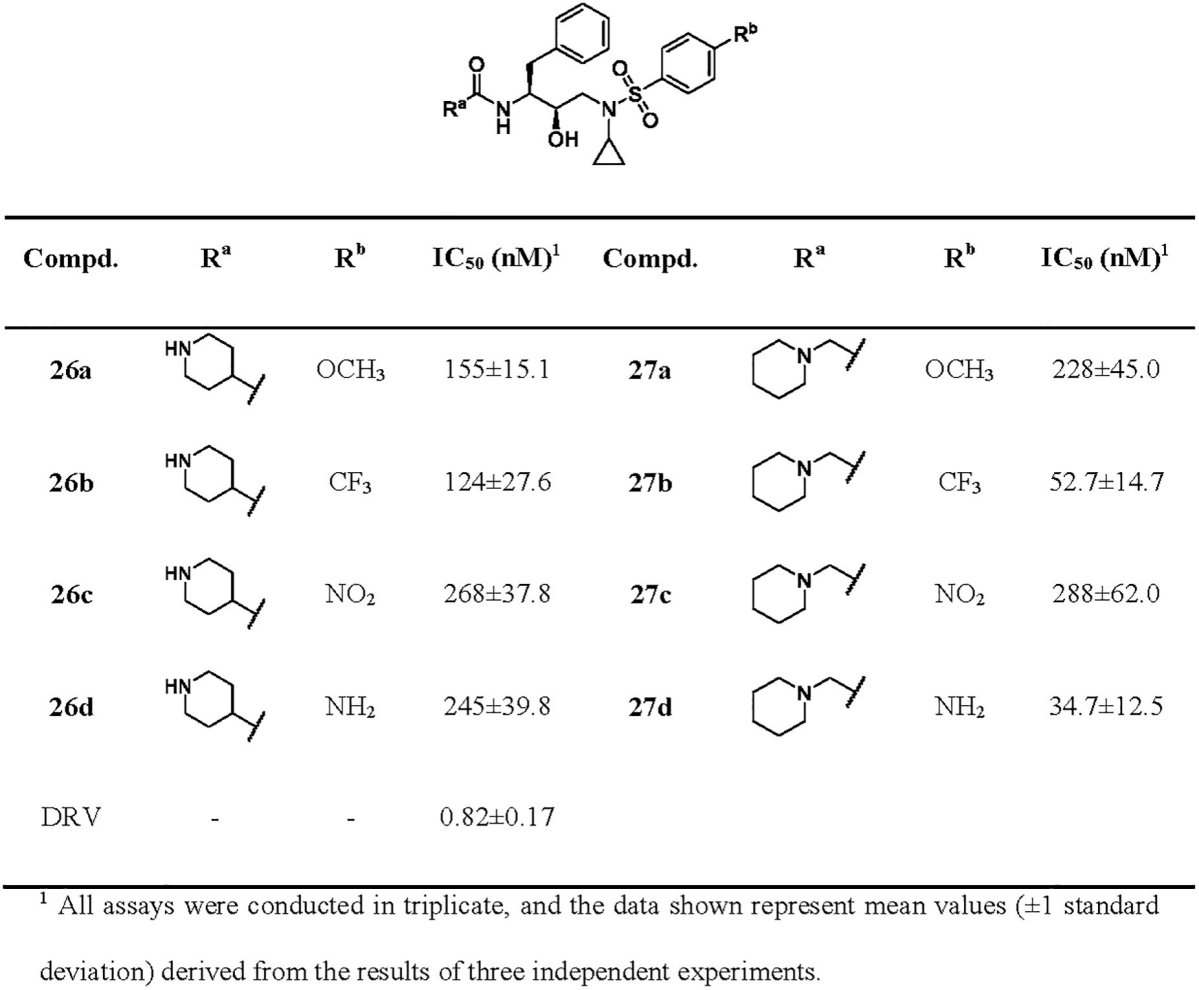
Enzymatic inhibitory activity of inhibitors 26a-27d.

All target compounds were evaluated the inhibitory potency against wild-type HIV-1 PR using the fluorescence resonance energy transfer (FRET) method, including DRV as the positive control [23, 24]. The results are presented in Figs 3–5. As can be seen, the piperidine-derived inhibitors exhibited potent enzymatic activity with IC_50_ values of submicromolar to nanomolar in general. Especially, inhibitor **22a**, containing a (*R*)-piperidine-3-carboxamide as the P2-ligand and a 4-methoxylphenylsulfonamide as the P2’-ligand, displayed the most impressive activity with an IC_50_ value of 3.61 nM.

As it turned out in Figs 3 and 4, inhibitors with (*R*)-piperidine derivatives as the P2-ligands exhibited superior activity than those with (*S*)-configuration as a whole. For instance, compound **22a** showed a 120-fold improvement of potency over **23a**, which revealed the significance of configurations. In addition, the activity decreased when the nitrogen atom of (*R*)-piperidine scaffold was methylated, such as **22a** *vs* **24a**, **22b** *vs* **24b**, **22c** *vs* **24c**, and **22d** *vs* **24d**. The results might be attributed to both the smaller volume of the P2-ligand which was more suitable for the cavity of S2 Appendix-subsite and the capacity of the exposed nitrogen atom which could form hydrogen bonding interactions with the carbonyl oxygen or amide NHs of Asp30 and Asp29 [25–28].

The functional P2’-ligands also exerted great impact on the potency of inhibitors. Compounds containing 4-methoxyl (**22-25a**), 4-trifluoromethyl (**22-25b**) and 4-amino (**22-25d**) substituents exhibited more potent enzyme inhibitory than the corresponding 4-nitro substituent compounds (**22-25c**). The oxygen atom of methoxyl group is capable of forming hydrogen bonds with the backbone NH and side-chain carboxylate of Asp30’ directly, which could enhance the antiviral potency [29]. The 4-trifluoromethyl group in the P2’-ligand may cause favorable halogen interactions and van der Waals interactions with the P2’-pocket [30, 31], in spite of its weak electron-withdrawing inductive effect that impaired the binding affinity slightly [15]. Although the amino of 4-aminobenzene sulfonamide could participate in direct or water-mediated hydrogen bonds with Asp30’, these interactions were weaker than those between 4-methoxybenzene sulfonamide and the cavity of S2 Appendix-subsite [10]. On the contrary, the strong electron-withdrawing property of nitro in the P2’-ligand, including both electron-withdrawing inductive effect and conjugation, was likely to weaken not only the hydrogen bonds between the nitro oxygen and Asp30’, but also the water-mediated interactions between the sulfonyl oxygen and Ile50’ [19, 15].

Furthermore, the cytotoxicity of selected inhibitors was assayed [32]. Surprisingly, all of them exhibited low cytotoxicity. Therefore, this kind of inhibitors with potent activity and low toxicity deserved in-depth study.

However, compounds **26a** and **27a** bearing 4- or 1-subsituted piperdine derivatives in Fig 5 showed more than 50-fold loss of potency over the 3-subsituted piperidine derivative **22a**. The same trend can also be observed in the other three groups (**22** *vs* **26**, **27b**; **22** *vs* **26**, **27c**; **22** *vs* **26**, **27d**), which suggested that the shift of substituent position and the length of linker can impact the activity remarkably. Moreover, the effect of functional P2’-ligands on potency is similar to that shown in Fig 3.

### HIV-1 infectivity assay

**Fig 6:**
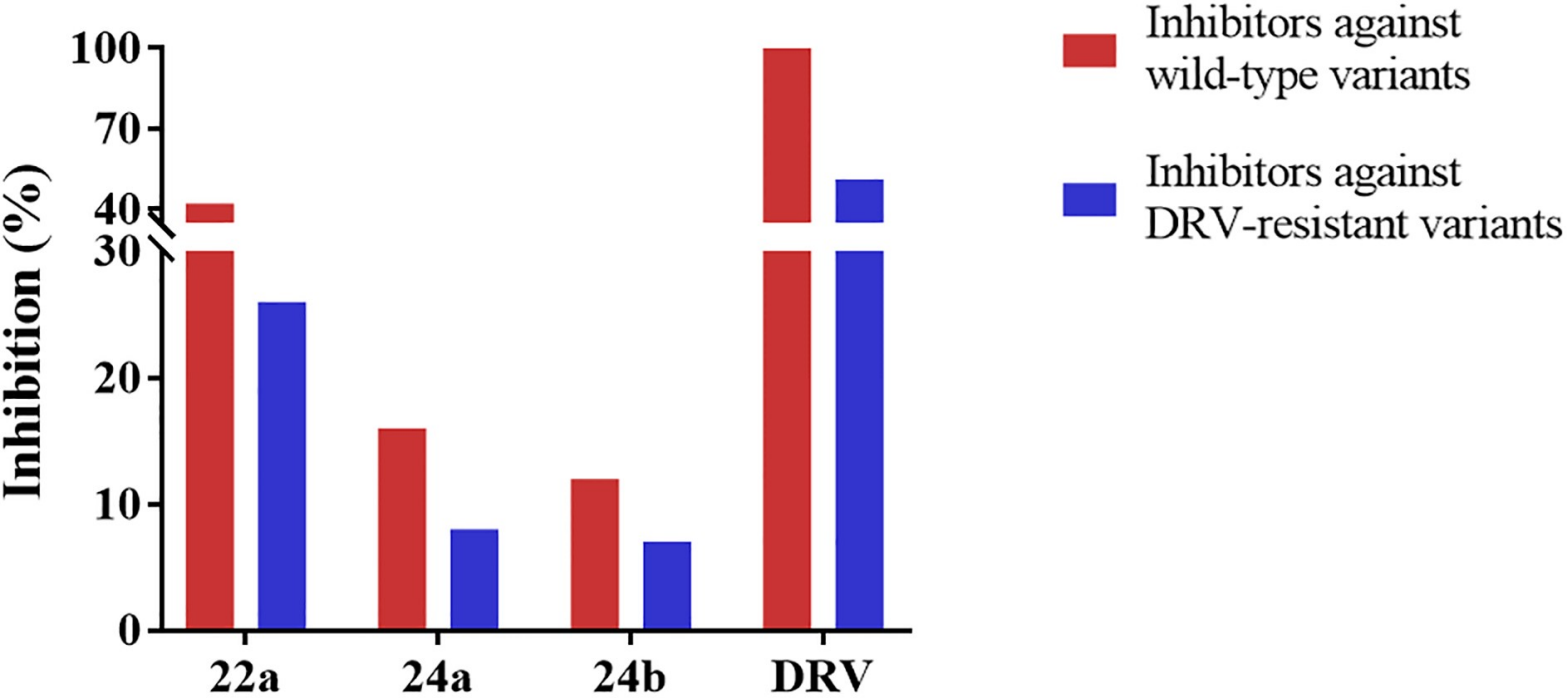
Inhibition of inhibitors against wild-type and DRV-resistant HIV-1 variants. Inhibition of inhibitors 22a, 24a and 24b was conducted at the concentration of 10 μM, and DRV at 20 nM.

In the assays against HIV-1 wild-type and DRV-resistant variants [33, 34], selected compounds **22a**, **24a** and **24b** exhibited inhibition activity to some extent (Fig 6). Notably, **22a** exhibited the most remarkable activity with inhibition of 42% and 26% against wild-type and DRV-resistant HIV-1 variants, respectively, which agreed with the activity tested *in vitro*. Although compounds **24a** and **24b** showed inconspicuous inhibition (with 16% and 12% against wild-type variants, and 8% and 7% against DRV-resistant variants, respectively), there still revealed regularity. Generally, compounds with 4-methoxylphenylsulfonamide as the P2’-ligand exhibited superior activity than those with 4-trifluoromethylphenylsulfonamide as the P2’-ligand *in vivo*, which pointed the way for further study.

### Molecular docking

**Fig 7:**
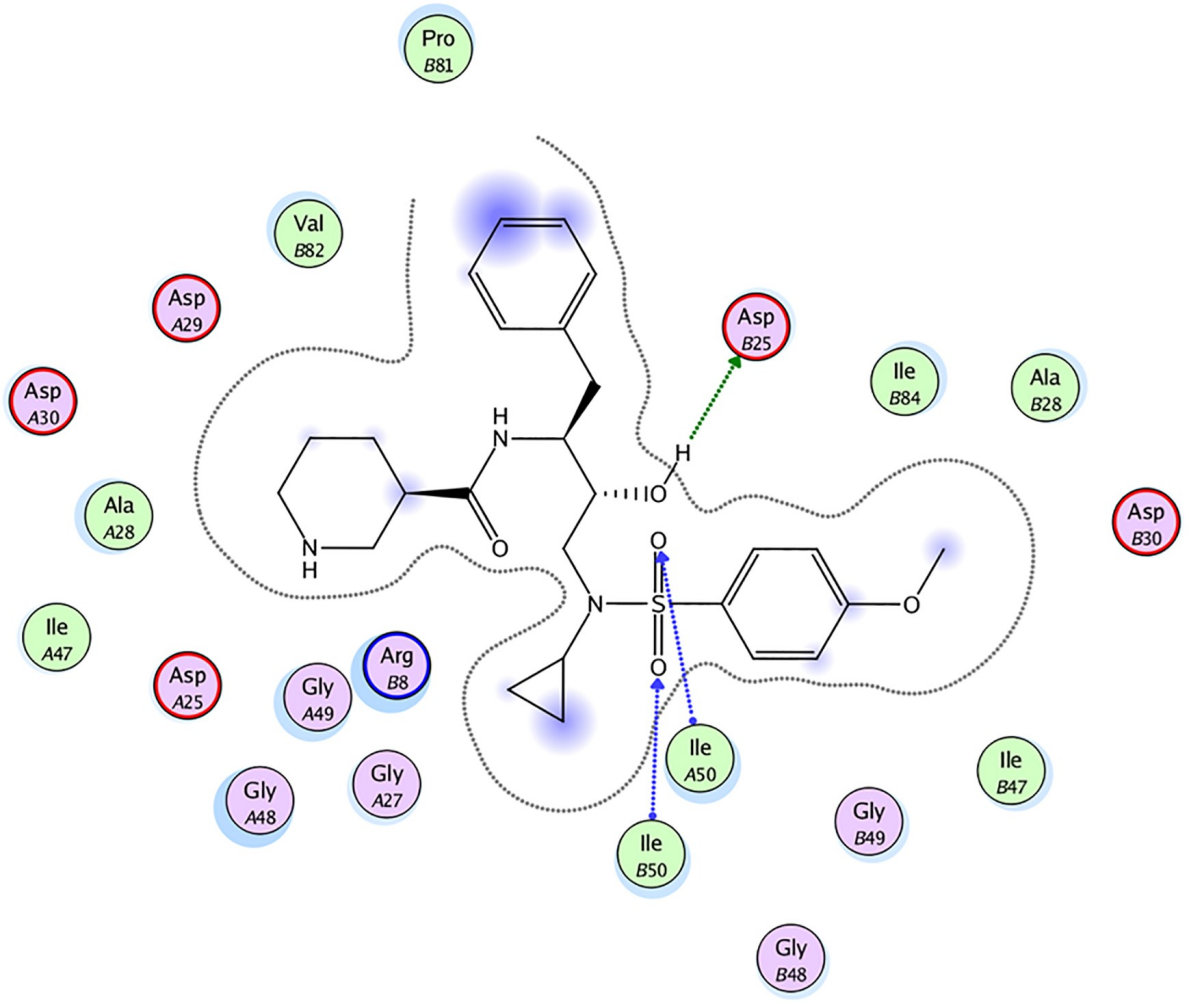
Docking of 22a in HIV-1 PR. Ligand exposures are described as purple spheres. Hydrogen bonding interactions between ligands and residues are represented as blue or green arrows.

In order to gain insight into the ligand-binding site interactions, the molecular modeling for **22a** was done in the molecular modeling software MOE with a HIV-1 PR crystal structure (PDB-ID: 4mc9) [35]. Remarkably, **22a** fitted into the active site of PR perfectly. As illustrated in Fig 7, several hydrogen bonding interactions were formed between the scaffold of the inhibitor and the residues Asp25, Ile50 (A chain) and Ile50 (B chain). Furthermore, plentiful van der Waals interactions between atoms in the P2-ligand, P1-ligand, P1’-ligand or P2’-ligand and external enzyme atoms were also observed. Especially, the newly introduced piperidine could produce favorable interactions with the active site of PR and be inserted into the cavity of S2 Appendix-subsite properly. All of the above mentioned might be responsible for the promising inhibitory activity of **22a**.

## Conclusions

In summary, we have reported the structure-based design and synthesis of a series of novel HIV-1 PIs incorporating flexible piperidine moieties as the P2-ligands, 4-substituted phenylsulfonamides as the P2’-ligands and a cyclopropyl group as the P1’-ligand. Introduction of poperidine in the P2-ligand was for the sake of promoting hydrogen bonding or van der Waals interactions with the active site of HIV-1 PR backbone. A number of inhibitors exhibited excellent potency and low cytotoxicity. In particular, inhibitor **22a** containing a (*R*)-piperidine-3-carboxamide as the P2-ligand and a 4-methoxylphenylsulfonamide as the P2’-ligand showed the most remarkable enzyme inhibitory activity, with an IC_50_ value of 3.61 nM, as well as activity with inhibition of 42% and 26% against wild-type and DRV-resistant HIV-1 variants, respectively. We demonstrated that the stereochemistry and substitution position of piperidine derivatives in the P2-ligands, as well as functional phenylsulfonamides in the P2’-ligands, are decisive for the potency. Moreover, the molecular docking of **22a** showed that the piperidine could fill in the pocket of S2 Appendix-subsite perfectly and make strong interactions with residues of HIV-1 PR, which was consistent with its potent antiviral activity. Further studies on the evaluation of other flexible *N*-containing heterocycle derivatives are currently in progress.

## Supporting information

S1 Appendix Description of synthetic experiments. (DOCX)

S2 Appendix ^1^H NMR, ^13^C NMR and HR MS spectrums of compounds. (DOCX)

S3 Appendix Description of biological evaluation and docking study. (DOCX)
