# Supplementary material for: Piperidine scaffold as the novel P2-ligands in cyclopropyl-containing HIV-1 protease inhibitors: Structure-based design, synthesis, biological evaluation and docking study
Source: PLoS One. 2020 Jul 22;15(7):e0235483. doi: 10.1371/journal.pone.0235483 (PMC7375528; doi:10.1371/journal.pone.0235483)
Supplement: S1 Appendix — (DOCX) [file pone.0235483.s001.docx]

**S1** **Appendix. Description of synthetic experiments.**

All experiments requiring anhydrous conditions were conducted in flame-dried glassware fitted with rubber septa under a positive pressure of dry argon, unless otherwise noted. THF was distilled under argon from sodium-benzophenone ketyl and CH_2_Cl_2_ was distilled under argon from calcium hydride. All reactions were monitored by thin-layer chromatography on silica gel plates (GF-254) and visualized with the UV light. Melting points were taken on MP70 Melting Point System with revised. High resolution mass spectra were obtained on an Autospee Ultima-TOF spectrometer. ^1^H NMR and ^13^C NMR spectra were recorded in CDCl_3_, CD_3_OD or DMSO-d_6_ on a Bruker AVANCE III 400 MHz, 500 MHz or 600 MHz spectrometer (Bruker Inc) with tetramethylsilane (TMS) as an internal reference. The chemical shifts are given in δ (ppm) referenced to the respective solvent peak (CDCl_3_: ^1^H, δ = 7.26 ppm, ^13^C, δ = 77.16 ppm; CD_3_OD: ^1^H, δ = 3.31 ppm, ^13^C, δ = 49.00 ppm; DMSO-d_6_: ^1^H, δ = 2.49 ppm, ^13^C, δ = 39.5 ppm), and coupling constants are reported in Hz. Almost target compounds were characterized by ^1^H and ^13^C NMRs and HRMS spectra.

1. Ethyl (*R*)-1-methylpiperidine-3-carboxylate (**7**)

Ehyl (*R*)-piperidine-3-carboxylate (**4**, 0.25 g, 1.59 mmol) was dissolved in methanol (4 mL), 40 percent formaldehyde (0.60 mL, 7.95 mmol) and formic acid (0.12 mL, 3.18 mmol) were slowly added in sequence at 0 ℃. The mixture was refluxed for 6 hours, then cooled and brought to a pH in the region of 9 using saturated aqueous Na_2_CO_3_ solution. The solution was concentrated under reduced pressure, added H_2_O (15 mL) and extracted with dichloromethane (3×15 mL). The organic phase was dried over anhydrous Na_2_SO_4_ and concentrated under reduced pressure to afford **7** in the form of a yellowish oil: yield 0.26 g (95.6%); ^1^H NMR (500 MHz, CD_3_OD) δ 4.15 (q, *J* = 7.0 Hz, 2H), 2.97 (d, *J* = 10.0 Hz, 1H), 2.76 (d, *J* = 10.0 Hz, 1H), 2.64 – 2.55 (m, 1H), 2.30 (s, 3H), 2.22 – 2.13 (m, 1H), 2.09 – 2.01 (m, 1H), 1.99 – 1.92 (m, 1H), 1.81 – 1.74 (m, 1H), 1.66 – 1.57 (m, 1H), 1.49 – 1.39 (m, 1H), 1.27 (t, *J* = 7.0 Hz, 3H); LC-MS (ESI) [M+H]^+^ m/z 172.4.

2. Ethyl (*S*)-1-methylpiperidine-3-carboxylate (**8**)

Compound **8** was prepared from ethyl (*S*)-piperidine-3-carboxylate (**5**, 0.25 g, 1.59 mmol) by following the same procedure outlined for **7** to give a yellowish oil: yield 0.25 g (93.4%));^1^H NMR (500 MHz, CD_3_OD) δ 4.15 (q, *J* = 7.0 Hz, 2H), 2.97 (d, *J* = 10.0 Hz, 1H), 2.77 (d, *J* = 10.0 Hz, 1H), 2.64 – 2.56 (m, 1H), 2.31 (s, 3H), 2.24 – 2.15 (m, 1H), 2.06 (t, *J* = 10.0 Hz, 1H), 1.99 – 1.92 (m, 1H), 1.81 – 1.75 (m, 1H), 1.67 – 1.57 (m, 1H), 1.49 – 1.40 (m, 1H), 1.27 (t, *J* = 7.0 Hz, 3H); LC-MS (ESI) [M+H]^+^ m/z 172.4.

3. (*R*)-1-(*tert*-butoxycarbonyl)piperidine-3-carboxylic acid (**9**)

To a stirred solution of (*R*)-piperidine-3-carboxylic acid (**1**, 0.5 g, 3.87 mmol) in THF/H_2_O (10 mL/10 mL) was added (Boc)_2_O (1.18 g, 5.42 mmol) and NaHCO_3_ (0.45 g, 5.42 mmol). The reaction was stirred overnight at 25 ℃ under argon. The mixture was diluted with H_2_O (5 mL) and petroleum ether (5 mL), then the aqueous layer was acidified with 1N HCl to pH 2 and the product was extracted with ethyl acetate (3×20 mL). The organic phase was washed with saturated aqueous NaCl solution, dried over anhydrous Na_2_SO_4_ and concentrated under reduced pressure to afford **9** as a white powder: yield 0.82 g (92.5%); ^1^H NMR (500 MHz, CDCl_3_) δ 4.30 – 3.99 (m, 1H), 3.88 (d, *J* = 12.5 Hz, 1H), 3.16 – 2.94 (m, 1H), 2.85 (t, *J* = 11.5 Hz, 1H), 2.53 – 2.44 (m, 1H), 2.06 (d, *J* = 13.5 Hz, 1H), 1.71 (d, *J* = 11.5 Hz, 1H), 1.65 (d, *J* = 11.5 Hz, 1H), 1.53 – 1.47 (m, 1H), 1.45 (s, 9H); LC-MS (ESI) [M-H]^-^ m/z 228.3.

4. (*S*)-1-(*tert*-butoxycarbonyl)piperidine-3-carboxylic acid (**10**)

Compound **10** was prepared from (*S*)-piperidine-3-carboxylic acid (**2**, 0.5 g, 3.87 mmol) by following the same procedure outlined for **9** to give a white powder: yield 0.84 g (94.8%); ^1^H NMR (500 MHz, CDCl_3_) δ 4.27 – 3.96 (m, 1H), 3.88 (d, *J* = 13.0 Hz, 1H), 3.18 – 2.91 (m, 1H), 2.86 (t, *J* = 12.0 Hz, 1H), 2.54 – 2.42 (m, 1H), 2.06 (d, *J* = 12.0 Hz, 1H), 1.77 – 1.69 (m, 1H), 1.68 – 1.59 (m, 1H), 1.52 – 1.47 (m, 1H), 1.45 (s, 9H); LC-MS (ESI) [M-H]^-^ m/z 228.3.

5. 1-(*Tert*-butoxycarbonyl)piperidine-4-carboxylic acid (**11**)

Compound **11** was prepared from piperidine-4-carboxylic acid (**3**, 0.45 g, 3.50 mmol) by following the same procedure outlined for **9** to give a white powder: yield 0.78 g (97.3%); ^1^H NMR (500 MHz, CDCl_3_) δ 4.02 (s, 2H), 2.85 (t, *J* = 12.5 Hz, 2H), 2.49 (t, *J* = 9.5 Hz, 1H), 1.90 (d, *J* = 13.0 Hz, 2H), 1.64 (q, *J* = 12.0 Hz, 2H), 1.45 (s, 9H); LC-MS (ESI) [M-H]^-^ m/z 228.4.

6. (*R*)-1-methylpiperidine-3-carboxylic acid (**12**)

NaOH (0.14 g, 3.60 mmol) was dissolved in H_2_O (4 mL) and then added dropwise into ethyl (*R*)-1-methylpiperidine-3-carboxylate (**7**, 0.20 g, 1.20 mmol). The reaction was stirred for 1 hour at 25 ℃. The mixture was acidified to pH 2 with 1 N HCl and stirred for 0.5 hour at 0 ℃. After this period, the reaction mixture was washed with petroleum ether and concentrated under reduced pressure. The solid was dissolved with anhydrous methanol, filtered through Celite 545^®^ and concentrated to afford **12** in the form of a white powder: yield 0.17 g (99.1%); ^1^H NMR (500 MHz, CD_3_OD) δ 3.75 – 3.66 (m, 1H), 3.53 – 3.38 (m, 1H), 3.21 – 3.00 (m, 2H), 2.90 (s, 3H), 2.26 – 2.13 (m, 1H), 2.08 – 2.00 (m, 1H), 1.95 – 1.71 (m, 2H), 1.63 – 1.52 (m, 1H); LC-MS (ESI) [M-H]^-^ m/z 142.4.

7. (*S*)-1-methylpiperidine-3-carboxylic acid (**13**)

Compound **13** was prepared from ethyl (*S*)-1-methylpiperidine-3-carboxylate (**8**, 0.20 g, 1.20 mmol) by following the same procedure outlined for **12** to give a white powder: yield 0.17 g (99.6%); ^1^H NMR (500 MHz, CD_3_OD) δ 3.73 – 3.67 (m, 1H), 3.54 – 3.38 (m, 1H), 3.25 – 2.93 (m, 2H), 2.90 (s, 3H), 2.26 – 2.11 (m, 1H), 2.08 – 1.96 (m, 1H), 1.95 – 1.67 (m, 2H), 1.65 – 1.50 (m, 1H); LC-MS (ESI) [M-H]^-^ m/z 142.4.

8. 2-(Piperidin-1-yl)acetic acid (**14**)

To a stirred solution of piperidine (**6**, 0.13 g, 1.50 mmol) and K_2_CO_3_ (0.52 g, 3.75 mmol) in anhydrous DMF (3.5 mL) was added bromoacetic acid (0.23 g, 1.80 mmol) dissolved in 2 mL anhydrous DMF under argon atmosphere. The mixture was stirred at 25 ℃ overnight. After this period, the reaction mixture was diluted with H_2_O (5 mL) and filtered through Celite 545^®^. The filtrate was acidified to pH 2 with 1 N HCl in an ice bath. The solvent was removed under reduced pressure. The obtained solid was dissolved with anhydrous methanol and filtered through Celite 545^®^. The filtrate was concentrated under reduced pressure to obtain **14** in the form of a yellow oil: yield 0.18 g (83.0%); ^1^H NMR (500 MHz, CD_3_OD δ 3.96 (s, 2H), 3.57 (s, 2H), 3.00 (s, 2H), 1.86 (s, 5H), 1.51 (s, 1H); LC-MS (ESI) [M+H]^+^ m/z 144.3.

9. *Tert*-butyl ((2*S*,3*R*)-4-(cyclopropylamino)-3-hydroxy-1-phenylbutan-2-yl)carbamate (**16**)

To a suspension of (2*S*, 3*S*) -1, 2-epoxy-3-(boc-amino)-4-phenylbutane (**15**, 5.00 g, 19.0 mmol) in acetonitrile (20 mL) was added cyclopropanamine (2.70 g, 47.4 mmol). The reaction was refluxed for 7 hours, and the solvent was removed under reduced pressure. The solid product was then suspended in petroleum ether (25 mL) and stirred for 0.5 hour. The precipitate was isolated by filtration and dried, *in vacuo*, over P_2_O_5_ to give **16** as a white powder: yield 5.32 g (87.5%); ^1^H NMR (500 MHz, CDCl_3_) δ 7.38 – 7.28 (m, 3H), 7.27 – 7.22 (m, 2H), 4.62 (s, 1H), 3.81 (s, 1H), 3.52 (s, 1H), 3.12 – 2.62 (m, 5H), 2.17 (s, 1H), 1.39 (s, 9H), 0.49 (s, 2H), 0.38 (s, 2H); LC-MS (ESI) [M+H]^+^ m/z 321.5.

10. *Tert*-butyl ((2*S*,3*R*)-4-((*N*-cyclopropyl-4-methoxyphenyl)sulfonamido)-3-hydroxy-1-phenylbutan-2-yl)carbamate (**17a**)

To a cold (0 ℃) solution of *tert*-butyl ((2*S*,3*R*)-4-(cyclopropylamino)-3-hydroxy-1-phenylbutan-2-yl)carbamate (**16**, 1.60 g, 5.00 mmol) in THF (20 mL) was added DIEA (0.71 g, 5.50 mol) and DMAP (0.06 g, 0.50 mmol). 4-methoxybenzenesulfonyl chloride (1.13 g, 5.50 mmol) dissolved in 10 mL THF was added slowly to the reaction. The mixture was stirred at 25 ℃ overnight. The solvent was removed under reduced pressure. The residue was diluted with ethyl acetate (30 mL) and washed with H_2_O and saturated aqueous NaCl solution, and then dried over anhydrous Na_2_SO_4_. The reaction mixture was concentrated under reduced pressure, and the residue was purified by silica gel column chromatography (hexanes/ethyl acetate, 2:1) to furnish **17a** as a white solid: yield 2.21 g (90.3%); ^1^H NMR (400 MHz, CDCl_3_) δ 7.81 – 7.71 (m, 2H), 7.34 – 7.27 (m, 2H), 7.26 – 7.17 (m, 3H), 7.02 – 6.94 (m, 2H), 4.69 (d, *J* = 8.0 Hz, 1H), 3.94 – 3.85 (m, 4H), 3.79 (s, 1H), 3.26 – 3.12 (m, 2H), 3.01 (dd, *J* = 14.0, 5.0 Hz, 1H), 2.90 (dd, *J* = 24.0, 8.0 Hz, 1H), 2.08 – 1.99 (m, 1H), 1.36 (s, 9H), 0.91 – 0.81 (m, 2H), 0.72 – 0.63 (m, 2H); LC-MS (ESI) [M+Na]^+^ m/z 513.5.

11. *Tert*-butyl ((2*S*,3*R*)-4-((*N*-cyclopropyl-4-(trifluoromethyl)phenyl)sulfonamido)-3-hydroxy-1-phenylbutan-2-yl)carbamate (**17b**)

Compound **17b** was prepared from *tert*-butyl ((2*S*,3*R*)-4-(cyclopropylamino)-3-hydroxy-1-phenylbutan-2-yl)carbamate (**16**, 1.60 g, 5.00 mmol) and 4-(trifluoromethyl)benzenesulfonyl chloride (1.35 g, 5.50 mmol) by following the same procedure outlined for **17a** to give a white powder: yield 2.34 g (88.7%); ^1^H NMR (600 MHz, CDCl_3_) δ 7.96 (d, *J* = 6.0 Hz, 2H), 7.79 (d, *J* = 6.0 Hz, 2H), 7.34 – 7.28 (m, 2H), 7.26 – 7.21 (m, 3H), 4.67 (d, *J* = 8.4 Hz, 1H), 3.99 – 3.89 (m, 1H), 3.84 – 3.75 (m, 1H), 3.60 (s, 1H), 3.32 – 3.26 (m, 1H), 3.25 – 3.19 (m, 1H), 3.05 – 2.96 (m, 1H), 2.95 – 2.86 (m, 1H), 2.14 – 2.06 (m, 1H), 1.37 (s, 9H), 0.98 – 0.84 (m, 2H), 0.79 – 0.68 (m, 2H); LC-MS (ESI) [M+Na]^+^ m/z 551.5.

12. *Tert*-butyl ((2*S*,3*R*)-4-((*N*-cyclopropyl-4-nitrophenyl)sulfonamido)-3-hydroxy-1-phenylbutan-2-yl)carbamate (**17c**)

Compound **17c** was prepared from *tert*-butyl ((2*S*,3*R*)-4-(cyclopropylamino)-3-hydroxy-1-phenylbutan-2-yl)carbamate (**16**, 1.60 g, 5.00 mmol) and 4-nitrobenzenesulfonyl chloride (1.22 g, 5.50 mmol) by following the same procedure outlined for **17a** to give a white powder: yield 2.33 g (92.1%); ^1^H NMR (500 MHz, CDCl_3_) δ 8.37 (d, *J* = 8.0 Hz, 2H), 8.03 (d, *J* = 8.0 Hz, 2H), 7.42 – 7.27 (m, 3H), 7.25 – 7.16 (m, 2H), 4.68 (s, 1H), 3.96 (s, 1H), 3.81 (s, 1H), 3.74 – 3.57 (m, 1H), 3.38 – 3.25 (m, 2H), 3.05 – 2.96 (m, 1H), 2.96 – 2.72 (m, 1H), 2.16 (s, 1H), 1.39 (s, 9H), 1.00 – 0.87 (m, 2H), 0.83 – 0.69 (m, 2H); LC-MS (ESI) [M+Na]^+^ m/z 528.5.

13. *N*-((2*R*,3*S*)-3-amino-2-hydroxy-4-phenylbutyl)-*N*-cyclopropyl-4-methoxybenzenesulfonamide (**18a**)

To a stirred solution of **17a** (1.00 g, 2.00 mmol) in CH_2_Cl_2_ (9 mL) was added CF_3_COOH (3 mL) dropwise. The reaction mixture was stirred at 25 ℃ for 5 h. The solvent was neutralized with saturated aqueous Na_2_CO_3_ solution to pH 7.0, and then extracted with CH_2_Cl_2_ (3×10 mL). The organic phase was washed with saturated aqueous NaCl solution and dried over anhydrous Na_2_SO_4_, and then concentrated under reduced pressure. The crude product was purified by silica gel column chromatography (CH_2_Cl_2_/methanol, 20:1) to furnish **18a** as a white solid: yield 0.63 g (80.4%); ^1^H NMR (400 MHz, CDCl_3_) δ 7.84 – 7.77 (m, 2H), 7.34 – 7.27 (m, 2H), 7.25 – 7.16 (m, 3H), 7.03 – 6.96 (m, 2H), 3.99 – 3.90 (m, 1H), 3.87 (s, 3H), 3.36 – 3.30 (m, 2H), 3.21 (m, 1H), 2.99 (dd, *J* = 12.0 Hz, 4.0 Hz, 1H), 2.69 (s, 2H), 2.60 – 2.52 (m, 1H), 2.11 – 2.03 (m, 1H), 0.99 – 0.82 (m, 2H), 0.74 – 0.63 (m, 2H); LC-MS (ESI) [M+H]^+^ m/z 391.5.

14. *N*-((2*R*,3*S*)-3-amino-2-hydroxy-4-phenylbutyl)-*N*-cyclopropyl-4-(trifluoromethyl)benzenesulfonamide (**18b**)

Compound **18b** was prepared from **17b** (1.06 g, 2.00 mmol) by following the same procedure outlined for **18a** to give a white powder: yield 0.68 g (78.9%); ^1^H NMR (400 MHz, CDCl_3_) δ 8.01 (d, *J* = 8.0 Hz, 2H), 7.81 (d, *J* = 8.0 Hz, 2H), 7.31 (t, *J* = 7.2 Hz, 2H), 7.25 – 7.19 (m, 3H), 3.96 – 3.90 (m, 1H), 3.41 – 3.36 (m, 2H), 3.18 (dt, *J* = 10.0, 4.2 Hz, 1H), 2.96 (dd, *J* = 13.6, 4.0 Hz, 1H), 2.58 – 2.50 (m, 1H), 2.30 (s, 2H), 2.18 – 2.11 (m, 1H), 1.00 – 0.87 (m, 2H), 0.79 – 0.68 (m, 2H); LC-MS (ESI) [M+H]^+^ m/z 429.4.

15. *N*-((2*R*,3S)-3-amino-2-hydroxy-4-phenylbutyl)-N-cyclopropyl-4-nitrobenzenesulfonamide (**18c**)

Compound **18c** was prepared from **17c** (2.00 g, 4.00 mmol) by following the same procedure outlined for **18a** to give a white powder: yield 1.21 g (74.2%); ^1^H NMR (400 MHz, CDCl_3_) δ 8.41 – 8.33 (m, 2H), 8.11 – 8.02 (m, 2H), 7.32 (t, *J* = 7.2Hz, 2H), 7.24 – 7.15 (m, 3H), 3.88 (q, *J* = 6.0 Hz, 1H), 3.42 (d, *J* = 8 Hz, 2H), 3.18 – 3.11 (m, 1H), 2.97 – 2.86 (m, 1H), 2.56 – 2.46 (m, 1H), 2.23 – 2.15 (m, 1H), 0.93 (m, 2H), 0.86 – 0.66 (m, 2H); LC-MS (ESI) [M+H]^+^ m/z 406.5.

16. 4-Amino-*N*-((2*R*,3*S*)-3-amino-2-hydroxy-4-phenylbutyl)-*N*-cyclopropylbenzenesulfonamide (**18d**)

To a solution of compound **18c** (0.81 g, 2.00 mmol) in ethyl acetate/methanol (2.5 mL/5 mL) was added 10% Pd/C (0.81 g). The mixture was stirred at 25 ℃ under the hydrogen atmosphere of 50 psi pressure for 4 h. The reaction solution was filtered with Celite 545^®^ and washed with methanol. The filtrate was concentrated under reduced pressure to give **18d** as a yellow solid: yield 0.73 g (96.9%); ^1^H NMR (500 MHz, CDCl_3_) δ 7.67 (d, *J* = 10.0 Hz, 2H), 7.38 – 7.27 (m, 3H), 7.26 – 7.21 (m, 2H), 6.73 (d, *J* =10.0 Hz, 2H), 4.23 (s, 2H), 3.96 – 3.87 (m, 1H), 3.41 – 3.31 (m, 2H), 3.24 – 3.16 (m, 1H), 3.05 – 2.97 (m, 1H), 2.61 – 2.50 (m, 1H), 2.23 – 1.95 (m, 3H), 1.02 – 0.95 (m, 1H), 0.94 – 0.84 (m, 1H), 0.77 – 0.65 (m, 2H); LC-MS (ESI) [M+H]^+^ m/z 376.5.

17. *Tert*-butyl (*R*)-3-(((2*S*,3*R*)-4-((*N*-cyclopropyl-4-methoxyphenyl)sulfonamido)-3-hydroxy-1-phenylbutan-2-yl)carbamoyl)piperidine-1-carboxylate (**19a**)

To a stirred solution of **9** (34.4 mg, 0.15 mmol) and **18a** (61.4 mg, 0.16 mmol) in anhydrous DMF (1 mL) was added EDCI (43.1 mg, 0.23 mmol), HOBt (22.3 mg, 0.16 mmol) and DMAP (3.70 mg, 0.03 mmol) at 0 ℃ under argon atmosphere. The reaction mixture was warmed to 25 ℃ and stirred for 8 hours. After this period, the reaction was diluted with 5 mL ethyl acetate, washed with H_2_O and saturated aqueous NaCl solution and dried over anhydrous Na_2_SO_4_, and then concentrated under reduced pressure. The crude product was purified by silica gel column chromatography (hexanes/ ethyl acetate, 3:2) to furnish **19a** as a colorless oil: yield 86.1 mg (95.4%); ^1^H NMR (500 MHz, DMSO-*d*_6_) δ 7.90 – 7.79 (m, 1H), 7.73 (d, *J* = 8.5 Hz, 2H), 7.25 – 7.18 (m, 4H), 7.14 (d, *J* = 8.5 Hz, 3H), 5.13 (d, *J* = 5.0 Hz, 1H), 3.90 – 3.82 (m, 4H), 3.82 – 3.76 (m, 1H), 3.74 – 3.56 (m, 2H), 3.11 – 3.02 (m, 1H), 2.84 – 2.74 (m, 1H), 2.47 – 2.40 (m, 1H), 2.17 – 2.06 (m, 1H), 2.03 – 1.90 (m, 2H), 1.72 – 1.60 (m, 2H), 1.60 – 1.54 (m, 1H), 1.44 – 1.34 (m, 11H), 0.94 – 0.88 (m, 3H), 0.74 – 0.67 (m, 1H), 0.60 – 0.53 (m, 1H); LC-MS (APCI) [M+H]^+^ m/z 602.6.

18. *Tert*-butyl (*R*)-3-(((2*S*,3*R*)-4-((*N*-cyclopropyl-4-(trifluoromethyl)phenyl)sulfonamido)-3-hydroxy-1-phenylbutan-2-yl)carbamoyl)piperidine-1-carboxylate (**19b**)

Compound **19b** was prepared from **9** (34.4 mg, 0.15 mmol) and **18b** (67.4 mg, 0.16 mmol) by following the same procedure outlined for **19a** to give a colorless oil: yield 77.7 mg (81.1%); ^1^H NMR (500 MHz, DMSO-*d*_6_) δ 8.10 – 7.97 (m, 4H), 7.85 (d, *J* = 9.0 Hz, 1H), 7.26 – 7.19 (m, 4H), 7.18 – 7.11 (m, 1H), 5.17 (s, 1H), 3.90 – 3.77 (m, 2H), 3.76 – 3.53 (m, 2H), 3.28 (s, 1H), 3.10 – 3.03 (m, 1H), 3.02 – 2.95 (m, 1H), 2.57 – 2.52 (m, 1H), 2.48 – 2.40 (m, 1H), 2.14 – 2.07 (m, 2H), 1.64 (d, *J* = 13.0 Hz, 1H), 1.55 (d, *J* = 13.0 Hz, 1H), 1.40 (s, 9H), 1.38 – 1.34 (m, 1H), 1.31 – 1.18 (m, 2H), 0.95 – 0.88 (m, 2H), 0.79 – 0.73 (m, 1H), 0.65 – 0.58 (m, 1H); LC-MS (APCI) [M+H]^+^ m/z 640.6.

19. *Tert*-butyl (*R*)-3-(((2*S*,3*R*)-4-((*N*-cyclopropyl-4-nitrophenyl)sulfonamido)-3-hydroxy-1-phenylbutan-2-yl)carbamoyl)piperidine-1-carboxylate (**19c**)

Compound **19c** was prepared from **9** (34.4 mg, 0.15 mmol) and **18c** (63.8 mg, 0.16 mmol) by following the same procedure outlined for **19a** to give a yellow oil: yield 75.0 mg (81.2%); ^1^H NMR (500 MHz, DMSO-*d*_6_) δ 8.44 (d, *J* = 8.2 Hz, 2H), 8.08 (d, *J* = 8.1 Hz, 2H), 7.89 – 7.83 (m, 1H), 7.27 – 7.19 (m, 4H), 7.19 – 7.13 (m, 1H), 5.17 (s, 1H), 3.91 – 3.77 (m, 2H), 3.77 – 3.56 (m, 2H), 3.09 – 2.99 (m, 2H), 2.65 – 2.55 (m, 1H), 2.49 – 2.42 (m, 1H), 2.20 – 2.07 (m, 2H), 1.71 – 1.64 (m, 1H), 1.61 – 1.54 (m, 1H), 1.41 (s, 9H), 1.36 – 1.20 (m, 3H), 0.98 – 0.83 (m, 3H), 0.82 – 0.75 (m, 1H), 0.69 – 0.62 (m, 1H); LC-MS (APCI) [M+H]^+^ m/z 617.7.

20. *Tert*-butyl (*R*)-3-(((2*S*,3*R*)-4-((4-amino-*N*-cyclopropylphenyl)sulfonamido)-3-hydroxy-1-phenylbutan-2-yl)carbamoyl)piperidine-1-carboxylate (**19d**)

Compound **19d** was prepared from **9** (34.4 mg, 0.15 mmol) and **18d** (59.1 mg, 0.16 mmol) by following the same procedure outlined for **19a** to give a yellow oil: yield 67.0 mg (76.2%); ^1^H NMR (500 MHz, CD_3_OD) δ 7.54 (d, *J* = 8.5 Hz, 2H), 7.30 – 7.24 (m, 4H), 7.21 – 7.15 (m, 1H), 6.72 (d, *J* = 8.5 Hz, 2H), 4.26 – 4.19 (m, 1H), 4.09 – 4.03 (m, 1H), 4.02 – 3.96 (m, 1H), 3.95 – 3.90 (m, 1H), 3.85 – 3.74 (m, 1H), 3.28 – 3.22 (m, 1H), 2.99 – 2.93 (m, 1H), 2.82 – 2.78 (m, 1H), 2.69 – 2.56 (m, 2H), 2.23 – 2.19 (m, 1H), 2.05 – 2.02 (m, 1H), 1.84 – 1.79 (m, 1H), 1.66 – 1.62 (m, 1H), 1.48 (s, 9H), 0.99 – 0.93 (m, 4H), 0.77 – 0.70 (m, 1H), 0.67 – 0.61 (m, 1H); LC-MS (APCI) [M+H]^+^ m/z 587.7.

21. *Tert*-butyl (*S*)-3-(((2*S*,3*R*)-4-((*N*-cyclopropyl-4-methoxyphenyl)sulfonamido)-3-hydroxy-1-phenylbutan-2-yl)carbamoyl)piperidine-1-carboxylate (**20a**)

Compound **20a** was prepared from **10** (34.4 mg, 0.15 mmol) and **18a** (61.4 mg, 0.16 mmol) by following the same procedure outlined for **19a** to give a colorless powder: yield 73.4 mg (81.4%); ^1^H NMR (500 MHz, DMSO-*d*_6_) δ 7.94 – 7.81 (m, 1H), 7.74 (d, *J* = 8.0 Hz, 2H), 7.27 – 7.06 (m, 7H), 5.18 (s, 1H), 3.85 (s, 3H), 3.81 – 3.76 (m, 1H), 3.75 – 3.67 (m, 1H), 3.32 – 3.26 (m, 1H), 3.10 (d, *J* = 13.5 Hz, 1H), 2.90 (s, 1H), 2.81 – 2.72 (m, 2H), 2.65 – 2.57 (m, 1H), 2.14 (s, 1H), 1.95 – 1.89 (m, 1H), 1.52 (d, *J* = 7.5 Hz, 1H), 1.46 – 1.31 (m, 10H), 1.27 – 1.13 (m, 4H), 0.94 (s, 2H), 0.73 (s, 1H), 0.57 (s, 1H); LC-MS (APCI) [M+H]^+^ m/z 602.6.

22. *Tert*-butyl (*S*)-3-(((2*S*,3*R*)-4-((*N*-cyclopropyl-4-(trifluoromethyl)phenyl)sulfonamido)-3-hydroxy-1-phenylbutan-2-yl)carbamoyl)piperidine-1-carboxylate (**20b**)

Compound **20b** was prepared from **10** (34.4 mg, 0.15 mmol) and **18b** (67.4 mg, 0.16 mmol) by following the same procedure outlined for **19a** to give a yellow powder: yield 92.3 mg (96.3%); ^1^H NMR (500 MHz, DMSO-*d*_6_) δ 8.16 – 7.96 (m, 4H), 7.89 (s, 1H), 7.29 – 7.17 (m, 4H), 7.17 – 7.10 (m, 1H), 5.21 (s, 1H), 3.92 – 3.64 (m, 4H), 3.07 (d, *J* = 14.0 Hz, 1H), 2.93 (s, 1H), 2.69 – 2.52 (m, 2H), 2.18 – 2.00 (m, 2H), 1.55 – 1.48 (m, 1H), 1.48 – 1.42 (m, 1H), 1.37 (s, 9H), 1.30 – 1.09 (m, 4H), 0.99 – 0.83 (m, 2H), 0.81 – 0.72 (m, 1H), 0.66 – 0.57 (m, 1H); LC-MS (ESI) [M+Na]^+^ m/z 662.7.

23. *Tert*-butyl (*S*)-3-(((2*S*,3*R*)-4-((*N*-cyclopropyl-4-nitrophenyl)sulfonamido)-3-hydroxy-1-phenylbutan-2-yl)carbamoyl)piperidine-1-carboxylate (**20c**)

Compound **20c** was prepared from **10** (34.4 mg, 0.15 mmol) and **18c** (63.8 mg, 0.16 mmol) by following the same procedure outlined for **19a** to give a yellow powder: yield 89.7 mg (96.1%); ^1^H NMR (500 MHz, DMSO-*d*_6_) δ 8.46 (d, *J* = 8.5 Hz, 2H), 8.07 (d, *J* = 8.5 Hz, 2H), 7.97 – 7.83 (m, 1H), 7.26 – 7.18 (m, 4H), 7.17 – 7.11 (m, 1H), 5.21 (s, 1H), 3.91 – 3.76 (m, 3H), 3.75 – 3.67 (m, 1H), 3.11 – 2.94 (m, 2H), 2.92 – 2.87 (m, 1H), 2.76 – 2.71 (m, 1H), 2.68 – 2.57 (m, 1H), 2.18 – 2.08 (m, 2H), 1.56 – 1.48 (m, 1H), 1.47 – 1.42 (m, 1H), 1.38 (s, 9H), 1.26 – 1.17 (m, 3H), 1.01 – 0.89 (m, 2H), 0.83 – 0.76 (m, 1H), 0.68 – 0.61 (m, 1H); LC-MS (APCI) [M+H]^+^ m/z 617.5.

24. *Tert*-butyl (*S*)-3-(((2*S*,3*R*)-4-((4-amino-*N*-cyclopropylphenyl)sulfonamido)-3-hydroxy-1-phenylbutan-2-yl)carbamoyl)piperidine-1-carboxylate (**20d**)

Compound **20d** was prepared from **10** (34.4 mg, 0.15 mmol) and **18d** (59.1 mg, 0.16 mmol) by following the same procedure outlined for **19a** to give a yellow powder: yield 61.7 mg (70.2%);^1^H NMR (500 MHz, DMSO-*d*_6_) δ 7.86 (s, 1H), 7.44 (d, *J* = 8.0 Hz, 2H), 7.25 – 7.18 (m, 4H), 7.15 (s, 1H), 6.66 (d, *J* = 8.0 Hz, 2H), 6.03 (s, 2H), 5.11 (s, 1H), 3.95 – 3.69 (m, 4H), 3.08 (d, *J* = 13.5 Hz, 1H), 2.73 – 2.60 (m, 2H), 2.19 – 2.09 (m, 1H), 2.07 – 1.94 (m, 1H), 1.92 – 1.82 (m, 1H), 1.55 – 1.49 (m, 1H), 1.39 (s, 9H), 1.27 – 1.24 (m, 3H), 1.22 – 1.16 (m, 2H), 0.93 – 0.86 (m, 2H), 0.72 – 0.66 (m, 1H), 0.57 – 0.50 (m, 1H); LC-MS (ESI) [M+Na]^+^ m/z 609.7.

25. *Tert*-butyl 4-(((2*S*,3*R*)-4-((*N*-cyclopropyl-4-methoxyphenyl)sulfonamido)-3-hydroxy-1-phenylbutan-2-yl)carbamoyl)piperidine-1-carboxylate (**21a**)

Compound **21a** was prepared from **11** (34.4 mg, 0.15 mmol) and **18a** (61.4 mg, 0.16 mmol) by following the same procedure outlined for **19a** to give a white powder: yield 80.7 mg (89.5%); ^1^H NMR (500 MHz, CD_3_OD) δ 7.81 (d, *J* = 8.5 Hz, 2H), 7.29 – 7.22 (m, 4H), 7.21 – 7.16 (m, 1H), 7.13 (d, *J* = 8.5 Hz, 2H), 4.12 – 4.00 (m, 2H), 3.97 – 3.93 (m, 1H), 3.91 (s, 3H), 3.46 – 3.40 (m, 1H), 3.29 – 3.23 (m, 1H), 3.06 – 2.97 (m, 1H), 2.84 – 2.59 (m, 3H), 2.30 – 2.19 (m, 1H), 2.08 – 2.02 (m, 1H), 1.65 – 1.57 (m, 1H), 1.46 (s, 9H), 1.42 – 1.20 (m, 4H), 1.03 – 0.92 (m, 2H), 0.80 – 0.72 (m, 1H), 0.70 – 0.62 (m, 1H); LC-MS (ESI) [M+Na]^+^ m/z 624.6.

26. *Tert*-butyl 4-(((2*S*,3*R*)-4-((*N*-cyclopropyl-4-(trifluoromethyl)phenyl)sulfonamido)-3-hydroxy-1-phenylbutan-2-yl)carbamoyl)piperidine-1-carboxylate (**21b**)

Compound **21b** was prepared from **11** (34.4 mg, 0.15 mmol) and **18b** (67.4 mg, 0.16 mmol) by following the same procedure outlined for **19a** to give a white powder: yield 84.3 mg (88.0%); ^1^H NMR (500 MHz, CD_3_OD) δ 8.08 (d, *J* = 8.0 Hz, 2H), 7.95 (d, *J* = 8.0 Hz, 2H), 7.28 – 7.24 (m, 4H), 7.21 – 7.16 (m, 1H), 4.11 – 4.00 (m, 2H), 3.97 – 3.91 (m, 2H), 3.47 (d, *J* = 14.0 Hz, 1H), 3.25 (d, *J* = 14.0 Hz, 1H), 3.20 – 3.13 (m, 1H), 2.80 – 2.61 (m, 3H), 2.29 – 2.22 (m, 1H), 2.19 – 2.12 (m, 1H), 1.63 – 1.56 (m, 1H), 1.46 (s, 9H), 1.38 – 1.31 (m, 2H), 1.30 – 1.21 (m, 1H), 1.06 – 0.97 (m, 2H), 0.85 – 0.78 (m, 1H), 0.75 – 0.68 (m, 1H); LC-MS (ESI) [M+Na]^+^ m/z 662.6.

27. *Tert*-butyl 4-(((2*S*,3*R*)-4-((*N*-cyclopropyl-4-nitrophenyl)sulfonamido)-3-hydroxy-1-phenylbutan-2-yl)carbamoyl)piperidine-1-carboxylate (**21c**)

Compound **21c** was prepared from **11** (45.8 mg, 0.20 mmol) and **18c** (85.1 mg, 0.21 mmol) by following the same procedure outlined for **19a** to give a white powder: yield 113.4 mg (92.0%);^1^H NMR (500 MHz, CD_3_OD) δ 8.48 – 8.43 (m, 2H), 8.14 – 8.10 (m, 2H), 7.29 – 7.23 (m, 4H), 7.22 – 7.17 (m, 1H), 4.11 – 4.00 (m, 2H), 3.98 – 3.90 (m, 2H), 3.51 – 3.45 (m, 1H), 3.26 – 3.17 (m, 2H), 2.82 – 2.61 (m, 3H), 2.30 – 2.19 (m, 2H), 1.66 – 1.56 (m, 1H), 1.46 (s, 9H), 1.39 – 1.22 (m, 3H), 1.09 – 0.96 (m, 2H), 0.87 – 0.80 (m, 1H), 0.78 – 0.70 (m, 1H); LC-MS (ESI) [M+Na]^+^ m/z 639.6.

28. *Tert*-butyl 4-(((2*S*,3*R*)-4-((4-amino-*N*-cyclopropylphenyl)sulfonamido)-3-hydroxy-1-phenylbutan-2-yl)carbamoyl)piperidine-1-carboxylate (**21d**)

Compound **21d** was prepared from **11** (34.4 mg, 0.15 mmol) and **18d** (59.1 mg, 0.16 mmol) by following the same procedure outlined for **19a** to give a white powder: yield 62.3 mg (70.9%); ^1^H NMR (500 MHz, CD_3_OD) δ 7.53 (d, *J* = 8.5 Hz, 2H), 7.28 – 7.22 (m, 4H), 7.21 – 7.15 (m, 1H), 6.73 (d, *J* = 8.5 Hz, 2H), 4.10 – 4.02 (m, 2H), 3.96 – 3.90 (m, 2H), 3.42 – 3.36 (m, 1H), 3.29 – 3.24 (m, 1H), 2.99 – 2.92 (m, 1H), 2.82 – 2.59 (m, 3H), 2.28 – 2.20 (m, 1H), 2.07 – 1.99 (m, 1H), 1.65 – 1.57 (m, 1H), 1.46 (s, 9H), 1.38 – 1.30 (m, 4H), 1.28 – 1.19 (m, 1H), 1.01 – 0.92 (m, 2H), 0.77 – 0.70 (m, 1H), 0.67 – 0.59 (m, 1H); LC-MS (ESI) [M+Na]^+^ m/z 609.7.

29. (*R*)-*N*-((2*S*,3*R*)-4-((*N*-cyclopropyl-4-methoxyphenyl)sulfonamido)-3-hydroxy-1-phenylbutan-2-yl)piperidine-3-carboxamide (**22a**)

To a stirred solution of **19a** (85.0 mg, 0.14 mmol) in CH_2_Cl_2_ (1 mL) was bubbled hydrochloric acid gas at 25 ℃ for 0.5 hour. The reaction mixture was concentrated under reduced pressure, and the residue was neutralized with saturated aqueous NaHCO_3_ solution to pH 7.0, extracted with CH_2_Cl_2_ (3×5 mL), and then dried over anhydrous Na_2_SO_4_. The solvent was evaporated under reduced pressure to give **22a** as a white powder: yield 60.0 mg (85.5%); mp 162.8-164.3 ℃; ^1^H NMR (500 MHz, CDCl_3_) δ 8.07 (s, 1H), 7.77 (d, *J* = 9.0 Hz, 2H), 7.29 – 7.25 (m, 4H), 7.22 – 7.16 (m, 1H), 6.99 (d, *J* = 9.0 Hz, 2H), 4.22 – 4.16 (m, 1H), 4.04 – 4.00 (m, 1H), 3.87 (s, 3H), 3.42 (dd, *J* = 14.5, 5.0 Hz, 1H), 3.12 – 3.04 (m, 2H), 2.95 – 2.84 (m, 2H), 2.75 – 2.61 (m, 3H), 2.32 – 2.28 (m, 1H), 2.08 – 2.03 (m, 1H), 1.73 – 1.66 (m, 1H), 1.62 – 1.55 (m, 1H), 1.25 (s, 2H), 1.20 – 1.11 (m, 1H), 0.98 – 0.92 (m, 1H), 0.87 – 0.82 (m, 1H), 0.73 – 0.63 (m, 2H); ^13^C NMR (126 MHz, CDCl_3_) δ 176.46, 163.18, 138.42, 129.95, 129.43, 129.16, 128.48, 126.54, 114.29, 72.67, 55.74, 54.97, 54.60, 48.20, 46.39, 41.82, 35.18, 32.40, 27.26, 22.79, 7.58, 7.38; HRMS (ESI) m/z calcd. for C_26_H_35_N_3_O_5_S ([M-H]^-^): 500.2219, found 500.2207.

30. (*R*)-*N*-((2*S*,3*R*)-4-((*N*-cyclopropyl-4-(trifluoromethyl)phenyl)sulfonamido)-3-hydroxy-1-phenylbutan-2-yl)piperidine-3-carboxamide (**22b**)

Compound **22b** was prepared from **19b** (60.0 mg, 0.09 mmol) by following the same procedure outlined for **22a** to give a white powder: yield 42.5 mg (87.6%); mp 204.8-206.3 ℃; ^1^H NMR (500 MHz, CDCl_3_) δ 8.28 (s, 1H), 7.97 (d, *J* = 8.0 Hz, 2H), 7.79 (d, *J* = 8.0 Hz, 2H), 7.30 – 7.23 (m, 4H), 7.22 – 7.17 (m, 1H), 4.22 – 4.15 (m, 1H), 4.07 – 4.01 (m, 1H), 3.46 (dd, *J* = 14.0, 4.0 Hz, 1H), 3.17 (dd, *J* = 14.0, 8.0 Hz, 1H), 3.07 (dd, *J* = 14.0, 4.0 Hz, 1H), 2.95 – 2.85 (m, 2H), 2.79 – 2.68 (m, 2H), 2.66 – 2.58 (m, 1H), 2.35 (s, 1H), 2.16 – 2.08 (m, 1H), 1.75 – 1.67 (m, 1H), 1.62 – 1.54 (m, 1H), 1.34 – 1.27 (m, 1H), 1.13 (s, 1H), 1.02 – 0.95 (m, 1H), 0.88 – 0.82 (m, 1H), 0.78 – 0.66 (m, 2H); ^13^C NMR (126 MHz, CDCl_3_) δ 176.73, 141.62, 138.17, 134.63 (d, *J* = 32.9 Hz), 129.39, 128.62, 128.40, 126.76, 126.28 (q, *J* = 3.5 Hz), 123.33 (d, *J* = 272.9 Hz), 72.49, 55.56, 54.16, 47.97, 46.30, 41.34, 35.36, 31.82, 27.12, 22.36, 7.88, 7.44; HRMS (ESI) m/z calcd. for C_26_H_32_F_3_N_3_O_4_S ([M-H]^-^): 538.1987, found 538.1965.

31. (*R*)-*N*-((2*S*,3*R*)-4-((*N*-cyclopropyl-4-nitrophenyl)sulfonamido)-3-hydroxy-1-phenylbutan-2-yl)piperidine-3-carboxamide (**22c**)

Compound **22c** was prepared from **19c** (75.0 mg, 0.12 mmol) by following the same procedure outlined for **22a** to give a white powder: yield 53.8 mg (86.9%); mp 196.8-198.4 ℃; ^1^H NMR (500 MHz, CD_3_OD) δ 8.44 (d, *J* = 7.0 Hz, 2H), 8.11 (d, *J* = 7.0 Hz, 2H), 7.19 (d, *J* = 33.8 Hz, 5H), 4.04 (s, 1H), 3.90 (s, 1H), 3.45 (d, *J* = 13.0 Hz, 1H), 3.23 – 3.13 (m, 2H), 2.95 – 2.81 (m, 1H), 2.60 (s, 2H), 2.51 (t, *J* = 10.0 Hz, 1H), 2.40 (t, *J* = 10.5 Hz, 1H), 2.26 – 2.14 (m, 2H), 1.79 – 1.69 (m, 1H), 1.66 – 1.59 (m, 1H), 1.56 – 1.39 (m, 2H), 1.35 – 1.28 (m, 2H), 1.06 – 0.88 (m, 2H), 0.85 – 0.67 (m, 2H); ^13^C NMR (126 MHz, CD_3_OD) δ 176.27, 151.67, 145.12, 140.09, 130.38, 130.36, 129.21, 127.25, 125.31, 73.07, 55.98, 54.76, 49.46, 46.45, 44.42, 36.47, 32.56, 28.48, 25.48, 9.07, 7.79; HRMS (ESI) m/z calcd. for C_25_H_32_N_4_O_6_S ([M-H]^-^): 515.1965, found 515.1942.

32. (*R*)-*N*-((2*S*,3*R*)-4-((4-amino-*N*-cyclopropylphenyl)sulfonamido)-3-hydroxy-1-phenylbutan-2-yl)piperidine-3-carboxamide (**22d**)

Compound **22d** was prepared from **19d** (30.0 mg, 0.05 mmol) by following the same procedure outlined for **22a** to give a white powder: yield 20.7 mg (85.2%); mp 216.5-218.3 ℃; ^1^H NMR (500 MHz, CD_3_OD) δ 7.54 (d, *J* = 8.5 Hz, 2H), 7.27 – 7.23 (m, 4H), 7.20 – 7.15 (m, 1H), 6.73 (d, *J* = 8.5 Hz, 2H), 4.10 – 4.05 (m, 1H), 3.97 – 3.91 (m, 1H), 3.40 (dd, *J* = 14.5, 4.0 Hz, 1H), 3.25 (dd, *J* = 14.0, 3.5 Hz, 1H), 2.99 – 2.93 (m, 2H), 2.72 – 2.59 (m, 3H), 2.55 – 2.49 (m, 1H), 2.36 – 2.29 (m, 1H), 2.06 – 2.00 (m, 1H), 1.84 – 1.78 (m, 1H), 1.74 – 1.67 (m, 1H), 1.62 – 1.49 (m, 2H), 1.05 – 0.87 (m, 3H), 0.76 – 0.71 (m, 1H), 0.66 – 0.61 (m, 1H); ^13^C NMR (101 MHz, CD_3_OD) δ 175.73, 154.59, 140.23, 130.90, 130.42, 129.17, 127.18, 124.25, 114.27, 73.52, 56.42, 54.76, 46.16, 43.45, 36.55, 33.50, 28.13, 28.10, 24.74, 8.82, 7.57; HRMS (ESI) m/z calcd. for C_25_H_34_N_4_O_4_S ([M-H]^-^): 485.2223, found 485.2219.

33. (*S*)-*N*-((2*S*,3*R*)-4-((*N*-cyclopropyl-4-methoxyphenyl)sulfonamido)-3-hydroxy-1-phenylbutan-2-yl)piperidine-3-carboxamide (**23a**)

Compound **23a** was prepared from **20a** (73.4 mg, 0.12 mmol) by following the same procedure outlined for **22a** to give a white powder: yield 56.2 mg (93.5%); mp 92.9-94.7 ℃; ^1^H NMR (500 MHz, CDCl_3_) δ 8.93 (s, 1H), 8.66 (s, 1H), 7.87 – 7.65 (m, 3H), 7.24 – 7.11 (m, 5H), 7.00 (s, 2H), 4.31 (s, 1H), 4.15 (s, 1H), 3.86 (s, 3H), 3.42 – 3.09 (m, 5H), 2.98 – 2.77 (m, 4H), 1.99 (s, 1H), 1.74 – 1.48 (m, 3H), 1.37 – 1.29 (m, 1H), 0.93 – 0.86 (m, 2H), 0.69 – 0.56 (m, 2H); ^13^C NMR (126 MHz, CDCl_3_) δ 172.92, 163.22, 138.54, 130.11, 129.49, 129.16, 128.34, 126.34, 114.43, 71.45, 55.85, 54.20, 53.65, 45.81, 44.15, 38.70, 34.67, 32.01, 26.21, 20.91, 7.95, 7.08; HRMS (ESI) m/z calcd. for C_26_H_35_N_3_O_5_S ([M+H]^+^): 502.2375, found 502.2361.

34. (*S*)-*N*-((2*S*,3*R*)-4-((*N*-cyclopropyl-4-(trifluoromethyl)phenyl)sulfonamido)-3-hydroxy-1-phenylbutan-2-yl)piperidine-3-carboxamide (**23b**)

Compound **23b** was prepared from **20b** (70.0 mg, 0.11 mmol) by following the same procedure outlined for **22a** to give a yellow powder: yield 56.6 mg (95.5%); mp 236.5-238.4 ℃; ^1^H NMR (500 MHz, CD_3_OD) δ 8.09 (d, *J* = 8.2 Hz, 2H), 7.95 (d, *J* = 8.3 Hz, 2H), 7.28 – 7.22 (m, 4H), 7.19 – 7.14 (m, 1H), 4.21 – 4.15 (m, 1H), 3.97 – 3.92 (m, 1H), 3.57 (dd, *J* = 14.5, 4.5 Hz, 1H), 3.24 (dd, *J* = 14.0, 3.5 Hz, 1H), 3.19 – 3.08 (m, 3H), 3.04 – 2.96 (m, 2H), 2.71 – 2.66 (m, 1H), 2.64 – 2.58 (m, 1H), 2.16 – 2.11 (m, 1H), 1.67 – 1.61 (m, 1H), 1.53 – 1.46 (m, 1H), 1.39 – 1.32 (m, 2H), 1.06 – 1.00 (m, 1H), 0.99 – 0.94 (m, 1H), 0.83 – 0.77 (m, 1H), 0.72 – 0.66 (m, 1H); ^13^C NMR (126 MHz, CD_3_OD) δ 173.28 , 142.14 , 138.93 , 134.07 (d, *J* = 32.8 Hz), 129.26 , 128.57 , 128.06 , 126.17 (q, *J* = 3.8 Hz), 126.08 , 123.72 (d, *J* = 272.1 Hz), 71.95 , 54.63 , 53.44 , 44.92 , 43.73 , 37.74 , 35.01 , 31.41 , 25.93 , 20.08 , 7.60 , 6.55; HRMS (ESI) m/z calcd. for C_26_H_32_F_3_N_3_O_4_S ([M+H]^+^): 540.2144, found 540.2140.

35. (*S*)-*N*-((2*S*,3*R*)-4-((*N*-cyclopropyl-4-nitrophenyl)sulfonamido)-3-hydroxy-1-phenylbutan-2-yl)piperidine-3-carboxamide (**23c**)

Compound **23c** was prepared from **20c** (89.7 mg, 0.15 mmol) by following the same procedure outlined for **22a** to give a yellow powder: yield 73.7 mg (95.2%); mp 155.9-157.2 ℃; ^1^H NMR (500 MHz, CD_3_OD) δ 8.42 (d, *J* = 9.0 Hz, 2H), 8.09 (d, *J* = 9.0 Hz, 2H), 7.24 – 7.19 (m, 4H), 7.16 – 7.11 (m, 1H), 4.16 – 4.11 (m, 1H), 3.92 – 3.87 (m, 1H), 3.53 (dd, *J* = 14.5, 4.0 Hz, 1H), 3.21 – 3.11 (m, 3H), 3.06 (dd, *J* = 12.7, 4.0 Hz, 1H), 3.01 – 2.92 (m, 2H), 2.67 – 2.62 (m, 1H), 2.57 (dd, *J* = 14.0, 11.5 Hz, 1H), 2.17 – 2.12 (m, 1H), 1.64 – 1.57 (m, 1H), 1.50 – 1.41 (m, 1H), 1.35 – 1.28 (m, 2H), 1.03 – 1.97 (m, 1H), 0.96 – 0.90 (m, 1H), 0.82 – 0.76 (m, 1H), 0.71 – 0.64 (m, 1H); ^13^C NMR (126 MHz, CD_3_OD) δ 174.50, 151.68, 145.17, 140.09, 130.44, 130.37, 129.27, 127.30, 125.36, 73.03, 55.76, 54.65, 46.12, 44.94, 38.92, 36.26, 32.44, 27.13, 21.27, 8.91, 7.78; HRMS (ESI) m/z calcd. for C_25_H_32_N_4_O_6_S ([M+H]^+^): 517.212, found 517.2120.

36. (*S*)-*N*-((2*S*,3*R*)-4-((4-amino-*N*-cyclopropylphenyl)sulfonamido)-3-hydroxy-1-phenylbutan-2-yl)piperidine-3-carboxamide (**23d**)

Compound **23d** was prepared from **20d** (30.0 mg, 0.05 mmol) by following the same procedure outlined for **22a** to give a yellow powder: yield 21.0 mg (86.4%); mp 77.8-79.4 ℃; ^1^H NMR (500 MHz, CD_3_OD) δ 7.98 (d, *J* = 8.0 Hz, 2H), 7.56 (d, *J* = 8.0 Hz, 2H), 7.28 – 7.20 (m, 4H), 7.18 – 7.12 (m, 1H), 4.19 – 4.14 (m, 1H), 3.97 – 3.92 (m, 1H), 3.56 – 3.55 (m, 1H), 3.25 – 3.19 (m, 1H), 3.16 – 3.07 (m, 3H), 3.04 – 2.95 (m, 2H), 2.68 (s, 1H), 2.64 – 2.58 (m, 1H), 2.14 – 1.99 (m, 2H), 1.70 – 1.43 (m, 3H), 1.01 – 0.88 (m, 3H), 0.80 – 0.63 (m, 2H); ^13^C NMR (126 MHz, CD_3_OD) δ 174.43, 140.14, 138.53, 130.98, 130.83, 130.46, 129.25, 127.27, 124.08, 73.13, 55.88, 54.64, 46.17, 44.93, 39.05, 36.17, 32.73, 27.15, 21.35, 8.76, 7.70; HRMS (ESI) m/z calcd. for C_25_H_34_N_4_O_4_S ([M+H]^+^): 487.2379, found 487.2392.

37. (*R*)-*N*-((2*S*,3*R*)-4-((*N*-cyclopropyl-4-methoxyphenyl)sulfonamido)-3-hydroxy-1-phenylbutan-2-yl)-1-methylpiperidine-3-carboxamide (**24a**)

Compound **24a** was prepared from **12** (27.0 mg, 0.15 mmol) and **18a** (61.4 mg, 0.16 mmol) by following the same procedure outlined for **19a** to give a yellow powder: yield 57.6 mg (74.6%); mp 86.4-88.2 ℃; ^1^H NMR (500 MHz, CDCl_3_) δ 7.78 (d, *J* = 8.5 Hz, 2H), 7.29 – 7.25 (m, 4H), 7.21 – 7.15 (m, 1H), 6.99 (d, *J* = 8.5 Hz, 2H), 4.18 (s, 1H), 4.07 – 4.03 (m, 1H), 3.87 (s, 3H), 3.57 – 3.31 (m, 4H), 3.15 – 3.07 (m, 2H), 2.91 – 2.85 (m, 1H), 2.72 (s, 1H), 2.49 – 2.36 (m, 4H), 2.07 – 2.00 (m, 1H), 1.72 – 1.56 (m, 3H), 0.96 – 0.84 (m, 3H), 0.72 – 0.63 (m, 2H); ^13^C NMR (101 MHz, CDCl_3_) δ 174.15, 163.20, 138.67, 130.01, 129.66, 129.25, 128.44, 126.40, 114.33, 72.34, 56.49, 55.75, 55.04, 54.65, 54.51, 45.03, 41.26, 35.02, 32.33, 29.80, 25.20, 7.70, 7.32; HRMS (ESI) m/z calcd. for C_27_H_37_N_3_O_5_S ([M-H]^-^): 514.2376, found 514.2386.

38. (*R*)-*N*-((2*S*,3*R*)-4-((*N*-cyclopropyl-4-(trifluoromethyl)phenyl)sulfonamido)-3-hydroxy-1-phenylbutan-2-yl)-1-methylpiperidine-3-carboxamide (**24b**)

Compound **24b** was prepared from **12** (27.0 mg, 0.15 mmol) and **18b** (68.5 mg, 0.16 mmol) by following the same procedure outlined for **19a** to give a yellow powder: yield 60.0 mg (72.3%); mp 82.9-84.7 ℃; ^1^H NMR (400 MHz, CDCl_3_) δ 7.99 (d, *J* = 8.2 Hz, 2H), 7.80 (d, *J* = 8.2 Hz, 2H), 7.32 – 7.27 (m, 2H), 7.26 – 7.18 (m, 3H), 4.17 – 4.10 (m, 1H), 4.08 – 4.02 (m, 1H), 3.58 – 3.47 (m, 1H), 3.18 – 3.04 (m, 2H), 2.96 – 2.88 (m, 1H), 2.66 – 2.50 (m, 1H), 2.47 – 2.38 (m, 2H), 2.18 – 2.13 (m, 1H), 2.11 (s, 3H), 2.09 – 1.89 (m, 2H), 1.73 – 1.65 (m, 1H), 1.47 – 1.37 (m, 1H), 1.14 – 0.98 (m, 2H), 0.91 – 0.65 (m, 4H); ^13^C NMR (101 MHz, CDCl_3_) δ 177.16, 141.80, 138.30, 134.64 (q, *J* = 33.0 Hz), 129.41, 128.63, 128.41, 126.75, 126.27 (q, *J* = 3.6 Hz), 123.36 (q, *J* = 273.1 Hz), 72.48, 56.67, 56.06, 55.53, 54.04, 45.98, 41.53, 35.21, 31.86, 25.94, 21.86, 7.78, 7.44; HRMS (ESI) m/z calcd. for C_27_H_34_F_3_N_3_O_4_S ([M-H]^-^): 552.2144, found 552.2150.

39. (*R*)-*N*-((2*S*,3*R*)-4-((*N*-cyclopropyl-4-nitrophenyl)sulfonamido)-3-hydroxy-1-phenylbutan-2-yl)-1-methylpiperidine-3-carboxamide (**24c**)

Compound **24c** was prepared from **12** (27.0 mg, 0.15 mmol) and **18c** (63.8 mg, 0.16 mmol) by following the same procedure outlined for **19a** to give a yellow powder: yield 54.4 mg (68.4%); mp 159.9-161.2 ℃; ^1^H NMR (400 MHz, CDCl_3_) δ 8.37 (d, *J* = 8.8 Hz, 2H), 8.06 (d, *J* = 8.8 Hz, 2H), 7.32 – 7.26 (m, 3H), 7.25 – 7.19 (m, 2H), 4.18 – 4.11 (m, 1H), 4.08 – 4.02 (m, 1H), 3.49 (dd, *J* = 14.4, 4.0 Hz, 1H), 3.26 (dd, *J* = 14.4, 8.0 Hz, 1H), 3.06 (dd, *J* = 14.0, 4.4 Hz, 1H), 2.92 – 2.84 (m, 1H), 2.67 – 2.51 (m, 2H), 2.48 – 2.42 (m, 1H), 2.34 – 1.92 (m, 6H), 1.72 – 1.64 (m, 1H), 1.56 – 1.34 (m, 2H), 1.22 – 0.98 (m, 2H), 0.88 – 0.70 (m, 3H); ^13^C NMR (101 MHz, CDCl_3_) δ 177.80, 150.21, 144.30, 138.14, 129.38, 129.17, 128.65, 126.80, 124.28, 72.18, 56.58, 55.99, 55.42, 53.74, 45.75, 41.34, 35.40, 31.36, 25.77, 21.82, 8.12, 7.39; HRMS (ESI) m/z calcd. for C_26_H_34_N_4_O_6_S ([M-H]^-^): 529.2121, found 529.2107.

40. (*R*)-*N*-((2*S*,3*R*)-4-((4-amino-*N*-cyclopropylphenyl)sulfonamido)-3-hydroxy-1-phenylbutan-2-yl)-1-methylpiperidine-3-carboxamide (**24d**)

Compound **24d** was prepared from **12** (17.9 mg, 0.10 mmol) and **18d** (39.4 mg, 0.11 mmol) by following the same procedure outlined for **19a** to give a yellow powder: yield 20.1 mg (40.2%); mp 75.8-77.3 ℃; ^1^H NMR (500 MHz, CD_3_OD) δ 7.54 (d, *J* = 8.5 Hz, 2H), 7.27 (s, 4H), 7.21 – 7.17 (m, 1H), 6.73 (d, *J* = 8.5 Hz, 2H), 4.11 – 4.07 (m, 1H), 3.98 – 3.93 (m, 1H), 3.41 (dd, *J* = 14.0, 3.5 Hz, 1H), 3.26 (dd, *J* = 14.0, 3.5 Hz, 1H), 2.96 (dd, *J* = 14.0, 8.5 Hz, 1H), 2.73 – 2.67 (m, 1H), 2.66 – 2.60 (m, 1H), 2.37 – 2.31 (m, 2H), 2.22 (s, 3H), 2.09 – 1.97 (m, 3H), 1.94 – 1.86 (m, 1H), 1.71 – 1.57 (m, 3H), 1.54 – 1.47 (m, 1H), 1.02 – 0.96 (m, 2H), 0.95 – 0.91 (m, 1H), 0.77 – 0.71 (m, 1H), 0.68 – 0.59 (m, 1H); ^13^C NMR (101 MHz, CD_3_OD) δ 175.97, 154.59, 140.25, 130.90, 130.47, 129.21, 127.20, 124.21, 114.24, 73.49, 58.47, 56.44, 56.28, 54.74, 46.31, 44.07, 36.53, 33.53, 27.44, 24.79, 8.86, 7.53; HRMS (ESI) m/z calcd. for C_26_H_36_N_4_O_4_S ([M-H]^-^): 499.2379, found 499.2385.

41. (*S*)-*N*-((2*S*,3*R*)-4-((*N*-cyclopropyl-4-methoxyphenyl)sulfonamido)-3-hydroxy-1-phenylbutan-2-yl)-1-methylpiperidine-3-carboxamide (**25a**)

Compound **25a** was prepared from **13** (27.0 mg, 0.15 mmol) and **18a** (61.4 mg, 0.16 mmol) by following the same procedure outlined for **19a** to give a yellow powder: yield 50.7 mg (65.6%); mp 88.9-91.2 ℃; ^1^H NMR (500 MHz, CDCl_3_) δ 7.79 (d, *J* = 8.5 Hz, 2H), 7.65 (s, 1H), 7.33 – 7.26 (m, 1H), 7.25 – 7.21 (m, 3H), 7.17 – 7.13 (m, 1H), 7.00 (d, *J* = 8.5 Hz, 2H), 4.28 – 4.22 (m, 1H), 4.12 – 4.07 (m, 1H), 3.86 (s, 3H), 3.49 – 3.29 (m, 2H), 3.21 – 3.13 (m, 1H), 3.10 – 2.96 (m, 2H), 2.86 – 2.76 (m, 2H), 2.73 (s, 3H), 2.64 – 2.52 (m, 1H), 2.05 – 1.97 (m, 2H), 1.86 – 1.70 (m, 1H), 1.66 – 1.61 (m, 1H), 0.95 – 0.80 (m, 4H), 0.78 – 0.56 (m, 2H); ^13^C NMR (101 MHz, CDCl_3_) δ 171.54, 163.18, 138.59, 130.07, 129.58, 129.32, 128.34, 126.30, 114.37, 71.65, 56.07, 55.79, 54.68, 54.50, 53.46, 44.37, 40.51, 34.45, 32.13, 29.82, 25.43, 7.91, 7.16; HRMS (ESI) m/z calcd. for C_27_H_37_N_3_O_5_S ([M-H]^-^): 514.2376, found 514.2380.

42. (*S*)-*N*-((2*S*,3*R*)-4-((*N*-cyclopropyl-4-(trifluoromethyl)phenyl)sulfonamido)-3-hydroxy-1-phenylbutan-2-yl)-1-methylpiperidine-3-carboxamide (**25b**)

Compound **25b** was prepared from **13** (27.0 mg, 0.15 mmol) and **18b** (68.5 mg, 0.16 mmol) by following the same procedure outlined for **19a** to give a yellow powder: yield 54.5 mg (65.7%); mp 102.7-104.6 ℃; ^1^H NMR (400 MHz, CDCl_3_) δ 8.01 (d, *J* = 8.0 Hz, 2H), 7.81 (d, *J* = 8.0 Hz, 2H), 7.35 – 7.28 (m, 3H), 7.26 – 7.22 (m, 2H), 4.28 – 4.20 (m, 1H), 4.06 – 4.00 (m, 1H), 3.45 (dd, *J* = 14.5, 3.8 Hz, 1H), 3.27 (dd, *J* = 14.5, 8.8 Hz, 1H), 3.11 (dd, *J* = 14.3, 4.9 Hz, 1H), 2.88 (dd, *J* = 14.3, 10.4 Hz, 1H), 2.62 (s, 1H), 2.48 – 2.40 (m, 2H), 2.26 – 2.02 (m, 6H), 1.84 – 1.42 (m, 4H), 1.01 – 0.87 (m, 2H), 0.80 – 0.69 (m, 2H); ^13^C NMR (101 MHz, CDCl_3_) δ 176.49, 141.71, 137.78, 134.48 (q, *J* = 33.2 Hz), 129.15, 128.55, 128.31, 126.65, 126.11 (q, *J* = 3.7 Hz), 123.25 (q, *J* = 273.0 Hz), 72.27, 56.77, 55.49, 54.86, 54.33, 46.12, 41.55, 35.88, 31.67, 26.04, 22.59, 7.94, 7.36; HRMS (ESI) m/z calcd. for C_27_H_34_F_3_N_3_O_4_S ([M-H]^-^): 552.2144, found 552.2158.

43. (*S*)-*N*-((2*S*,3*R*)-4-((*N*-cyclopropyl-4-nitrophenyl)sulfonamido)-3-hydroxy-1-phenylbutan-2-yl)-1-methylpiperidine-3-carboxamide (**25c**)

Compound **25c** was prepared from **13** (27.0 mg, 0.15 mmol) and **18c** (63.8 mg, 0.16 mmol) by following the same procedure outlined for **19a** to give a yellow powder: yield 73.5 mg (92.4%); mp 133.2-134.8 ℃; ^1^H NMR (400 MHz, CDCl_3_) δ 8.37 (d, *J* = 8.8 Hz, 2H), 8.09 (d, *J* = 8.8 Hz, 2H), 7.38 – 7.26 (m, 4H), 7.23 – 7.17 (m, 1H), 4.31 – 4.23 (m, 1H), 4.12 – 4.06 (m, 1H), 3.50 – 3.30 (m, 3H), 3.17 – 2.97 (m, 2H), 2.93 – 2.53 (m, 6H), 2.24 – 2.15 (m, 1H), 2.12 – 1.95 (m, 1H), 1.88 – 1.76 (m, 1H), 1.75 – 1.61 (m, 1H), 1.46 – 1.34 (m, 1H), 1.04 – 0.83 (m, 3H), 0.82 – 0.75 (m, 1H), 0.74 – 0.67 (m, 1H); ^13^C NMR (101 MHz, CDCl_3_) δ 171.32, 150.17, 144.31, 138.26, 129.47, 129.25, 128.45, 126.50, 124.33, 71.42, 56.18, 54.81, 53.92, 53.79, 44.48, 40.62, 34.62, 31.22, 25.54, 22.38, 8.38, 7.36; HRMS (ESI) m/z calcd. for C_26_H_34_N_4_O_6_S ([M-H]^-^): 529.2121, found 529.2115.

44. (*S*)-*N*-((2*S*,3*R*)-4-((4-amino-*N*-cyclopropylphenyl)sulfonamido)-3-hydroxy-1-phenylbutan-2-yl)-1-methylpiperidine-3-carboxamide (**25d**)

Compound **25d** was prepared from **13** (17.9 mg, 0.10 mmol) and **18d** (39.4 mg, 0.11 mmol) by following the same procedure outlined for **19a** to give a yellow powder: yield 18.1 mg (36.2%); mp 76.8-78.5 ℃; ^1^H NMR (500 MHz, CD_3_OD) δ 7.54 (d, *J* = 8.5 Hz, 2H), 7.28 – 7.24 (m, 4H), 7.20 – 7.16 (m, 1H), 6.74 (d, *J* = 8.5 Hz, 2H), 4.11 – 4.06 (m, 1H), 3.96 – 3.92 (m, 1H), 3.42 – 3.37 (m, 1H), 3.28 – 3.24 (m, 1H), 2.99 – 2.94 (m, 1H), 2.76 – 2.67 (m, 2H), 2.66 – 2.60 (m, 1H), 2.38 – 2.33 (m, 1H), 2.29 (s, 3H), 2.24 – 2.14 (m, 1H), 2.11 – 1.98 (m, 3H), 1.63 (s, 1H), 1.55 – 1.44 (m, 2H), 1.20 – 1.11 (m, 1H), 1.01 – 0.90 (m, 3H), 0.77 – 0.71 (m, 1H), 0.66 – 0.61 (m, 1H); ^13^C NMR (101 MHz, CD_3_OD) δ 175.98, 154.59, 140.20, 130.91, 130.44, 129.17, 127.17, 124.21, 114.24, 73.47, 58.33, 56.50, 56.28, 54.66, 46.44, 43.93, 36.55, 33.52, 28.11, 27.78, 8.85, 7.53; HRMS (ESI) m/z calcd. for C_26_H_36_N_4_O_4_S ([M-H]^-^): 499.2379, found 499.2380.

45. *N*-((2*S*,3*R*)-4-((*N*-cyclopropyl-4-methoxyphenyl)sulfonamido)-3-hydroxy-1-phenylbutan-2-yl)piperidine-4-carboxamide (**26a**)

Compound **26a** was prepared from **21a** (65.0 mg, 0.11 mmol) by following the same procedure outlined for **22a** to give a white powder: yield 46.4 mg (84.2%); mp 219.9-221.3 ℃; ^1^H NMR (500 MHz, CD_3_OD) δ 7.83 (d, *J* = 8.0 Hz, 2H), 7.30 – 7.24 (m, 4H), 7.19 (s, 1H), 7.14 (d, *J* = 8.0 Hz, 2H), 4.15 (s, 1H), 3.97 (s, 1H), 3.92 (s, 3H), 3.52 (d, *J* = 14.0 Hz, 1H), 3.40 (d, *J* = 12.0 Hz, 1H), 3.26 (d, *J* = 13.5 Hz, 1H), 3.18 (d, *J* = 12.0 Hz, 1H), 3.06 – 2.98 (m, 2H), 2.96 – 2.90 (m, 1H), 2.65 (t, *J* = 12.5 Hz, 1H), 2.50 – 2.43 (m, 1H), 2.07 (s, 1H), 1.93 – 1.78 (m, 2H), 1.65 – 1.58 (m, 1H), 1.56 – 1.46 (m, 1H), 1.00 – 0.93 (m, 2H), 0.80 – 0.63 (m, 2H); ^13^C NMR (126 MHz, CD_3_OD) δ 175.26, 164.78, 140.25, 131.09, 130.56, 130.45, 129.21, 127.21, 115.31, 73.43, 56.26, 56.17, 54.68, 44.08, 44.07, 40.48, 36.19, 33.19, 26.77, 26.13, 8.72, 7.69; HRMS (ESI) m/z calcd. for C_26_H_35_N_3_O_5_S ([M+H]^+^): 502.2375, found 502.2352

46. *N*-((2*S*,3*R*)-4-((*N*-cyclopropyl-4-(trifluoromethyl)phenyl)sulfonamido)-3-hydroxy-1-phenylbutan-2-yl)piperidine-4-carboxamide (**26b**)

Compound **26b** was prepared from **21b** (70.0 mg, 0.11 mmol) by following the same procedure outlined for **22a** to give a white powder: yield 57.3 mg (96.6%); mp 243.3-245.1 ℃; ^1^H NMR (500 MHz, CD_3_OD) δ 8.10 (d, *J* = 8.0 Hz, 2H), 7.96 (d, *J* = 8.0 Hz, 2H), 7.29-7.25 (m, 4H), 7.21-7.17 (m, 1H), 4.17-4.11 (m, 1H), 3.99-3.91 (m, 1H), 3.58-3.51 (m, 1H), 3.43-3.37 (m, 1H), 3.27-3.15 (m, 3H), 3.05-2.91 (m, 2H), 2.70-2.63 (m, 1H), 2.51-2.45 (m, 1H), 2.21-2.15 (m, 1H), 1.93-1.78 (m, 2H), 1.65-1.59 (m, 1H), 1.56-1.47 (m, 1H), 1.07 – 0.95 (m, 2H), 0.85 – 0.70 (m, 2H); ^13^C NMR (126 MHz, CD_3_OD) δ 175.35 , 143.34 , 140.17 , 135.26 (q, *J* = 32.8 Hz), 130.44 , 129.77 , 129.23 , 127.35 (q, *J* = 3.7 Hz), 127.24 , 124.93 (q, *J* = 272.1 Hz), 73.13 , 55.92 , 54.77 , 44.07 , 40.49 , 36.26 , 32.66 , 26.77 , 26.11 , 8.87 , 7.81; HRMS (ESI) m/z calcd. for C_26_H_32_F_3_N_3_O_4_S ([M+H]^+^): 540.2144, found 540.2152.

47. *N*-((2*S*,3*R*)-4-((*N*-cyclopropyl-4-nitrophenyl)sulfonamido)-3-hydroxy-1-phenylbutan-2-yl)piperidine-4-carboxamide (**26c**)

Compound **26c** was prepared from **21c** (100.0 mg, 0.16 mmol) by following the same procedure outlined for **22a** to give a white powder: yield 58.7 mg (71.1%); mp 186.2-187.7 ℃; ^1^H NMR (400 MHz, CD_3_OD) δ 8.43 (d, *J* = 8.8 Hz, 2H), 8.10 (d, *J* = 8.8 Hz, 2H), 7.27 – 7.21 (m, 4H), 7.19 – 7.13 (m, 1H), 4.09 – 4.02 (m, 1H), 3.94 – 3.88 (m, 1H), 3.46 (dd, *J* = 14.8, 3.6 Hz, 1H), 3.24 – 3.15 (m, 2H), 3.08 – 3.01 (m, 1H), 2.98 – 2.92 (m, 1H), 2.68 – 2.50 (m, 3H), 2.26 – 2.15 (m, 2H), 1.65 – 1.48 (m, 2H), 1.40 – 1.32 (m, 2H), 1.06 – 0.91 (m, 2H), 0.86 – 0.78 (m, 1H), 0.75 – 0.67 (m, 1H); ^13^C NMR (101 MHz, CD_3_OD) δ 177.18, 151.66, 145.19, 140.08, 130.37, 130.34, 129.20, 127.20, 125.30, 73.16, 55.96, 54.78, 45.94, 45.90, 43.80, 36.50, 32.54, 29.79, 29.54, 9.05, 7.79; HRMS (ESI) m/z calcd. for C_25_H_32_N_4_O_6_S ([M+H]^+^): 517.2121, found 517.2130.

48. *N*-((2*S*,3*R*)-4-((4-amino-*N*-cyclopropylphenyl)sulfonamido)-3-hydroxy-1-phenylbutan-2-yl)piperidine-4-carboxamide (**26d**)

Compound **26d** was prepared from **21d** (20.0 mg, 0.03 mmol) by following the same procedure outlined for **22a** to give a white powder: yield 14.7 mg (89.0%); mp 204.7-206.5 ℃; ^1^H NMR (500 MHz, CD_3_OD) δ 7.55 (d, *J* = 8.0 Hz, 2H), 7.29-7.17 (m, 5H), 6.75 (d, *J* = 8.0 Hz, 2H), 4.17-4.11 (m, 1H), 3.96 (s, 1H), 3.48-3.46 (m, 1H), 3.45-3.43 (m, 1H), 3.26-3.23 (m, 1H), 3.22-3.16 (m, 1H), 3.04-2.89 (m, 3H), 2.68-2.61 (m, 1H), 2.49-2.43 (m, 1H), 2.08-2.02 (m, 1H), 1.94-1.77 (m, 2H), 1.66-1.58 (m, 1H), 1.55-1.47 (m, 1H), 0.99-0.92 (m, 2H), 0.77-0.62 (m, 2H); ^13^C NMR (126 MHz, CD_3_OD) δ 175.25, 154.61, 140.28, 130.92, 130.46, 129.20, 127.20, 124.24, 114.25, 73.47, 56.10, 54.70, 44.09, 44.07, 40.51, 36.33, 33.43, 26.75, 26.14, 8.75, 7.60; HRMS (ESI) m/z calcd. for C_25_H_34_N_4_O_4_S ([M+H]^+^): 487.2379, found 487.2378.

49. *N*-((2*S*,3*R*)-4-((*N*-cyclopropyl-4-methoxyphenyl)sulfonamido)-3-hydroxy-1-phenylbutan-2-yl)-2-(piperidin-1-yl)acetamide (**27a**)

Compound **27a** was prepared from **14** (21.4 mg, 0.15 mmol) and **18a** (61.4 mg, 0.16 mmol) by following the same procedure outlined for **19a** to give a yellow powder: yield 31.7 mg (41.0%); mp 63.6-65.8 ℃; ^1^H NMR (500 MHz, CDCl_3_) δ 7.78 (d, *J* = 8.5 Hz, 2H), 7.50 (s, 1H), 7.29 – 7.26 (m, 2H), 7.26 – 7.23 (m, 2H), 7.22 – 7.17 (m, 1H), 6.99 (d, *J* = 8.5 Hz, 2H), 4.23 (s, 1H), 4.20 – 4.13 (m, 1H), 4.00 – 3.95 (m, 1H), 3.88 (s, 3H), 3.36 – 3.31 (m, 1H), 3.17 – 3.07 (m, 2H), 2.99 – 2.92 (m, 1H), 2.90 – 2.75 (m, 2H), 2.26 – 2.18 (m, 3H), 2.11 – 2.05 (m, 1H), 1.67 (s, 1H), 1.46 – 1.35 (m, 6H), 0.90 – 0.86 (m, 2H), 0.71 – 0.65 (m, 2H); ^13^C NMR (101 MHz, CDCl_3_) δ 171.86, 163.30, 138.06, 130.04, 129.41, 129.13, 128.64, 126.63, 114.37, 72.75, 62.22, 55.78, 54.98, 54.90, 54.05, 34.97, 32.63, 26.16, 23.63, 7.51, 7.43; HRMS (ESI) m/z calcd. for C_27_H_37_N_3_O_5_S ([M-H]^-^): 514.2376, found 514.2377.

50. *N*-((2*S*,3*R*)-4-((*N*-cyclopropyl-4-(trifluoromethyl)phenyl)sulfonamido)-3-hydroxy-1-phenylbutan-2-yl)-2-(piperidin-1-yl)acetamide (**27b**)

Compound **27b** was prepared from **14** (28.6 mg, 0.2 mmol) and **18b** (89.9 mg, 0.21 mmol) by following the same procedure outlined for **19a** to give a white powder: yield 51.0 mg (46.1%); mp 72.3-75.2 ℃; ^1^H NMR (500 MHz, CDCl_3_) δ 7.99 (d, *J* = 8.0 Hz, 2H), 7.80 (d, *J* = 8.0 Hz, 2H), 7.51 (s, 1H), 7.31 – 7.26 (m, 3H), 7.25 – 7.19 (m, 2H), 4.31 (s, 1H), 4.18 – 4.11 (m, 1H), 4.04 – 3.97 (m, 1H), 3.44 – 3.38 (m, 1H), 3.24 – 3.17 (m, 1H), 3.13 – 3.07 (m, 1H), 2.98 – 2.76 (m, 3H), 2.28 – 2.13 (m, 5H), 1.45 – 1.35 (m, 6H), 0.97 – 0.87 (m, 2H), 0.77 – 0.68 (m, 2H); ^13^C NMR (101 MHz, CDCl_3_) δ 172.21, 141.54, 137.79, 134.72 (q, *J* = 33.0 Hz), 129.32, 128.74, 128.40, 126.78, 126.32 (q, *J* = 3.7 Hz), 123.32 (q, *J* = 273.7 Hz), 72.52, 62.18, 55.00, 54.55, 54.49, 35.09, 32.10, 26.19, 23.63, 7.70, 7.50; HRMS (ESI) m/z calcd. for C_27_H_34_F_3_N_3_O_4_S ([M-H]^-^): 552.2144, found 552.2164.

51. *N*-((2*S*,3*R*)-4-((*N*-cyclopropyl-4-nitrophenyl)sulfonamido)-3-hydroxy-1-phenylbutan-2-yl)-2-(piperidin-1-yl)acetamide (**27c**)

Compound **27c** was prepared from **14** (57.2 mg, 0.40 mmol) and **18c** (170.1 mg, 0.42 mmol) by following the same procedure outlined for **19a** to give a white powder: yield 97.6 mg (46.0%); mp 82.9-84.1 ℃; ^1^H NMR (500 MHz, CDCl_3_) δ 8.38 (d, *J* = 8.5 Hz, 2H), 8.05 (d, *J* = 8.5 Hz, 2H), 7.53 (s, 1H), 7.34 – 7.26 (m, 3H), 7.26 – 7.21 (m, 2H), 4.39 (s, 1H), 4.15 (s, 1H), 4.02 (s, 1H), 3.44 (d, *J* = 14.5 Hz, 1H), 3.31 – 3.25 (m, 1H), 3.10 (d, *J* = 14.0 Hz, 1H), 2.97 – 2.88 (m, 2H), 2.84 – 2.78 (m, 1H), 2.30 – 2.20 (m, 5H), 1.46 – 1.36 (m, 6H), 1.00 – 0.86 (m, 2H), 0.81 – 0.71 (m, 2H); ^13^C NMR (101 MHz, CDCl_3_) δ 172.39, 150.26, 144.06, 137.60, 129.25, 129.13, 128.79, 126.87, 124.32, 72.30, 62.13, 55.00, 54.78, 54.21, 35.25, 31.67, 26.18, 23.60, 7.94, 7.47; HRMS (ESI) m/z calcd. for C_26_H_34_N_4_O_6_S ([M-H]^-^): 529.2121, found 529.2120.

52. *N*-((2*S*,3*R*)-4-((4-amino-*N*-cyclopropylphenyl)sulfonamido)-3-hydroxy-1-phenylbutan-2-yl)-2-(piperidin-1-yl)acetamide (**27d**)

Compound **27d** was prepared from **14** (21.4 mg, 0.15 mmol) and **18d** (59.0 mg, 0.16 mmol) by following the same procedure outlined for **19a** to give a yellow powder: yield 26.0 mg (34.7%); mp 144.4-146.2 ℃; ^1^H NMR (500 MHz, CDCl_3_) δ 7.61 (d, *J* = 8.5 Hz, 2H), 7.50 (s, 1H), 7.35 – 7.26 (m, 3H), 7.25 – 7.17 (m, 2H), 6.68 (d, *J* = 8.5 Hz, 2H), 4.23 – 4.14 (m, 4H), 3.99 – 3.94 (m, 1H), 3.34 – 3.27 (m, 1H), 3.15 – 3.08 (m, 2H), 2.97 – 2.75 (m, 3H), 2.26 – 2.19 (m, 3H), 2.09 – 2.04 (m, 1H), 1.94 – 1.89 (m, 1H), 1.46 – 1.34 (m, 6H), 0.90 – 0.87 (m, 2H), 0.70 – 0.64 (m, 2H); ^13^C NMR (101 MHz, CDCl_3_) δ 170.90, 150.88, 138.00, 129.93, 129.31, 128.49, 126.47, 125.32, 114.00, 72.65, 62.08, 54.89, 54.84, 53.75, 34.86, 32.63, 26.02, 23.52, 7.40, 7.29; HRMS (ESI) m/z calcd. for C_26_H_36_N_4_O_4_S ([M-H]^-^): 499.2379, found 499.2379.
