# Supplementary material for: Piperidine scaffold as the novel P2-ligands in cyclopropyl-containing HIV-1 protease inhibitors: Structure-based design, synthesis, biological evaluation and docking study
Source: PLoS One. 2020 Jul 22;15(7):e0235483. doi: 10.1371/journal.pone.0235483 (PMC7375528; doi:10.1371/journal.pone.0235483)
Supplement: S2 Appendix — (DOCX) [file pone.0235483.s002.docx]

**S2 Appendix. ^1^H NMR, ^13^C NMR and HR MS spectrums of compounds.**


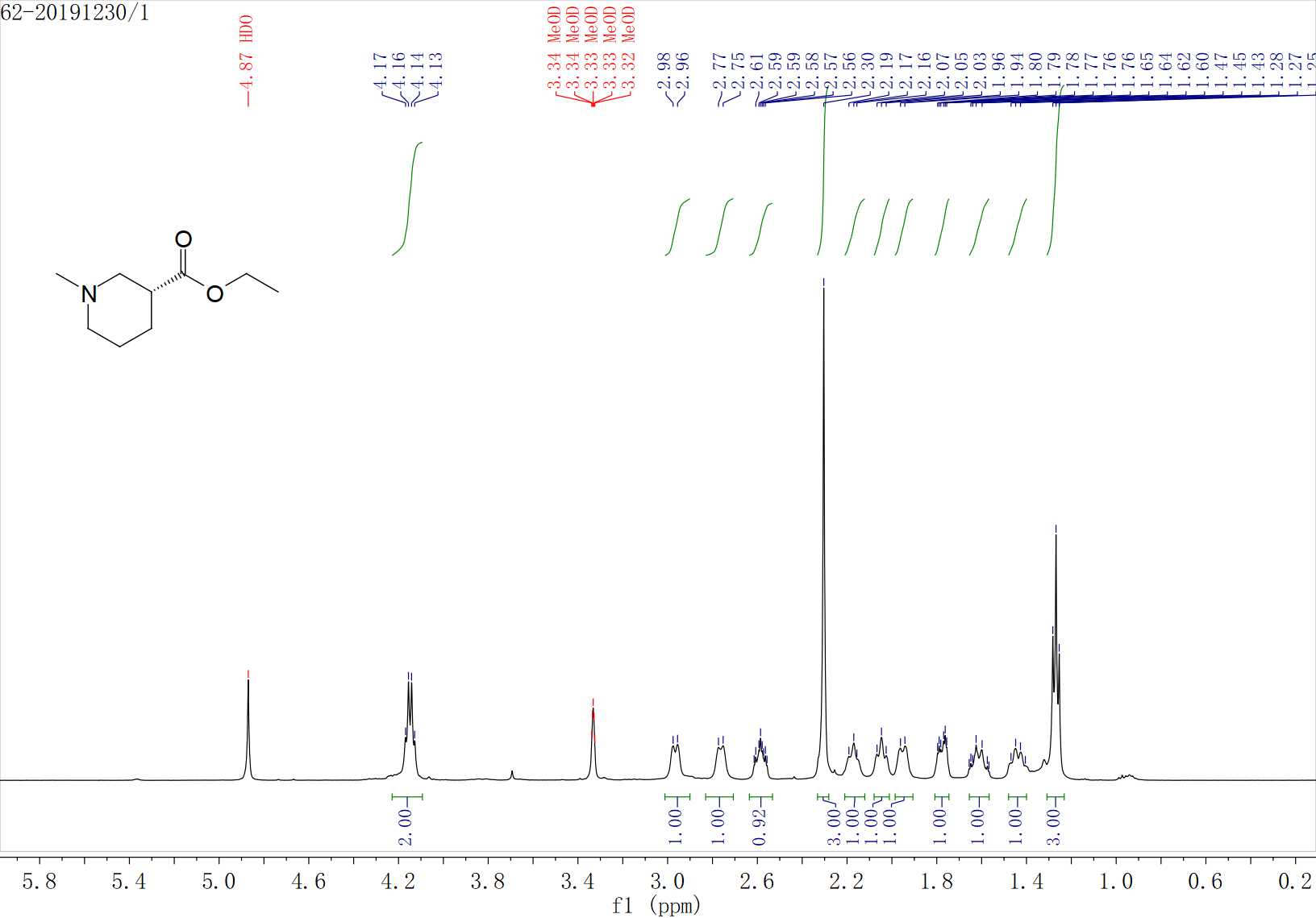


**Fig. S1.** ^1^H NMR Spectrum of compound **7**


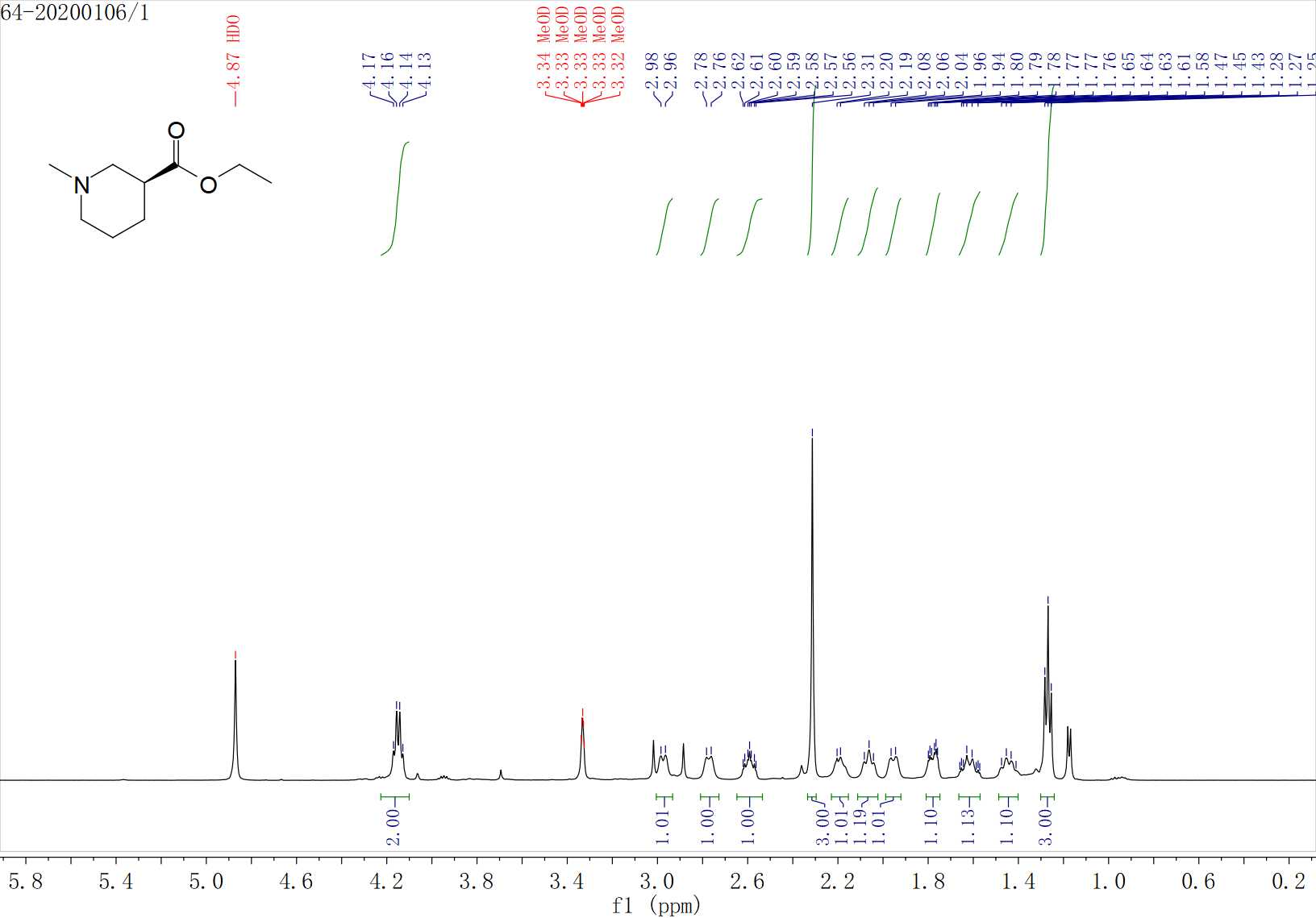


**Fig. S2.** ^1^H NMR Spectrum of compound **8**


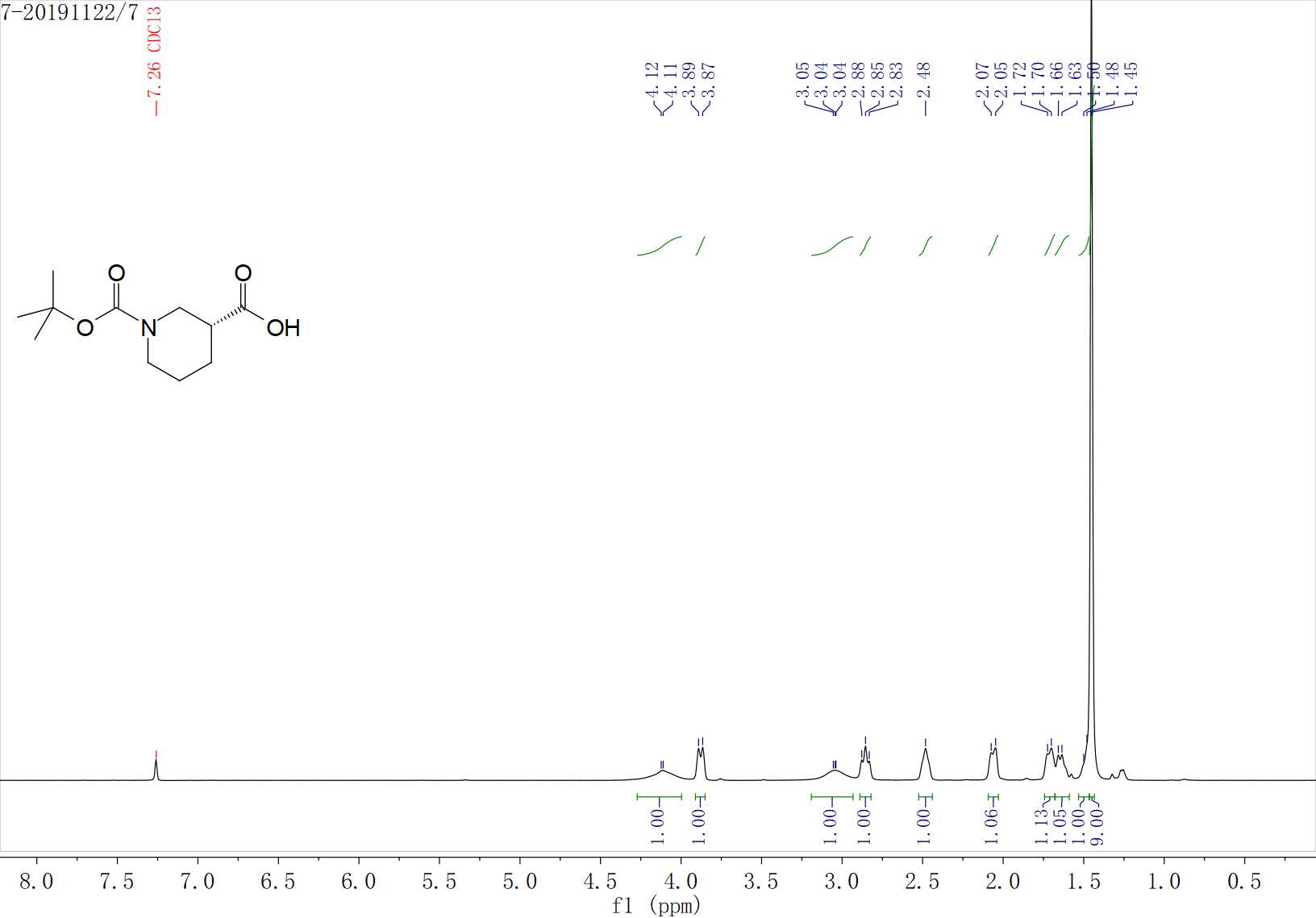


**Fig. S3.** ^1^H NMR Spectrum of compound **9**


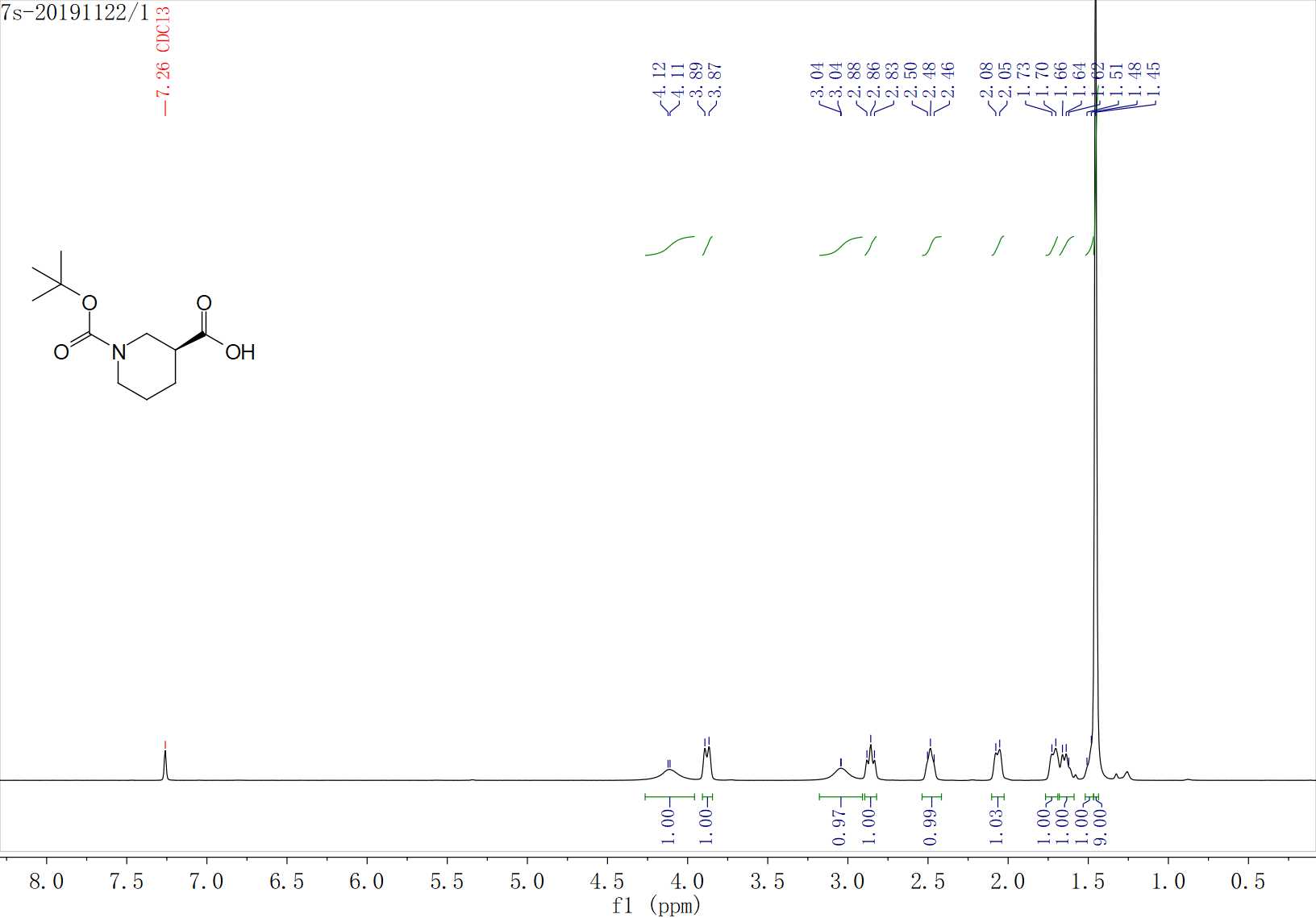


**Fig. S4.** ^1^H NMR Spectrum of compound **10**


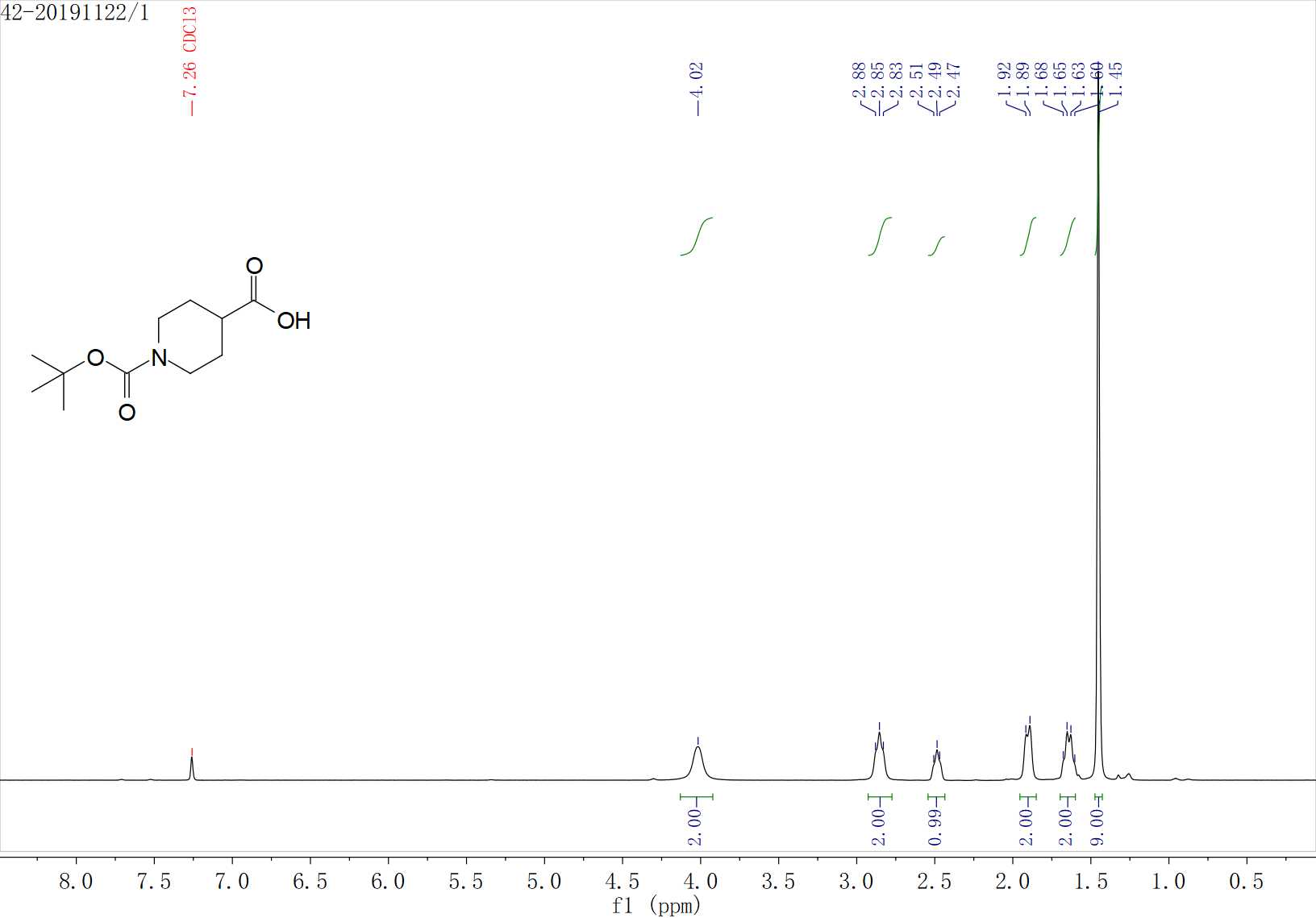


**Fig. S5.** ^1^H NMR Spectrum of compound **11**


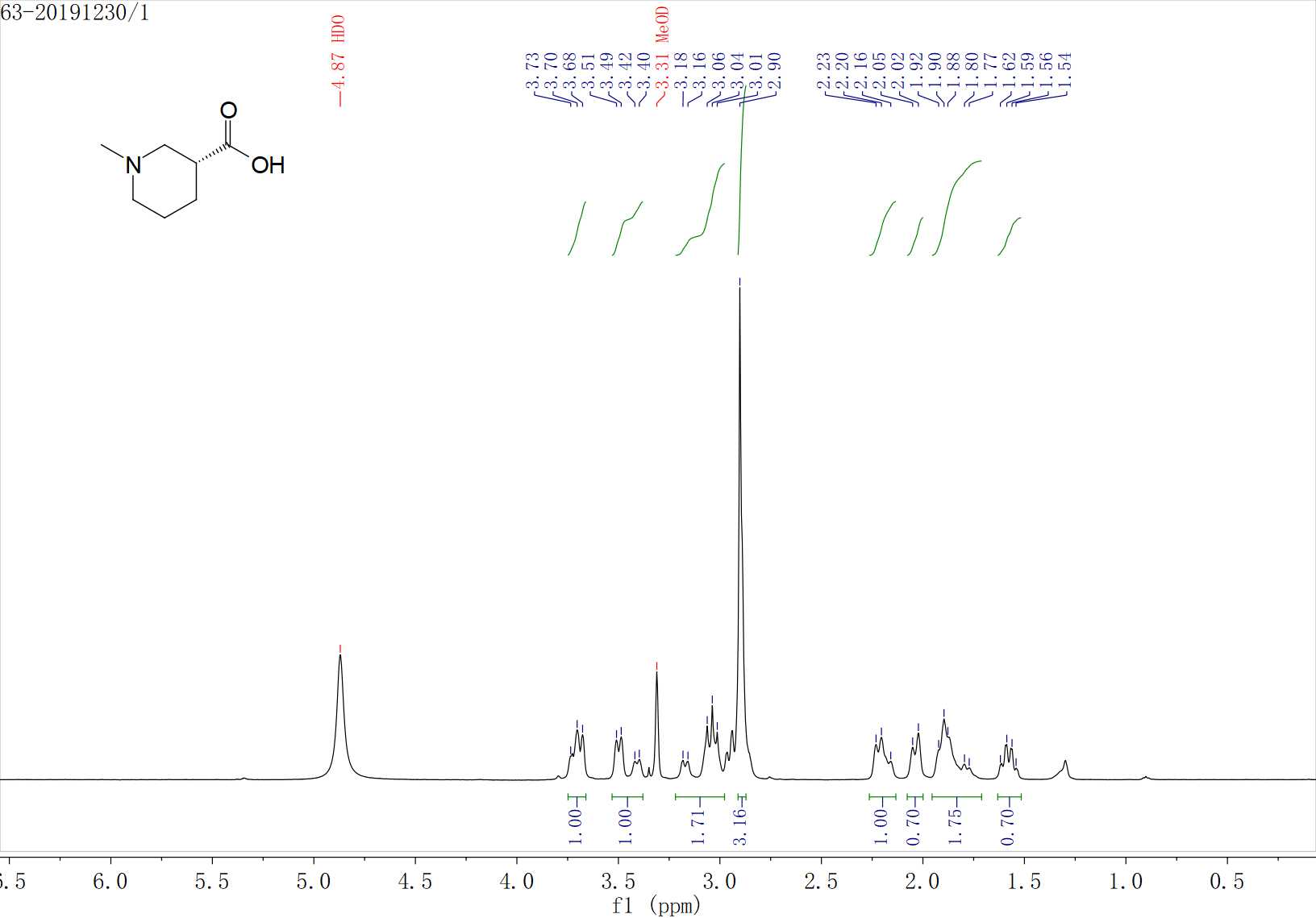


**Fig. S6.** ^1^H NMR Spectrum of compound **12**


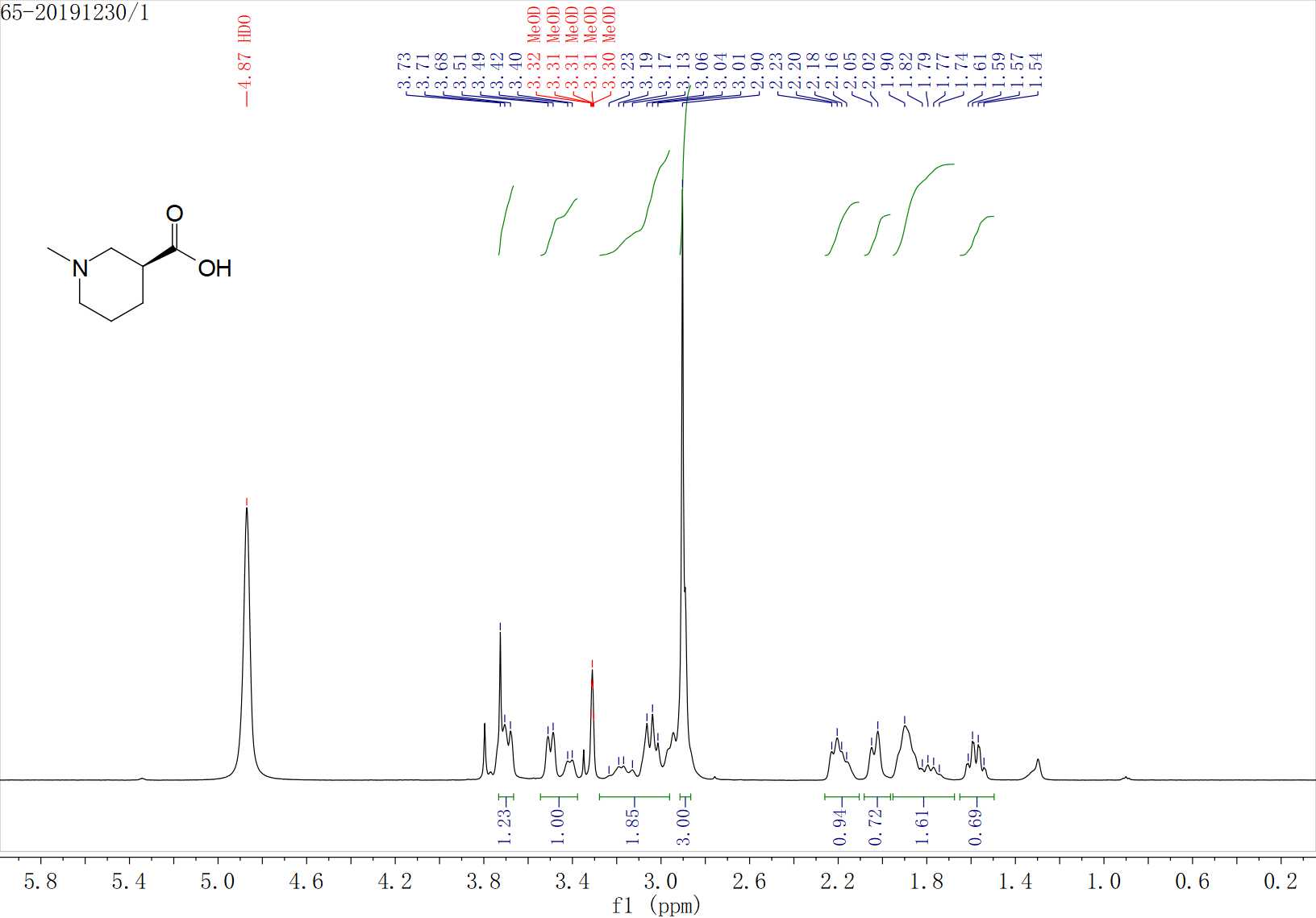


**Fig. S7.** ^1^H NMR Spectrum of compound **13**


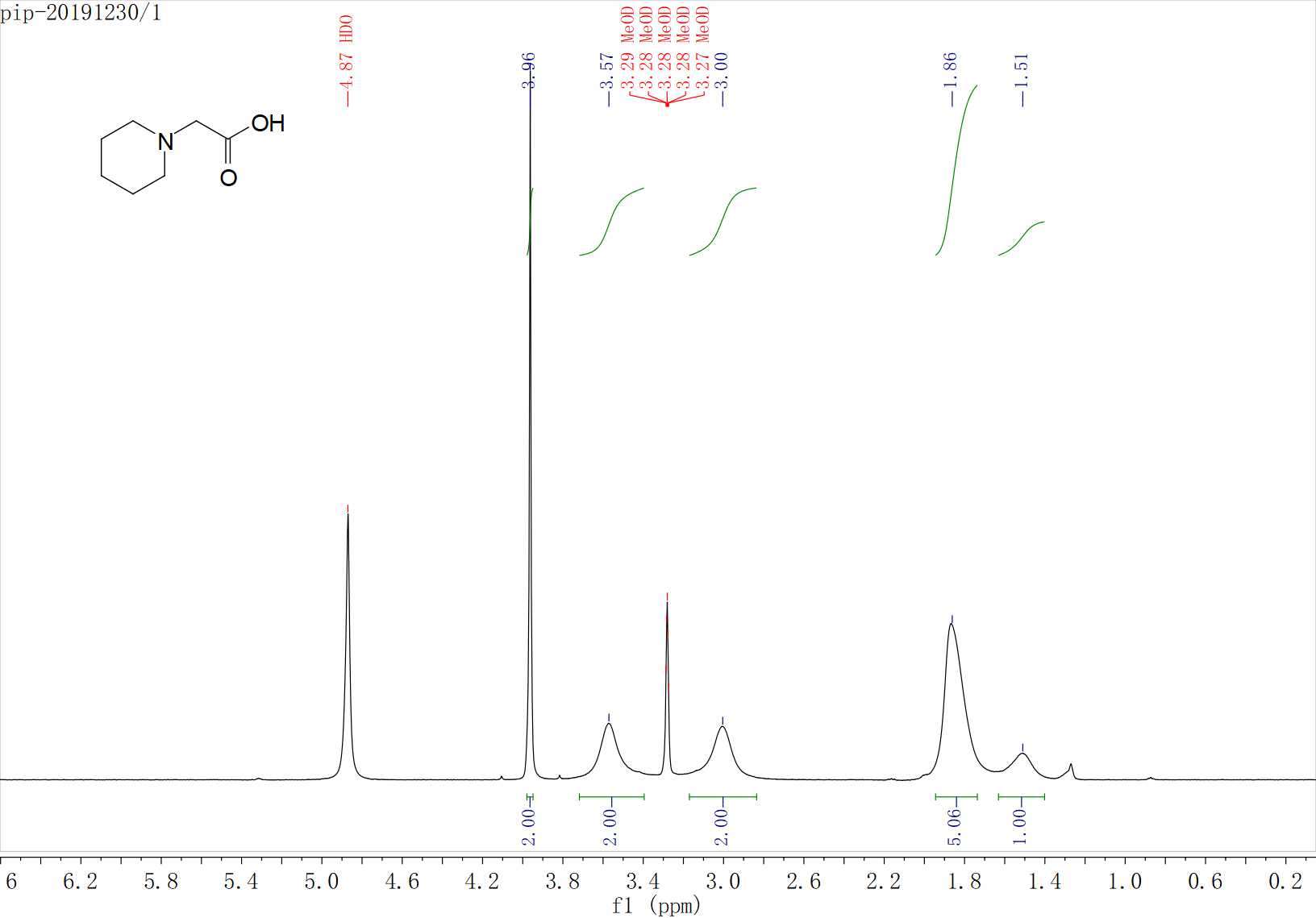


**Fig. S8.** ^1^H NMR Spectrum of compound **14**


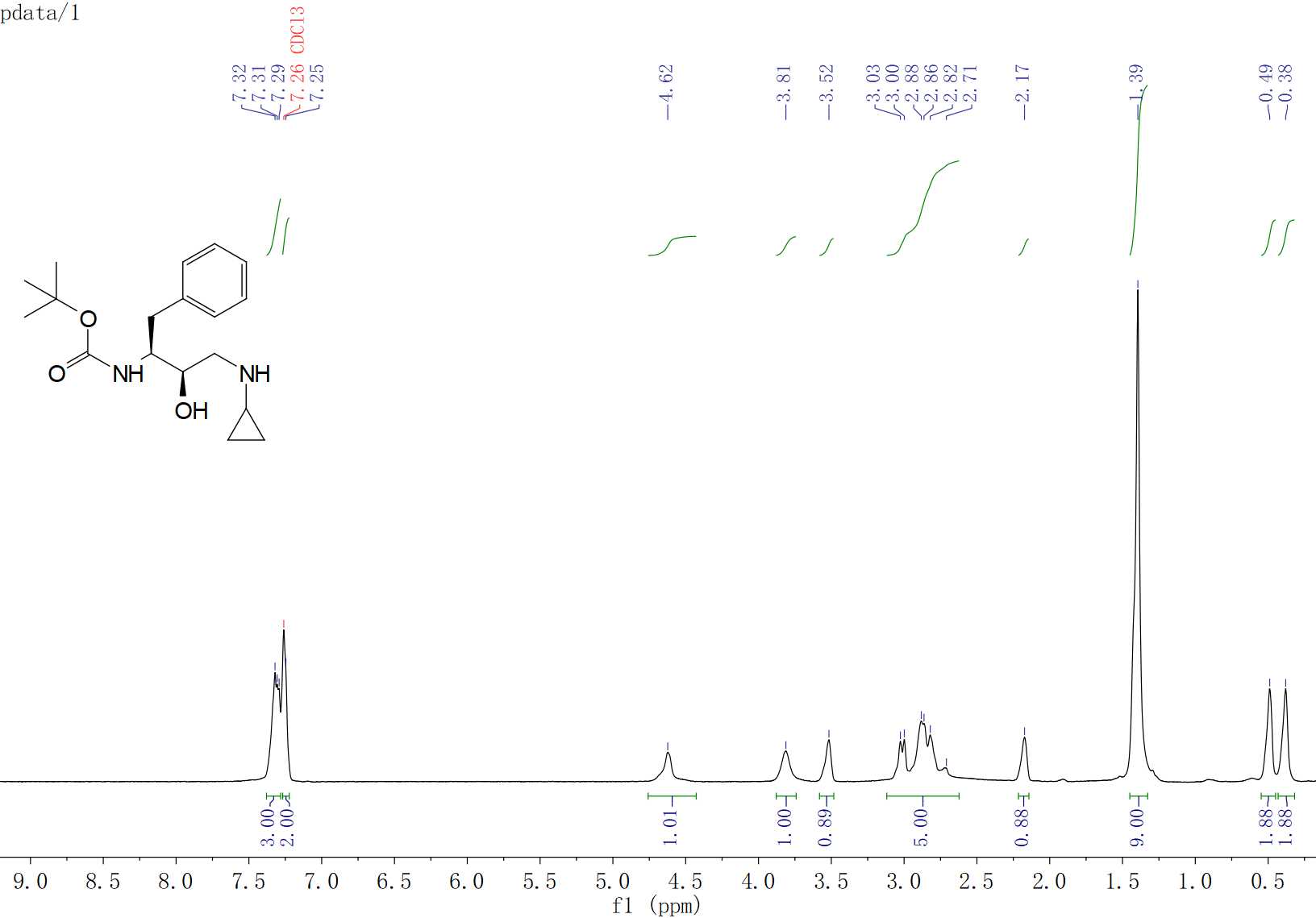


**Fig. S9.** ^1^H NMR Spectrum of compound **16**


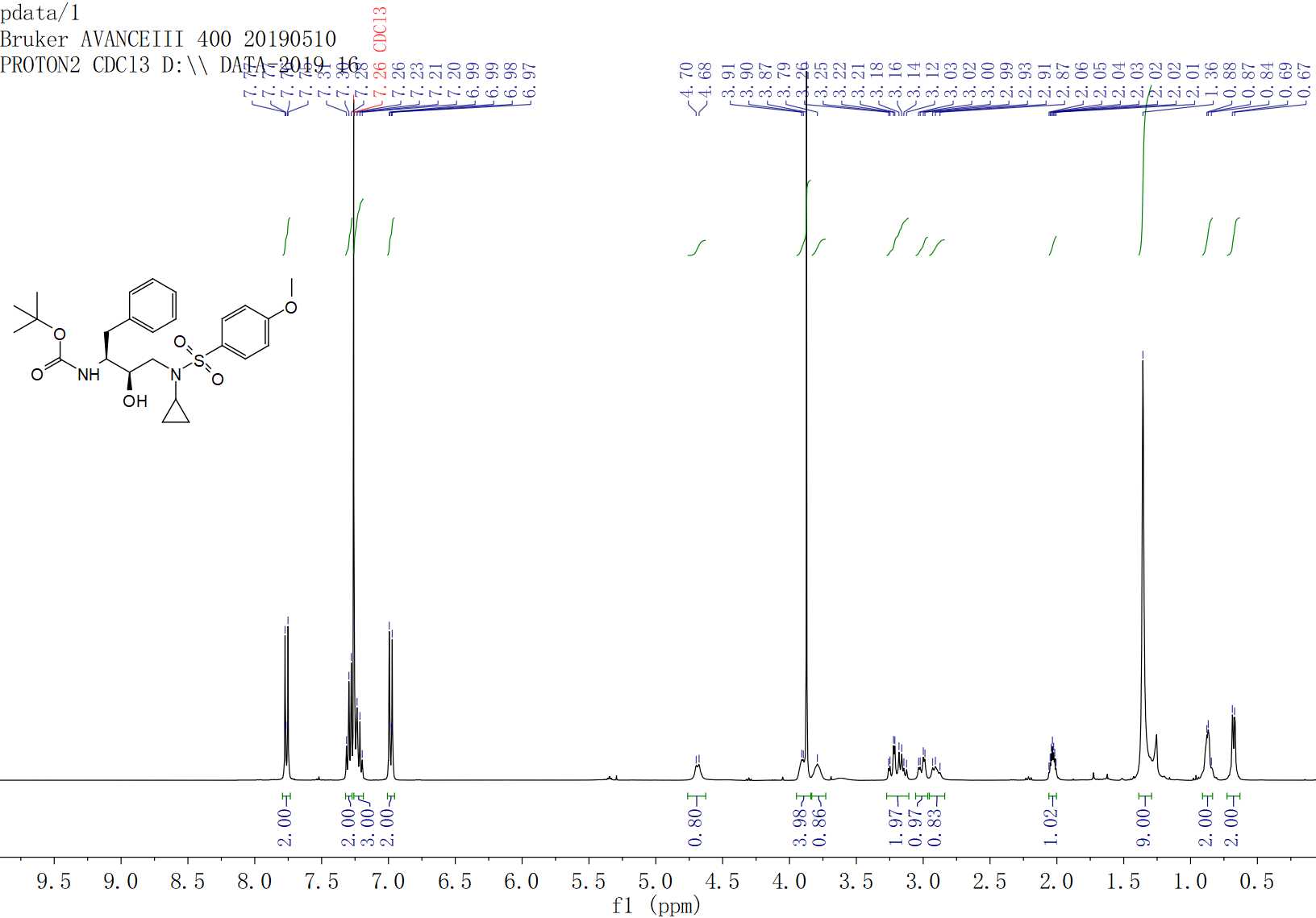


**Fig. S10.** ^1^H NMR Spectrum of compound **17a**


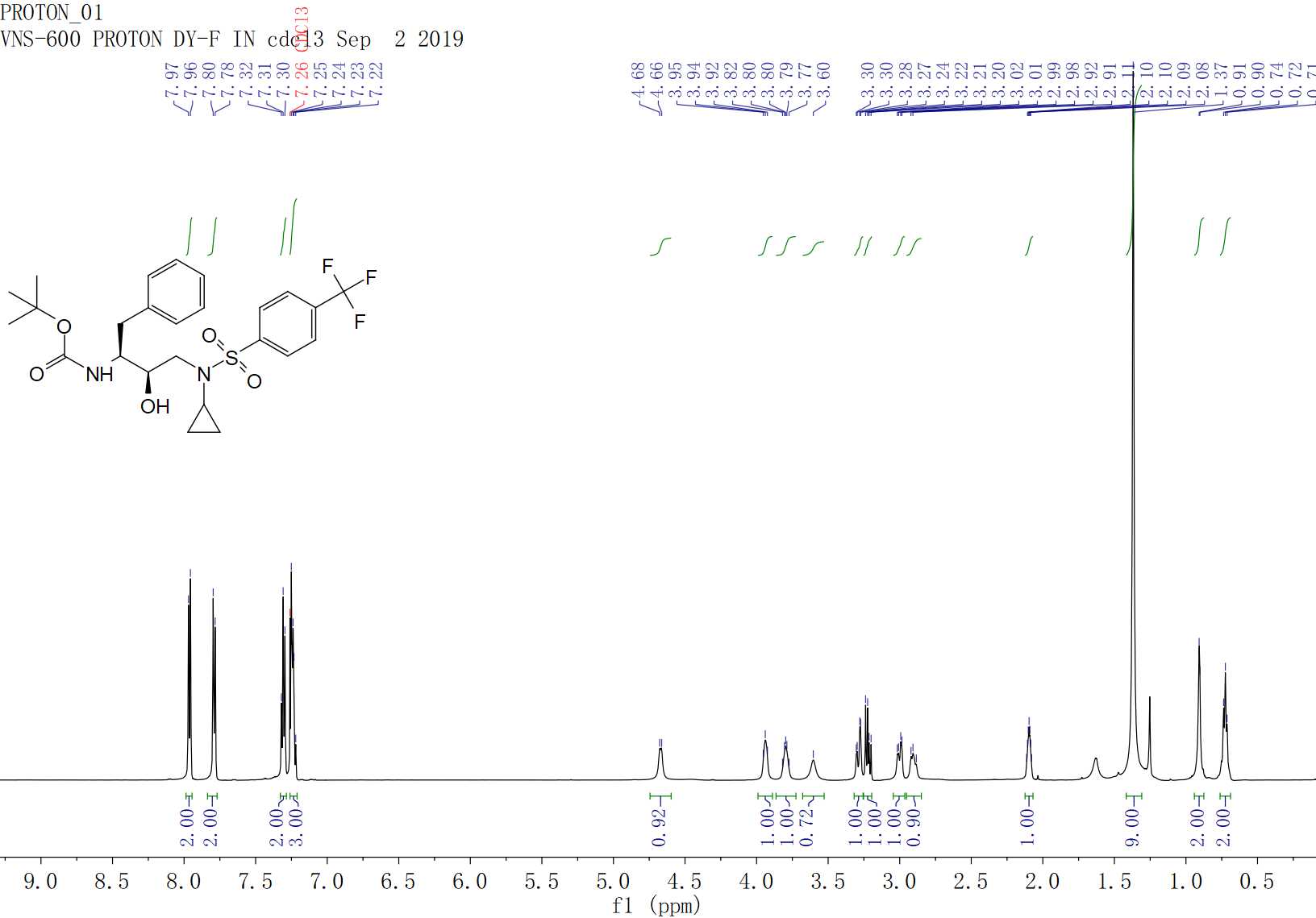


**Fig. S11.** ^1^H NMR Spectrum of compound **17b**


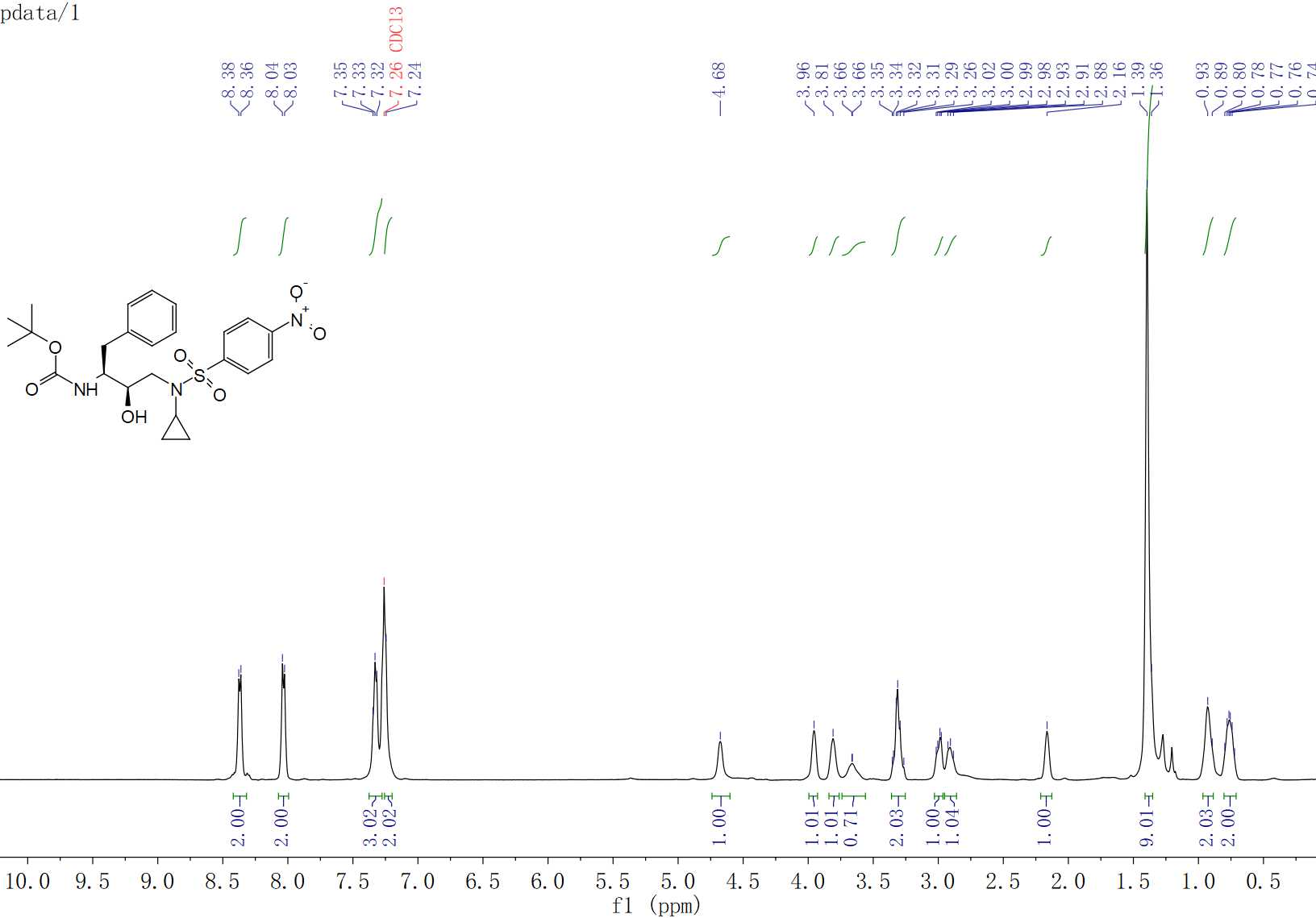


**Fig. S12.** ^1^H NMR Spectrum of compound **17c**


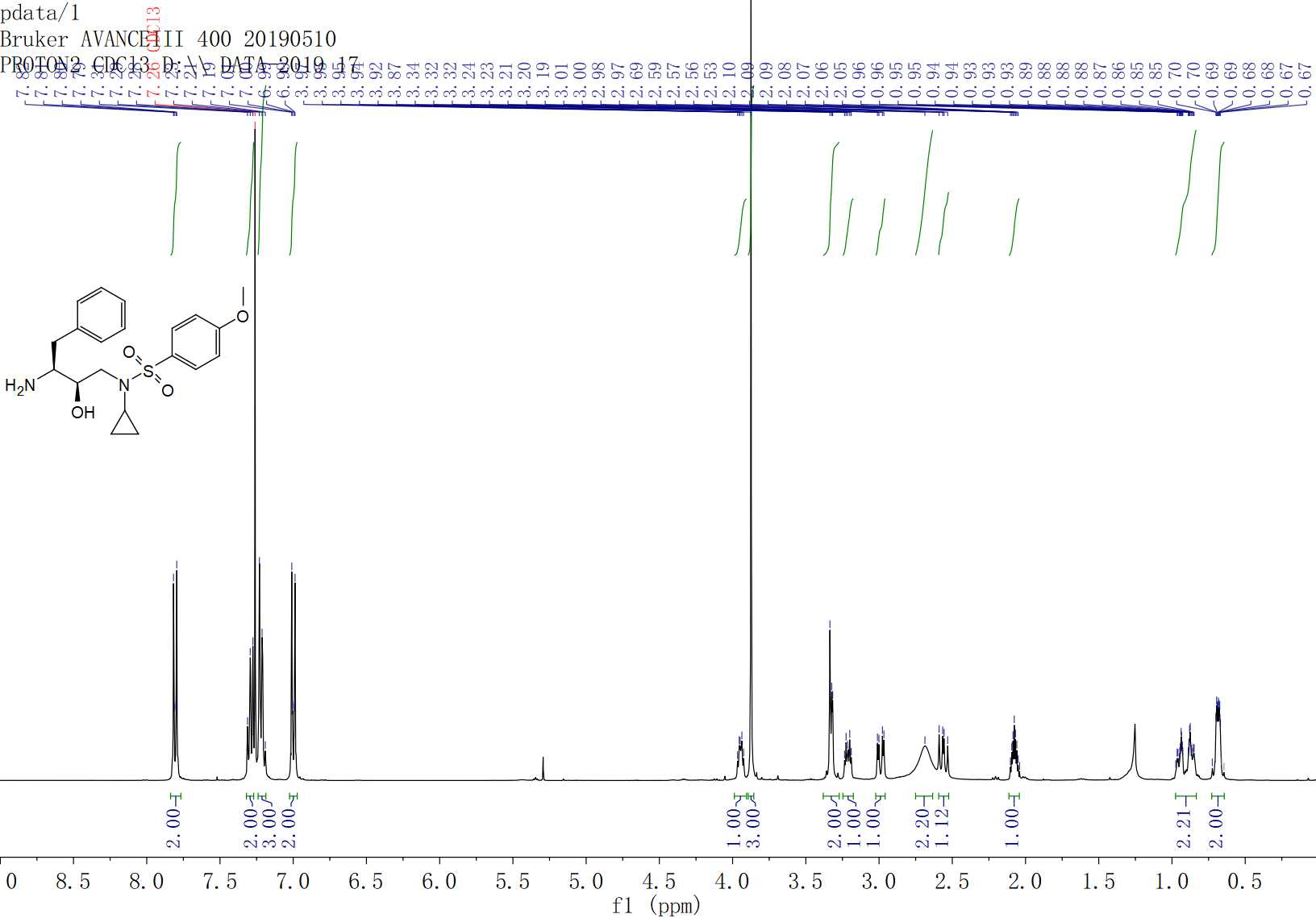


**Fig. S13.** ^1^H NMR Spectrum of compound **18a**


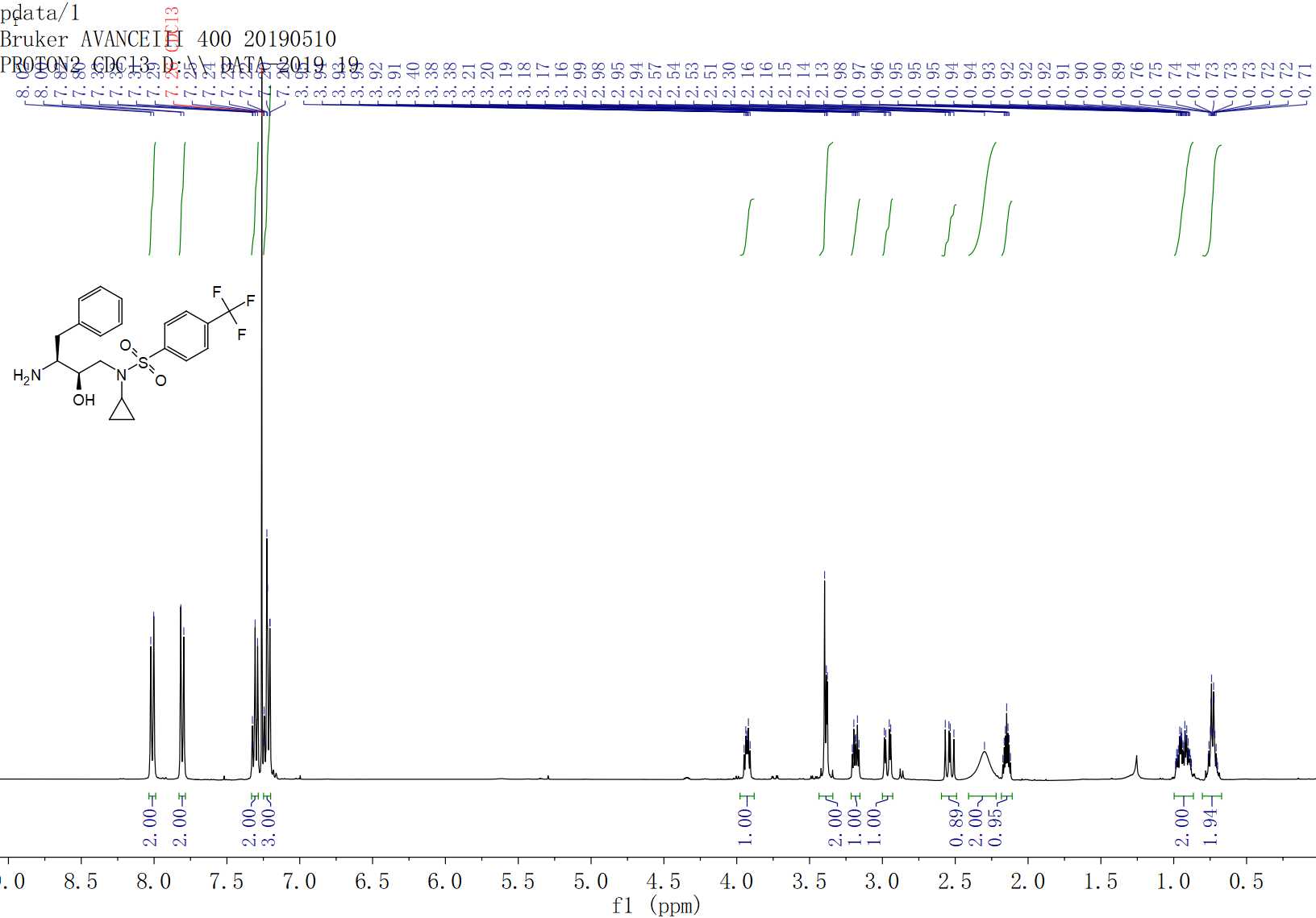


**Fig. S14.** ^1^H NMR Spectrum of compound **18b**


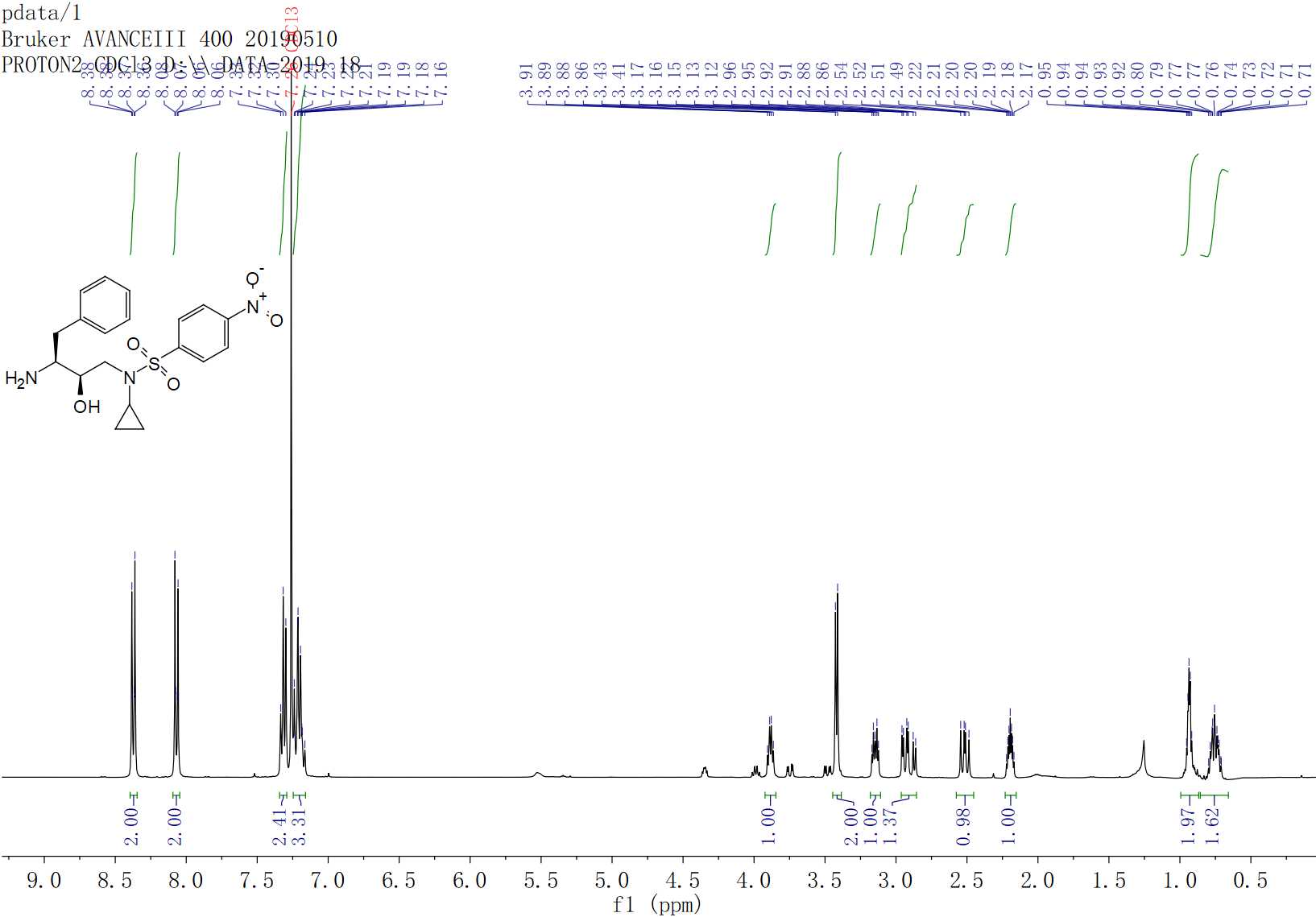


**Fig. S15.** ^1^H NMR Spectrum of compound **18c**


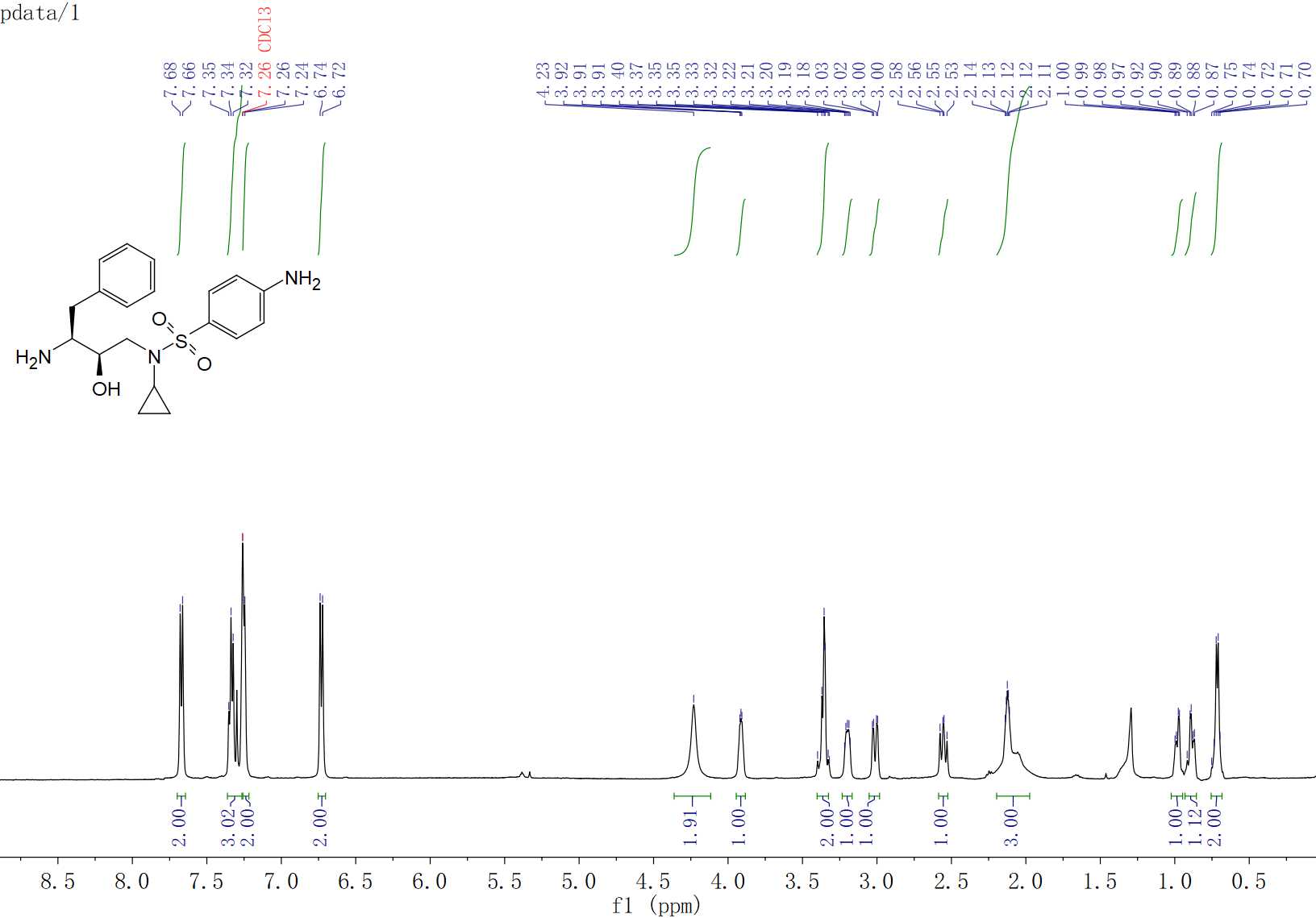


**Fig. S16.** ^1^H NMR Spectrum of compound **18d**

**
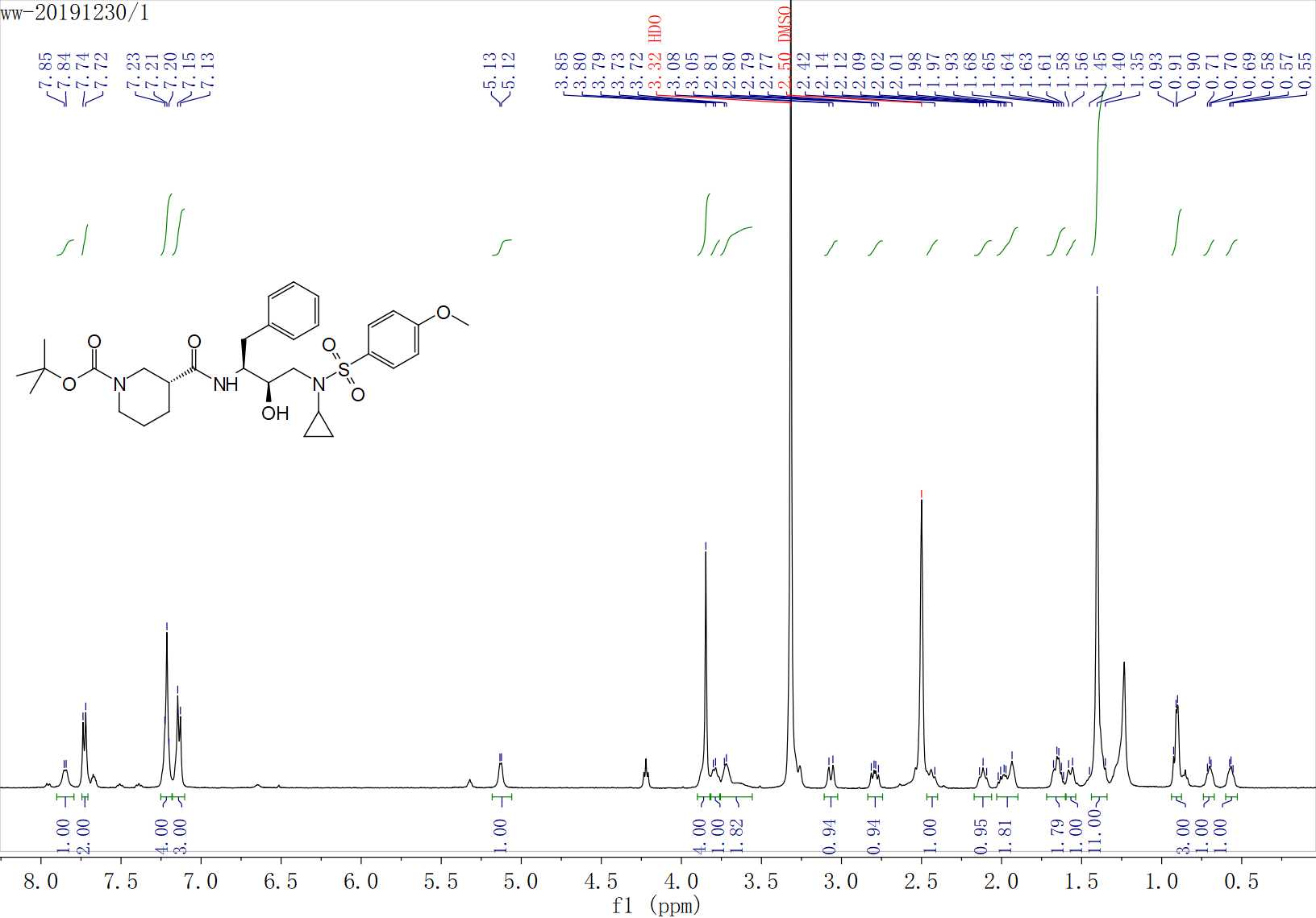
**

**Fig. S17.** ^1^H NMR Spectrum of compound **19a**

**
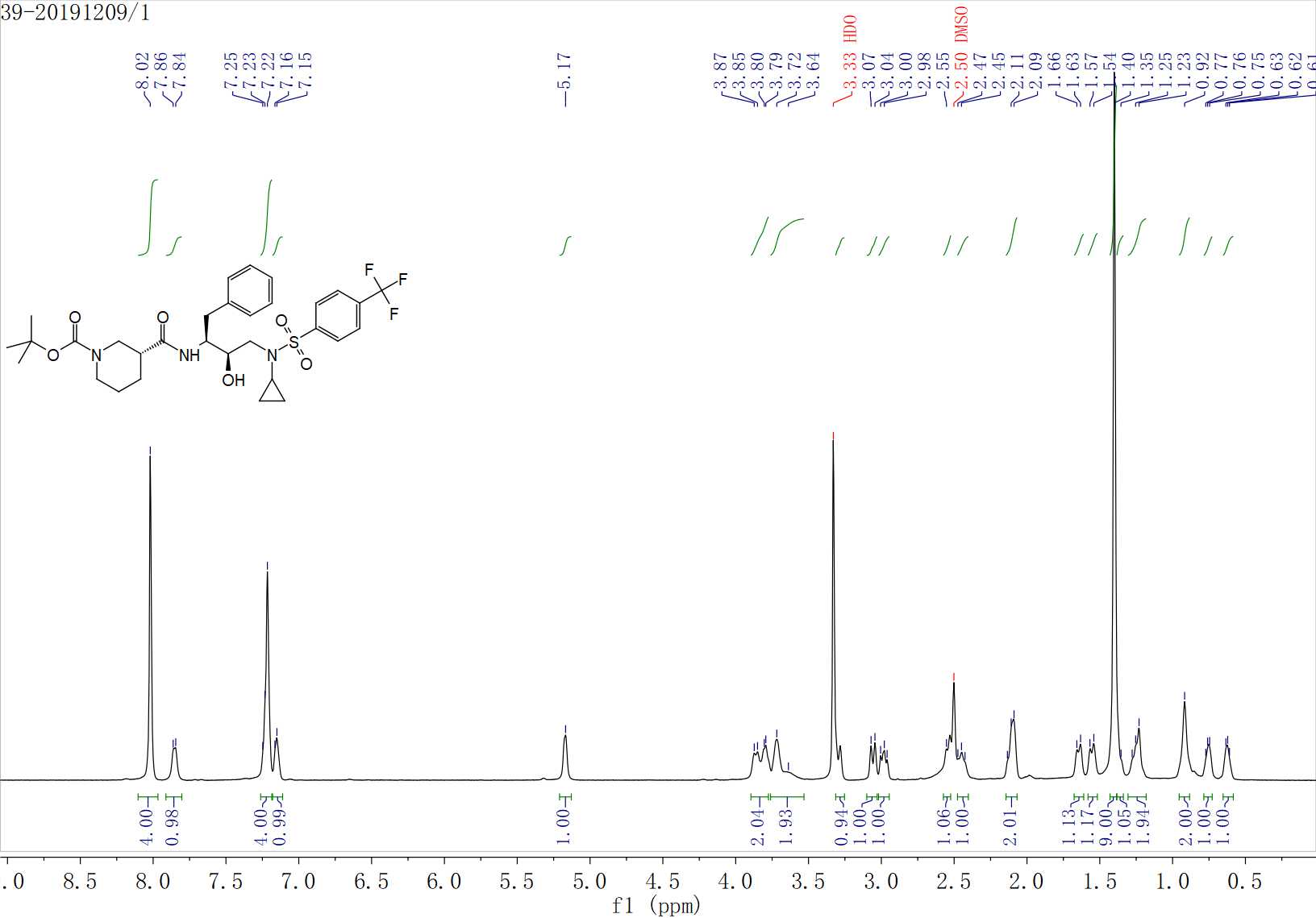
**

**Fig. S18.** ^1^H NMR Spectrum of compound **19b**

**
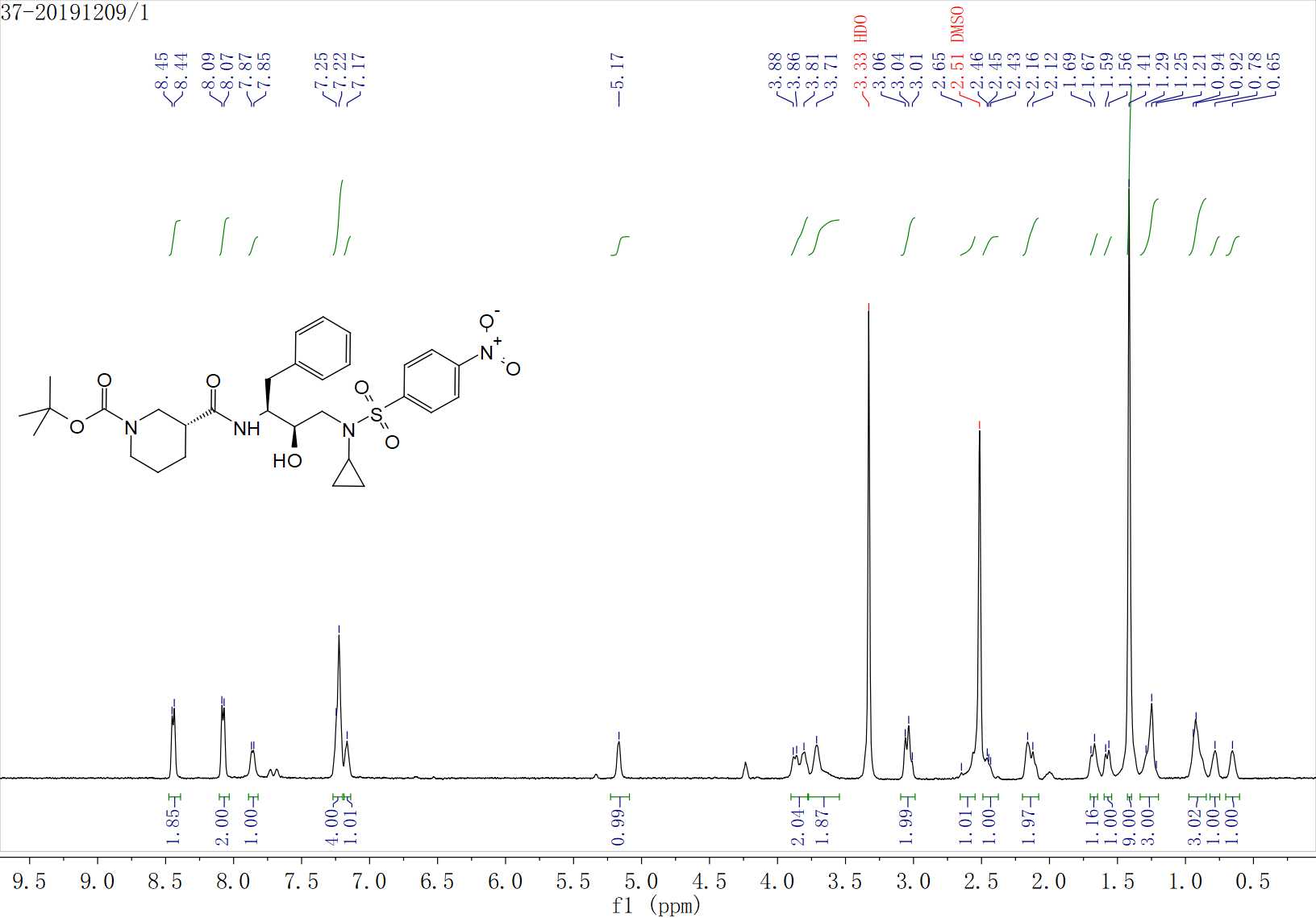
**

**Fig. S19.** ^1^H NMR Spectrum of compound **19c**

**
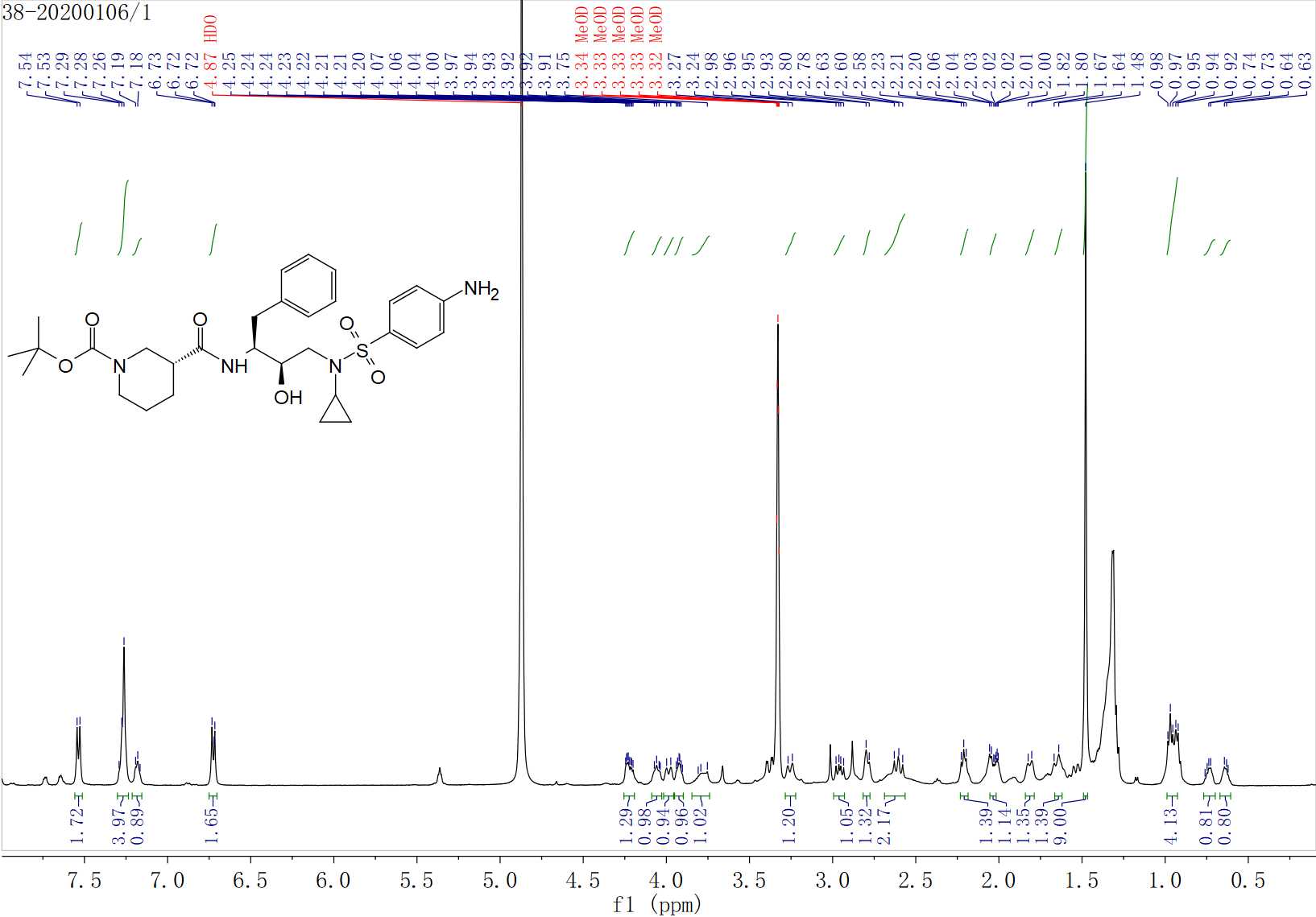
**

**Fig. S20.** ^1^H NMR Spectrum of compound **19d**

**
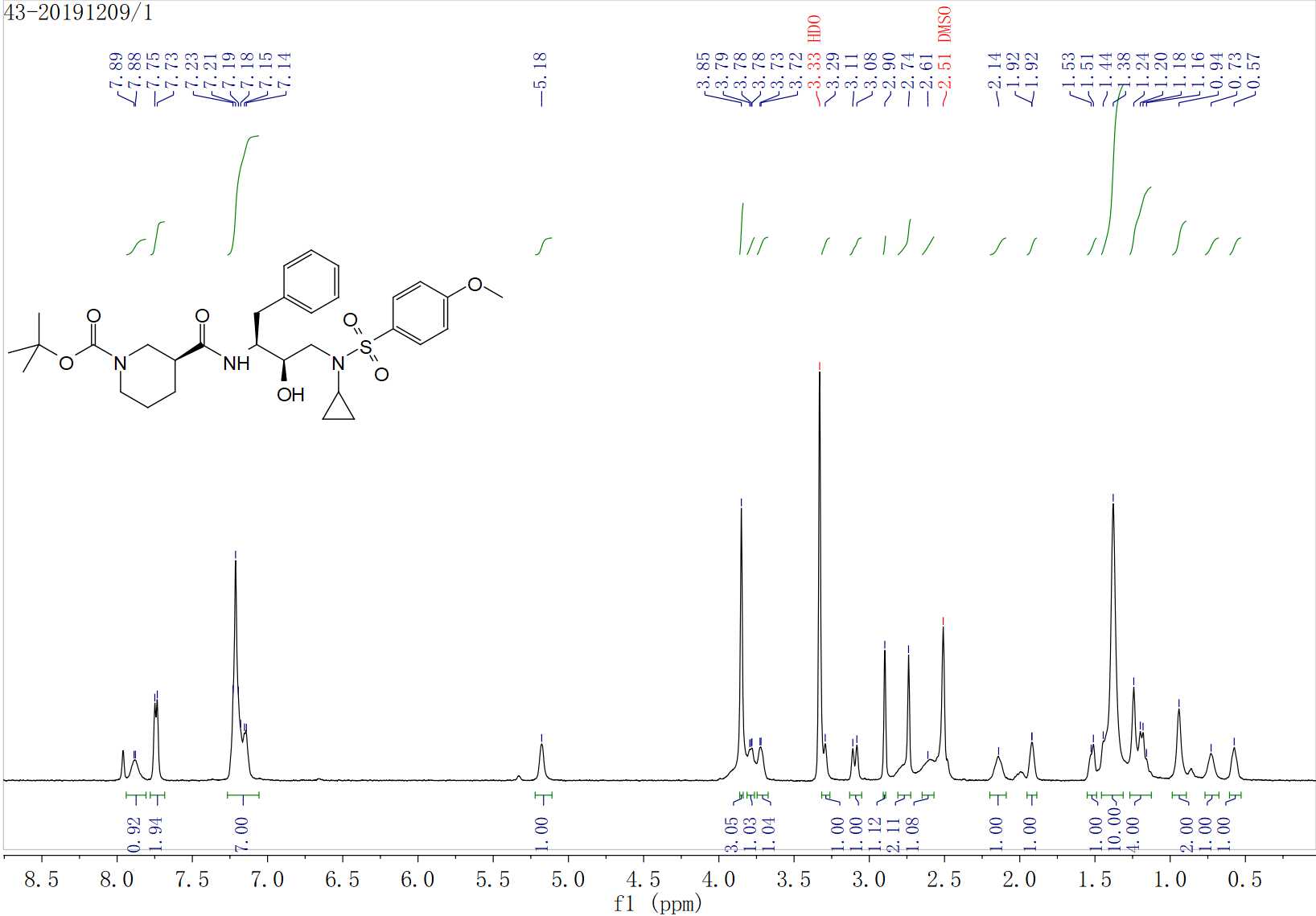
**

**Fig. S21.** ^1^H NMR Spectrum of compound **20a**


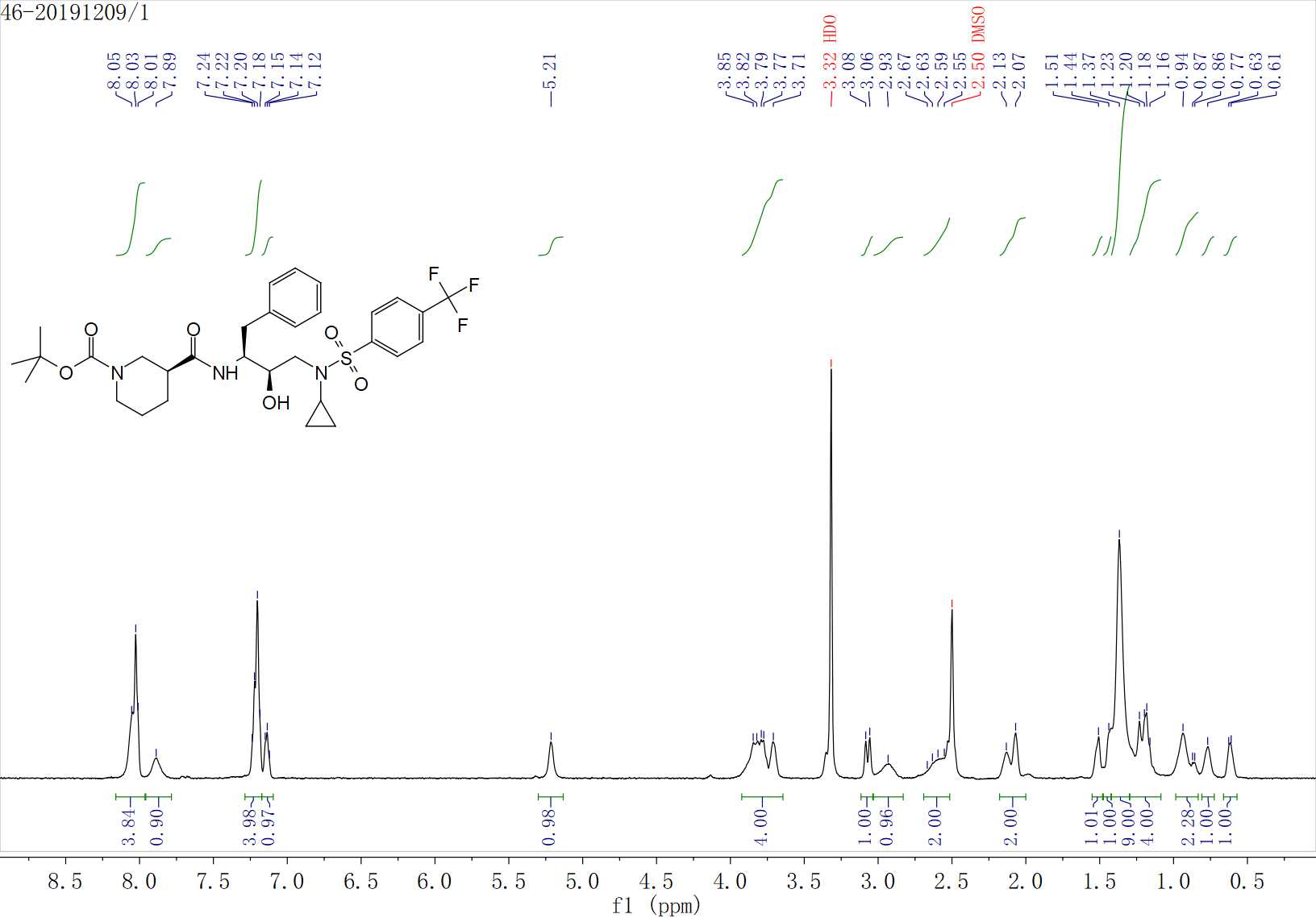


**Fig. S22.** ^1^H NMR Spectrum of compound **20b**

**
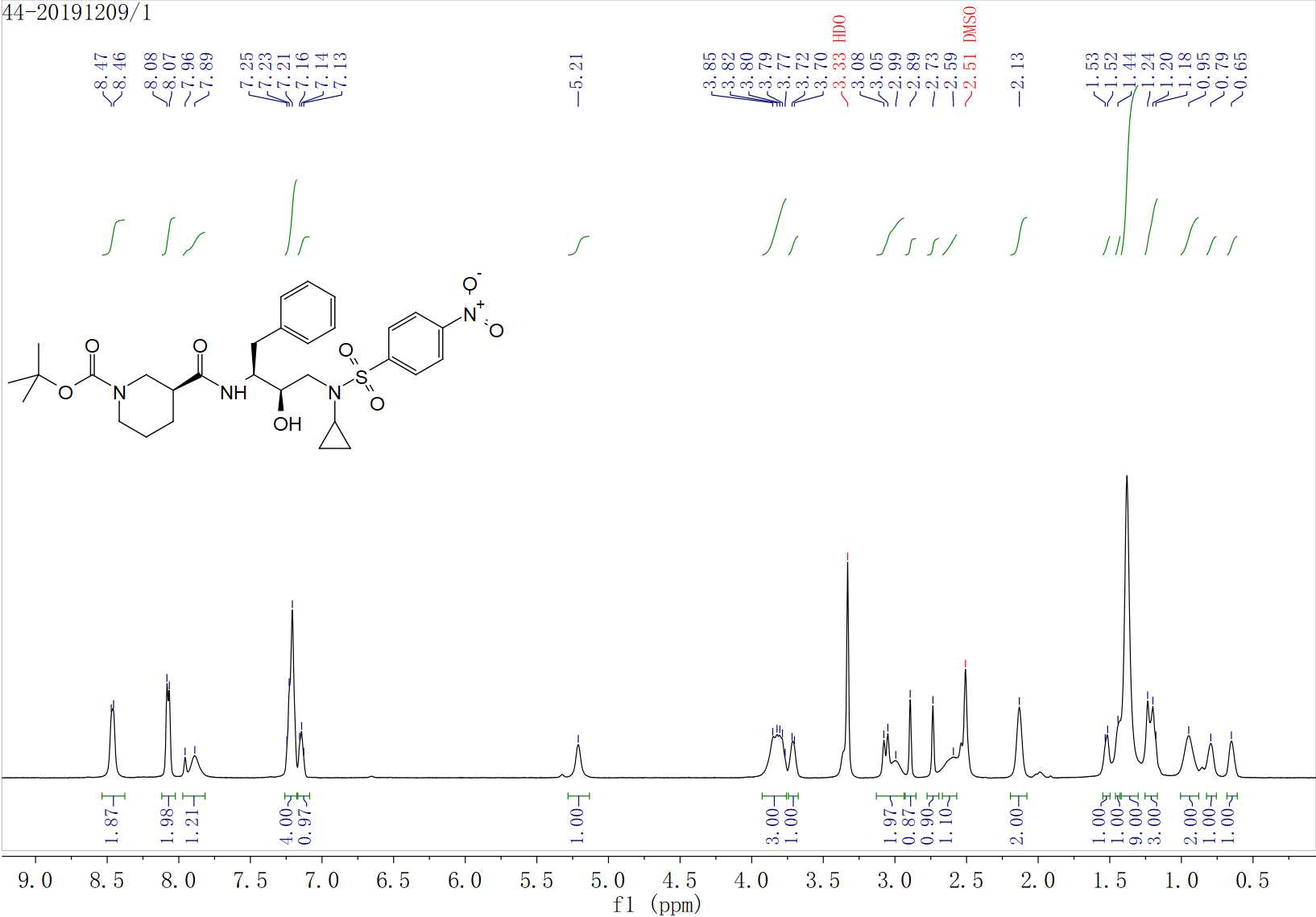
**

**Fig. S23.** ^1^H NMR Spectrum of compound **20c**


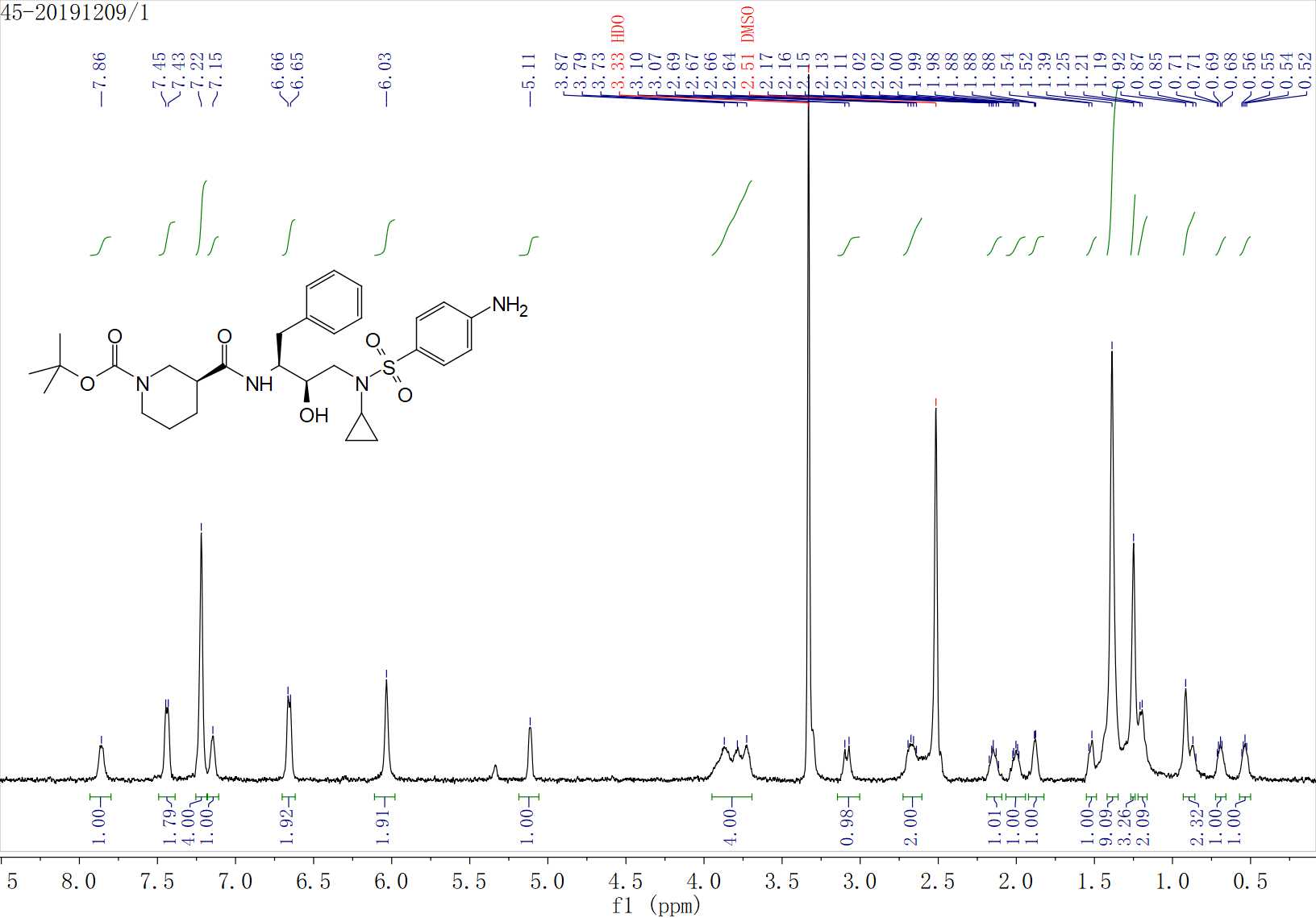


**Fig. S24.** ^1^H NMR Spectrum of compound **20d**

**
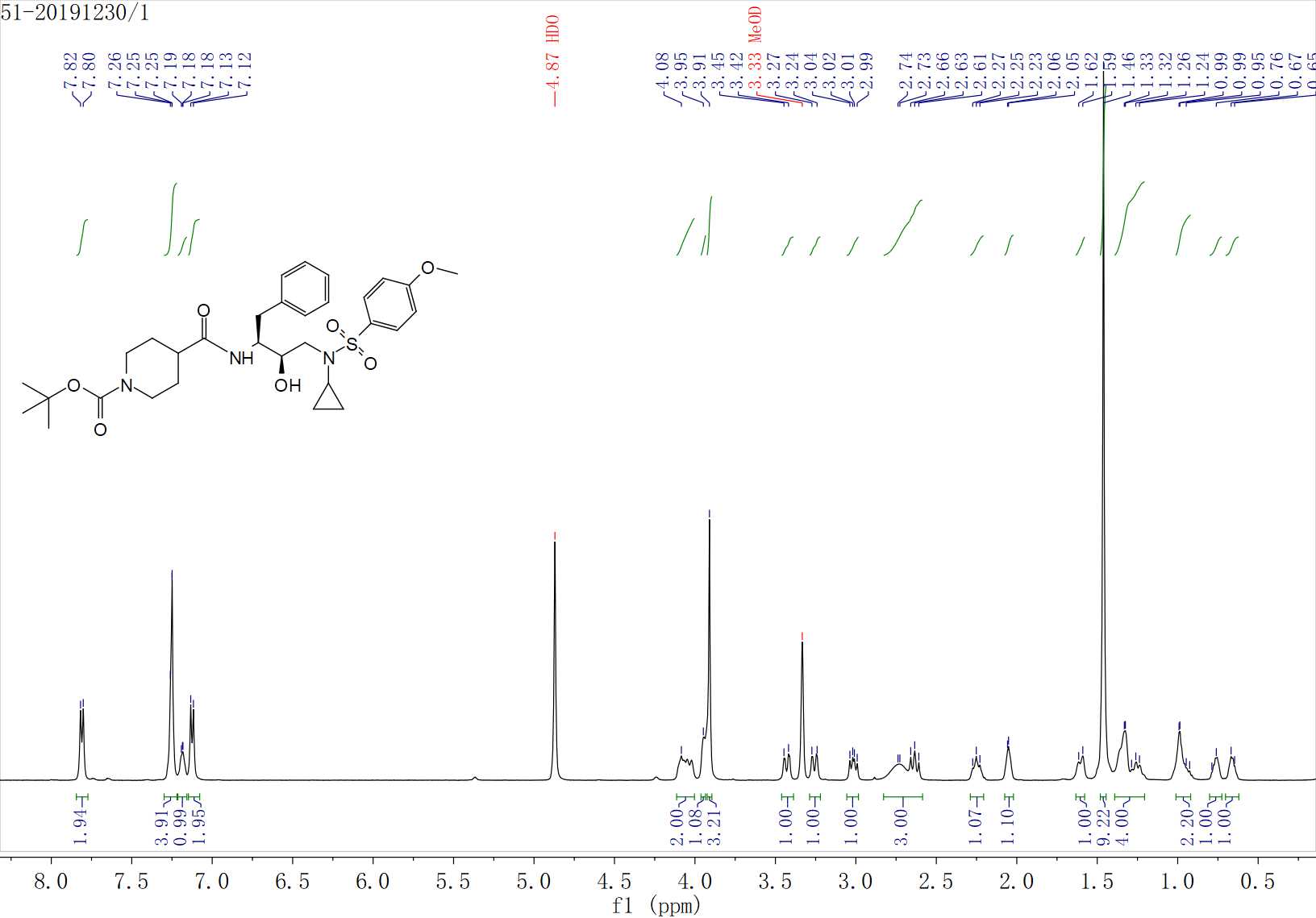
**

**Fig. S25.** ^1^H NMR Spectrum of compound **21a**

**
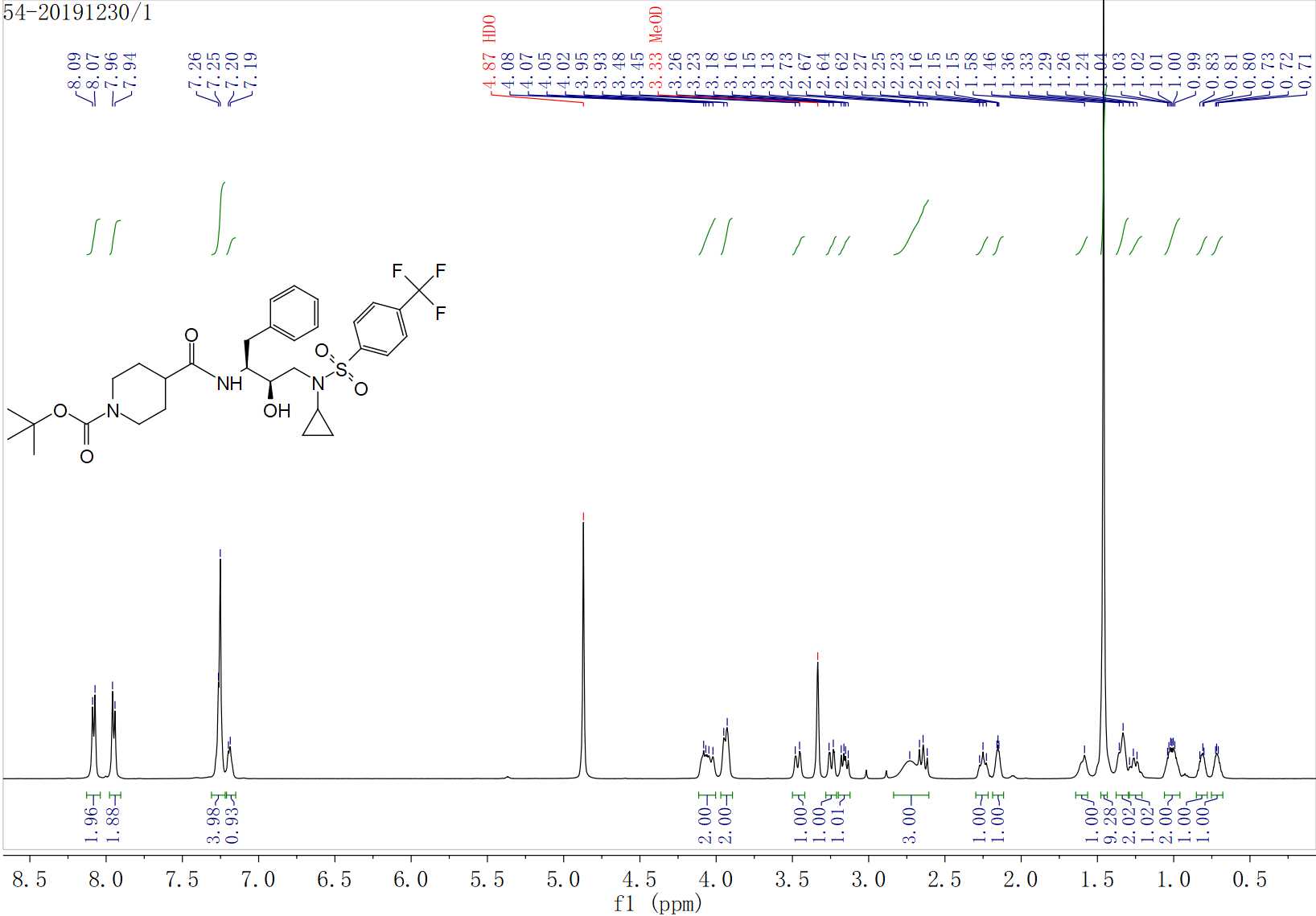
**

**Fig. S26.** ^1^H NMR Spectrum of compound **21b**

**
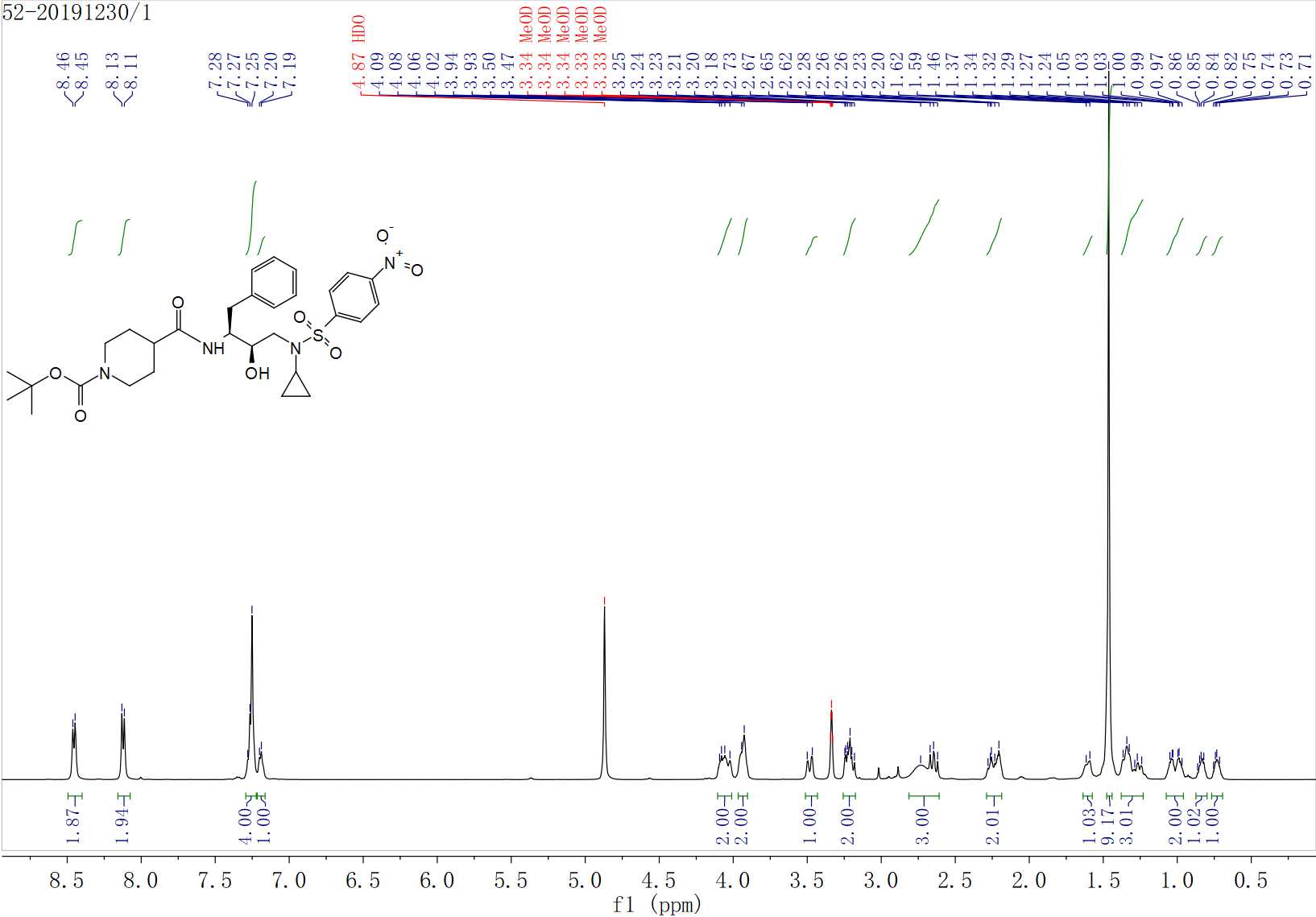
**

**Fig. S27.** ^1^H NMR Spectrum of compound **21c**

**
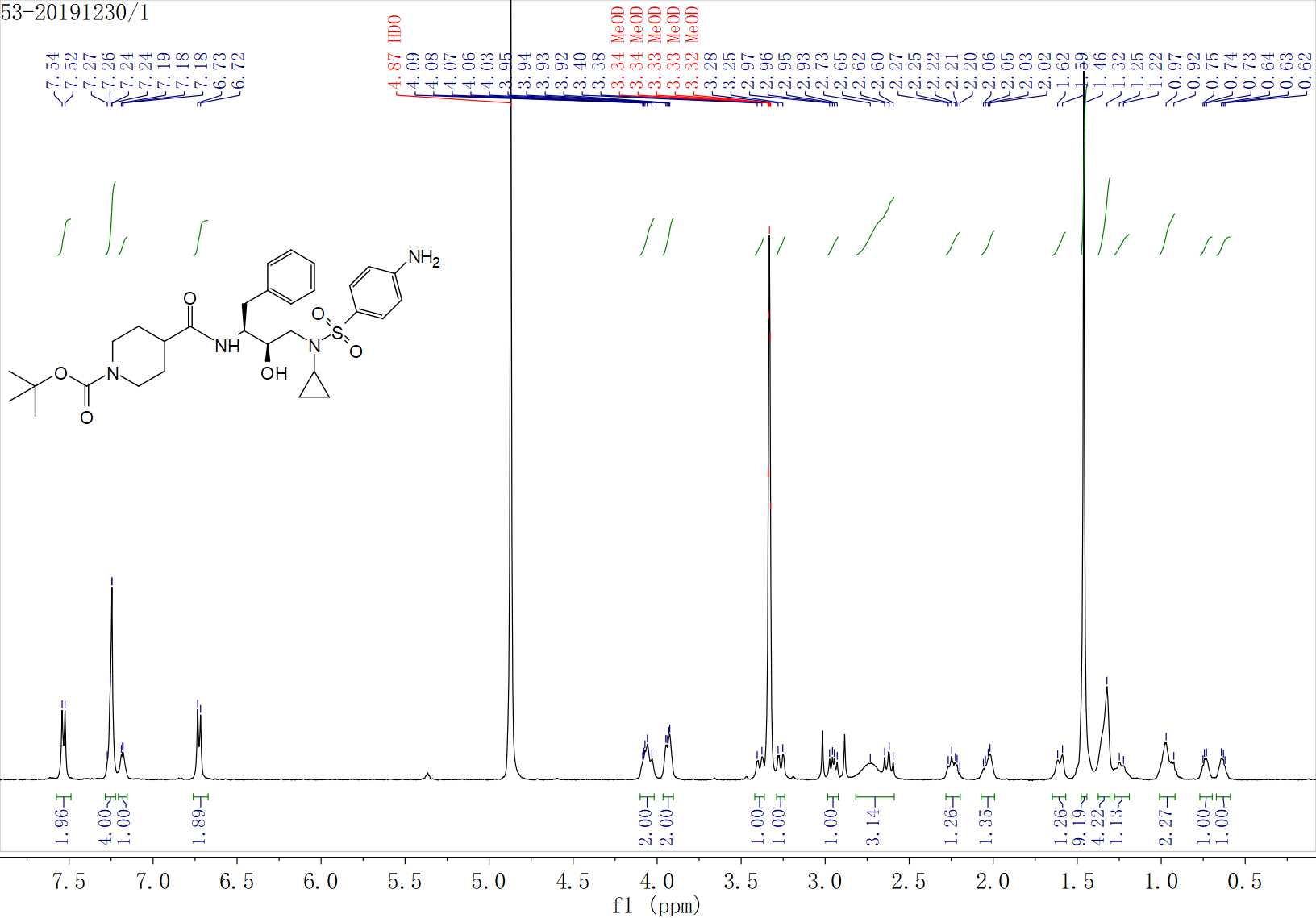
**

**Fig. S28.** ^1^H NMR Spectrum of compound **21d**


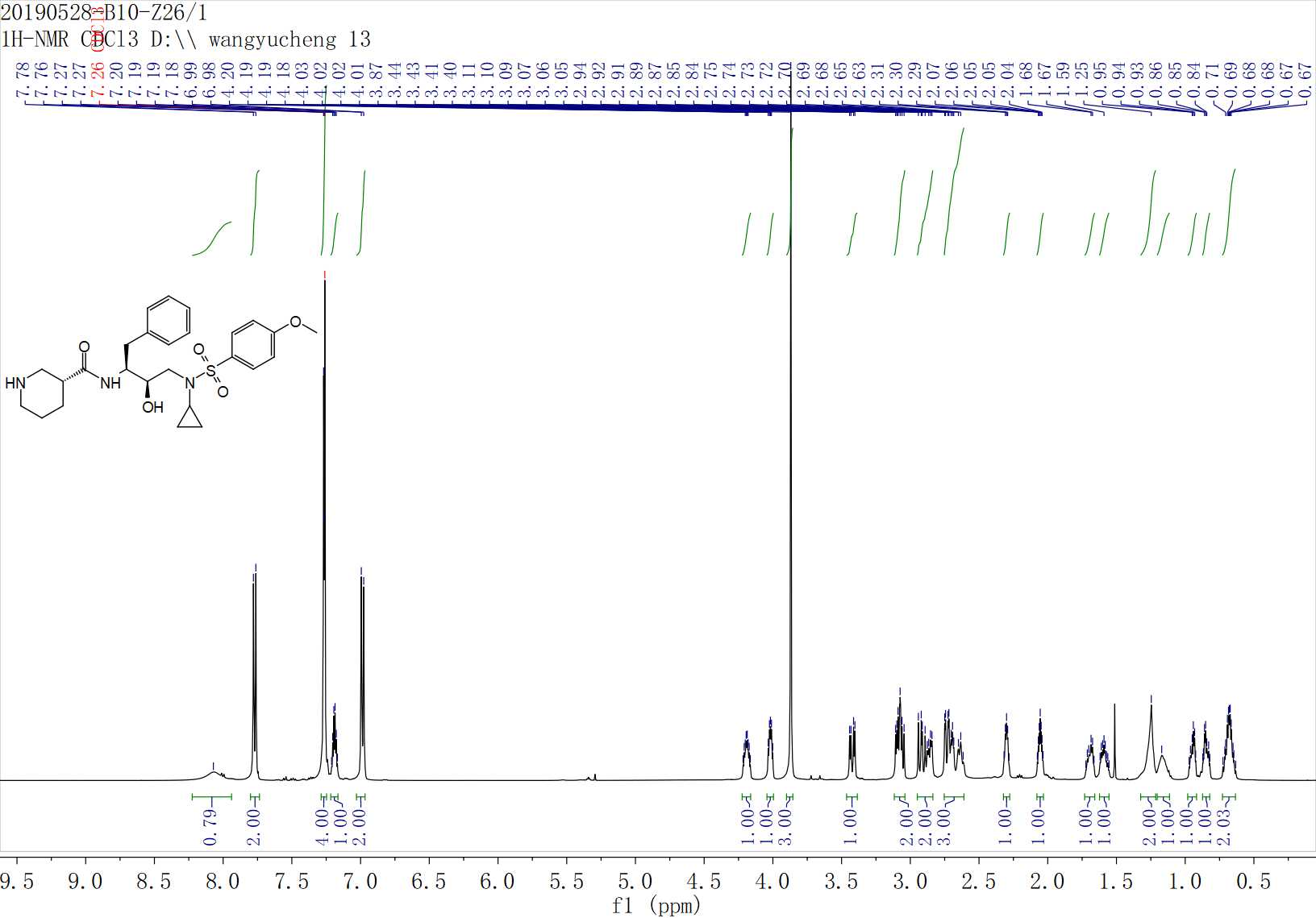


**Fig. S29.** ^1^H NMR Spectrum of compound **22a**


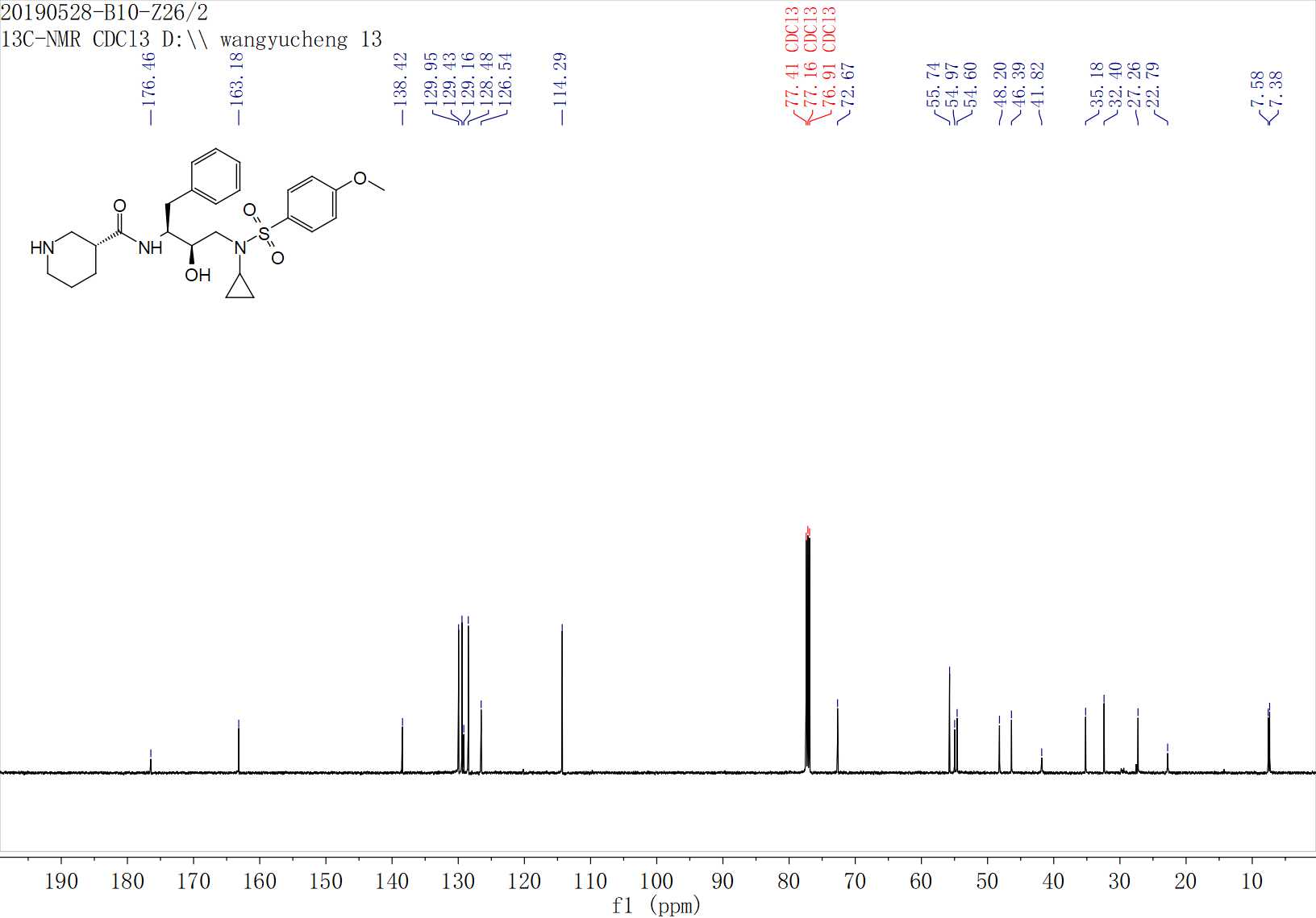


**Fig. S30.** ^13^C NMR Spectrum of compound **22a**

**
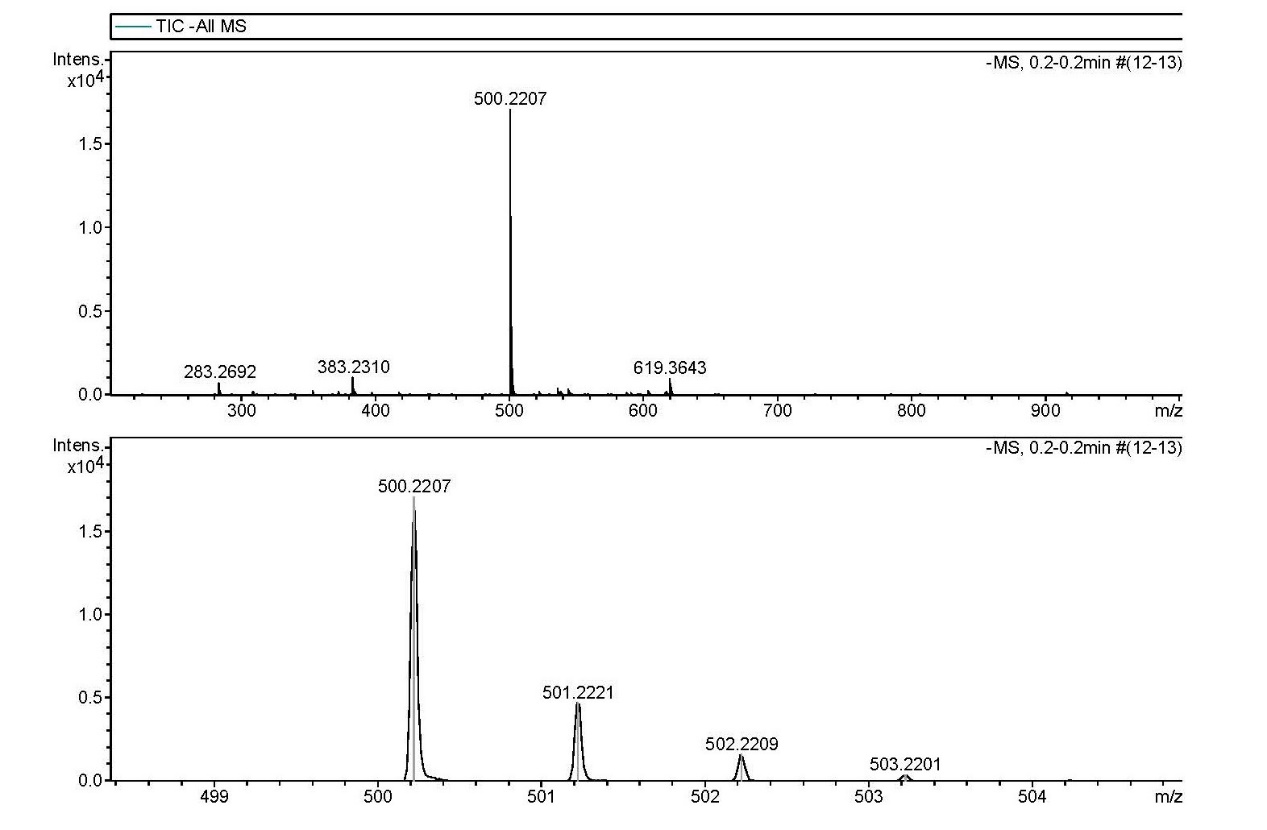
**

**Fig. S31.** HR MS Spectrum of compound **22a**


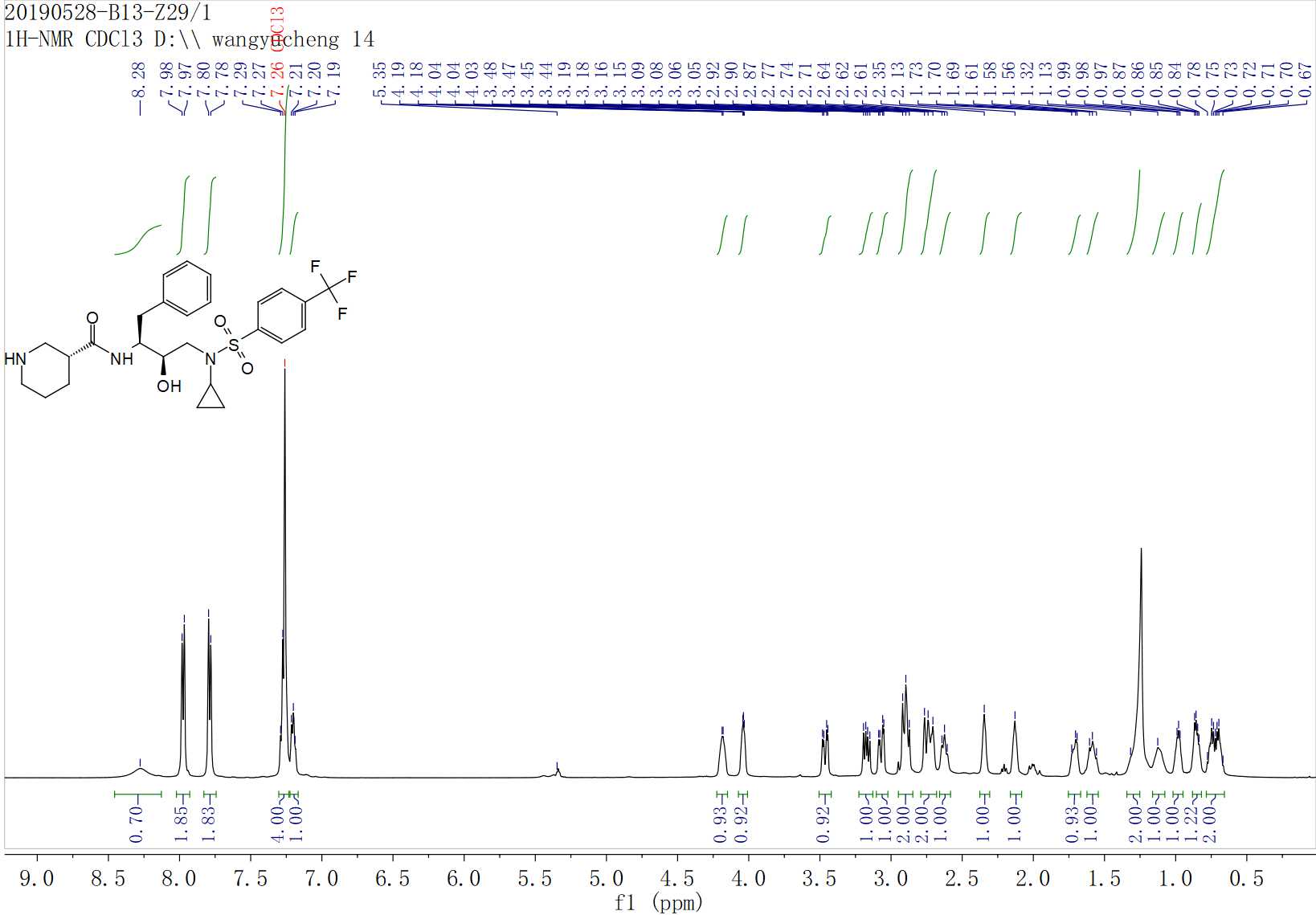


**Fig. S32.** ^1^H NMR Spectrum of compound **22b**


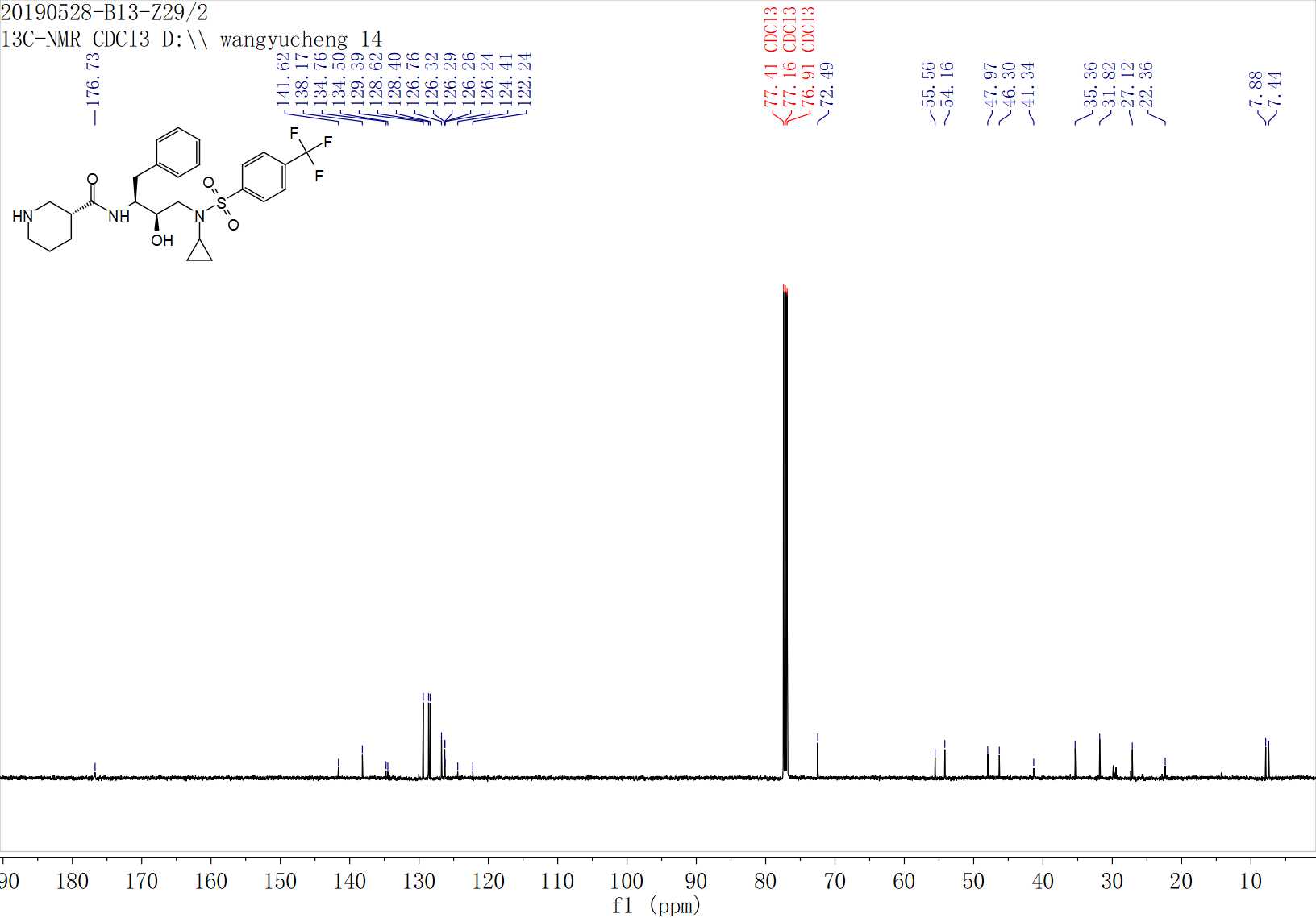


**Fig. S33.** ^13^C NMR Spectrum of compound **22b**

**
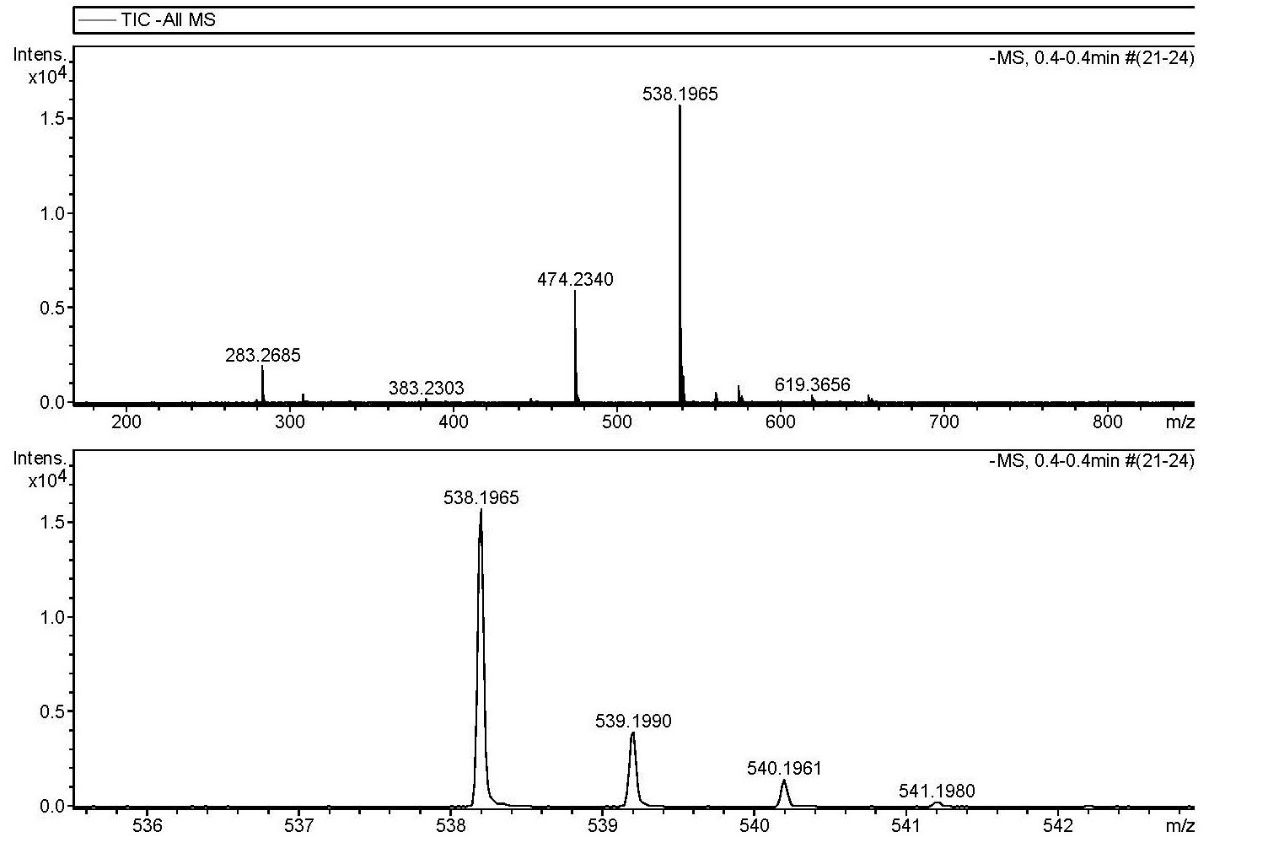
**

**Fig. S34.** HR MS Spectrum of compound **22b**


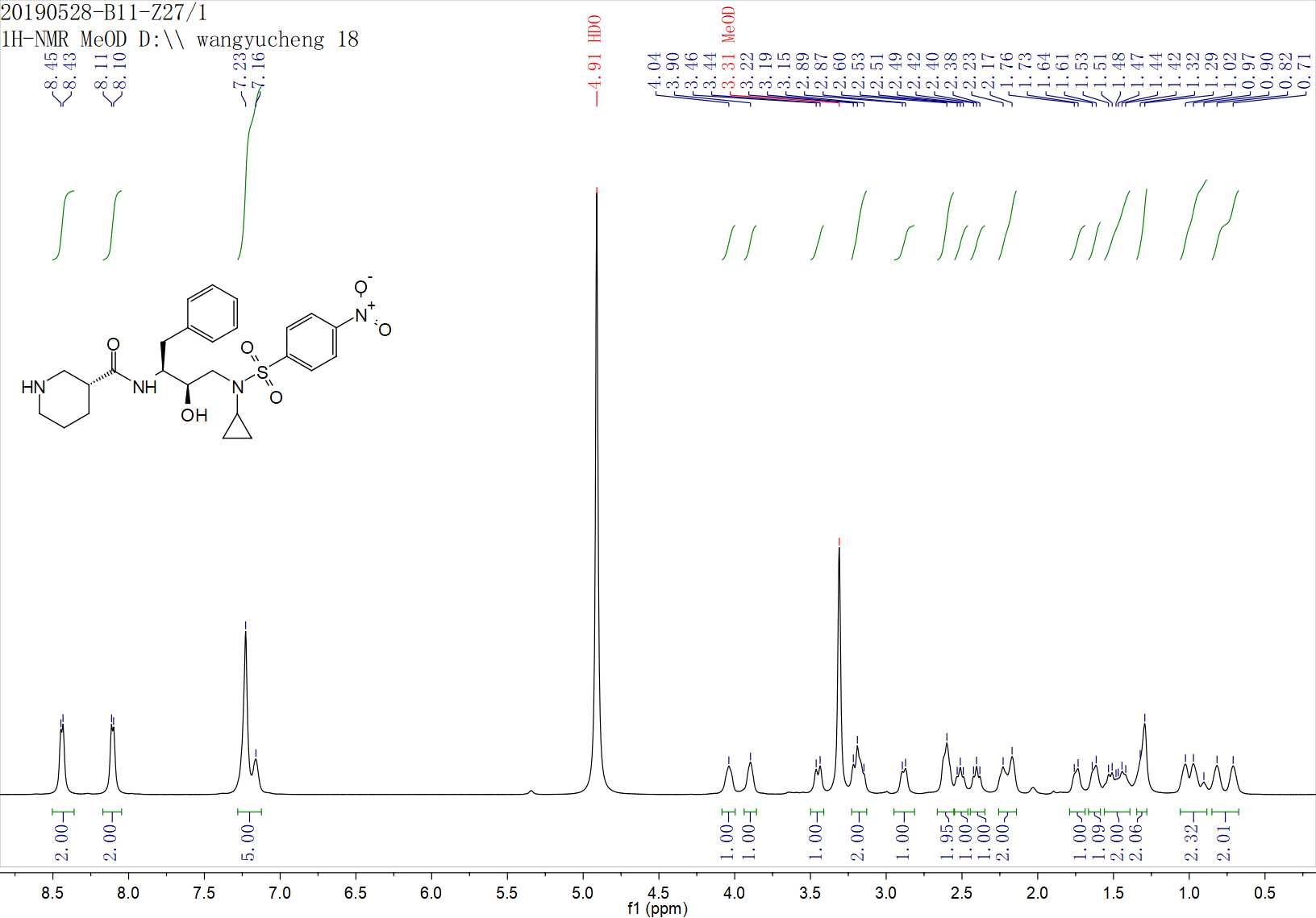


**Fig. S35.** ^1^H NMR Spectrum of compound **22c**


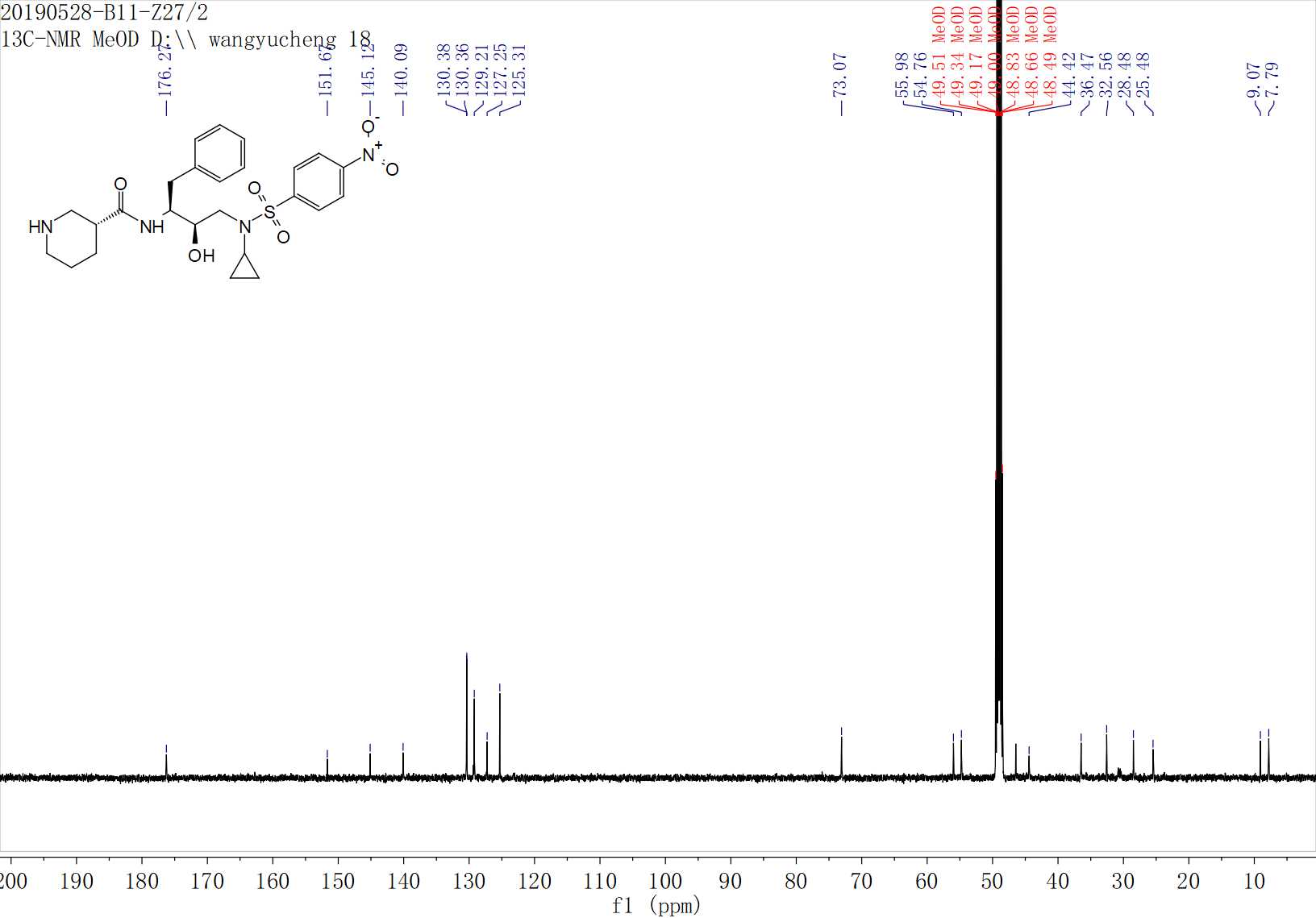


**Fig. S36.** ^13^C NMR Spectrum of compound **22c**

**
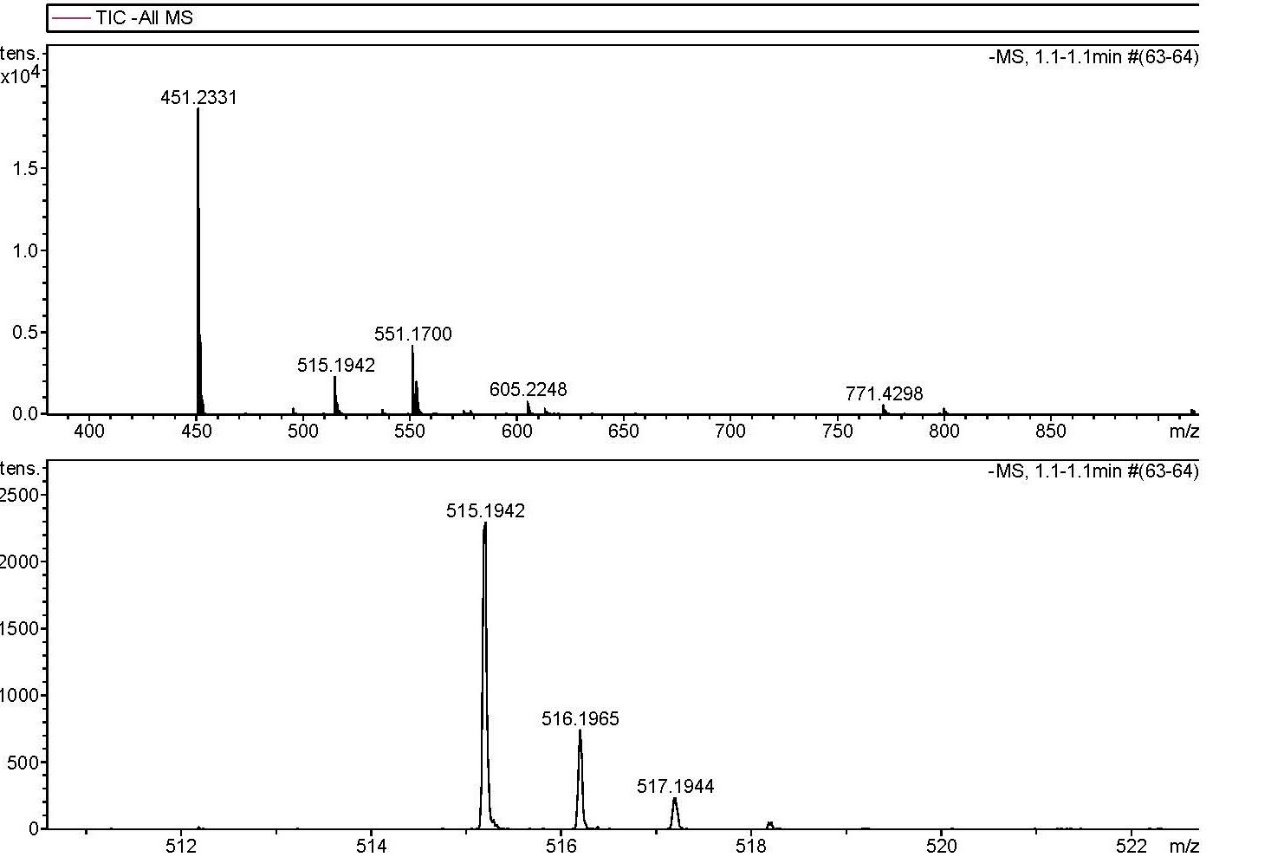
**

**Fig. S37.** HR MS Spectrum of compound **22c**


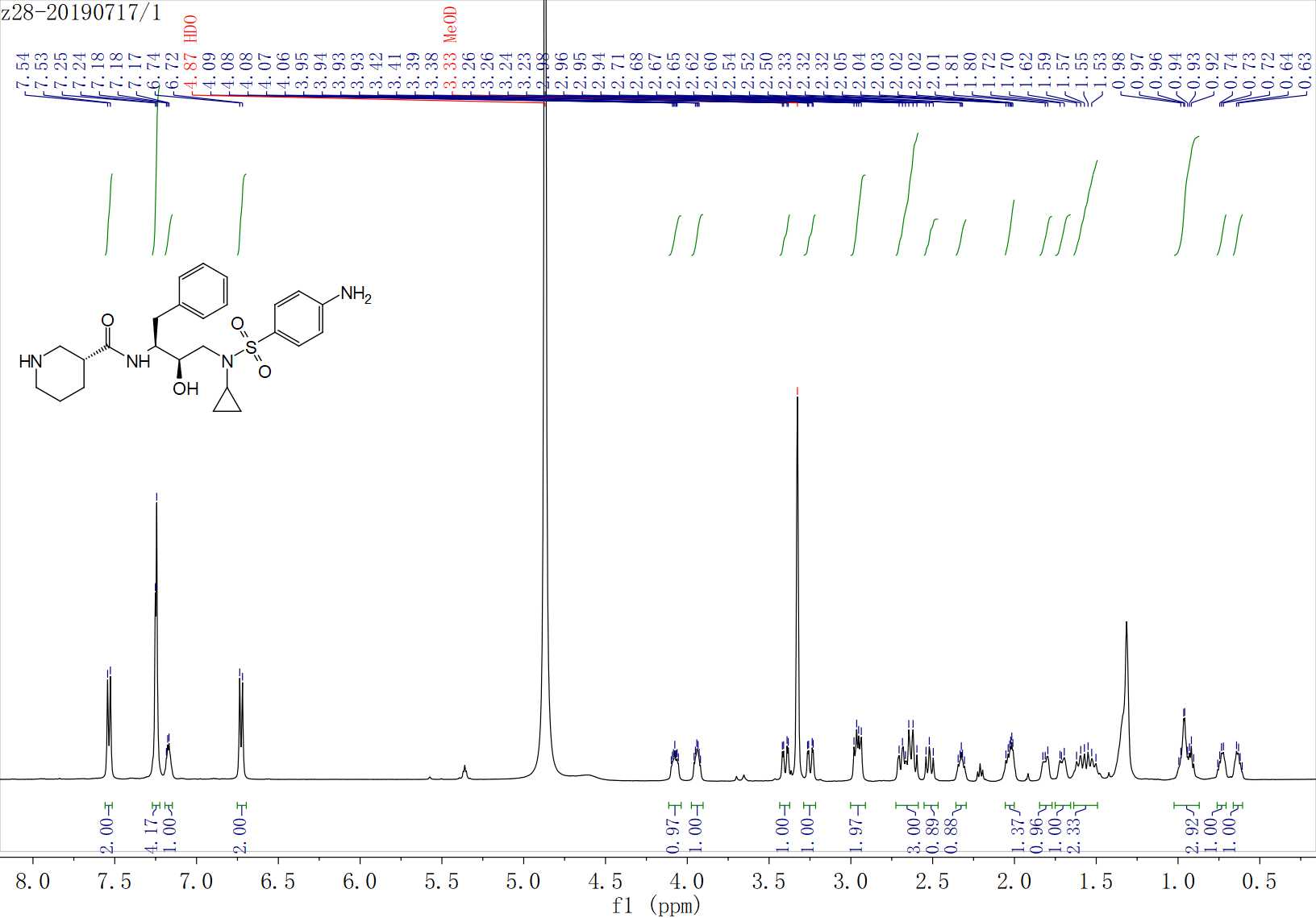


**Fig. S38.** ^1^H NMR Spectrum of compound **22d**


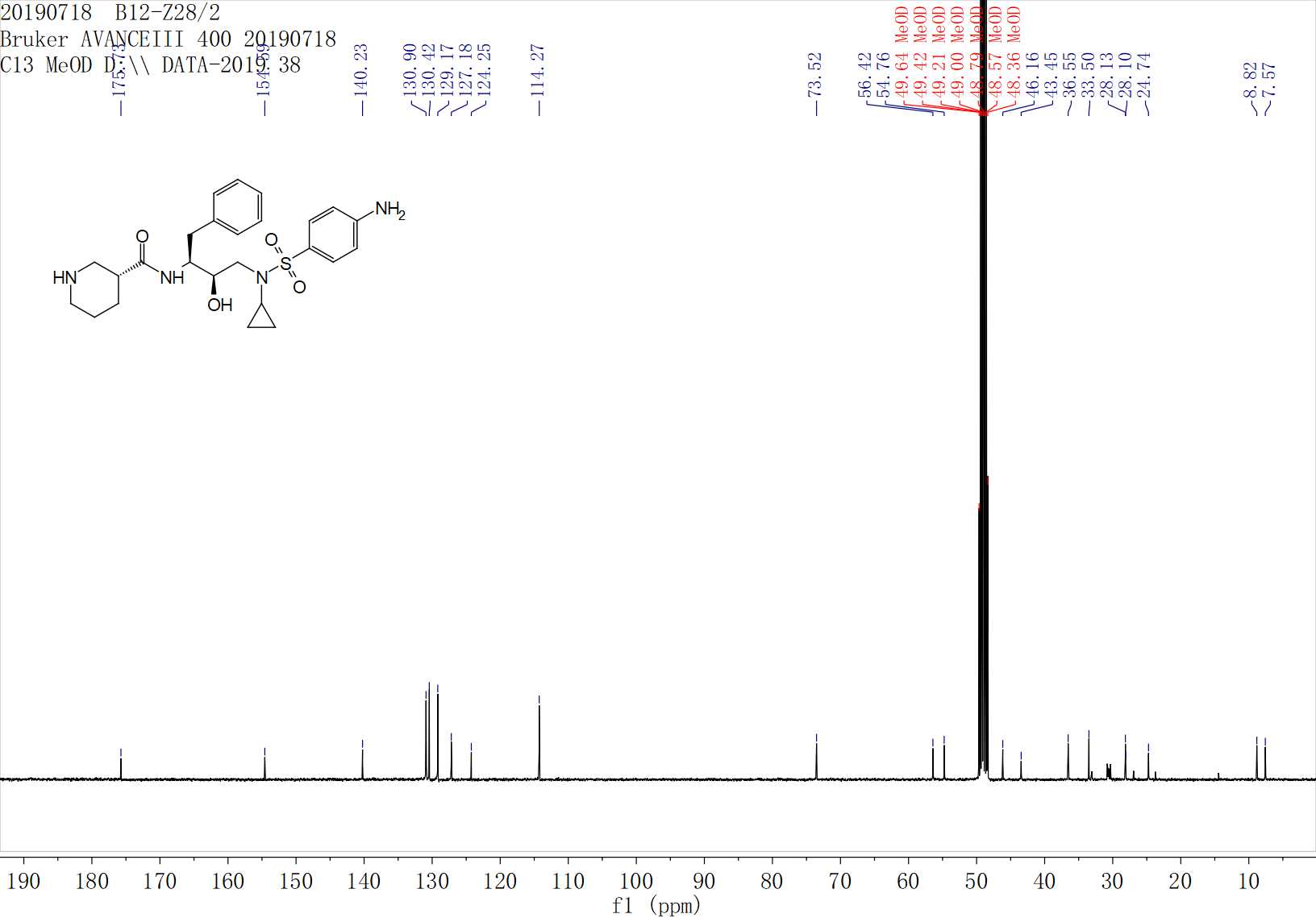


**Fig. S39.** ^13^C NMR Spectrum of compound **22d**

**
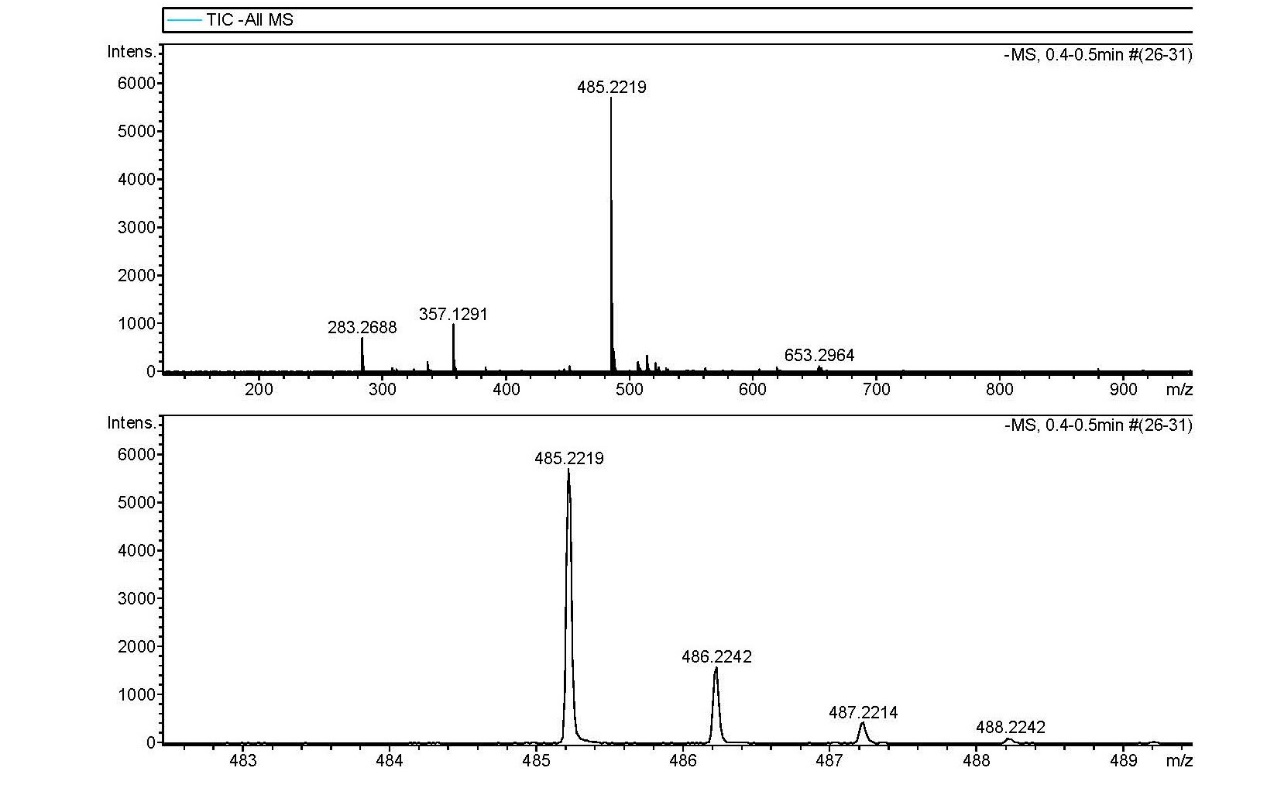
**

**Fig. S40.** HR MS Spectrum of compound **22d**


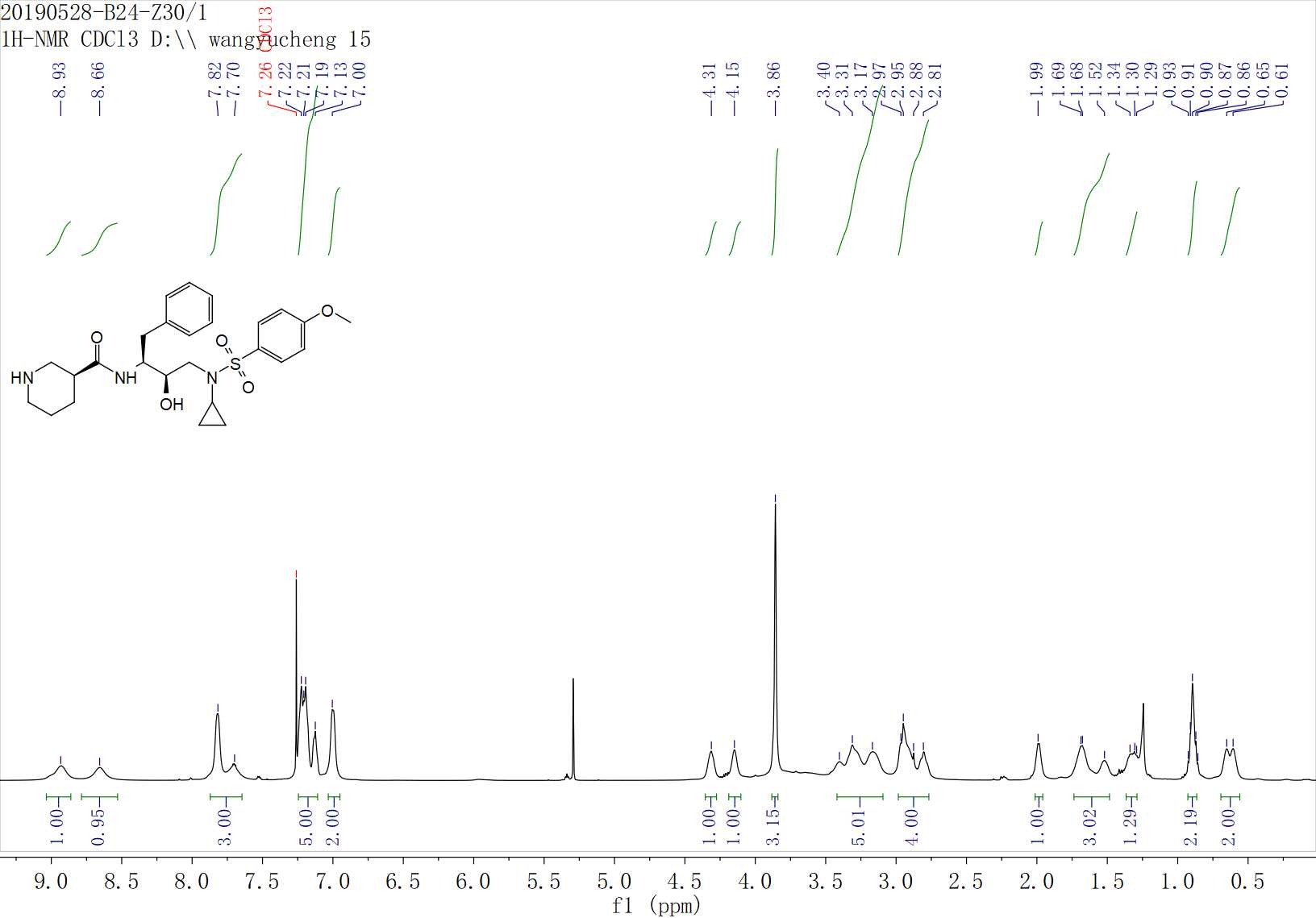


**Fig. S41.** ^1^H NMR Spectrum of compound **23a**


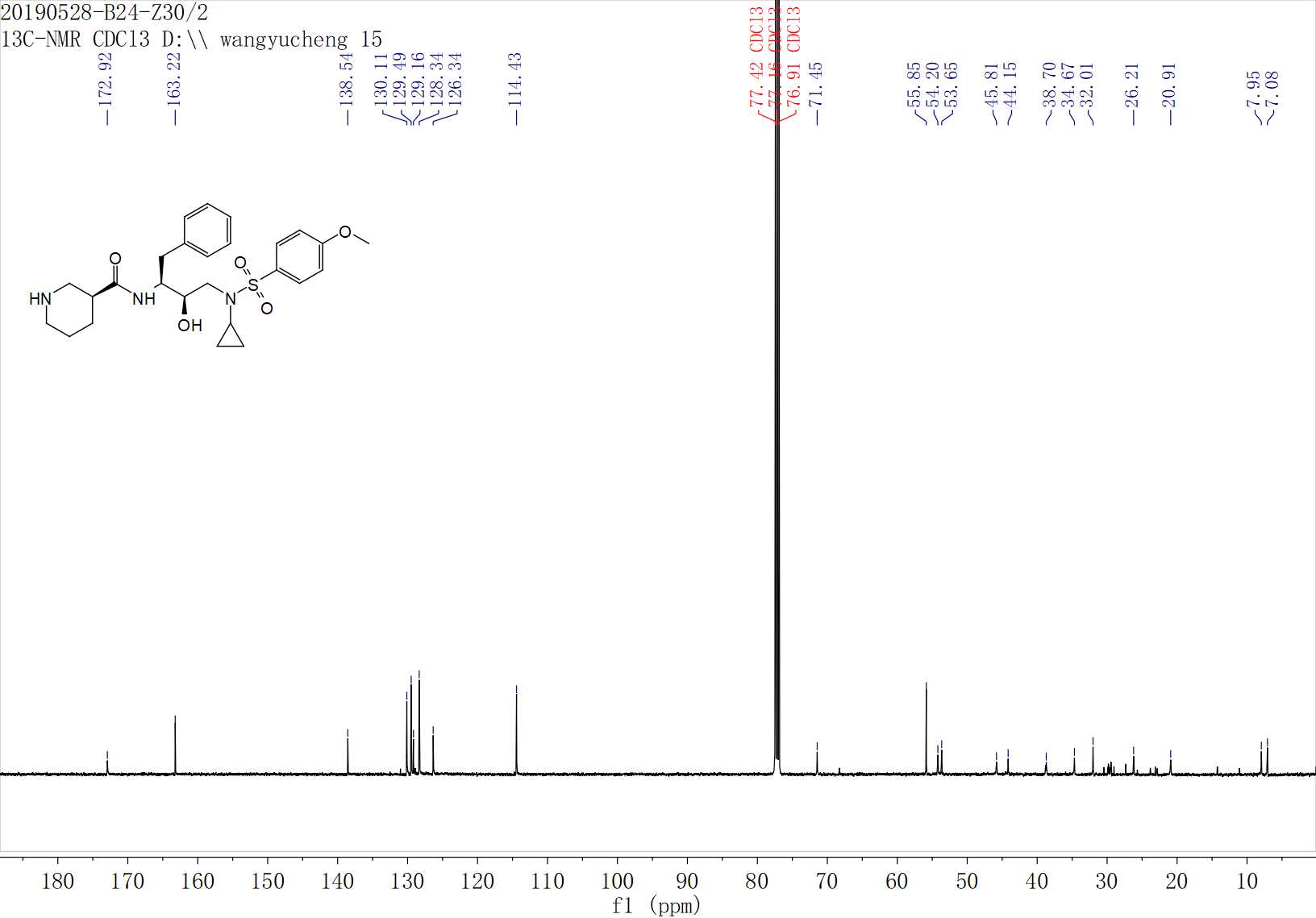


**Fig. S42.** ^13^C NMR Spectrum of compound **23a**

**
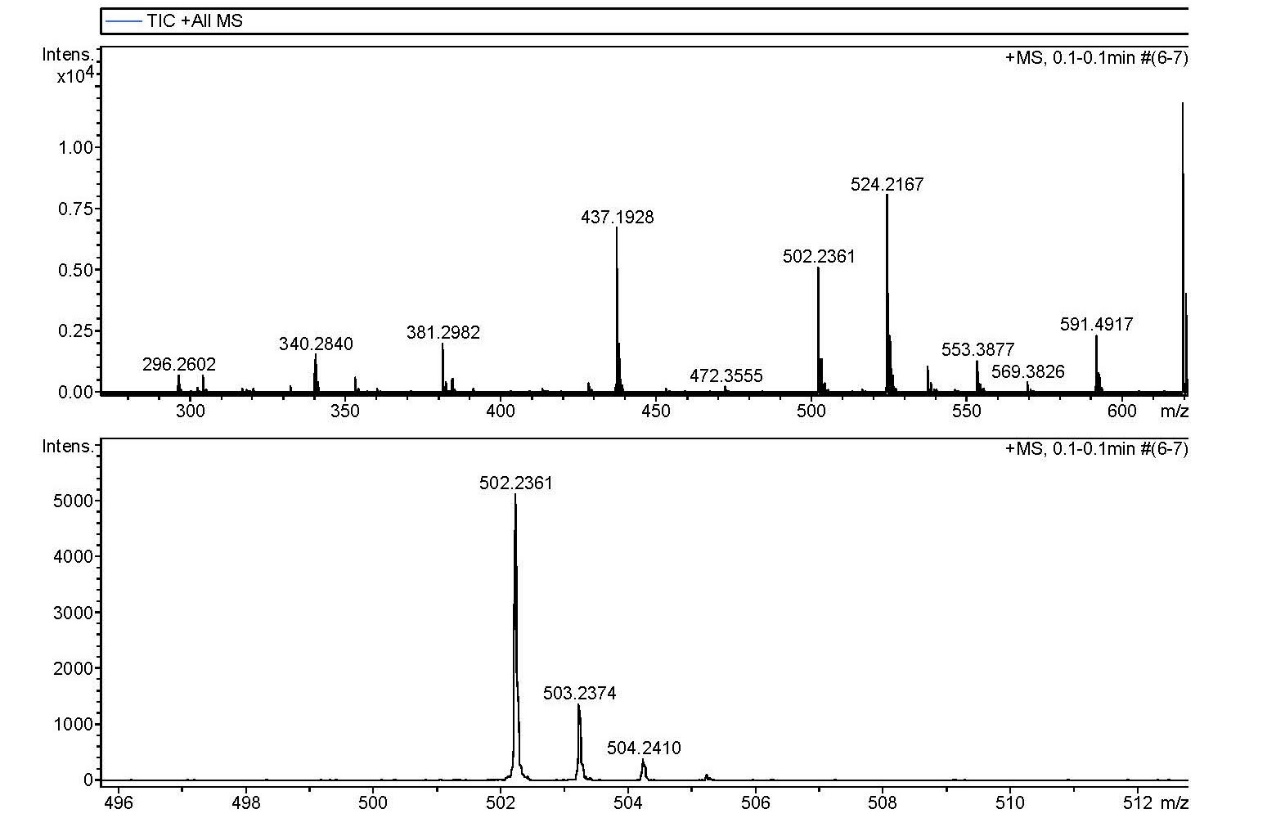
**

**Fig. S43.** HR MS Spectrum of compound **23a**


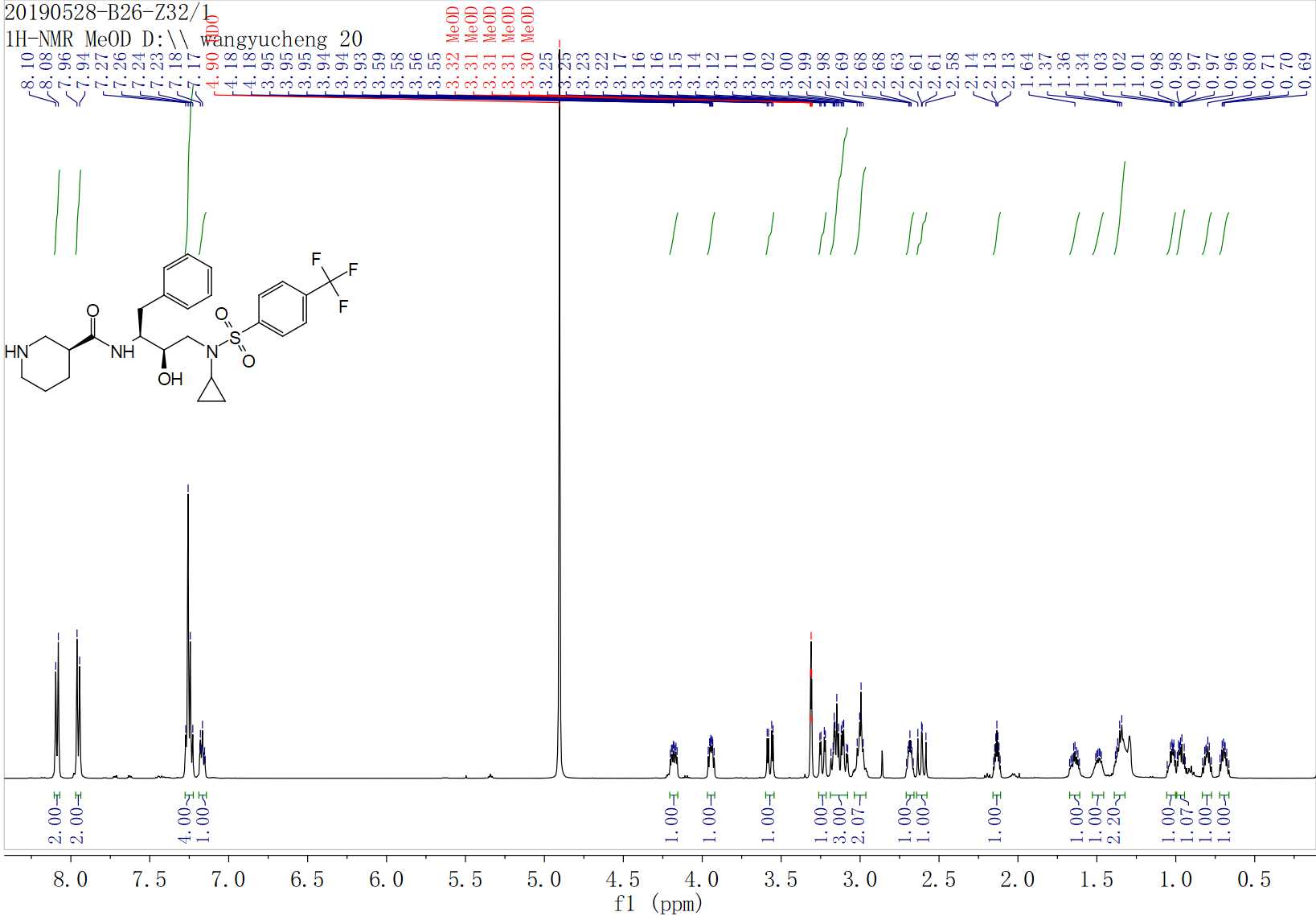


**Fig. S44.** ^1^H NMR Spectrum of compound **23b**


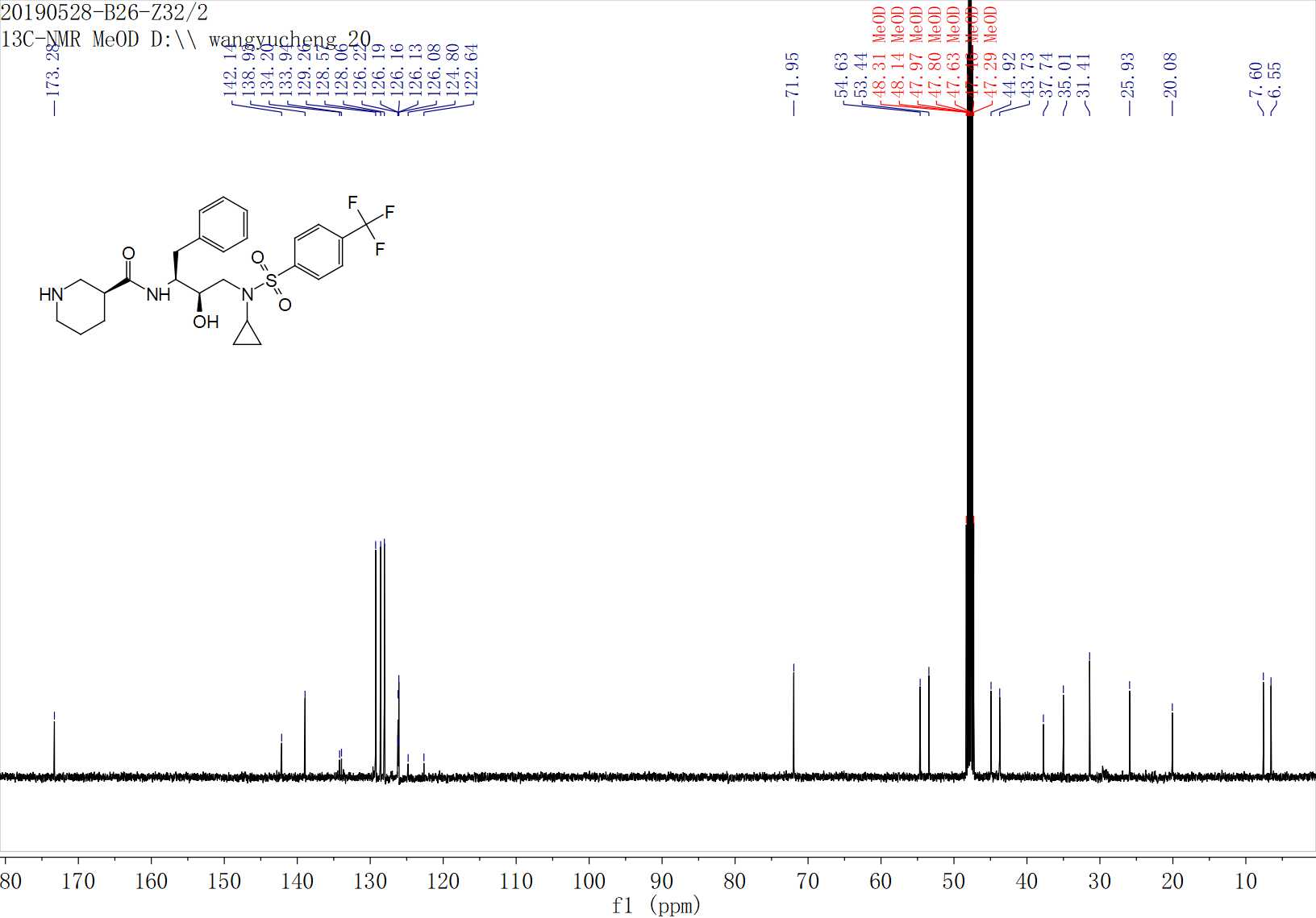


**Fig. S45.** ^13^C NMR Spectrum of compound **23b**

**
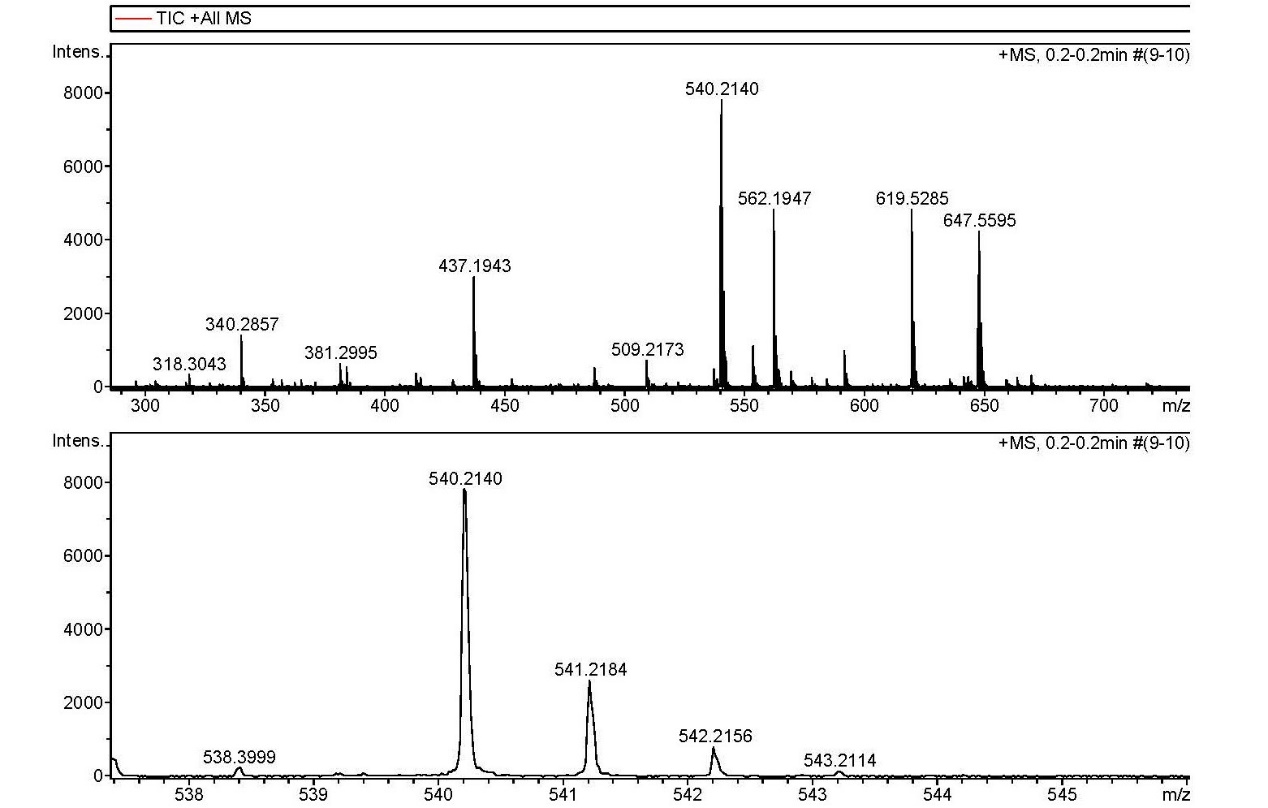
**

**Fig. S46.** HR MS Spectrum of compound **23b**


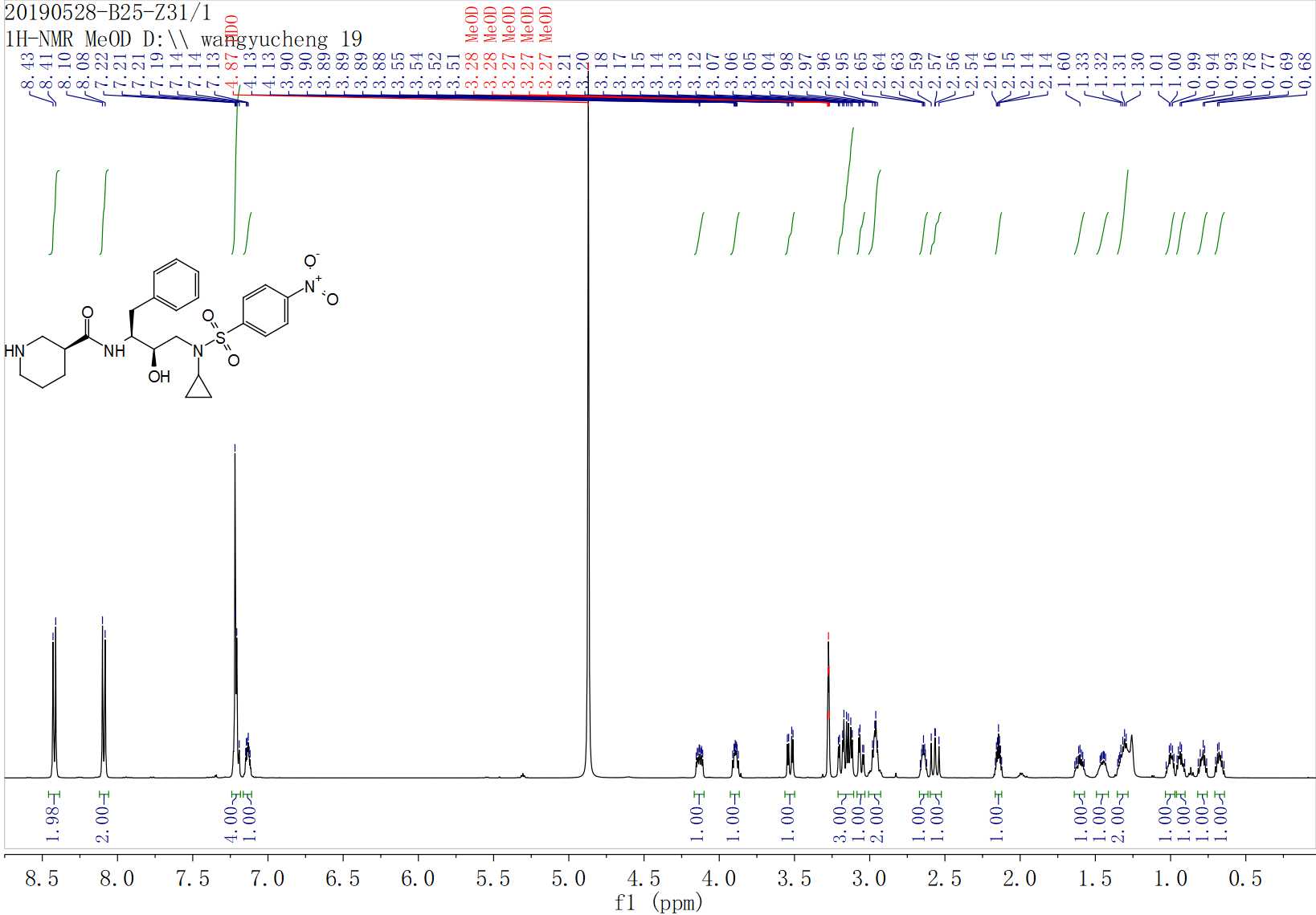


**Fig. S47.** ^1^H NMR Spectrum of compound **23c**


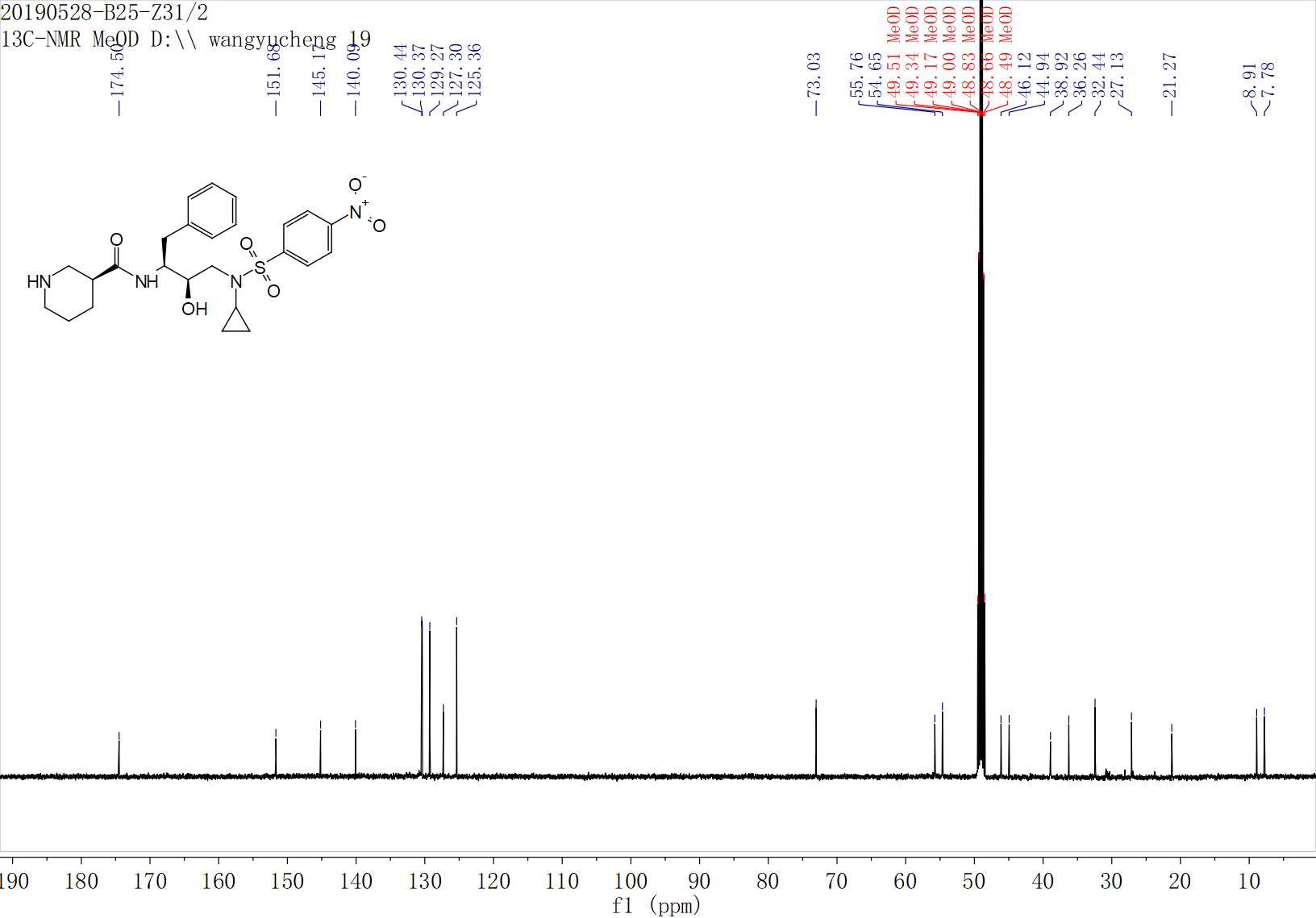


**Fig. S48.** ^13^C NMR Spectrum of compound **23c**

**
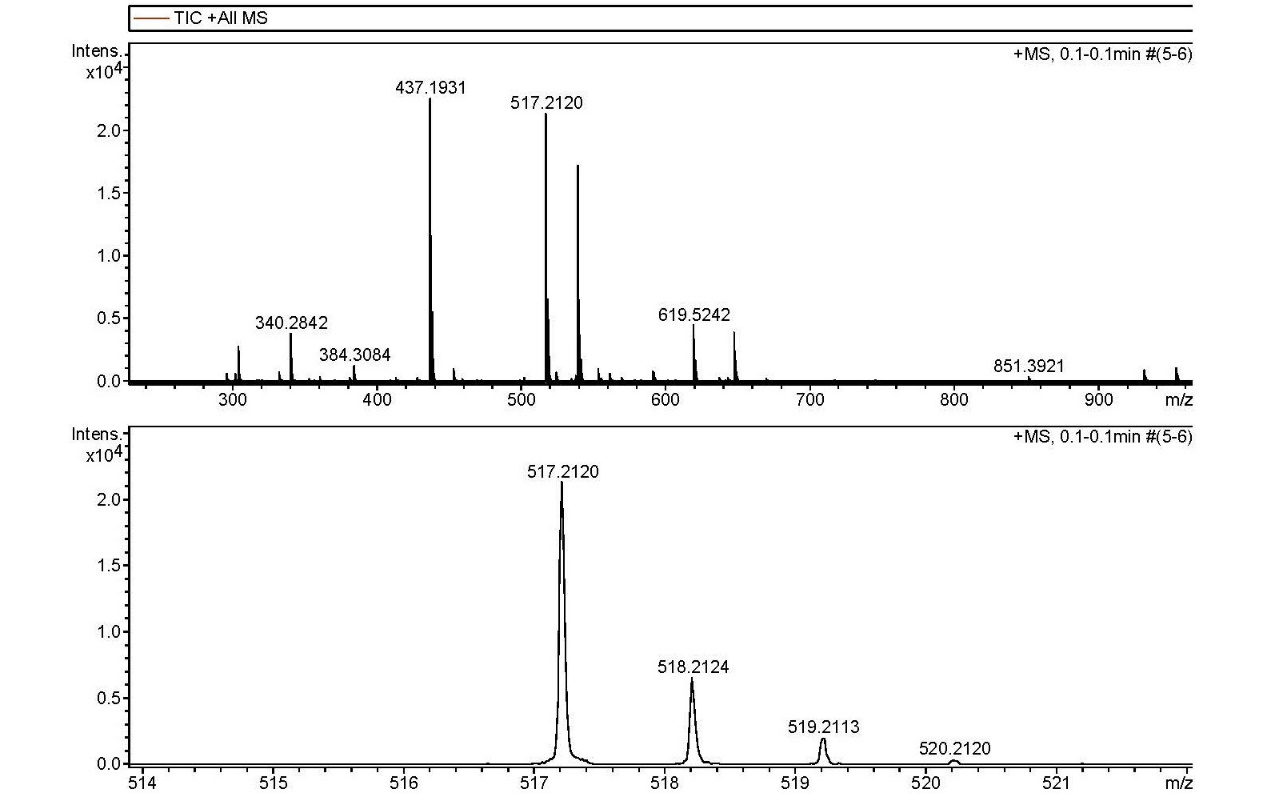
**

**Fig. S49.** HR MS Spectrum of compound **23c**


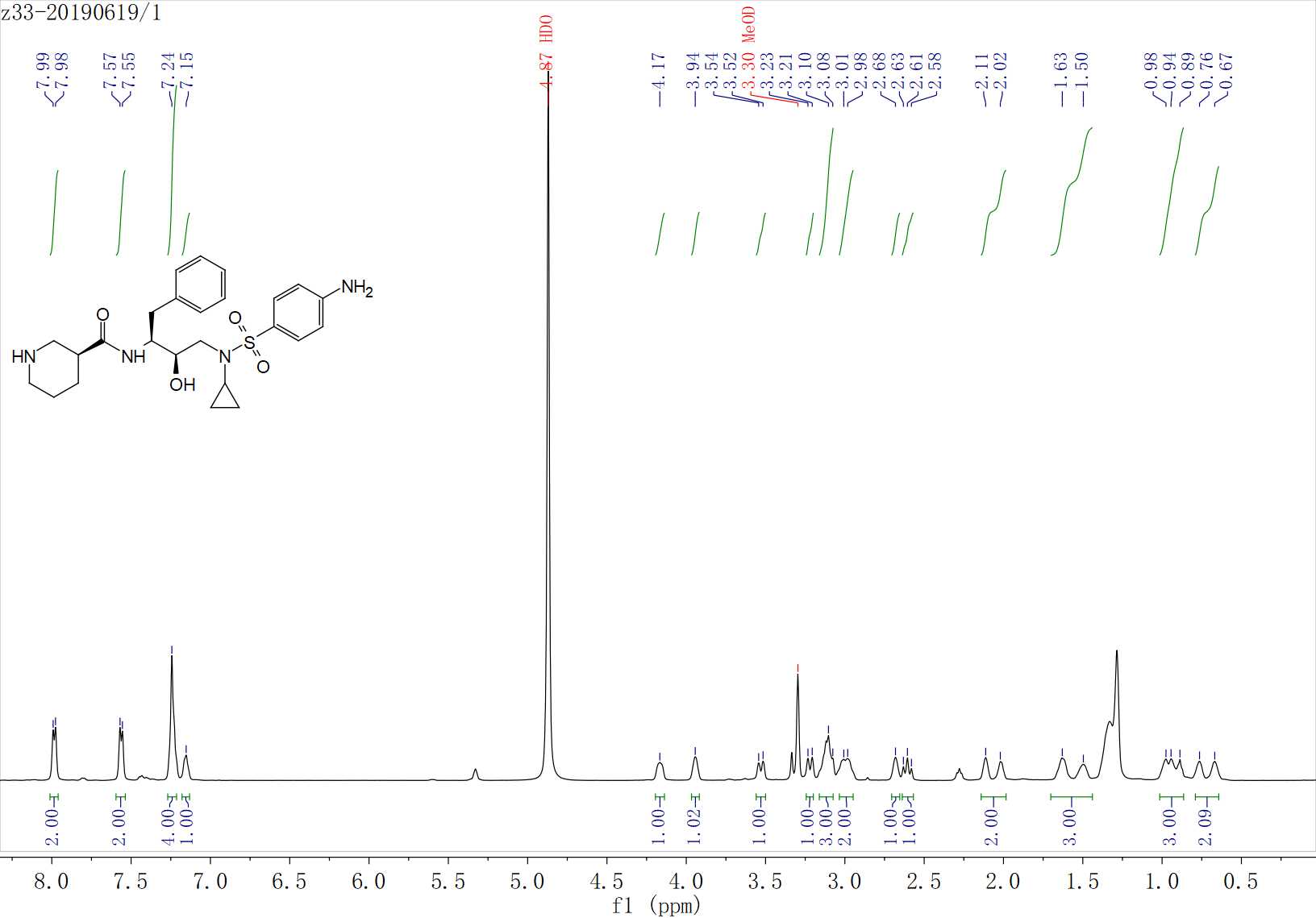


**Fig. S50.** ^1^H NMR Spectrum of compound **23d**


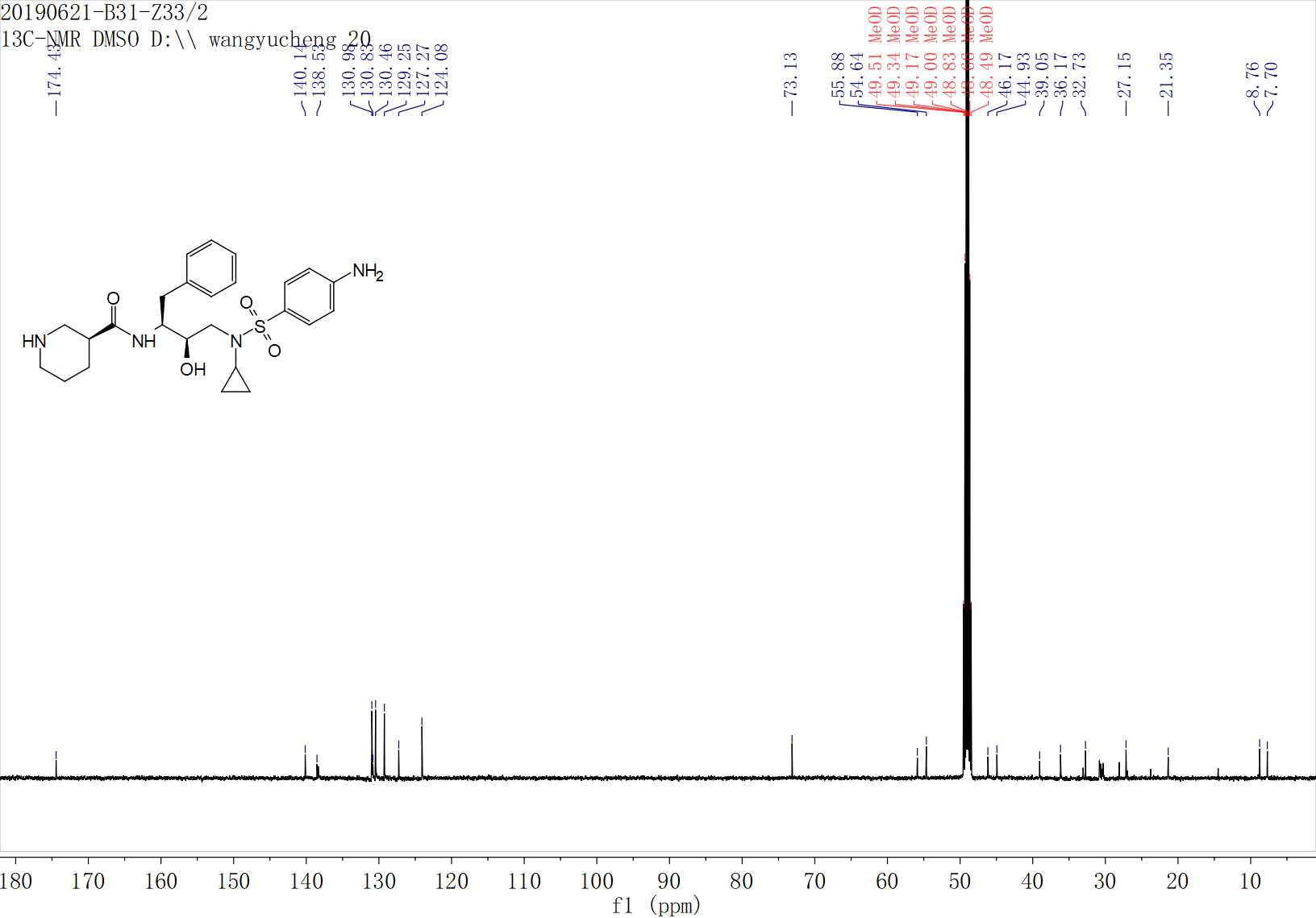


**Fig. S51.** ^13^C NMR Spectrum of compound **23d**

**
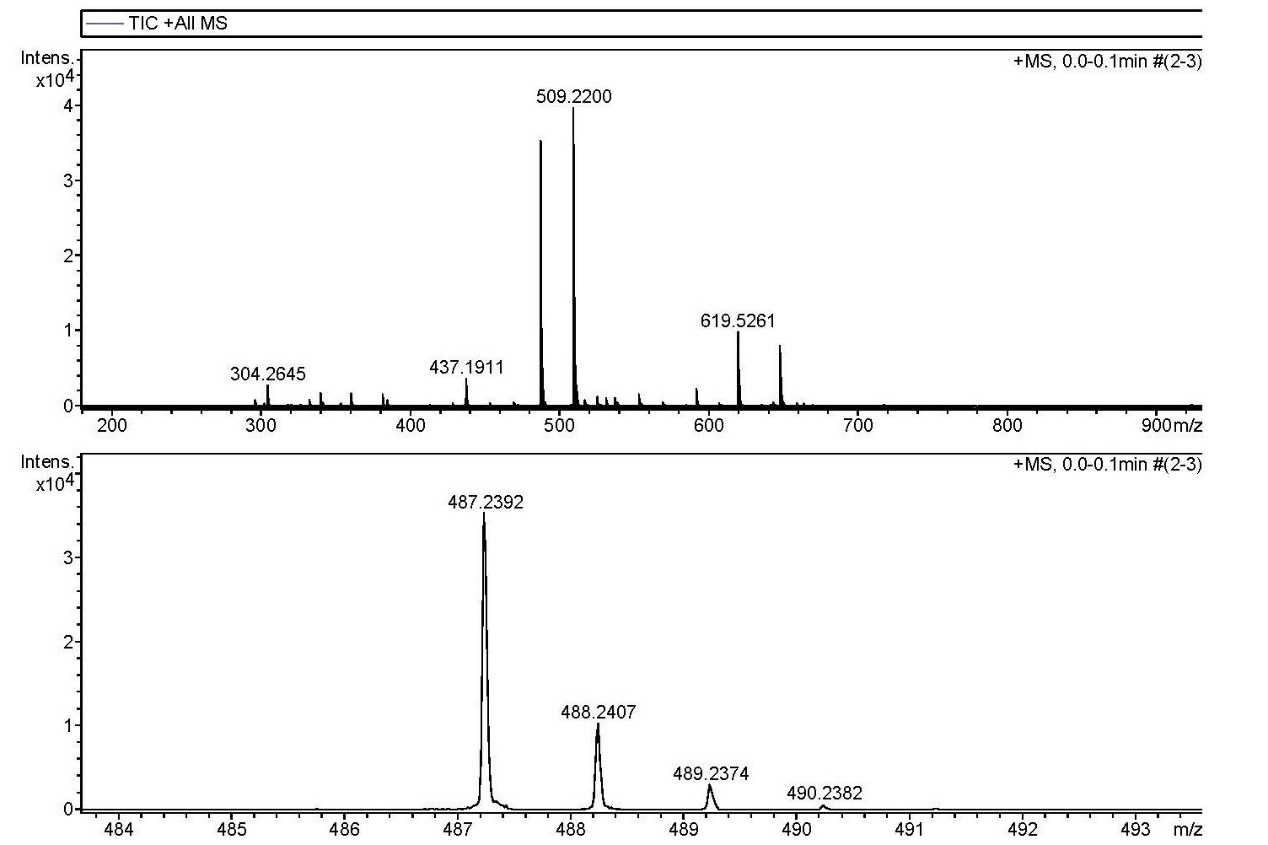
**

**Fig. S52.** HR MS Spectrum of compound **23d**

**
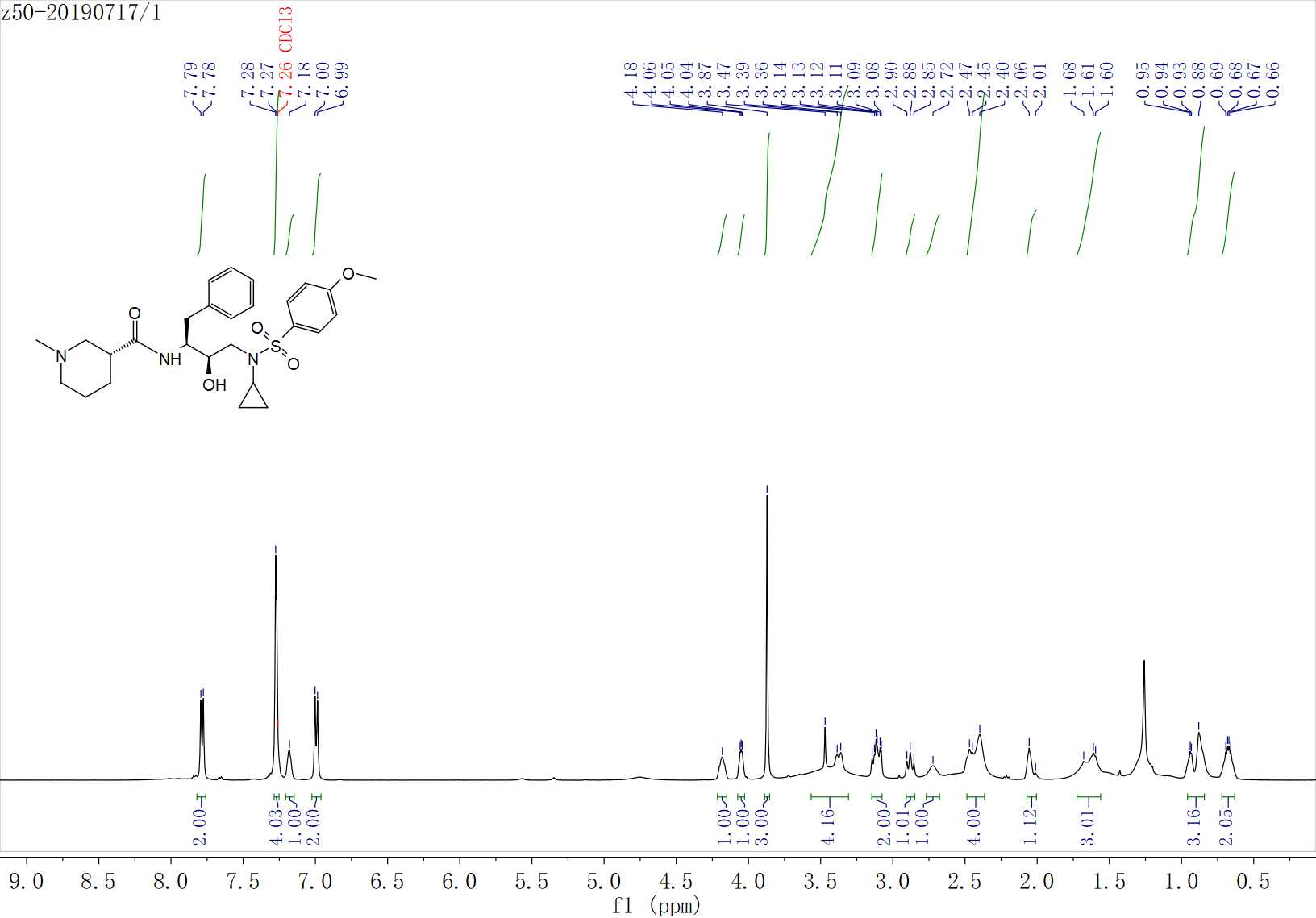
**

**Fig. S53.** ^1^H NMR Spectrum of compound **24a**


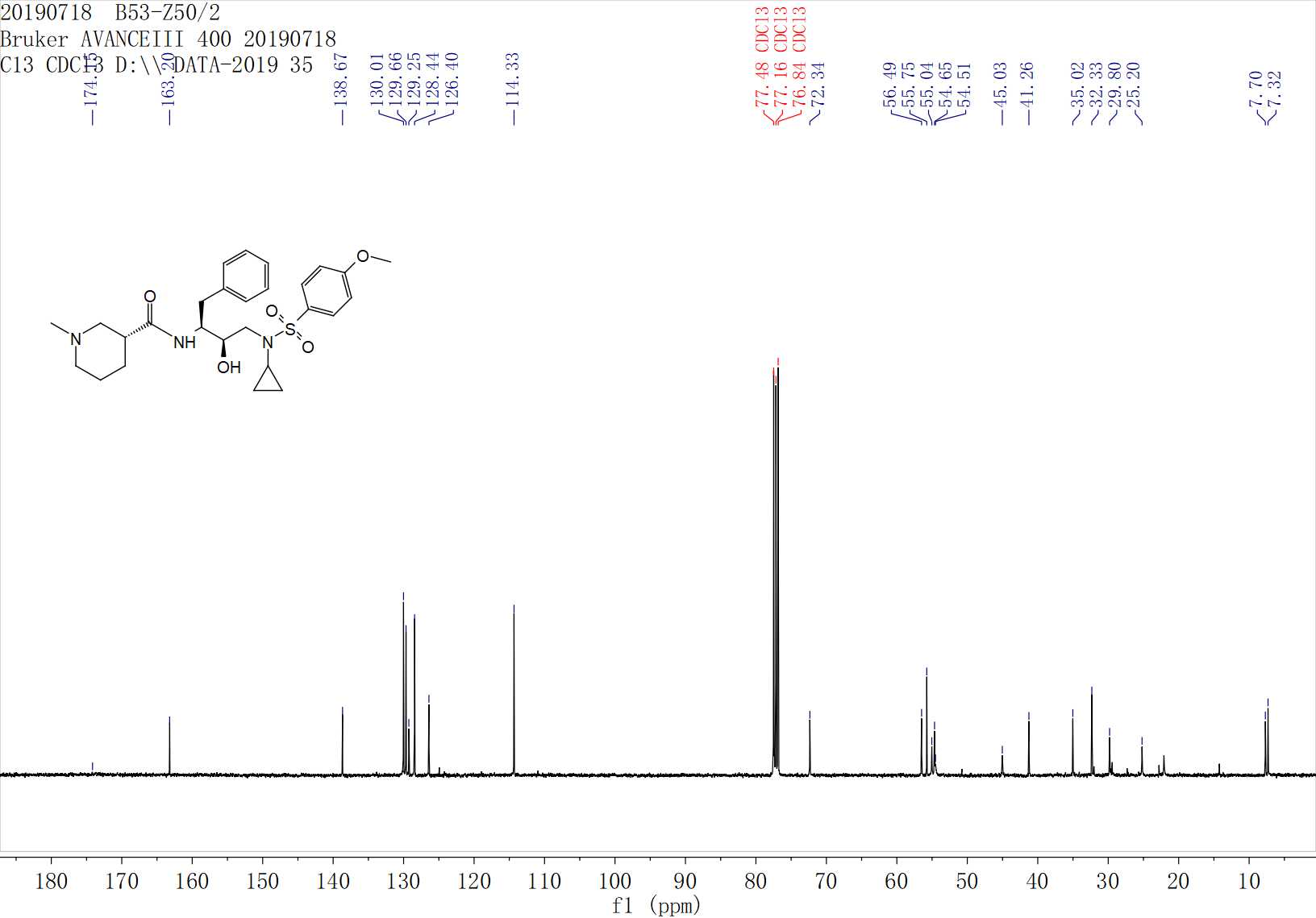


**Fig. S54.** ^13^C NMR Spectrum of compound **24a**

**
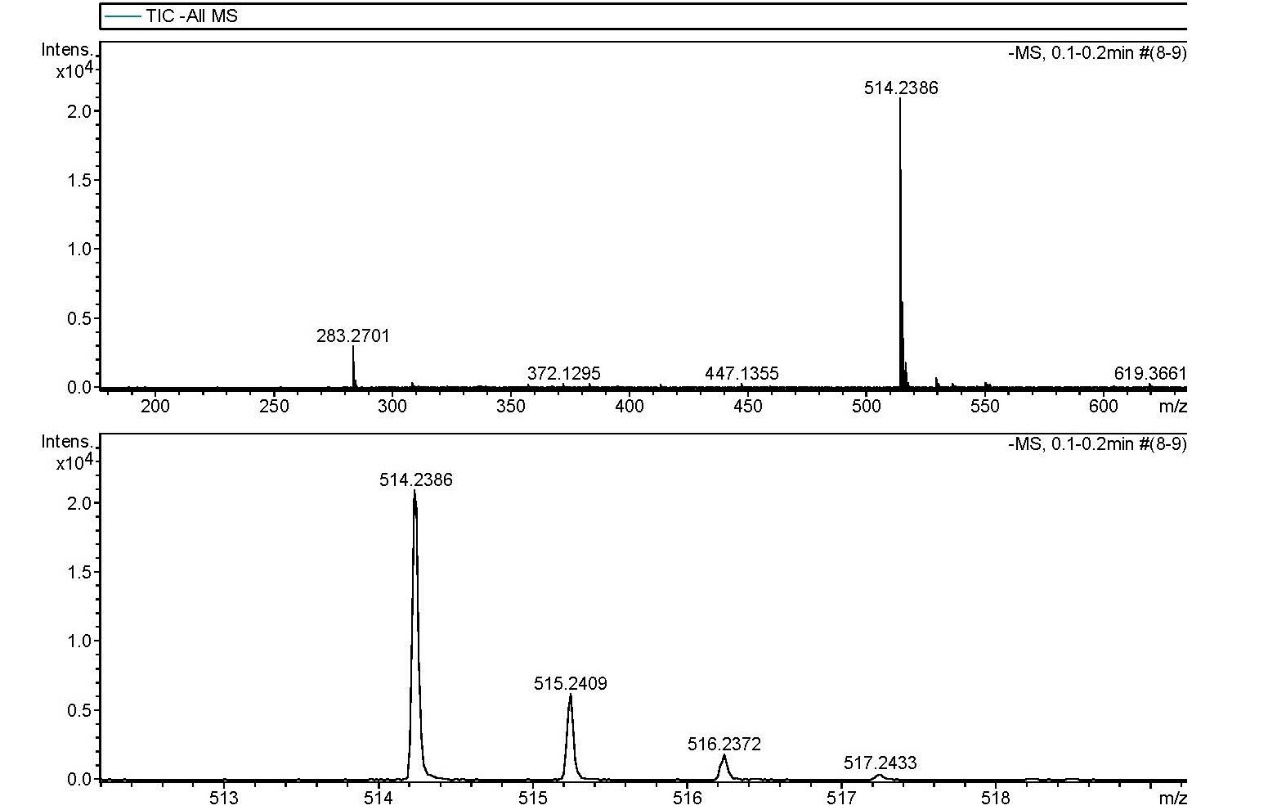
**

**Fig. S55.** HR MS Spectrum of compound **24a**


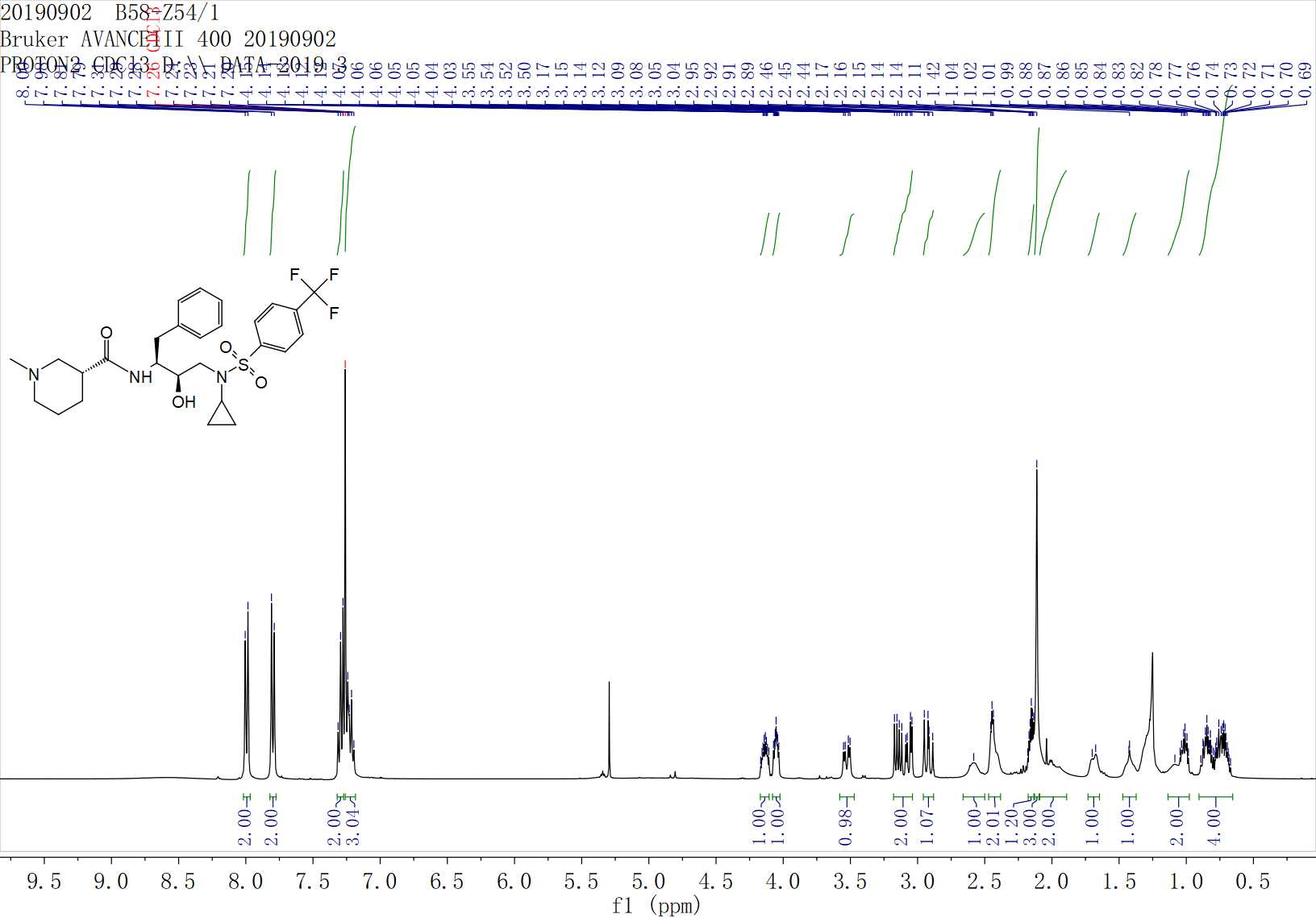


**Fig. S56.** ^1^H NMR Spectrum of compound **24b**


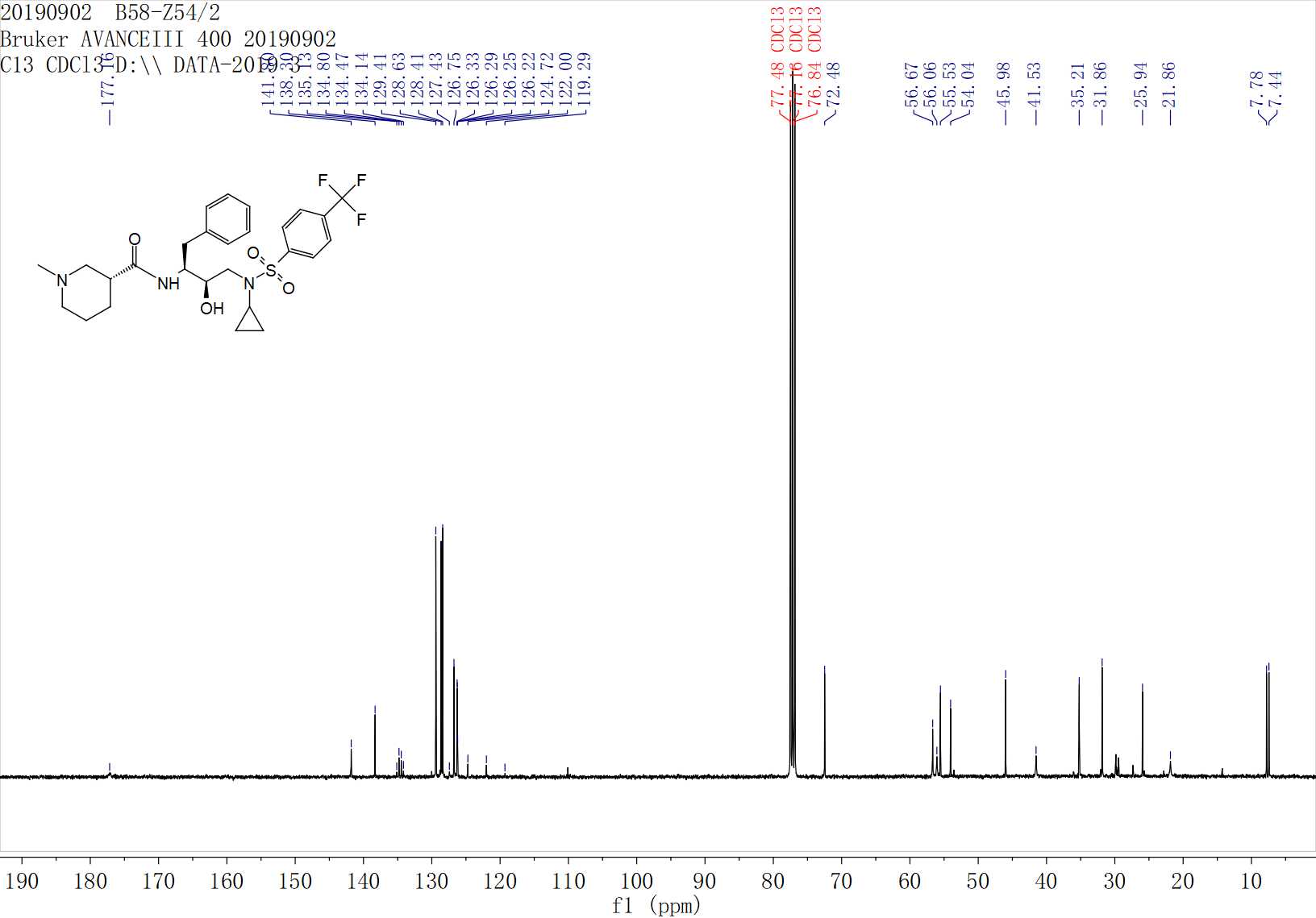


**Fig. S57.** ^13^C NMR Spectrum of compound **24b**

**
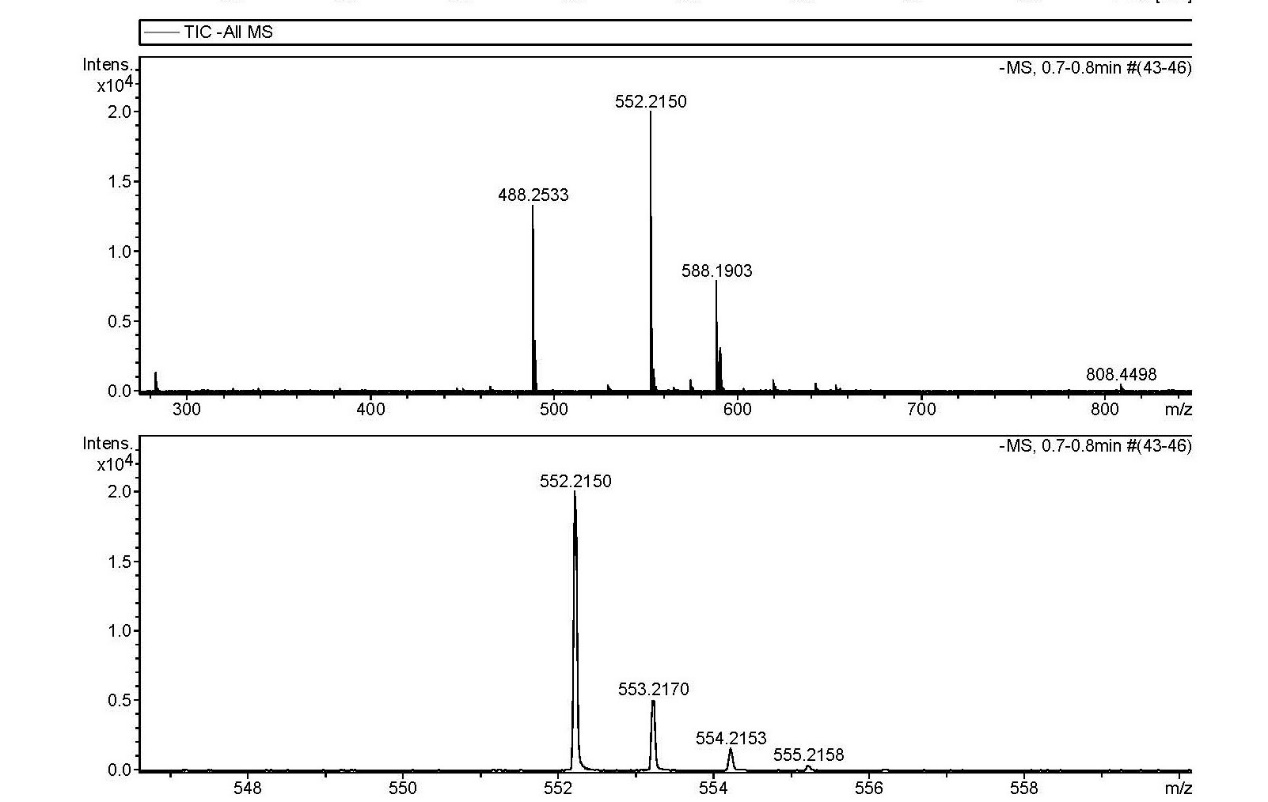
**

**Fig. S58.** HR MS Spectrum of compound **24b**


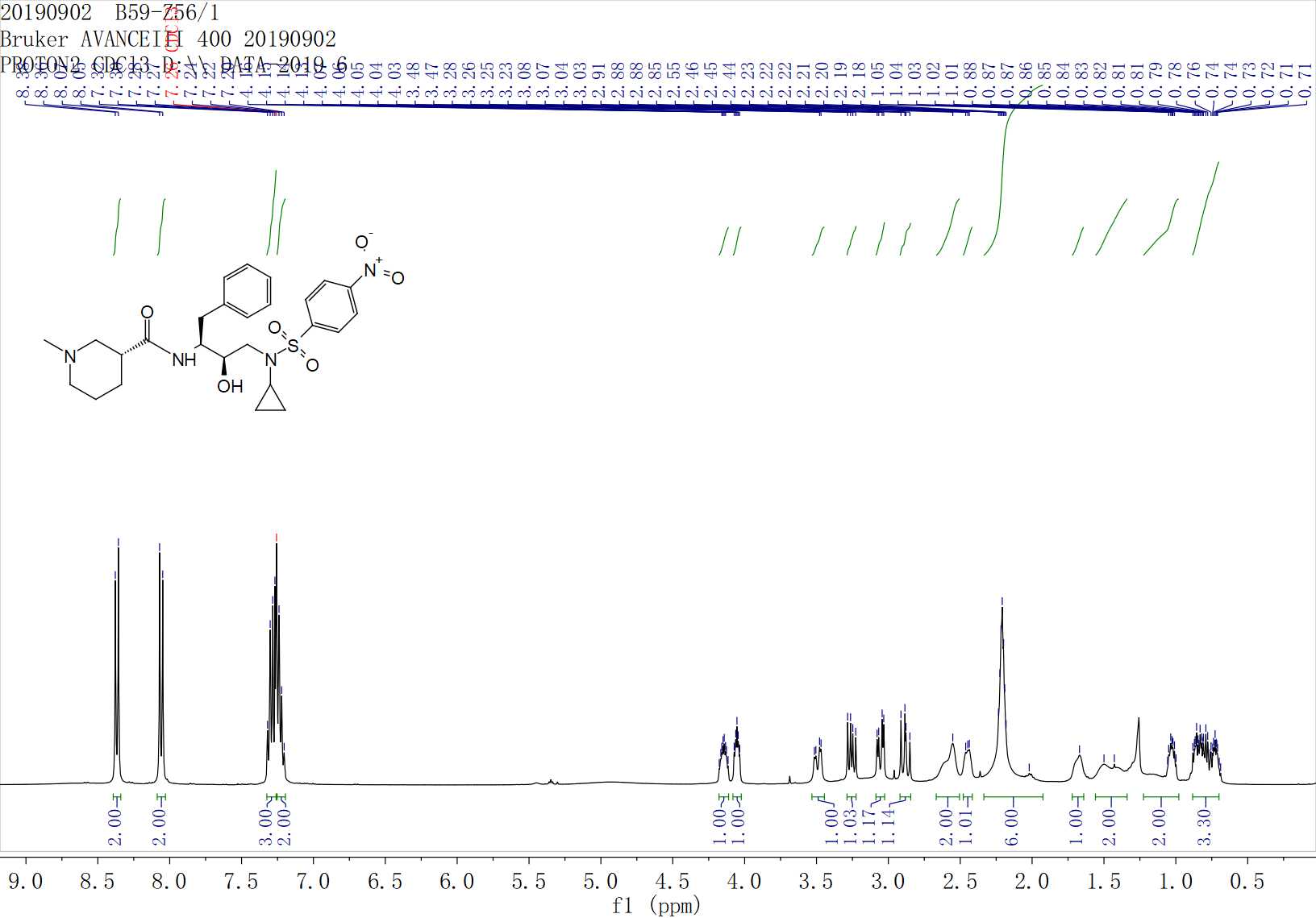


**Fig. S59.** ^1^H NMR Spectrum of compound **24c**


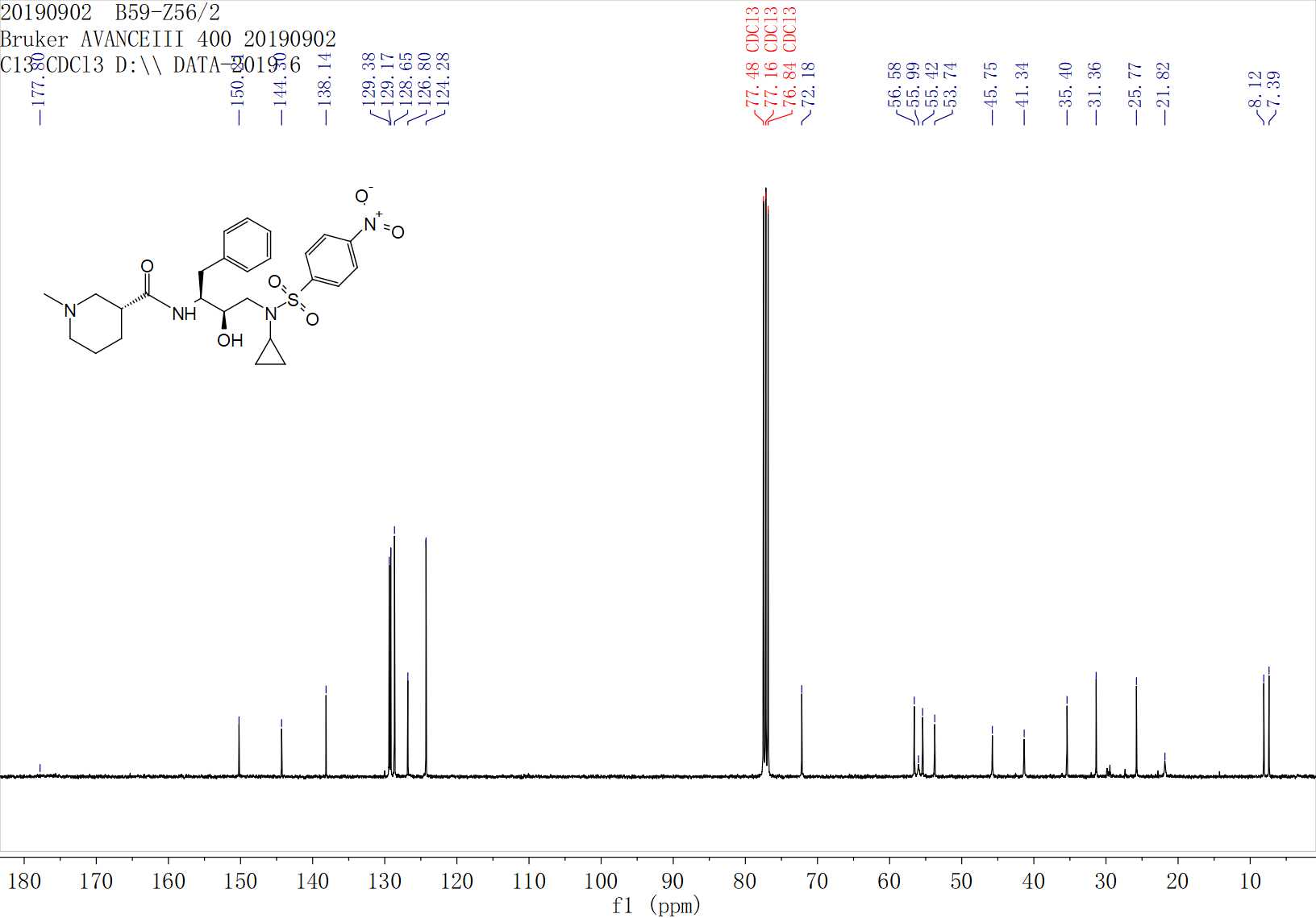


**Fig. S60.** ^13^C NMR Spectrum of compound **24c**

**
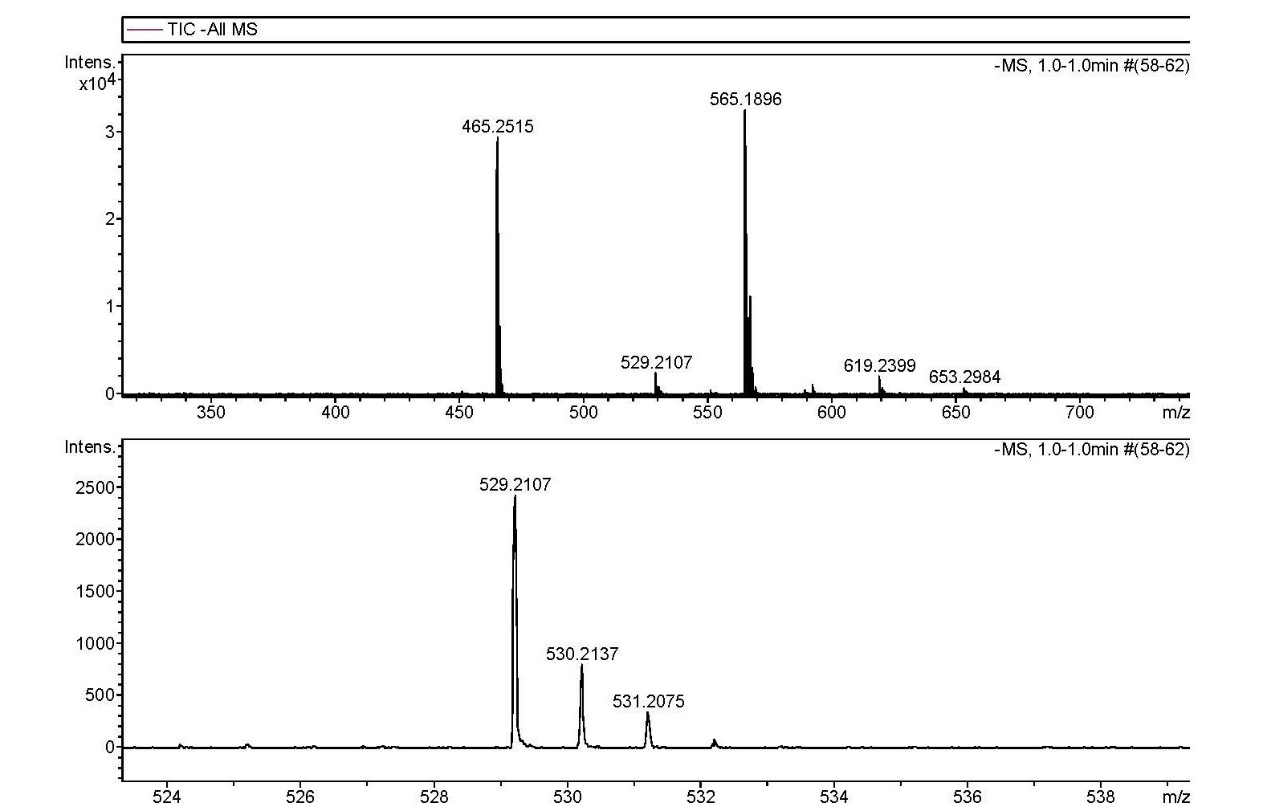
**

**Fig. S61.** HR MS Spectrum of compound **24c**


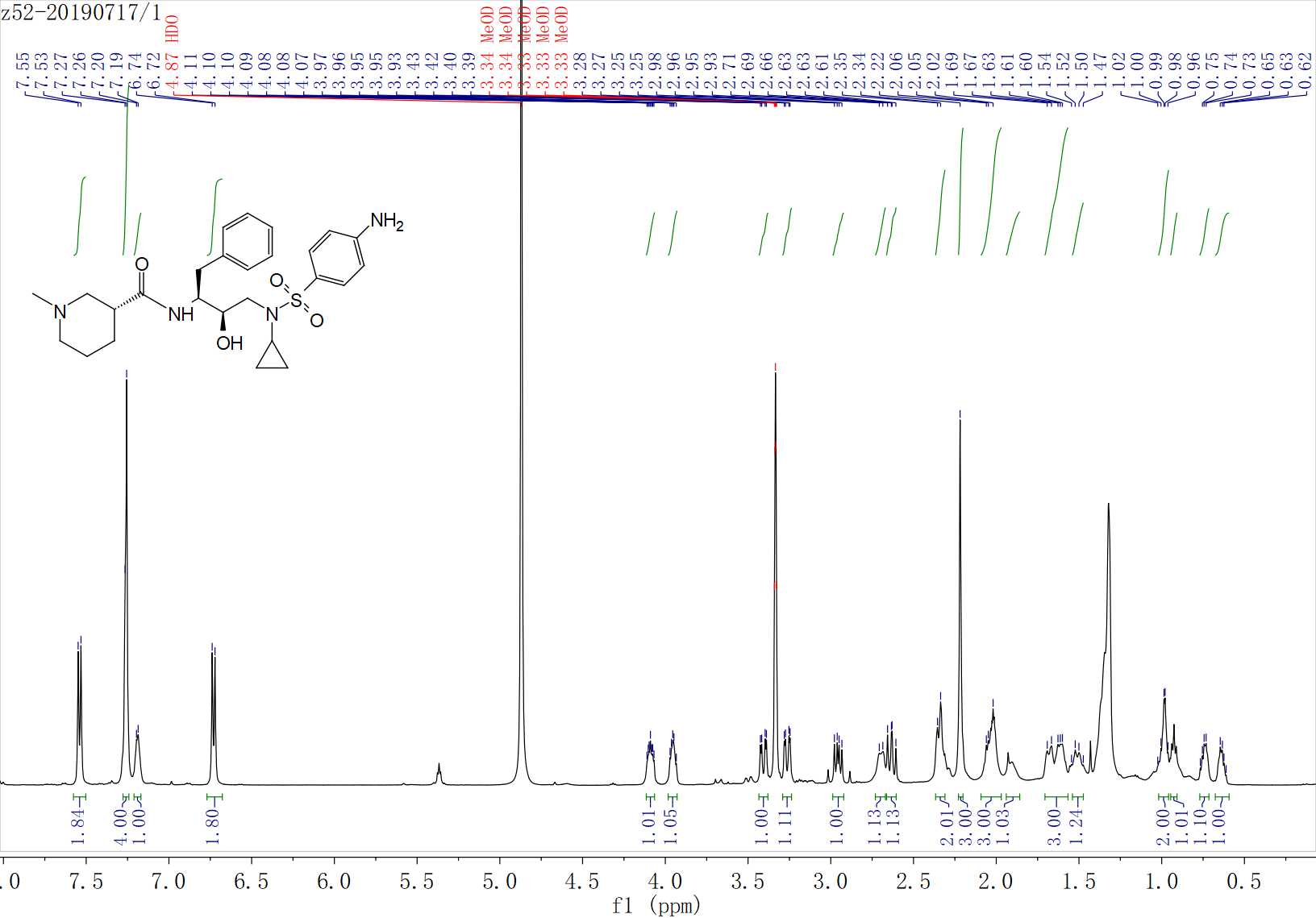


**Fig. S62.** ^1^H NMR Spectrum of compound **24d**


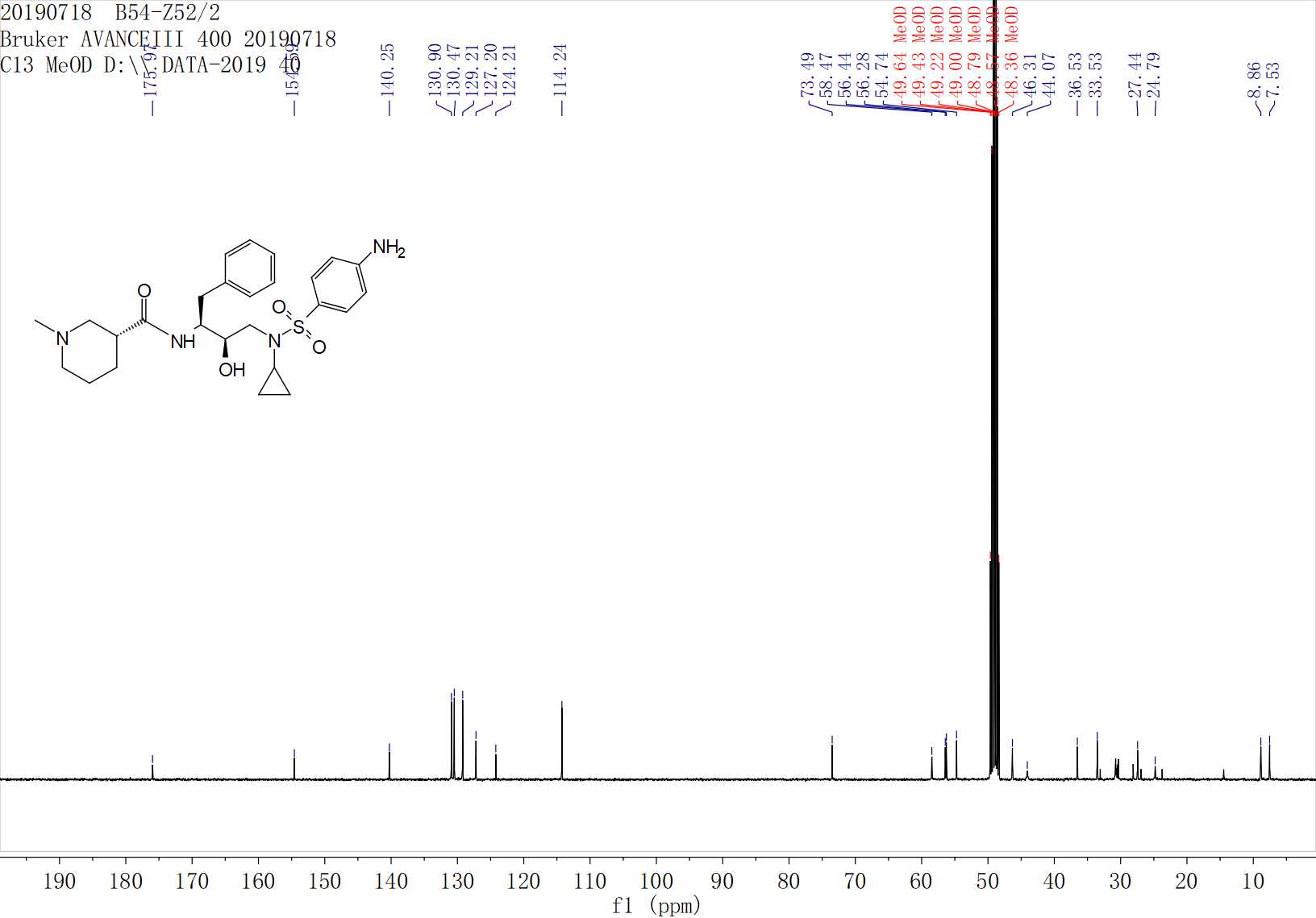


**Fig. S63.** ^13^C NMR Spectrum of compound **24d**

**
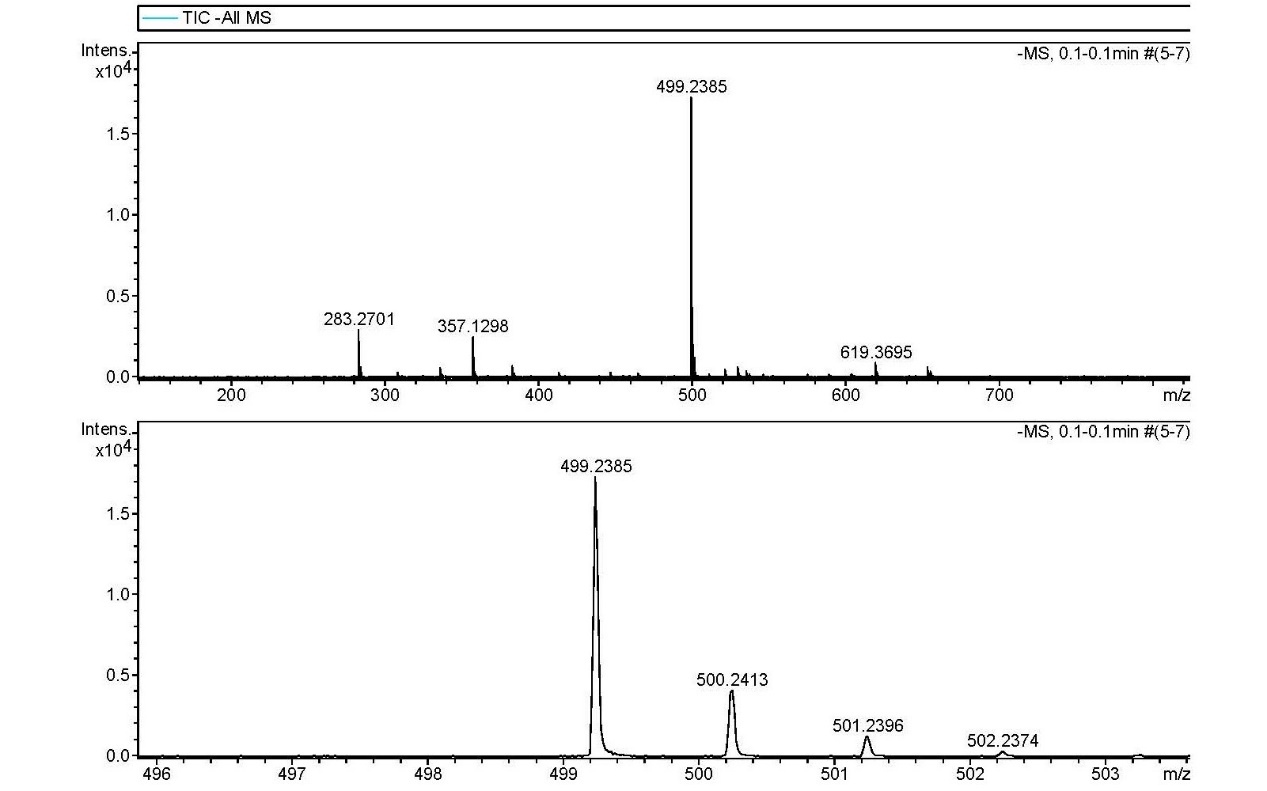
**

**Fig. S64.** HR MS Spectrum of compound **24d**


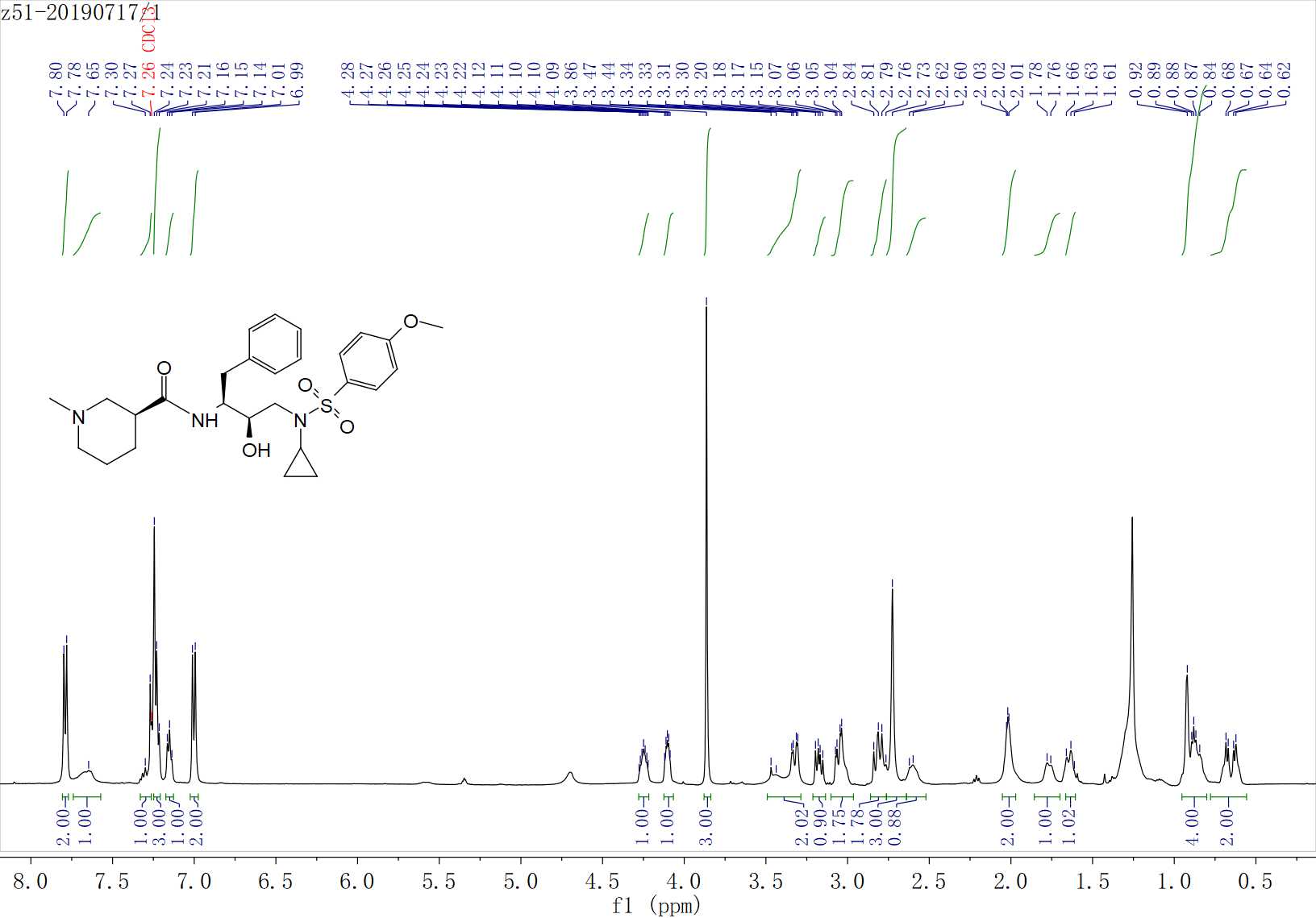


**Fig. S65.** ^1^H NMR Spectrum of compound **25a**


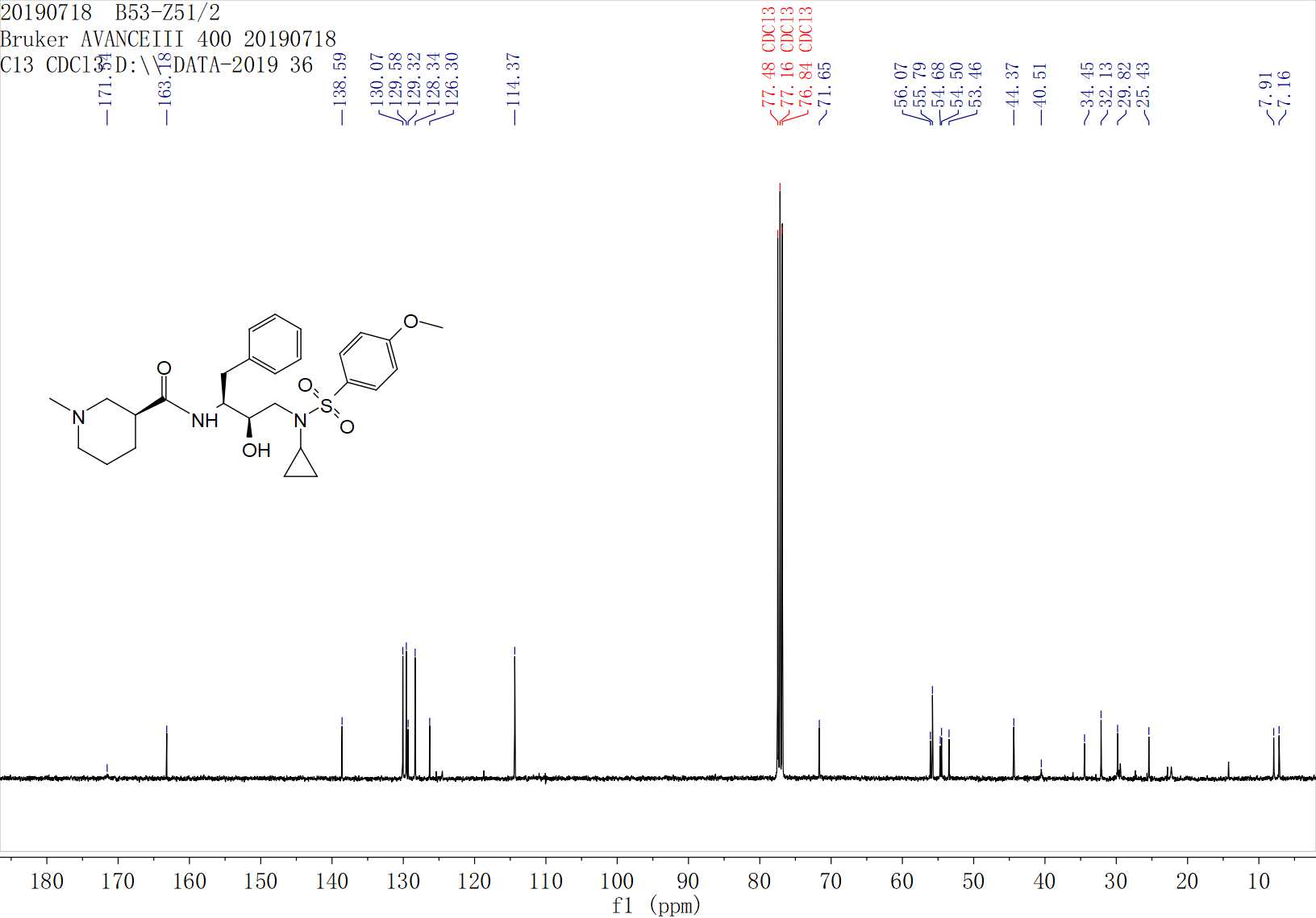


**Fig. S66.** ^13^C NMR Spectrum of compound **25a**

**
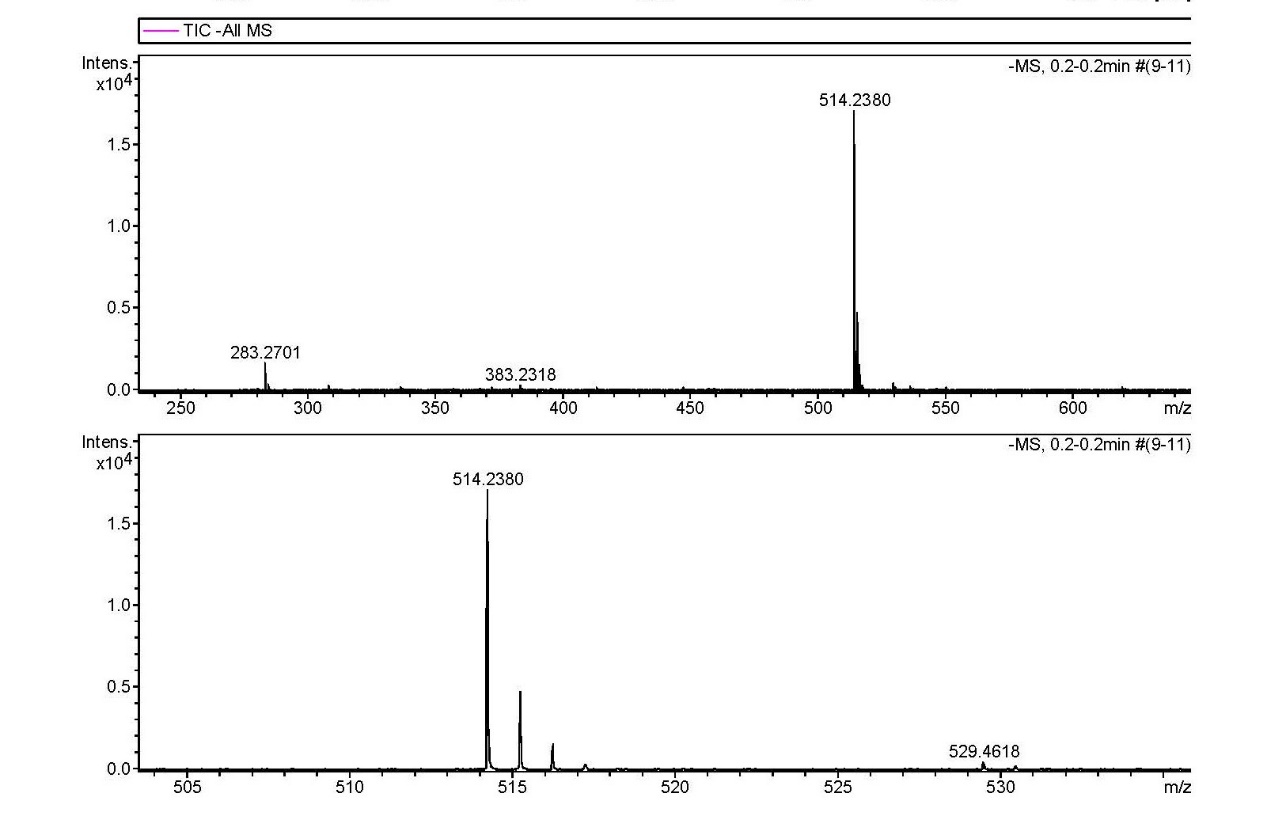
**

**Fig. S67.** HR MS Spectrum of compound **25a**


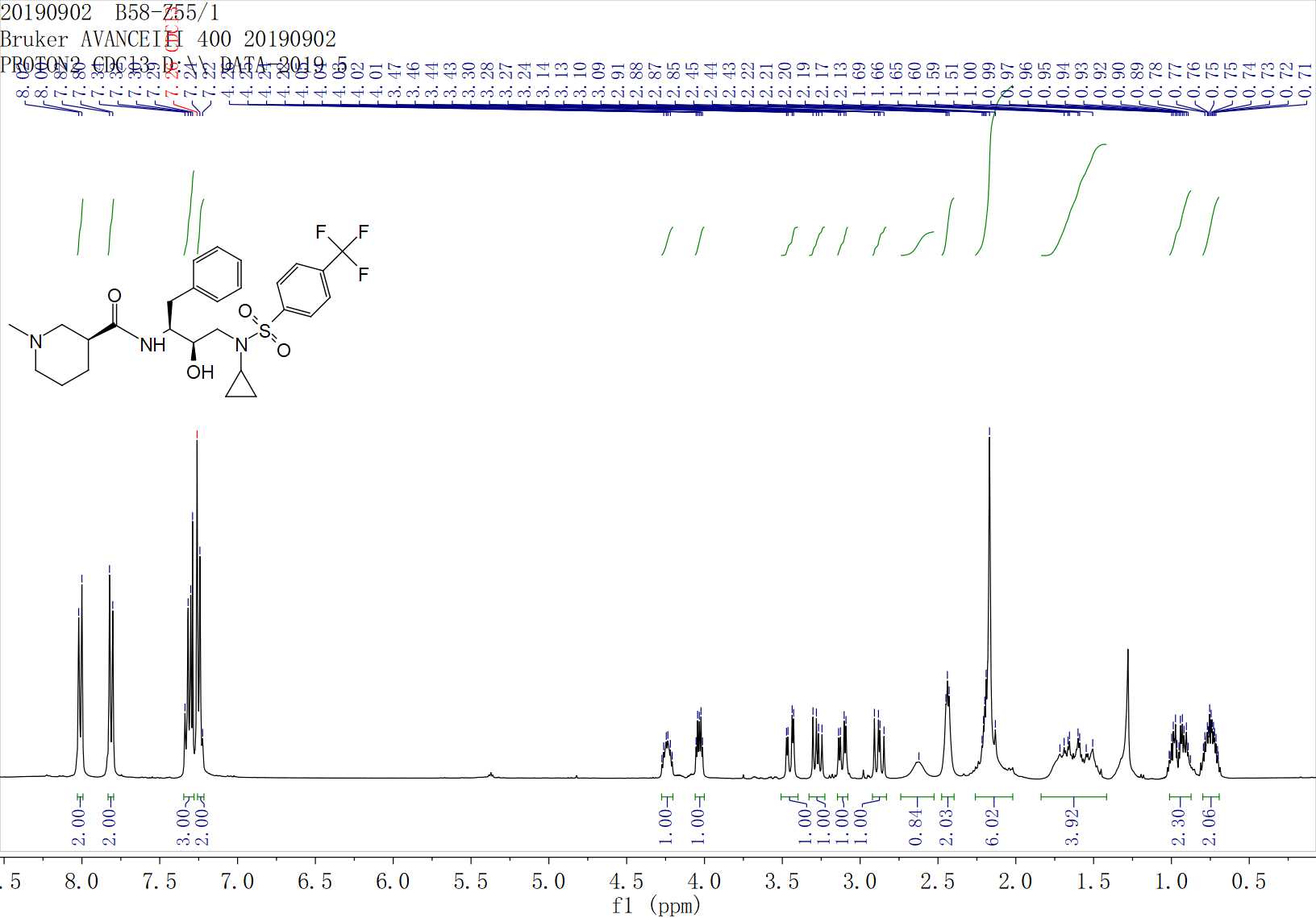


**Fig. S68.** ^1^H NMR Spectrum of compound **25b**


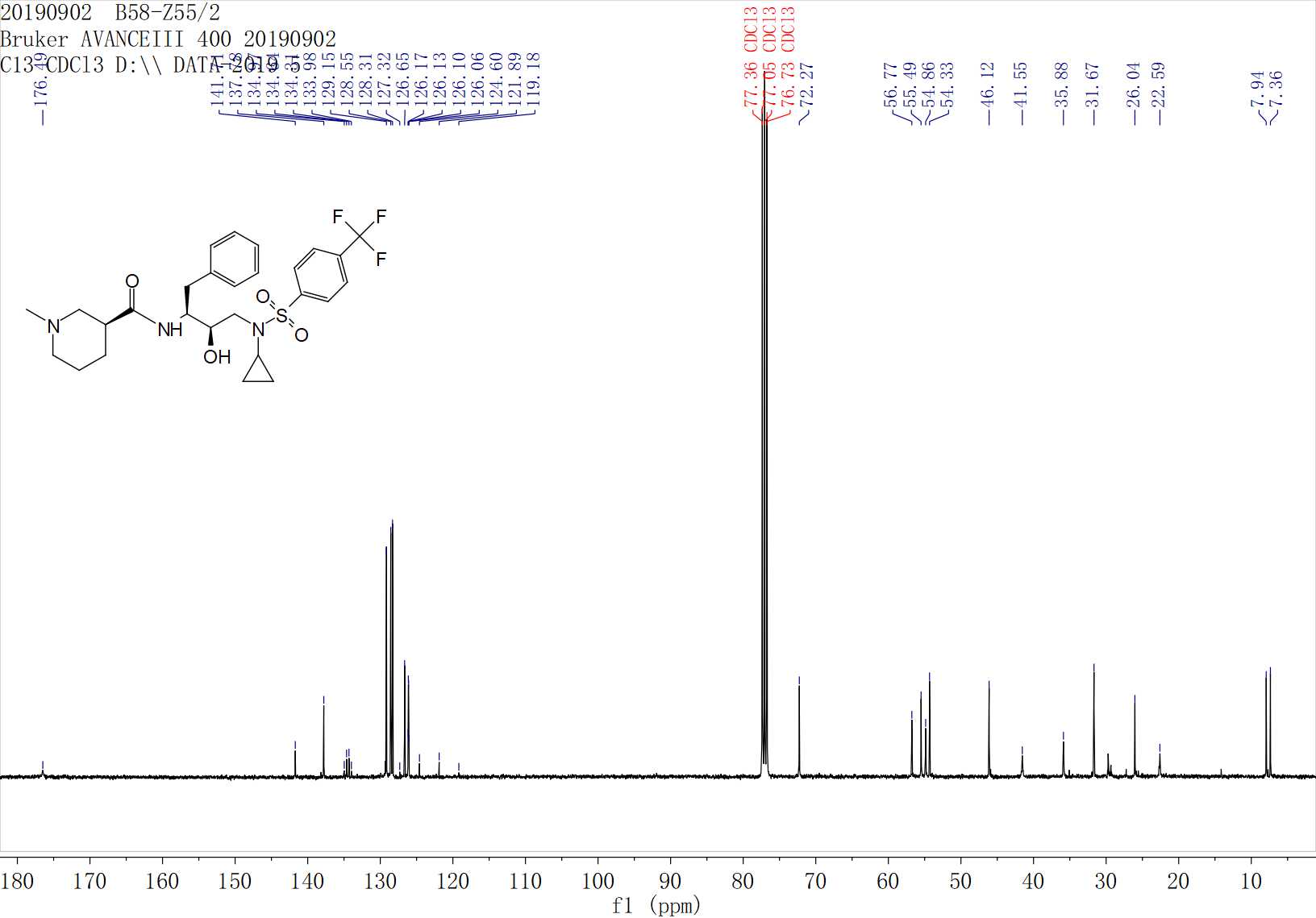


**Fig. S69.** ^13^C NMR Spectrum of compound **25b**

**
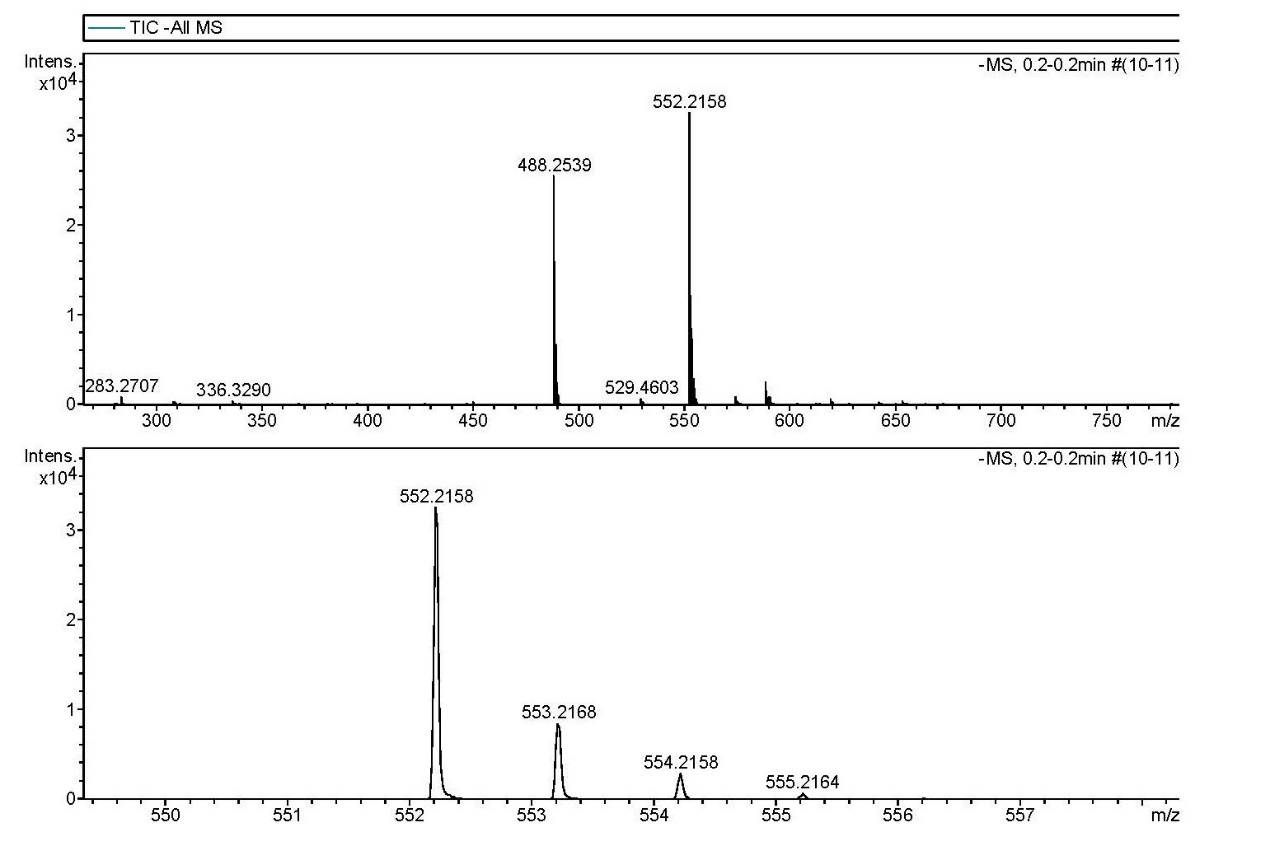
**

**Fig. S70.** HR MS Spectrum of compound **25b**


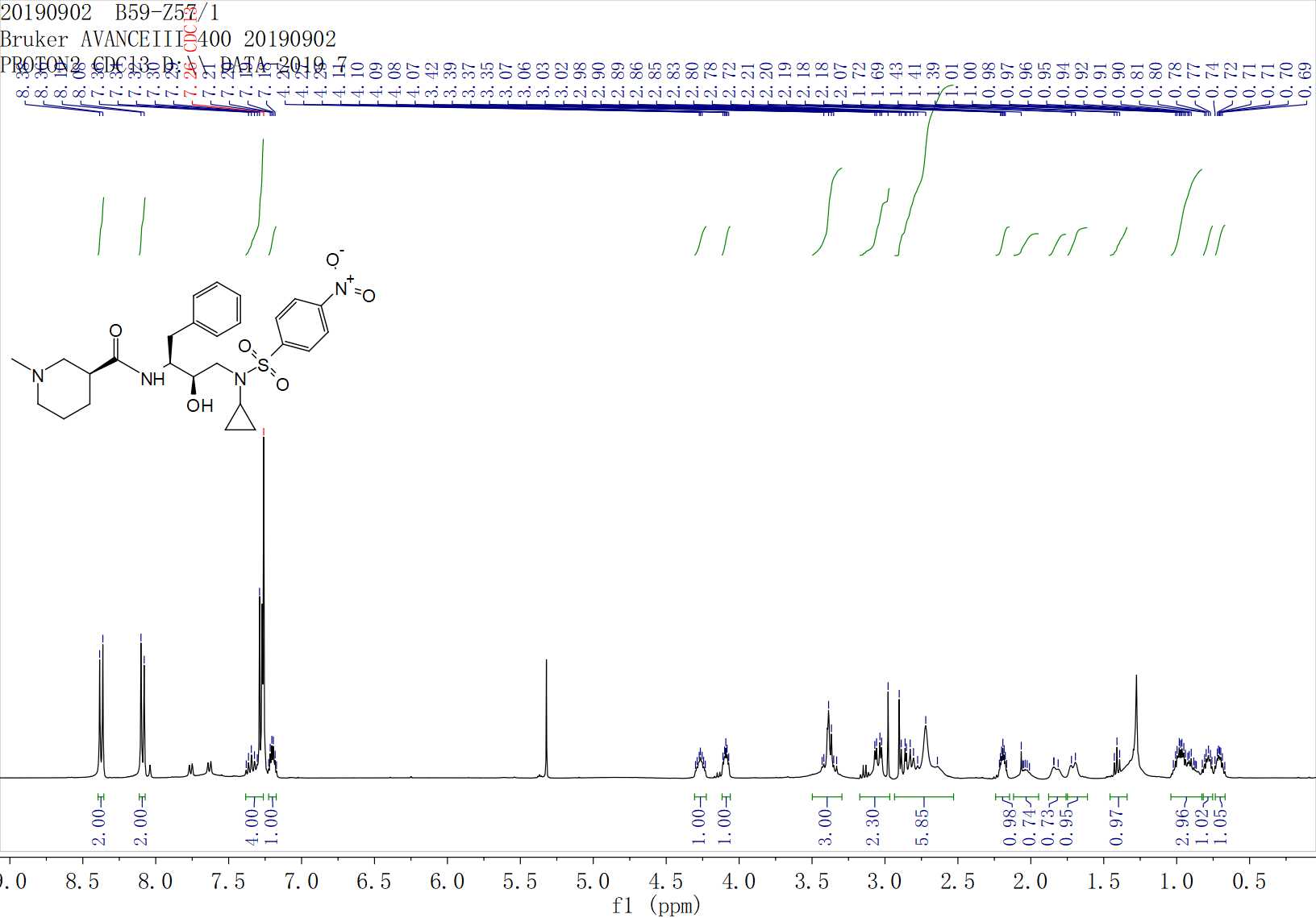


**Fig. S71.** ^1^H NMR Spectrum of compound **25c**


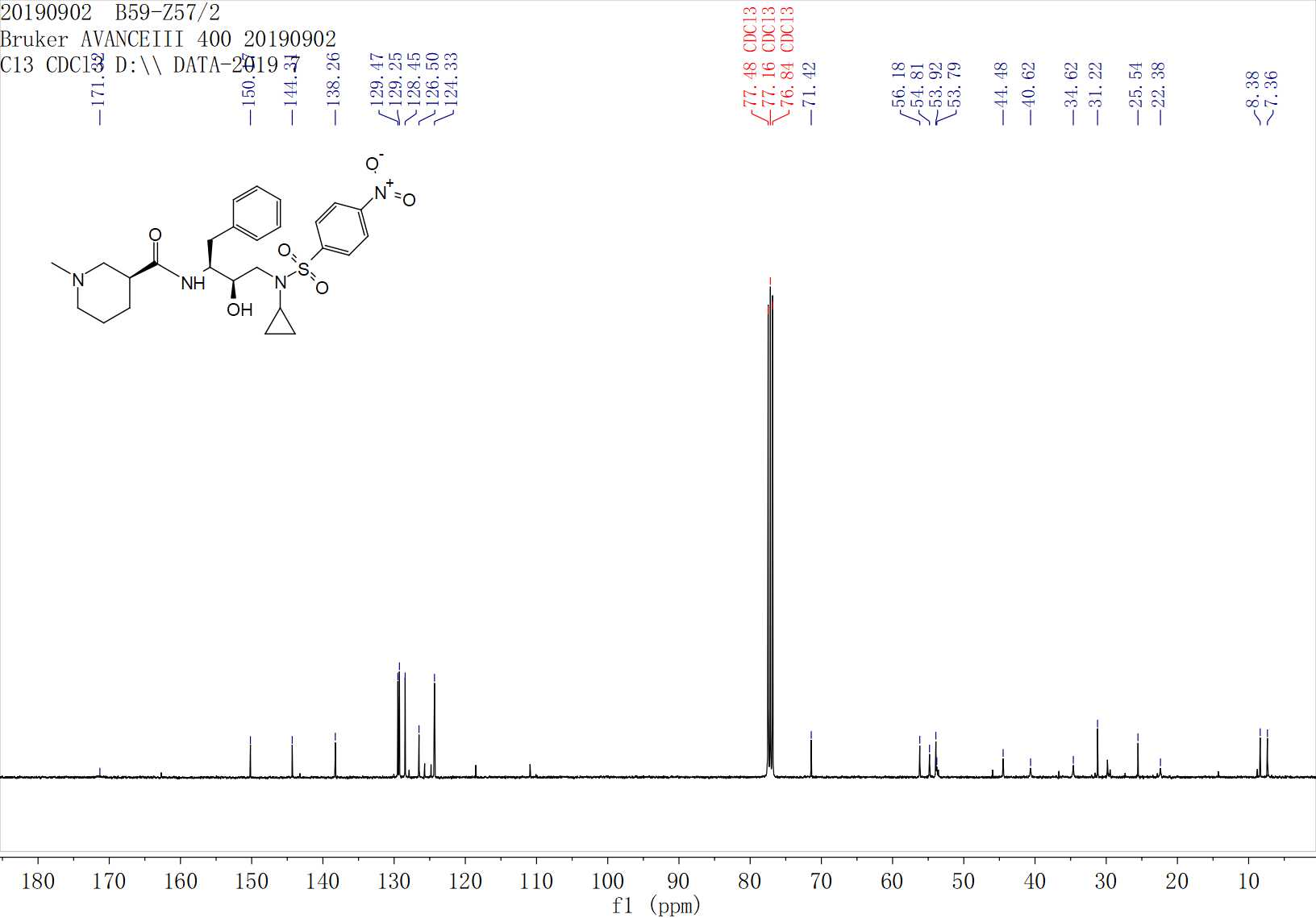


**Fig. S72.** ^13^C NMR Spectrum of compound **25c**

**
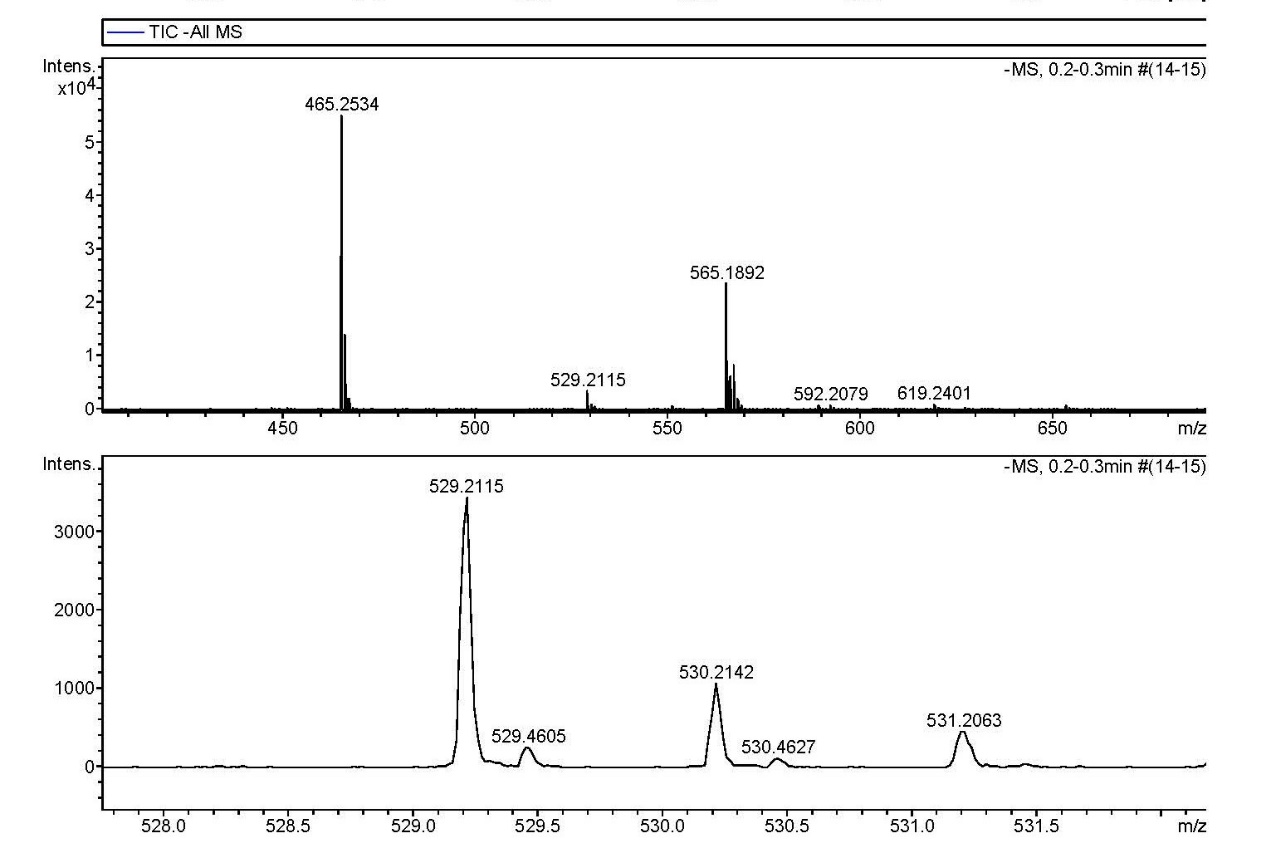
**

**Fig. S73.** HR MS Spectrum of compound **25c**


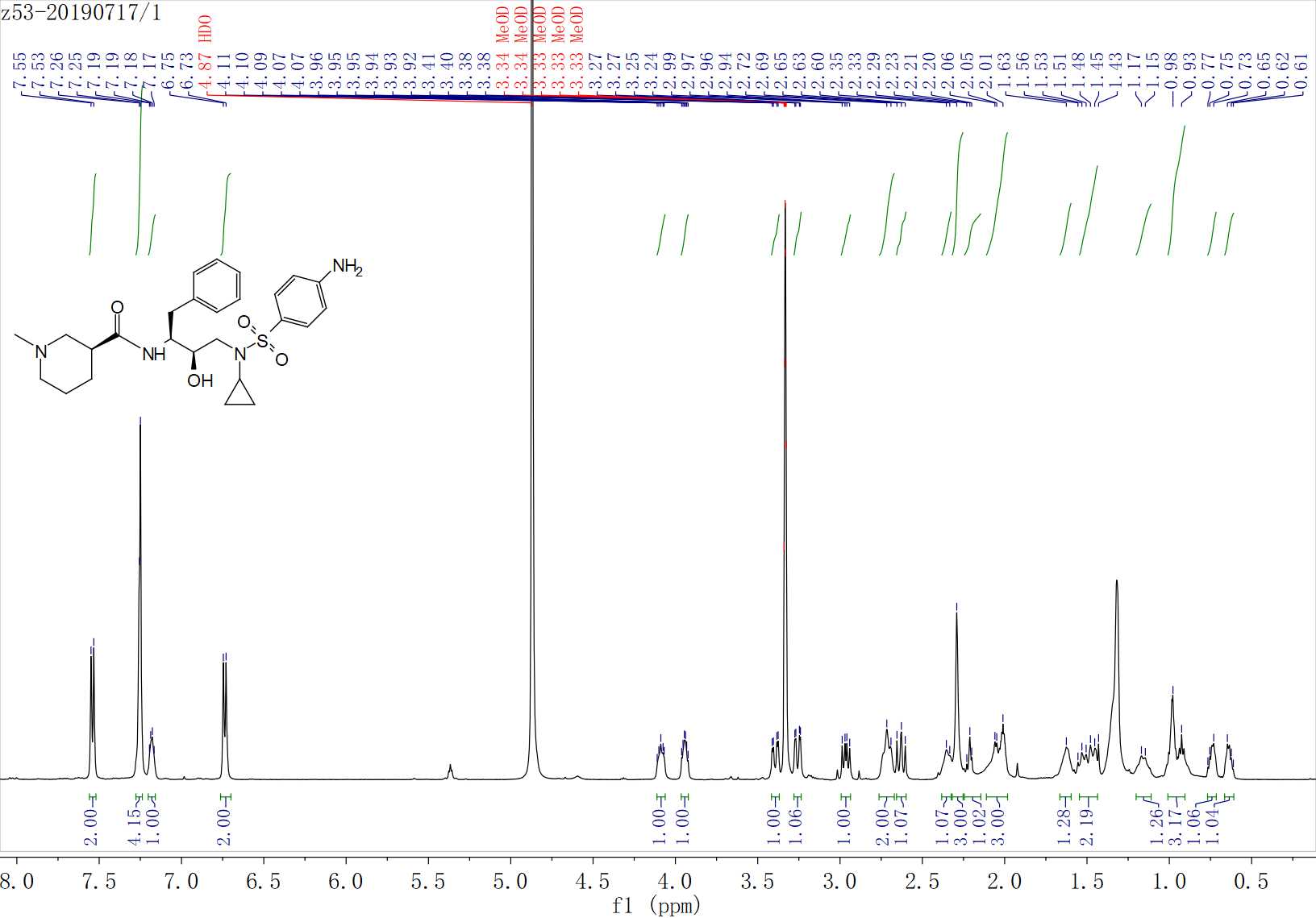


**Fig. S74.** ^1^H NMR Spectrum of compound **25d**


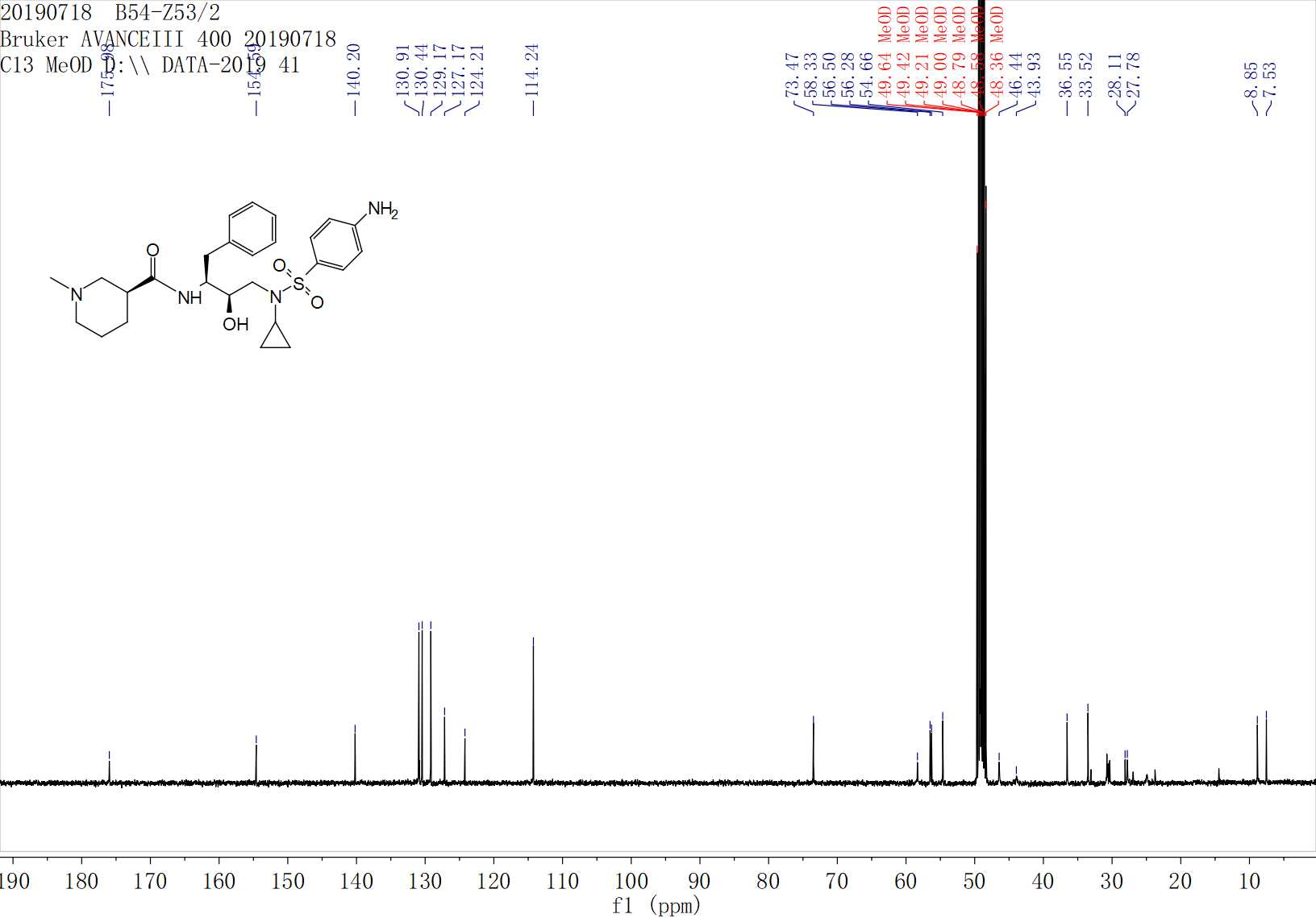


**Fig. S75.** ^13^C NMR Spectrum of compound **25d**

**
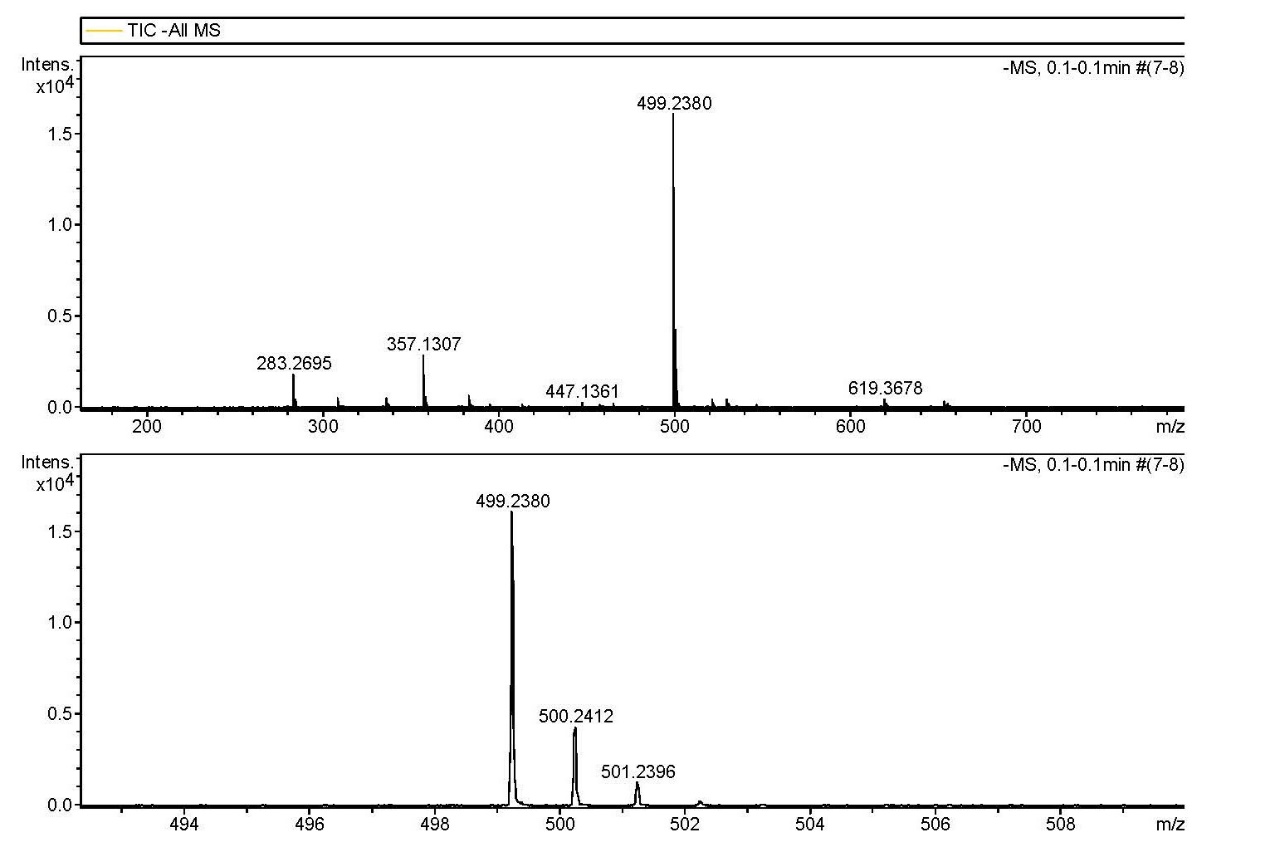
**

**Fig. S76.** HR MS Spectrum of compound **25d**


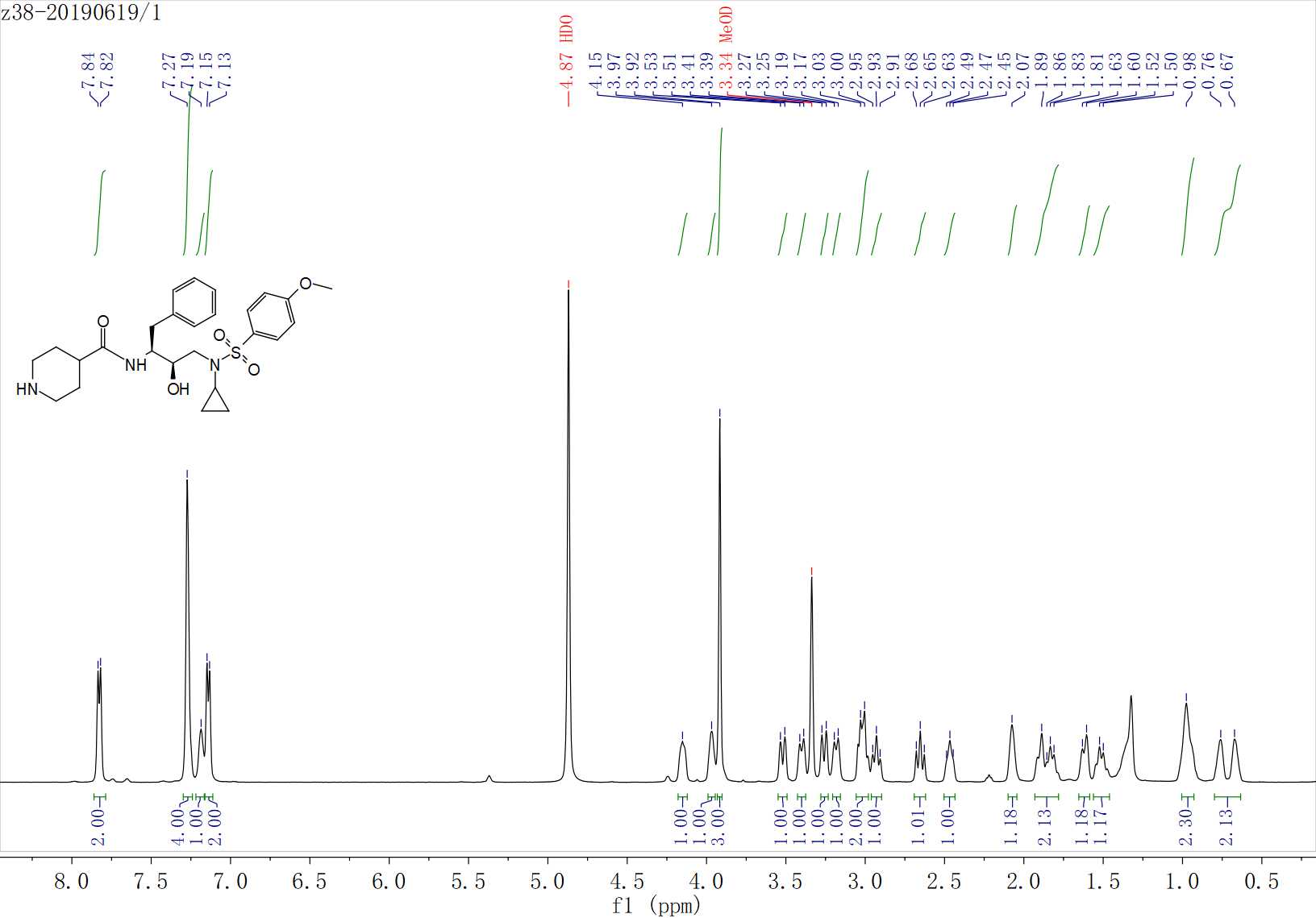


**Fig. S77.** ^1^H NMR Spectrum of compound **26a**


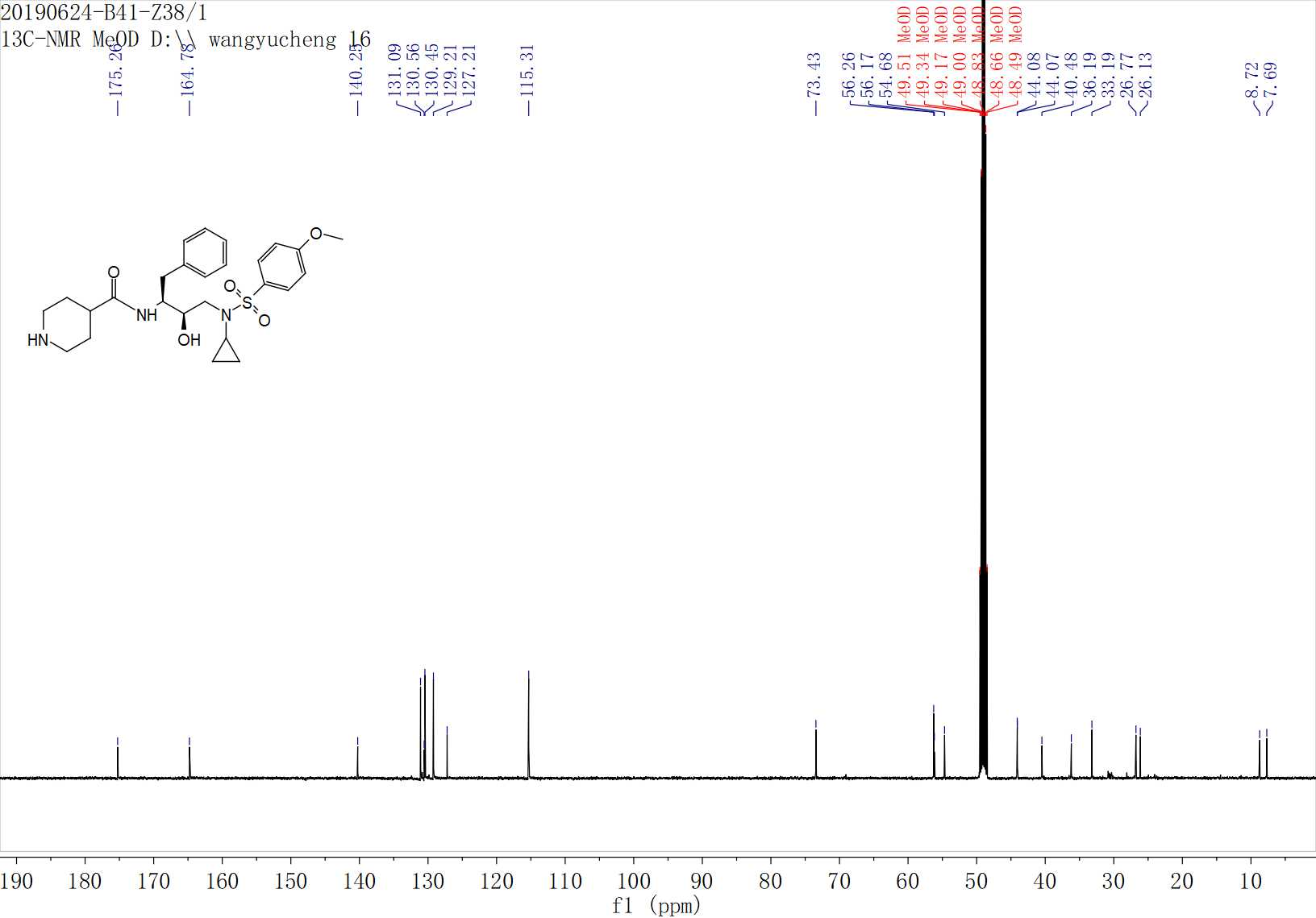


**Fig. S78.** ^13^C NMR Spectrum of compound **26a**

**
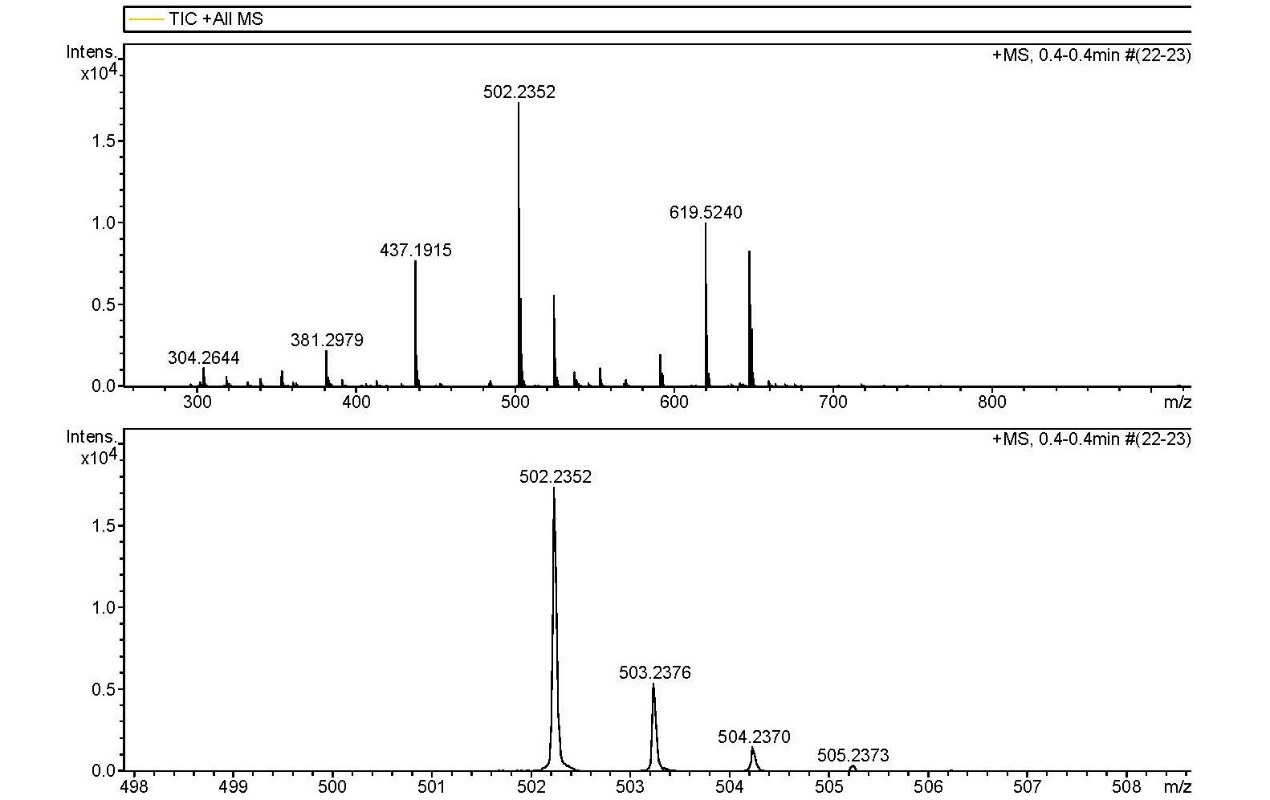
**

**Fig. S79.** HR MS Spectrum of compound **26a**


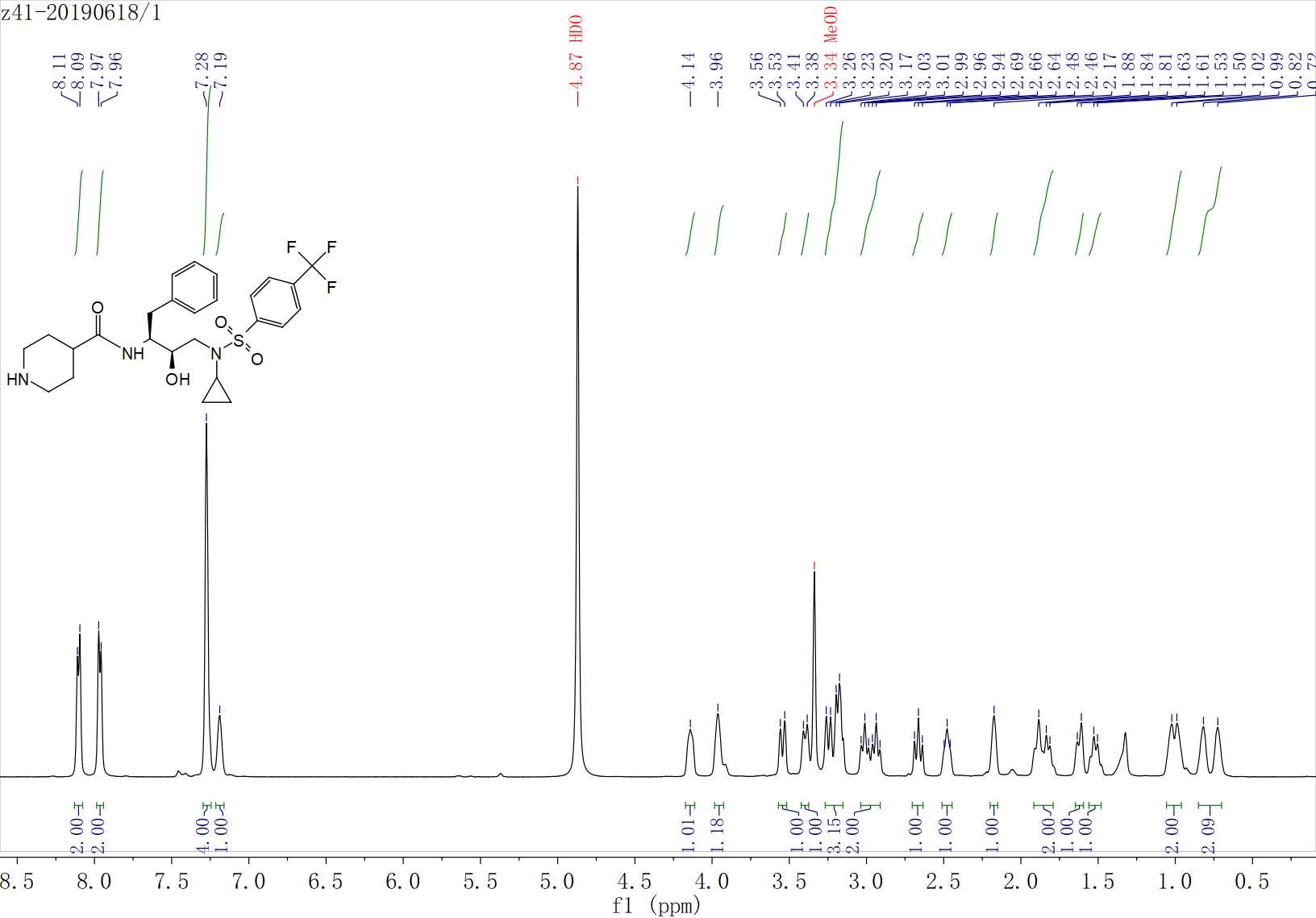


**Fig. S80.** ^1^H NMR Spectrum of compound **26b**


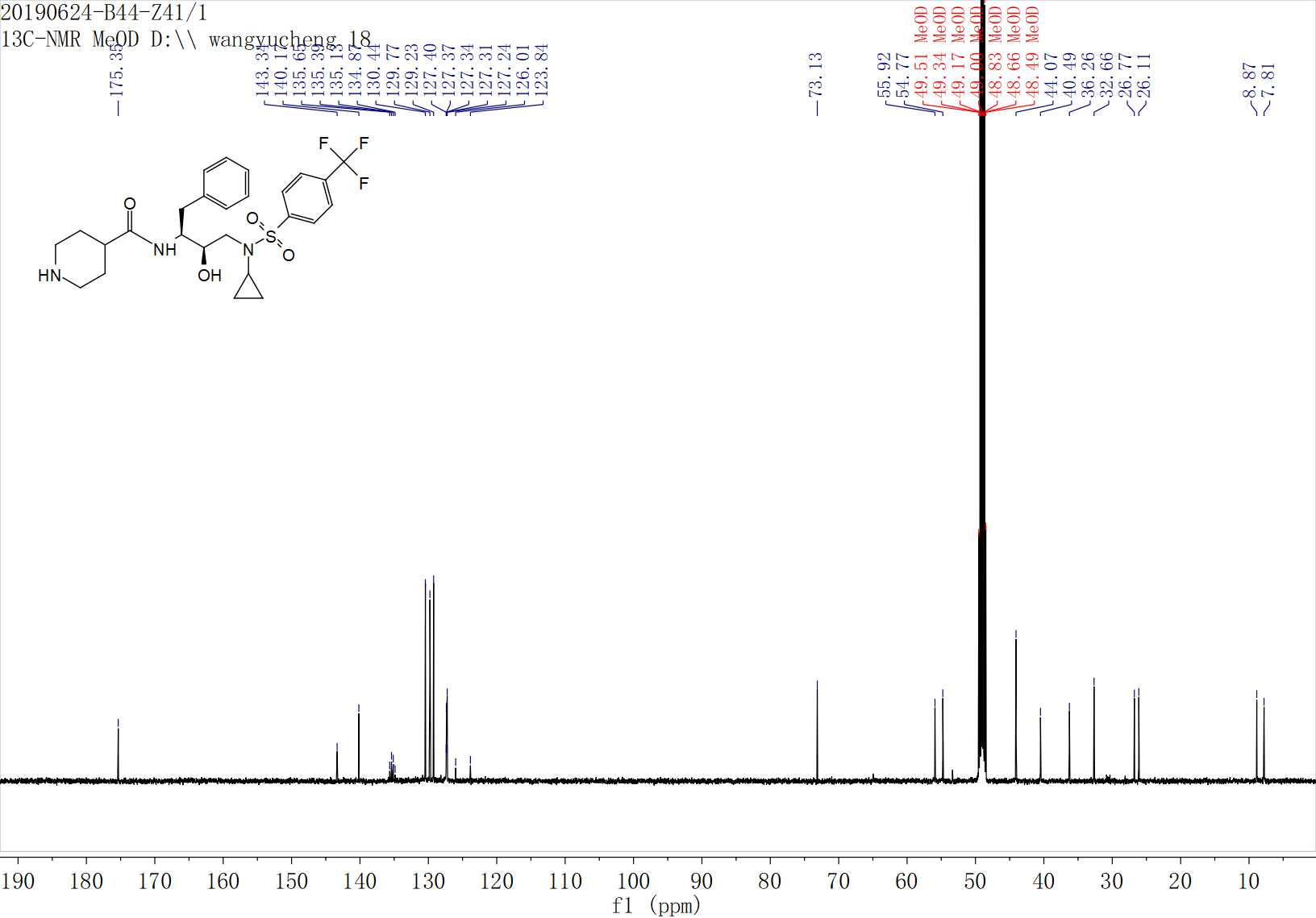


**Fig. S81.** ^13^C NMR Spectrum of compound **26b**

**
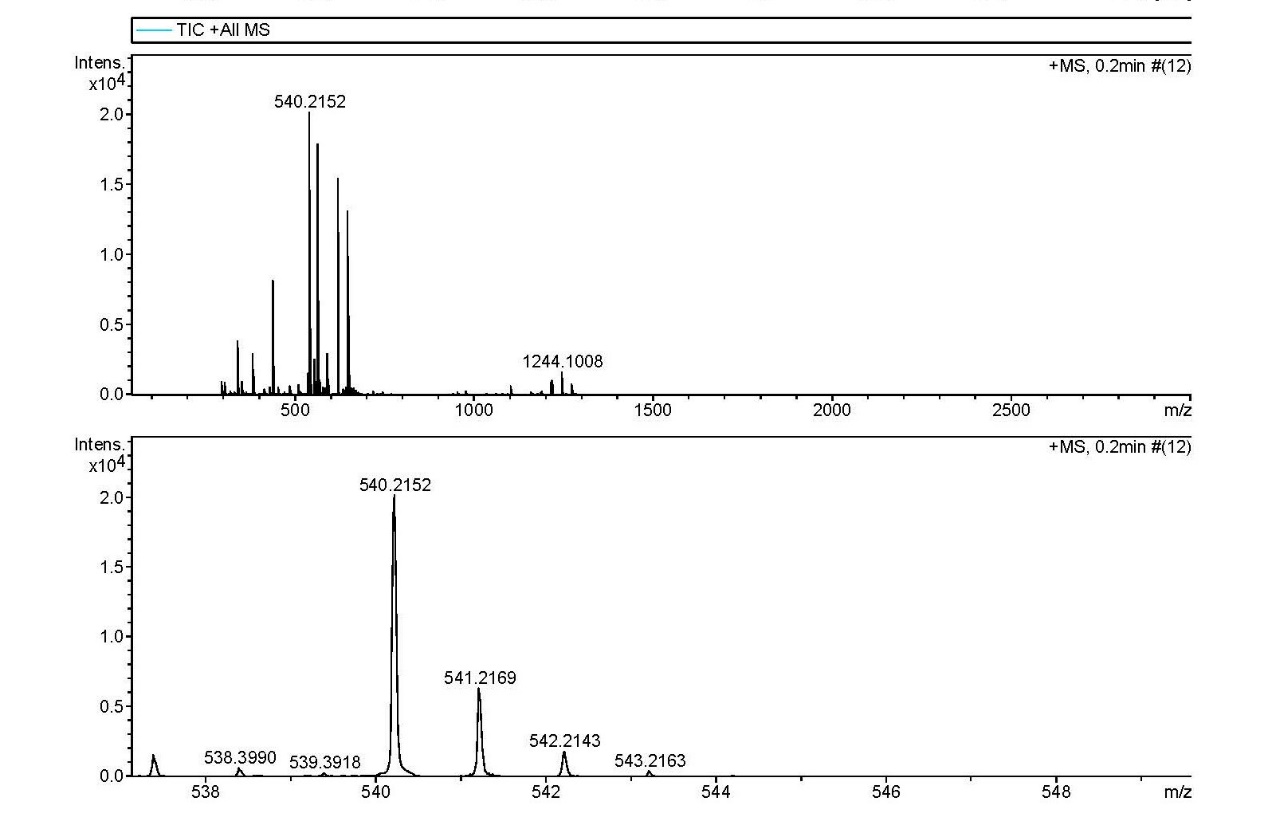
**

**Fig. S82.** HR MS Spectrum of compound **26b**


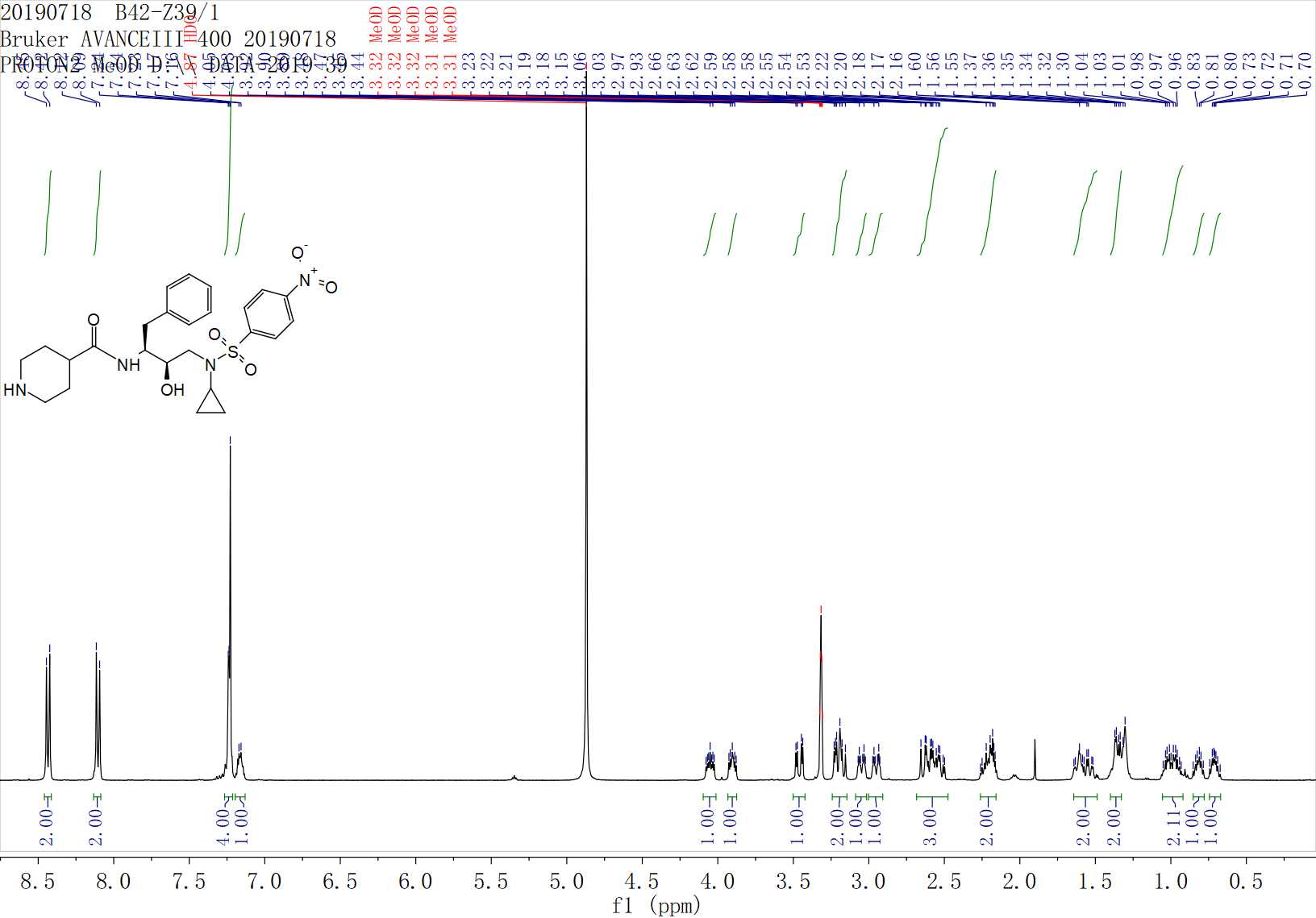


**Fig. S83.** ^1^H NMR Spectrum of compound **26c**


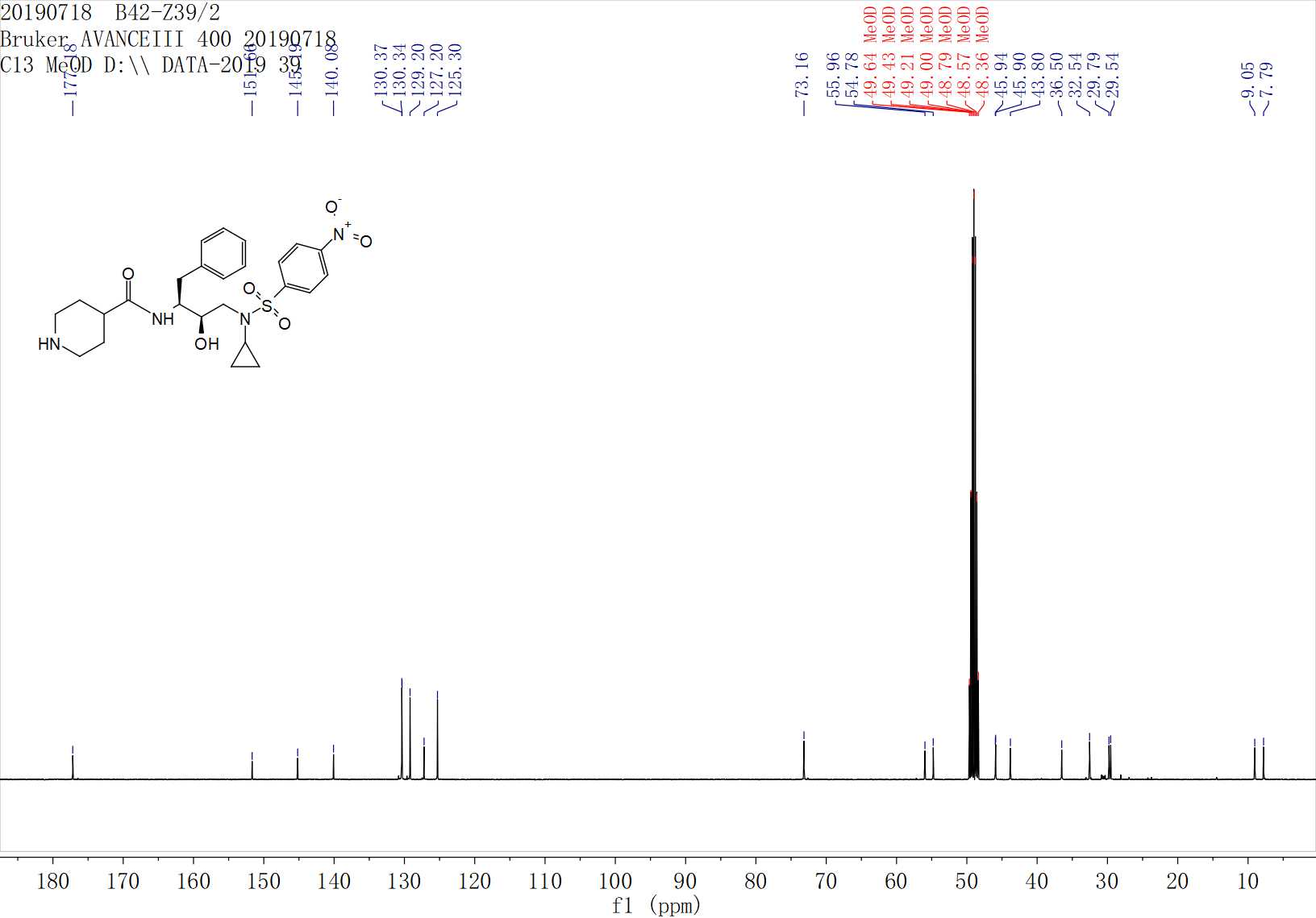


**Fig. S84.** ^13^C NMR Spectrum of compound **26c**

**
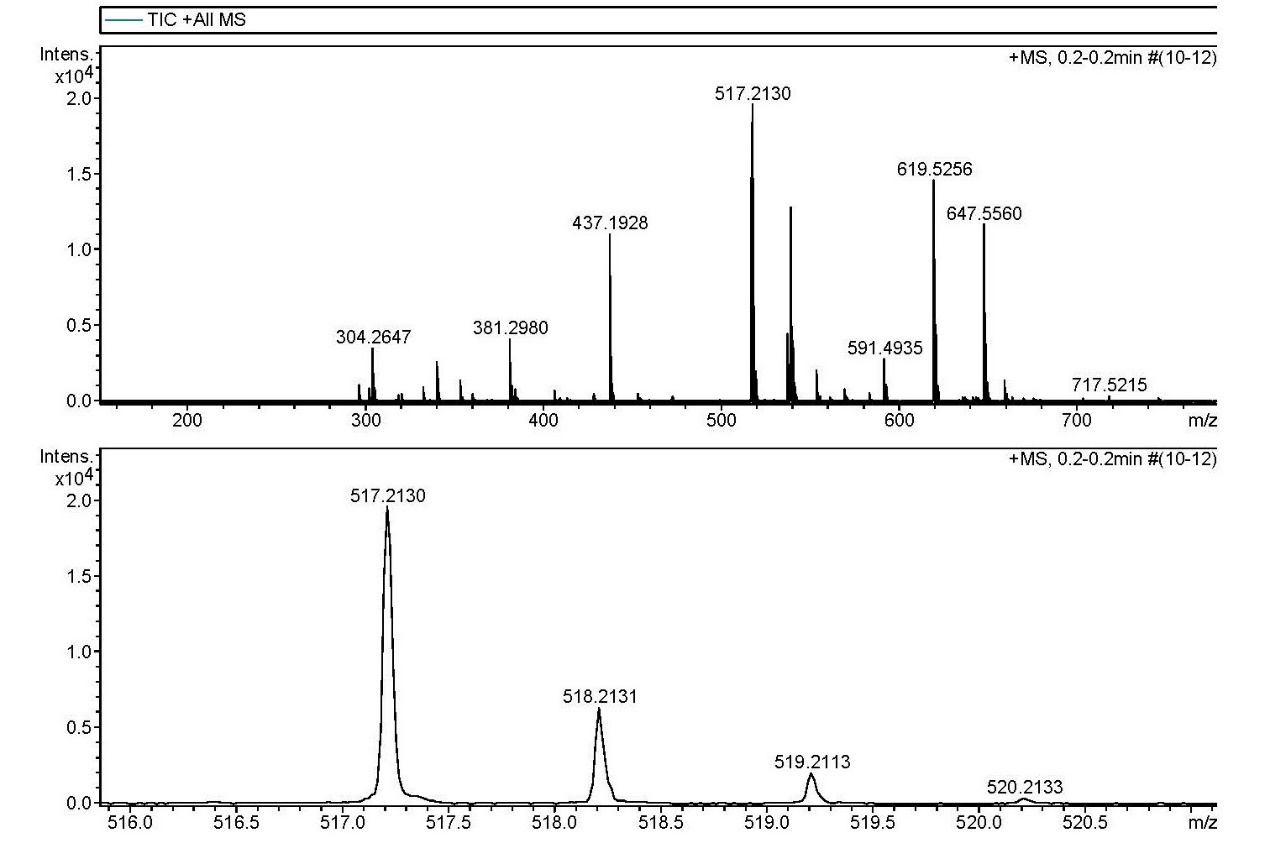
**

**Fig. S85.** HR MS Spectrum of compound **26c**


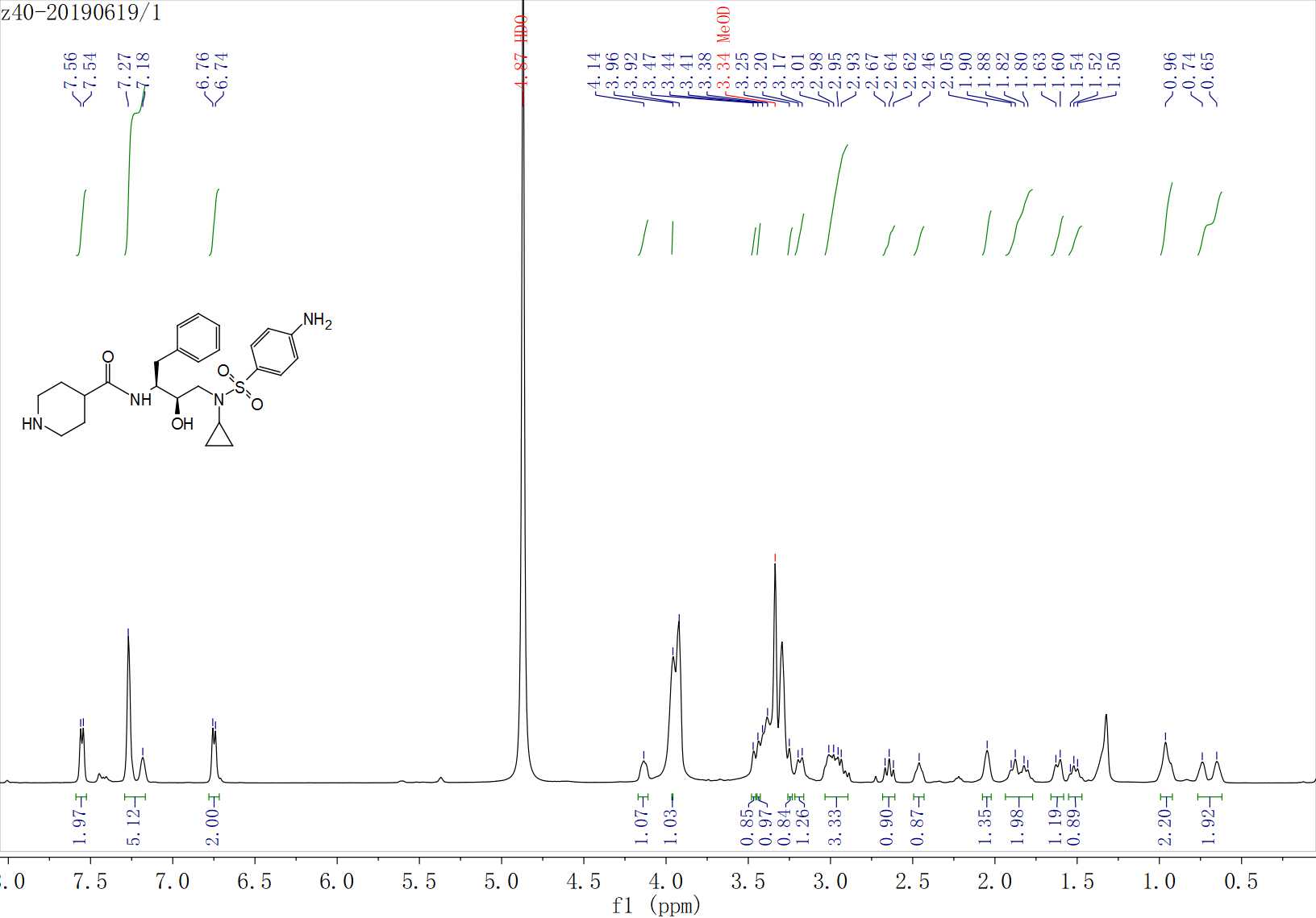


**Fig. S86.** ^1^H NMR Spectrum of compound **26d**


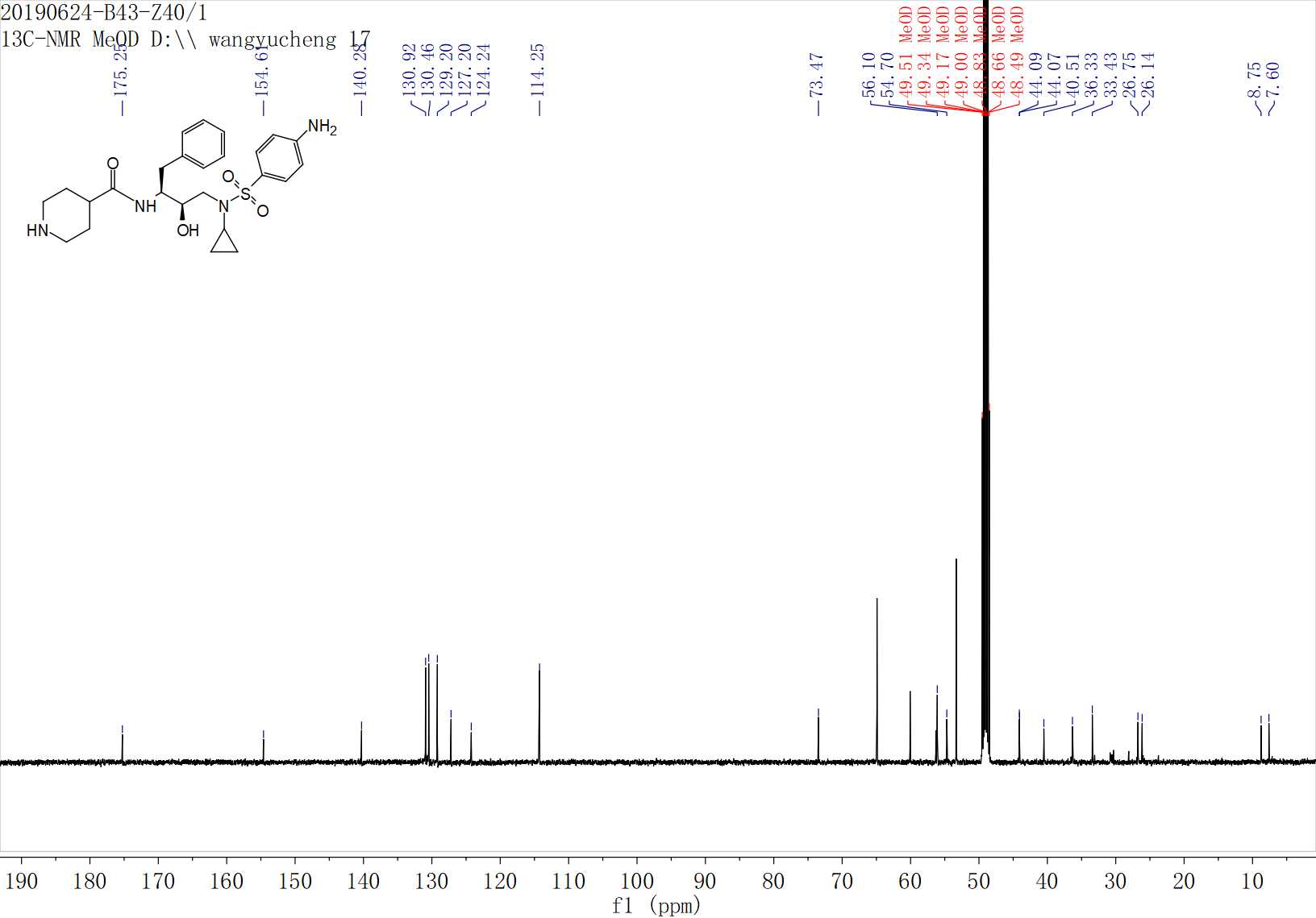


**Fig. S87.** ^13^C NMR Spectrum of compound **26d**

**
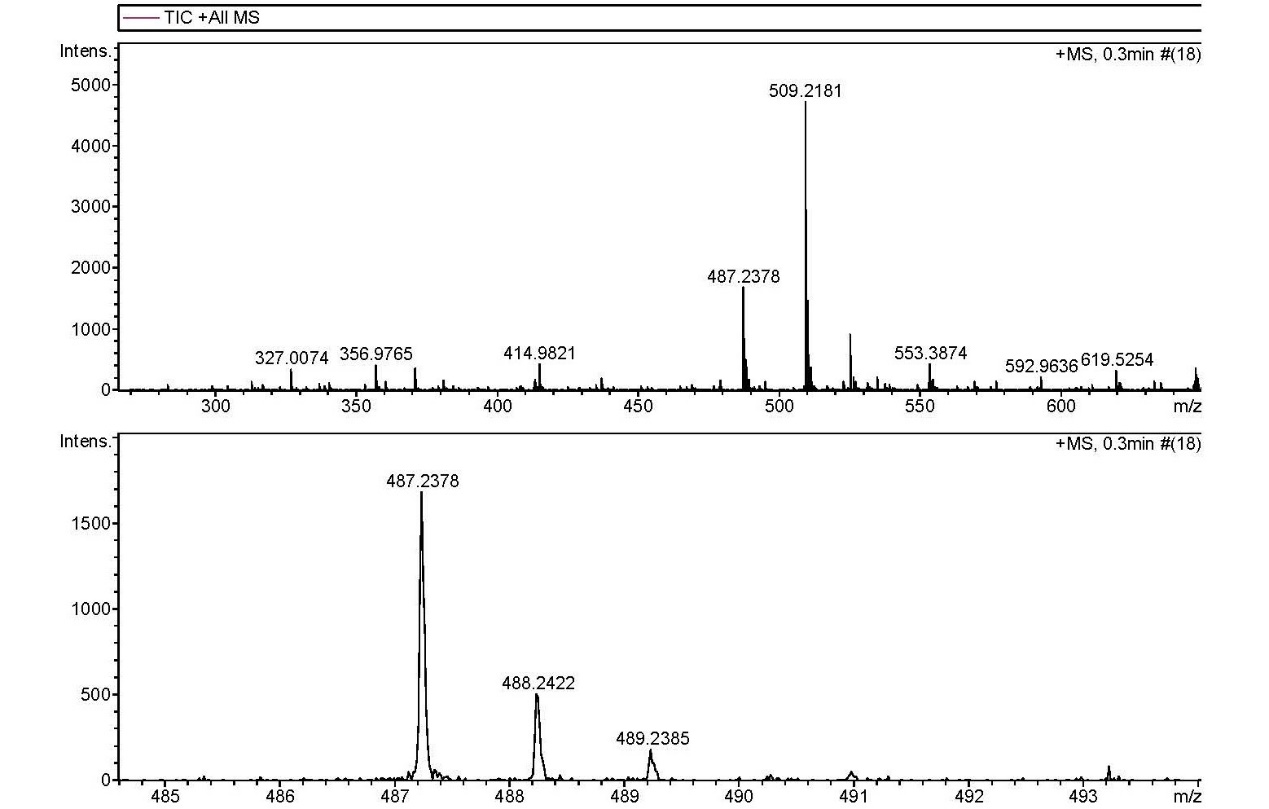
**

**Fig. S88.** HR MS Spectrum of compound **26d**


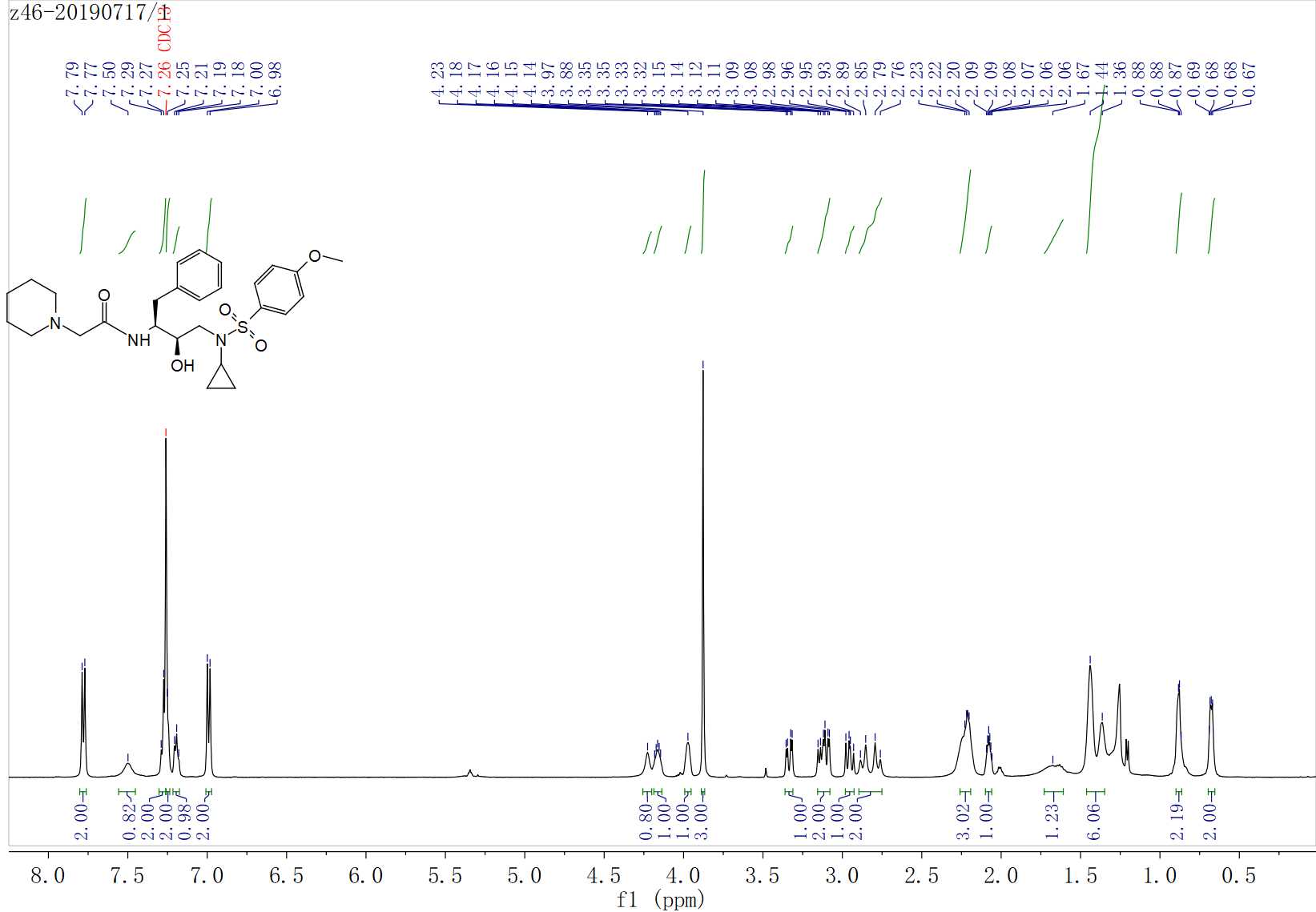


**Fig. S89.** ^1^H NMR Spectrum of compound **27a**


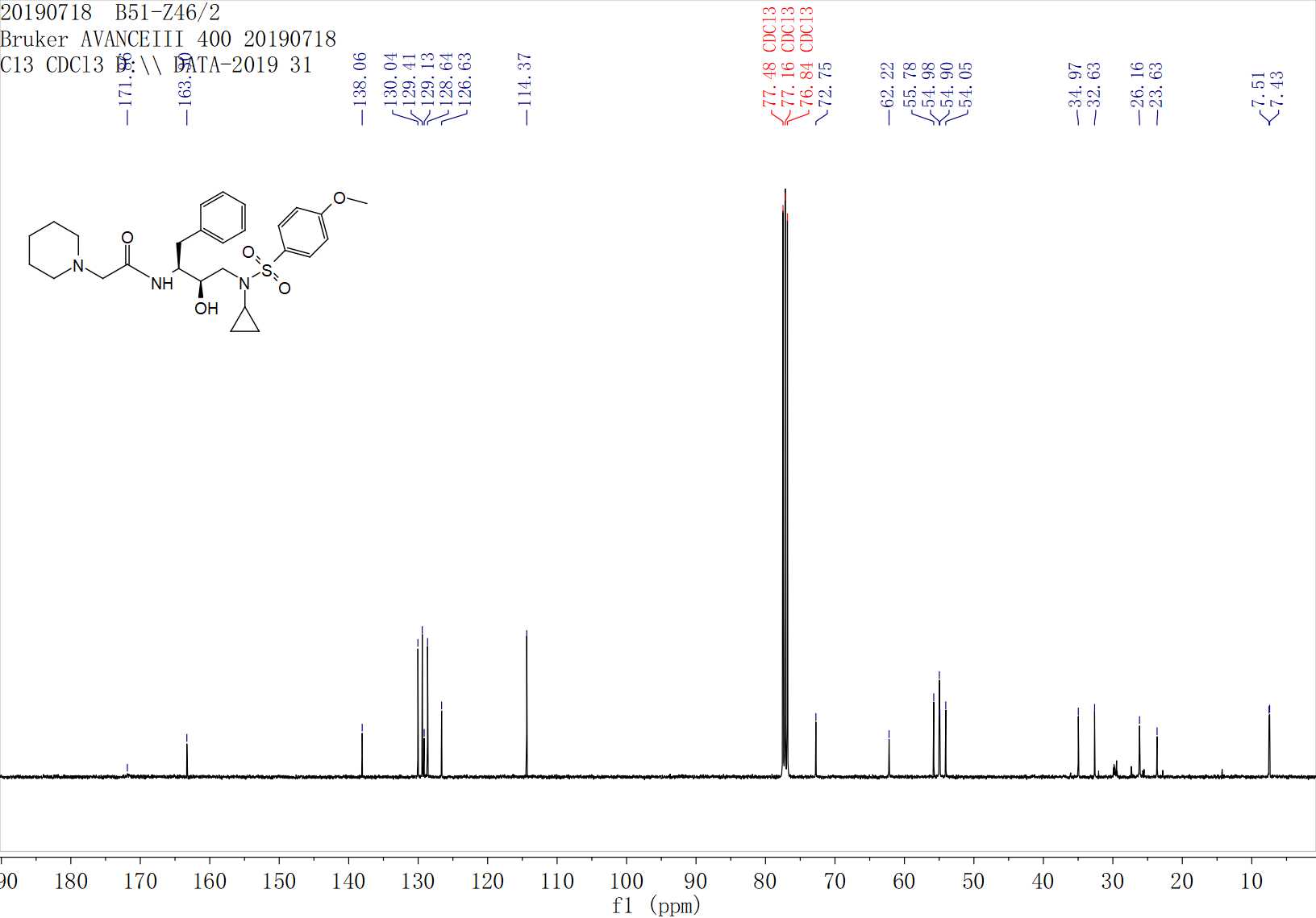


**Fig. S90.** ^13^C NMR Spectrum of compound **27a**

**
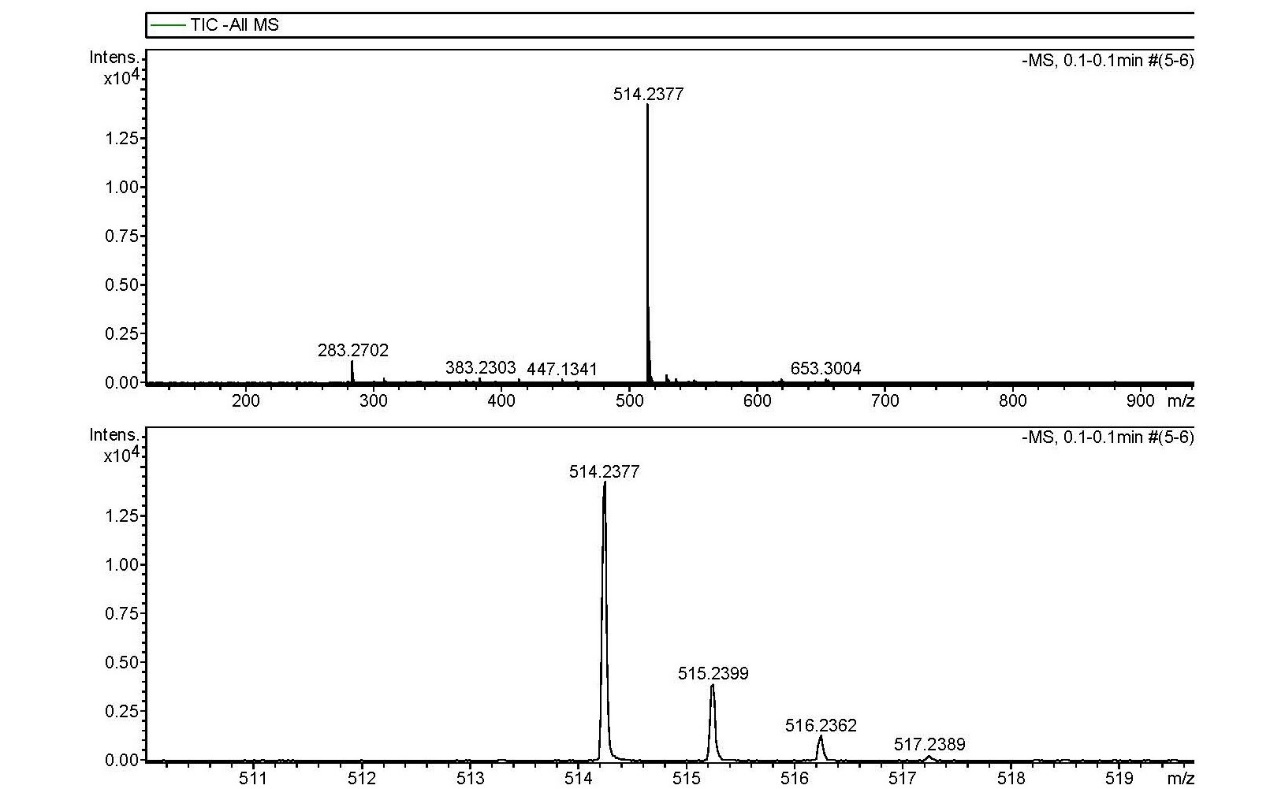
**

**Fig. S91.** HR MS Spectrum of compound **27a**


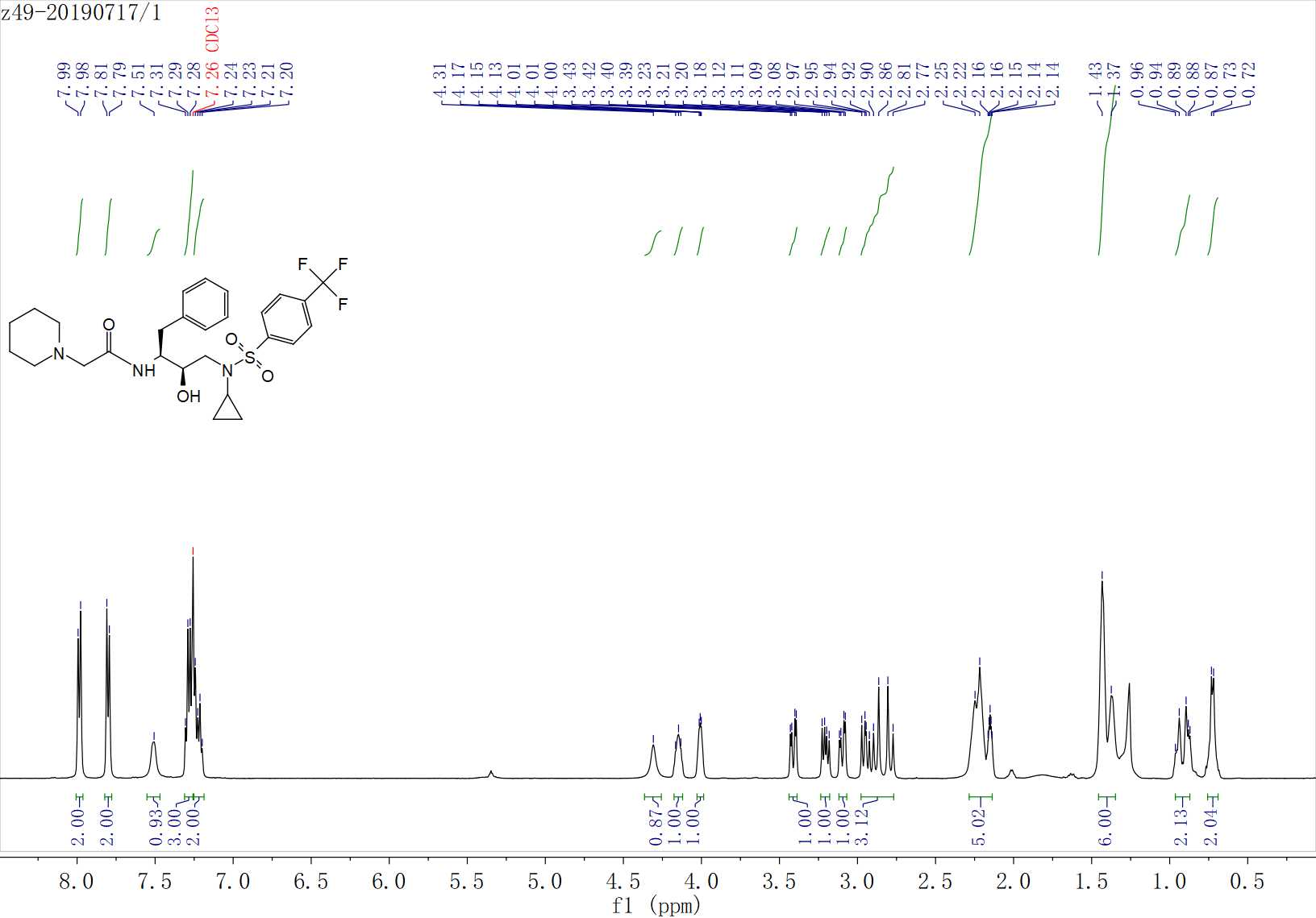


**Fig. S92.** ^1^H NMR Spectrum of compound **27b**


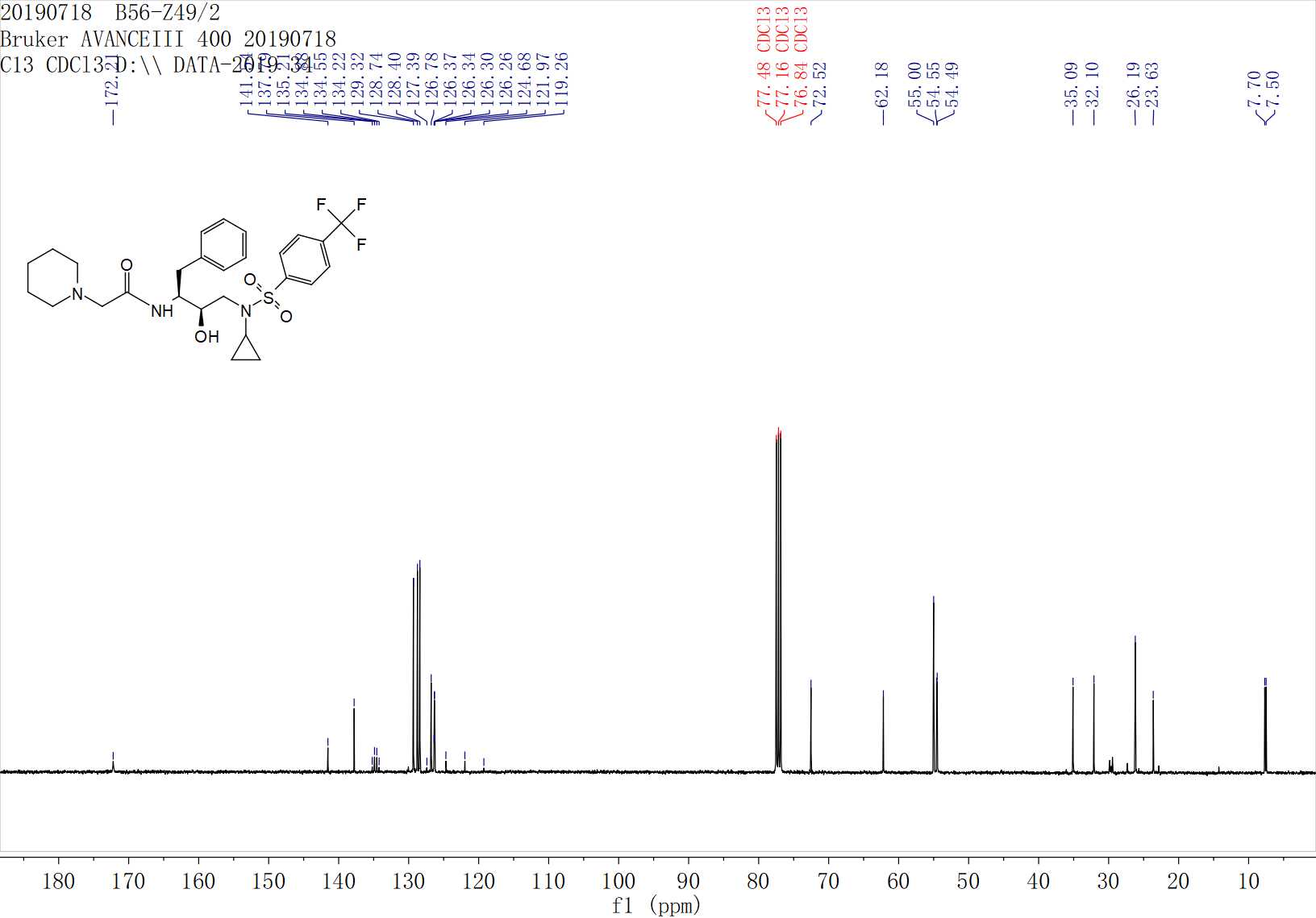


**Fig. S93.** ^13^C NMR Spectrum of compound **27b**

**
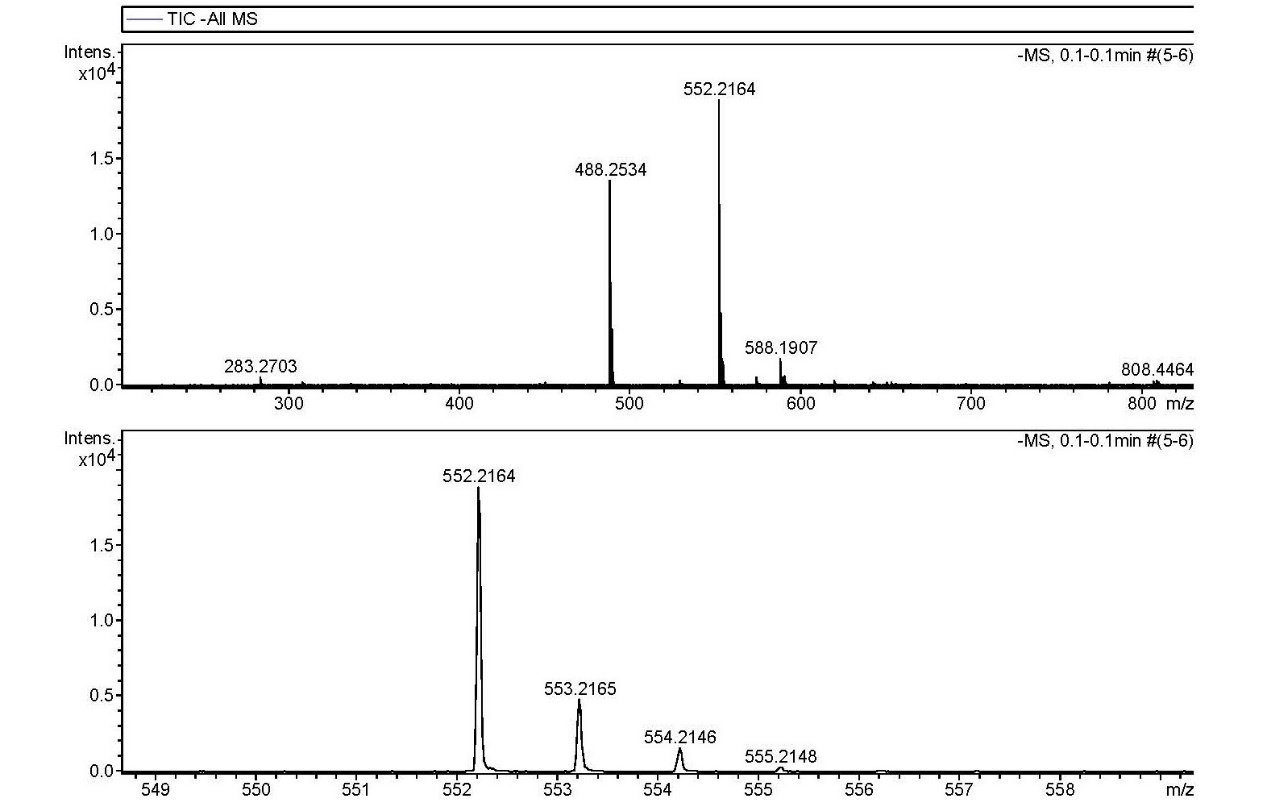
**

**Fig. S94.** HR MS Spectrum of compound **27b**


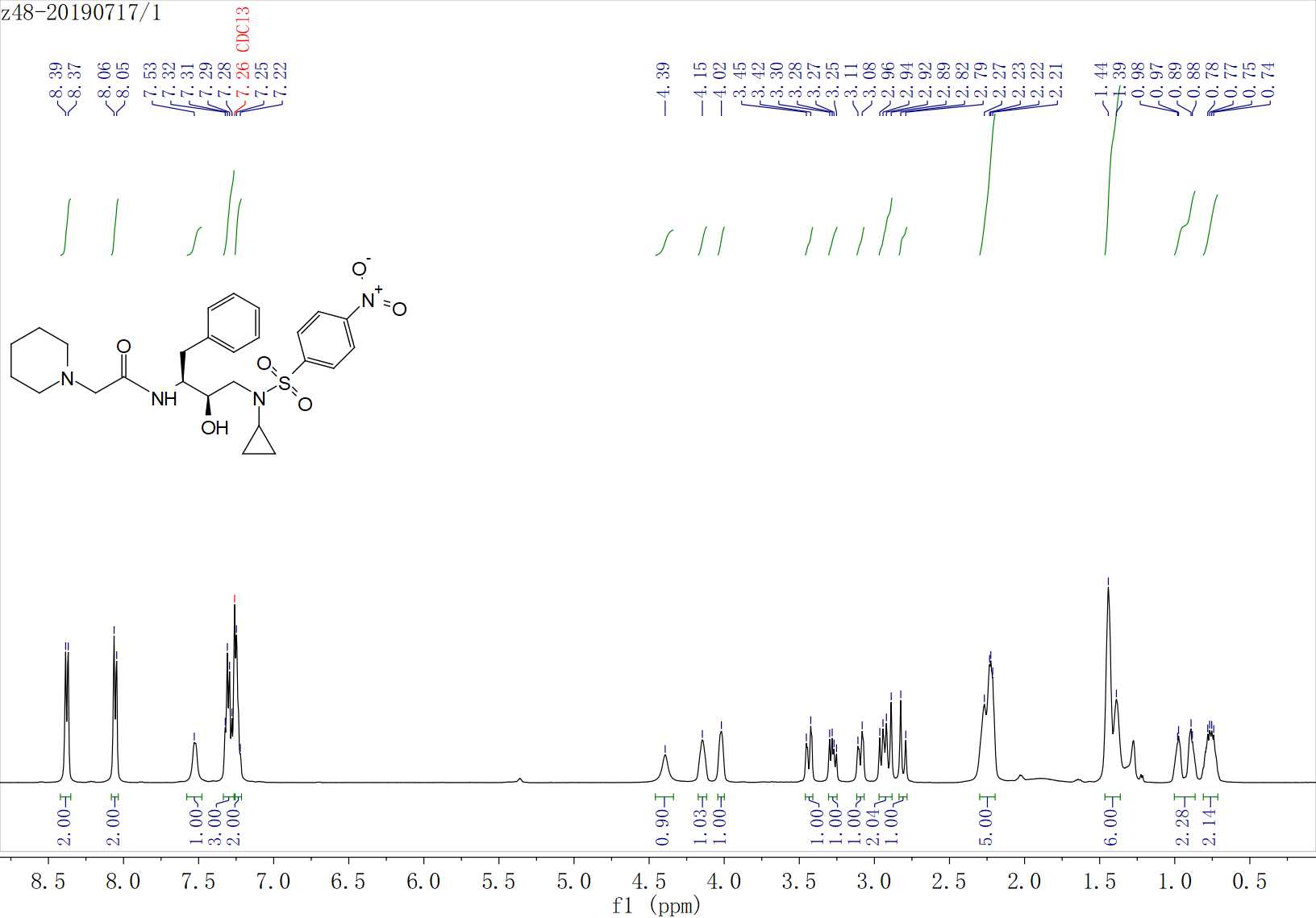


**Fig. S95.** ^1^H NMR Spectrum of compound **27c**


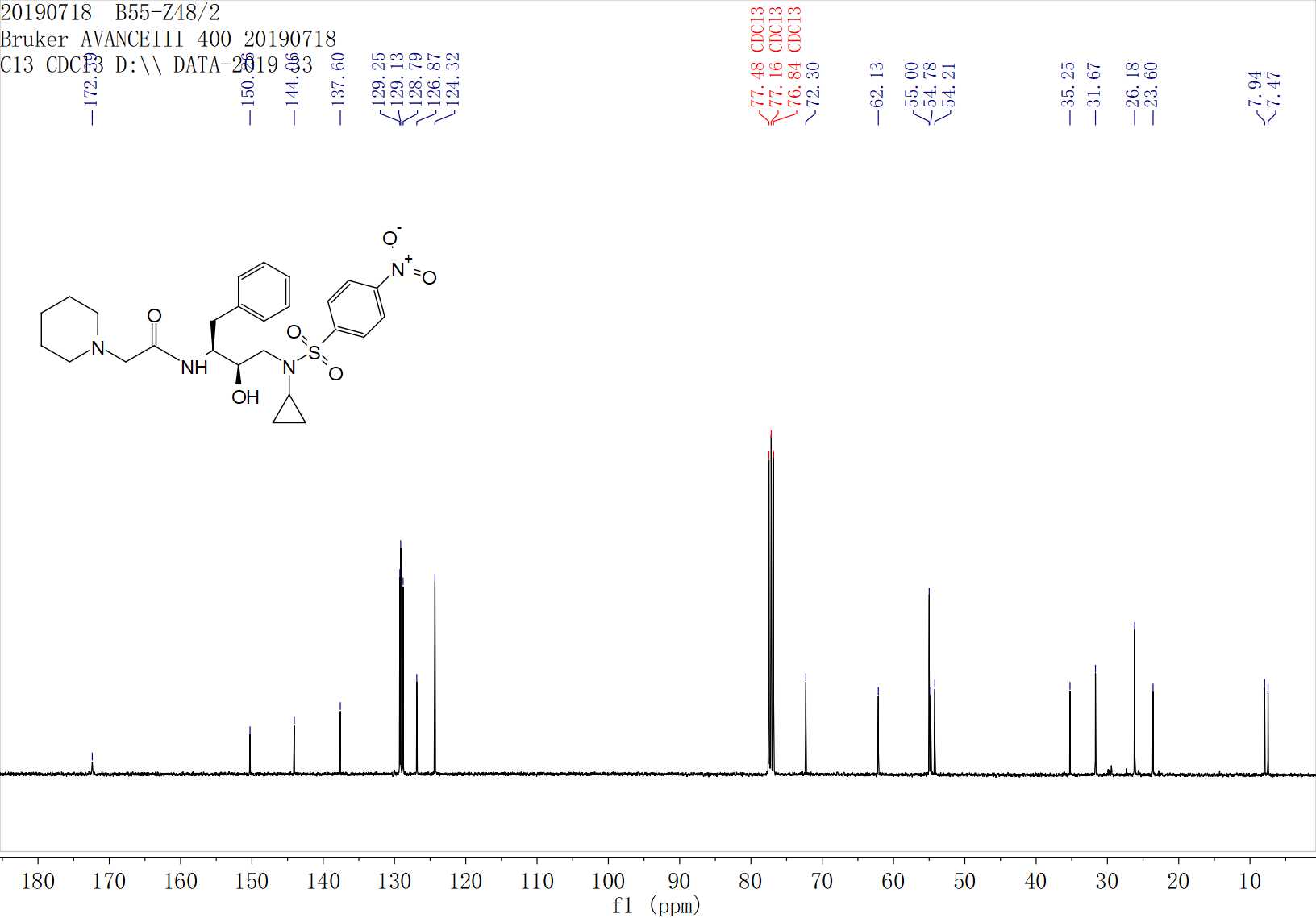


**Fig. S96.** ^13^C NMR Spectrum of compound **27c**

**
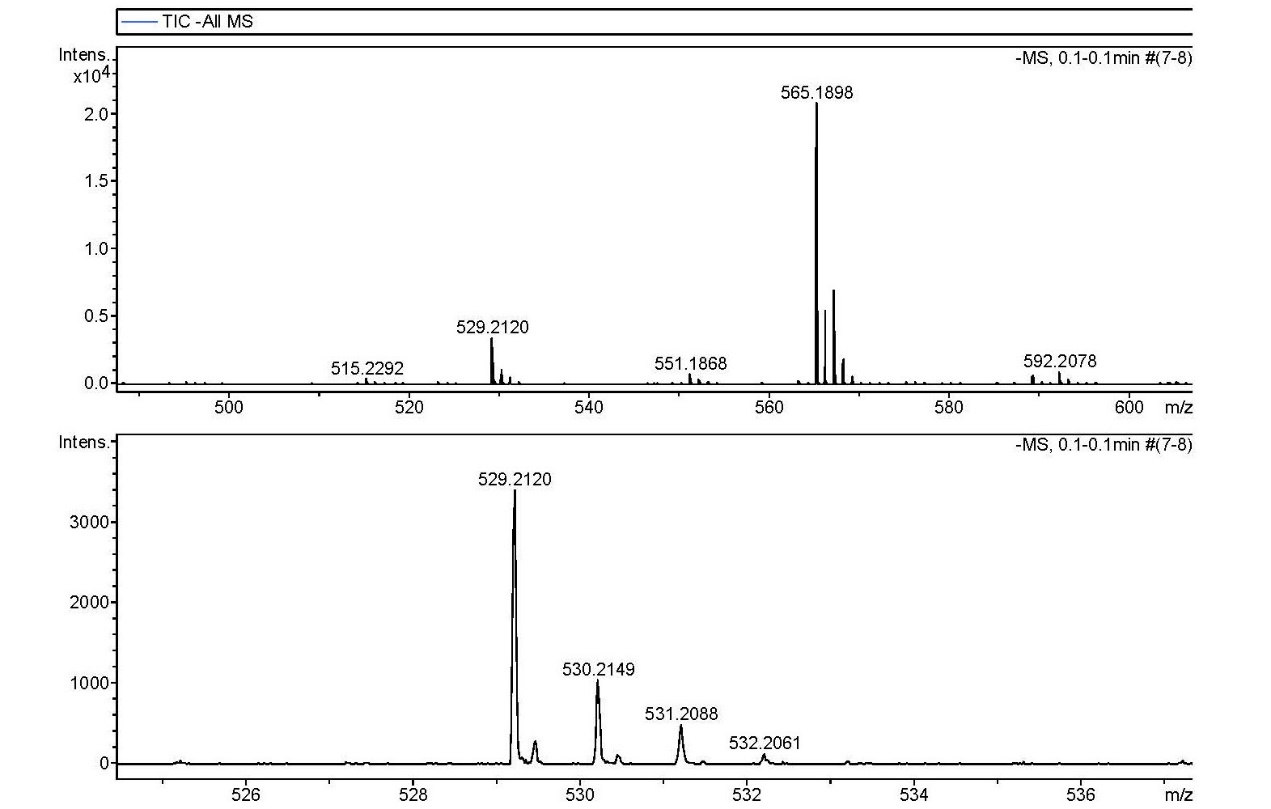
**

**Fig. S97.** HR MS Spectrum of compound **27c**


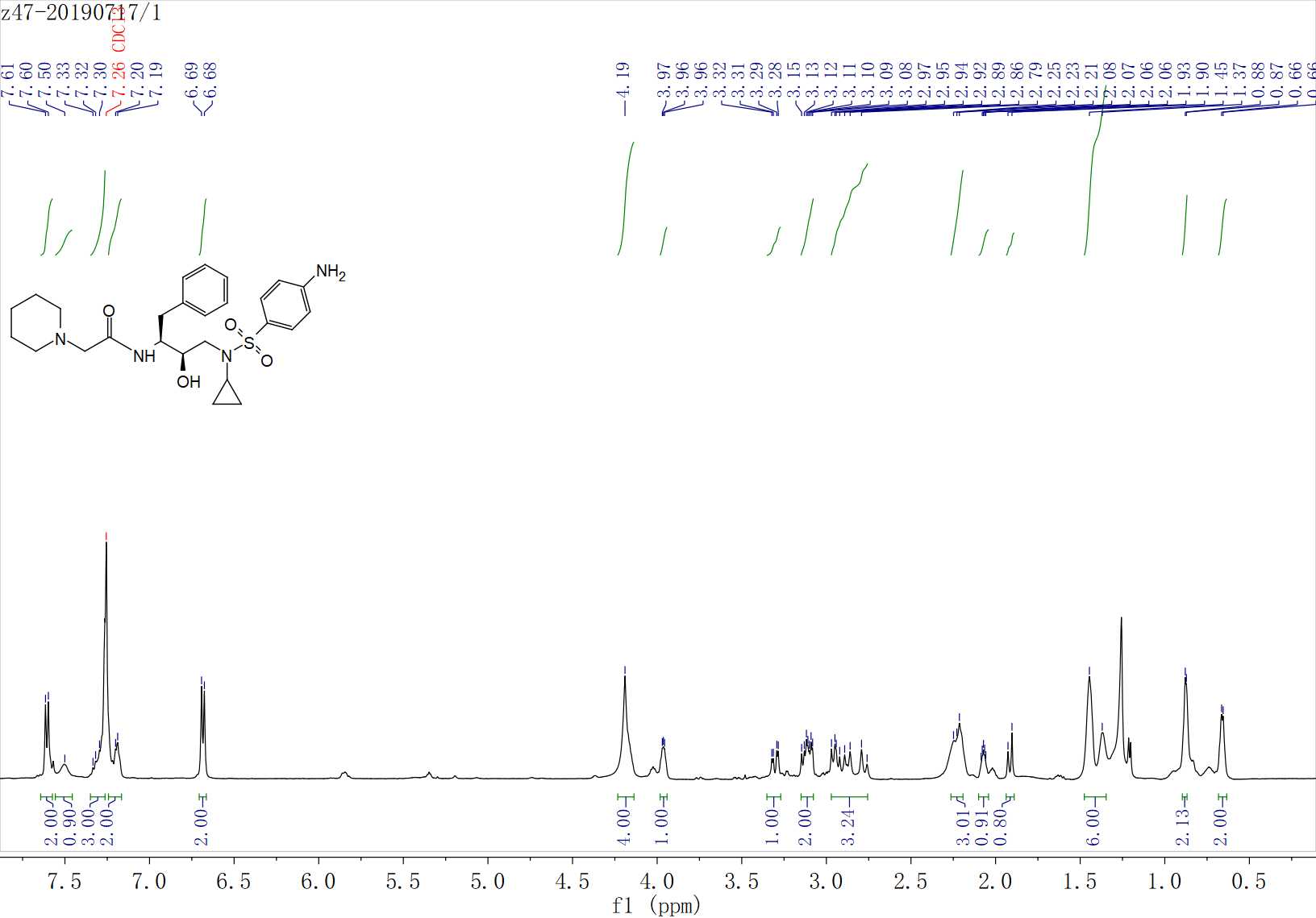


**Fig. S98.** ^1^H NMR Spectrum of compound **27d**


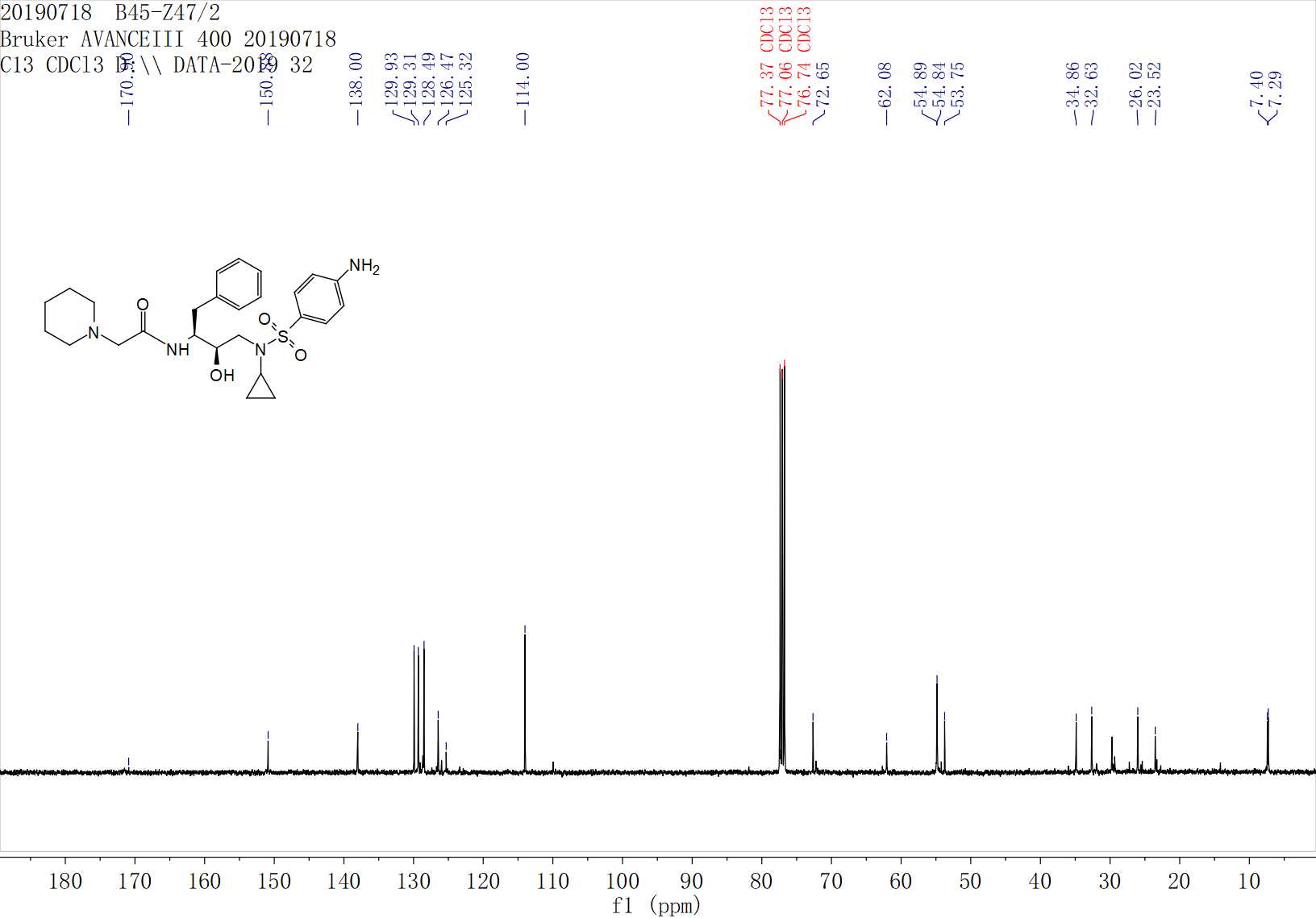


**Fig. S99.** ^13^C NMR Spectrum of compound **27d**

**
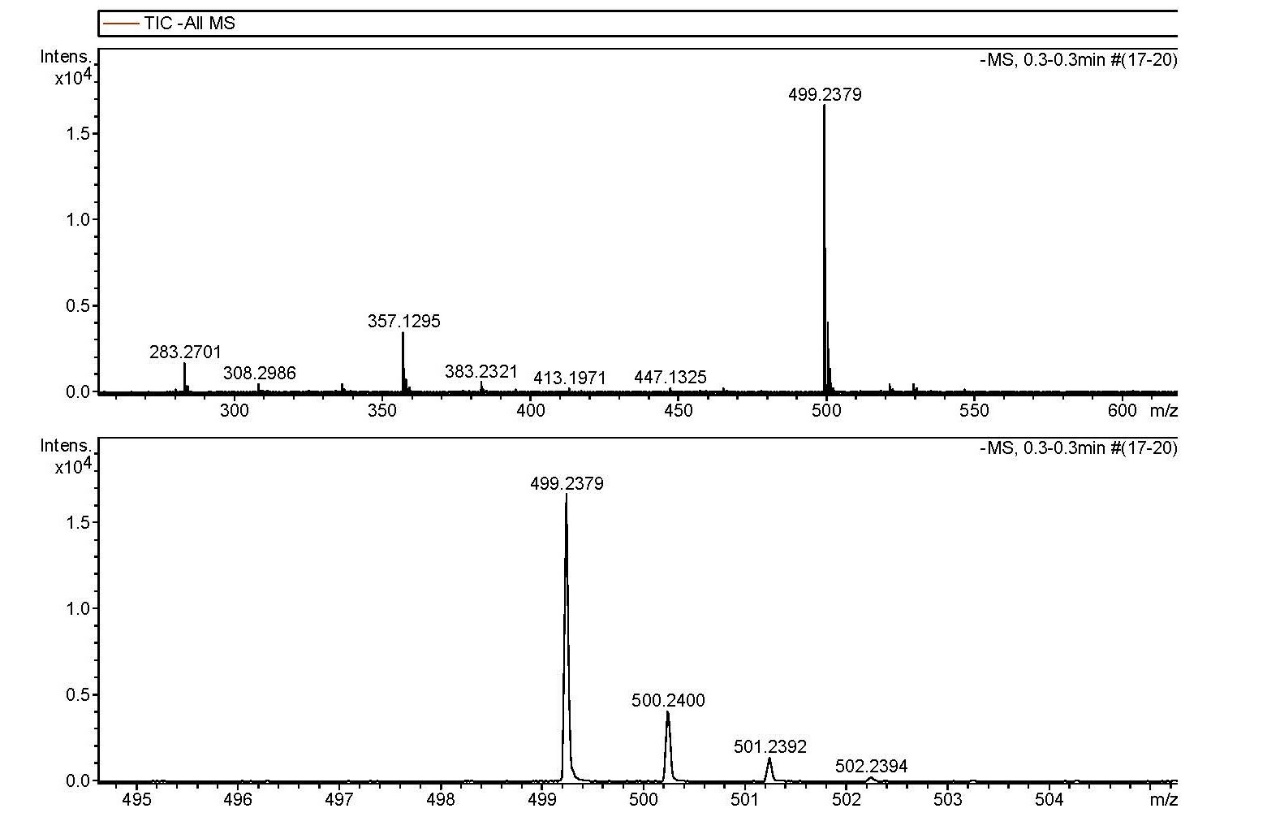
**

**Fig. S100.** HR MS Spectrum of compound **27d**
