# Supplementary material for: Piperidine scaffold as the novel P2-ligands in cyclopropyl-containing HIV-1 protease inhibitors: Structure-based design, synthesis, biological evaluation and docking study
Source: PLoS One. 2020 Jul 22;15(7):e0235483. doi: 10.1371/journal.pone.0235483 (PMC7375528; doi:10.1371/journal.pone.0235483)
Supplement: S3 Appendix — (DOCX) [file pone.0235483.s003.docx]

**S3 Appendix. Description of biological evaluation and docking study.**

**Ⅰ. *In vitro* HIV-1 PR activity assay**

The inhibitory effect of all new designed inhibitors were measured using fluorescence resonance energy transfer (FRET) method. Peptide (Arg-Glu (EDANS)-Ser-Gln-Asn-Tyr-Pro-Ile-Val-Gln- Lys(DABCYL)-Arg) purchased from AnaSpec was selected as the substrate. The energy transfer donor (EDANS) and acceptor (DABCYL) dyes are labeled at two ends of the peptide to perform FRET. Excitation and emission wavelengths were set at 340 nm and 490 nm. Inhibitors were dissolved in dimethylsulfoxide (DMSO) and diluted to appropriate concentrations. HIV-1 protease was cloned and heterologously expressed in Escherichia coli and purified. The experiment was carried out in 96-well plates. The FRET assay reaction buffer contained 0.1 M sodium acetate, 1 M sodium chloride, 1 mM ethylenediaminetetraacetic acid (EDTA), 1 mM dithiothreitol (DTT), 2% DMSO and 1 mg/mL bovine serum albumin (BSA) with an adjusted pH 4.7. Protease and inhibitor were mixed and incubated for 20**-**30 mins at room temperature and then the substrate was added. Each reaction was recorded for about 10 mins.

**Ⅱ. HIV-1 infectivity assay**

The inhibitory effect of compounds on HIV-1 infectivity were determined using a single-round HIV-1 infectivity assay. 293T cells were co-transfected with either plasmid pNL4-3-E^-^R^-^ (pHIV-1_NL4-3_) or DRV-resistant pNL4-3-E^-^R^-^ variants (pHIV-1_DRV_^R^_S_) and pHCMV-G (VSV-G) to produce VSV-G pseudotyped HIV-1. Inhibitors dissolved in dimethylsulfoxide (DMSO) and diluted to appropriate concentrations, were added into culture medium at 5 hours of post-transfection. After incubating for 48 hours at temperature 37 °C, pseudotyped viruses in 10 μL of supernatant were used to infect SupT1 cells for 48 hours, followed by measuring luciferase activity of newly infected cells using Centro LB960 (Berthold).

For the assay using wild type HIV-1, 1×10^6^ SupT1 cells were infected with 100 μL HIV-1 NL4-3 in the presence of 100 nM chemicals and 10 μg/mL polybrene, keeping a total volume of 500 μL (Spin infection at 1800rpm, 45min). Cells were washed once in the next morning and medium were replaced with fresh medium containing 100 nM chemicals. At 48 hpi, viruses were harvested and 50 μL of viruses were used to infect TZM-bl cells, followed by measuring luciferase activity in the infected cells.

**Ⅲ. Cytotoxicity Assay**

Selected inhibitors were further evaluated in cytotoxicity assay using a cell counting kit-8 assay. Plates were prepared with 20 000 293T cells per well. After 24h of culture, 1μL of drugs were added to each well. After another 24h of culture, 10 μL of CCK-8 was added to each well. Absorbance was quantified at wavelength 450 nm using an EnVision multilabel reader (PerkinElmer) after 2h at room temperature.

**Ⅳ. Molecular docking**

In general, the docking was performed through “DOCK” module in the Molecular Operating Environment (MOE) using the alpha triangle placement method. Refinement of the docked poses was carried out using the Forcefield refinement scheme and scored using both the affinity dG and london dG scoring system. The pose with the higher docking negative score implied better binding.
